# Bioinspired Electrochemical Cyclization toward the Divergent Synthesis of Mavacurane- and Akuammiline-Type Alkaloids

**DOI:** 10.1021/acs.orglett.5c03645

**Published:** 2025-10-14

**Authors:** Eisuke Sato, Tomohiro Nakahama, Yuika Nomura, Koichi Mitsudo, Seiji Suga

**Affiliations:** Department of Applied Chemistry, Graduate School of Environmental, Life, Natural Science and Technology, 12997 Okayama University, 3-1-1, Tsushima-naka, Kita-ku, Okayama, 700-8530, Japan

## Abstract

We report a divergent electrochemical strategy for the bioinspired synthesis of mavacurane- and akuammiline-type alkaloid frameworks from a common indole–malonate precursor. By tuning the redox mediator, selective N–C or C–C bond formation was achieved. Iodide-mediated electrolysis promoted iodination of the malonate carbanion, followed by intramolecular nucleophilic cyclization to furnish the mavacurane core. In contrast, ferrocene-mediated oxidation generated a malonate-centered radical that afforded the akuammiline skeleton.

## Graphical abstract

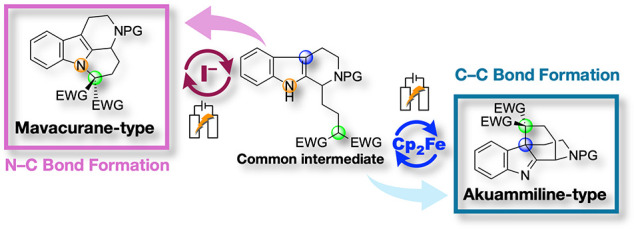

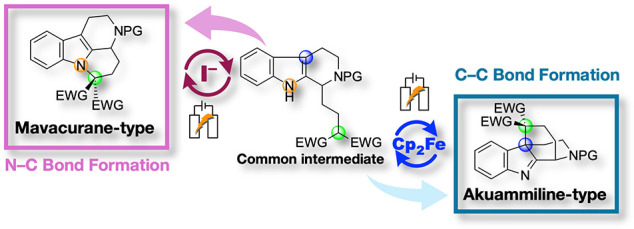

Monoterpene indole alkaloids comprise one of the largest families of natural products, and more than 3000 of these compounds have been isolated from various plant species.^1–3^ These alkaloids exhibit a wide range of structural diversity and biological activities. In biosynthesis, structural diversity is established through the oxidative cyclization of a single biosynthetic intermediate, strictosidine (**1**).^1^ Following the deglycosylation of **1**, a cascade of enzyme-catalyzed transformations, including intramolecular oxidative coupling, gives rise to a vast number of complex scaffolds (Scheme 1A). These downstream modifications are tightly regulated by enzyme specificity, contributing to the remarkable chemical diversity observed in nature.

**Scheme 1:**
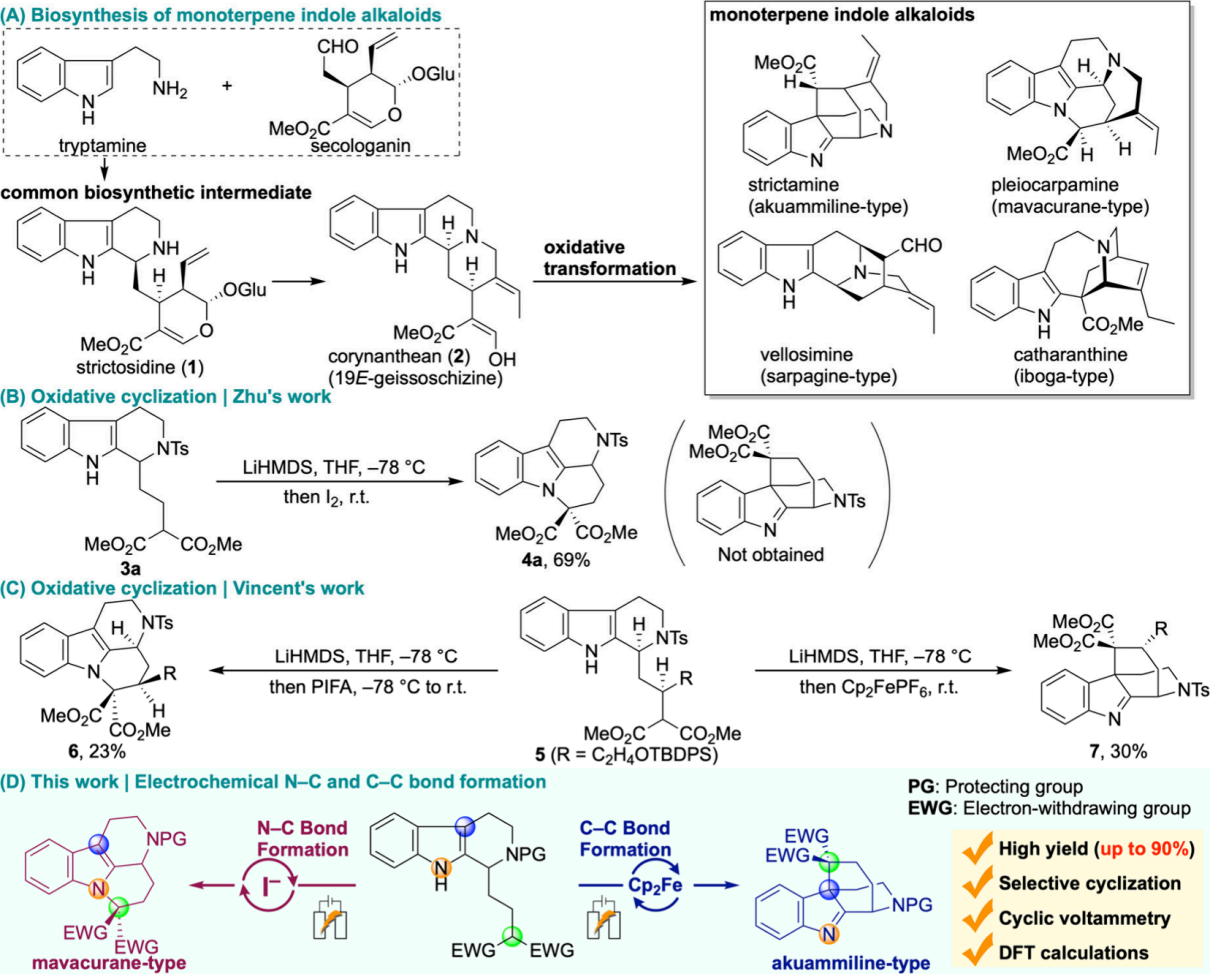
Biosynthesis of Monoterpene Indole Alkaloids and Bioinspired Oxidative Cyclization

Over the past decades, oxidative dianion coupling has been considered a novel tool for the C–C bond formation, and the groups of Baran,^4^ Overman,^5^ and Ma^6^ have developed total syntheses of indole alkaloids through a oxidative strategy. Building on the biosynthetic insight, Zhu reported an oxidative cyclization of a simplified cyclization precursor **3a**.^7^ Deprotonation of the 1,3-dicarbonyl moiety followed by I_2_ oxidation induced N–C bond formation, which gave the characteristic mavacurane framework (Scheme 1B). A subsequent study achieved both N–C and C–C bond formation to access five-membered rings.^8^ Whereas *N*-iodosuccinimide (NIS) led to N–C bond formation, use of Koser’s reagent (PhI­(OH)­OTs) promoted C–C bond formation. Vincent later explored a bioinspired oxidative cyclization to construct both the mavacurane and akuammiline scaffolds (Scheme 1C).^9^ Upon double deprotonation of the malonate and the indole moieties, subsequent addition of iodobenzene bis­(trifluoroacetate) (PIFA) favored N–C bond formation, whereas single-electron oxidation with Cp_2_FePF_6_ selectively generated the akuammiline core.

While bioinspired oxidative cyclizations have provided elegant strategies for constructing the architecturally complex skeletons, these methods suffer from significant limitations derived from the reliance on stoichiometric amounts of a strong base and oxidant. To overcome these challenges, we turned to electrochemical oxidation as an alternative activation mode. Electrochemical synthesis offers a tunable platform for redox transformations that proceed without the need for external oxidants or reductants.^10–14^ This operational simplicity has led to a surge of interest in electrochemical methods for natural product synthesis and complex molecule construction.^15–17^

Of particular relevance to our study is the concept of indirect electrolysis.^18–20^ This approach offers key advantages in terms of selectivity and functional group tolerance.^21,22^ A wide array of redox mediators—halide ions,^23^ ferrocene (Cp_2_Fe) derivatives,^24,25^ triarylamines^26^ and hypervalent iodines^27,28^ —have been used. Notably, the oxidation behavior of 1,3-dicarbonyl compounds was found to be dependent on the nature of the redox mediator.^29^ Zhang and Huang reported that iodide-mediated electrolysis enables selective N–C bond formation,^30^ while Xu demonstrated that Cp_2_Fe catalyzes the generation of carbon-centered radicals from 1,3-dicarbonyl substrates, enabling C–C bond-forming reactions.^31,32^ Most recently, Guo developed nickel-catalyzed enantioselective dehydrogenative coupling using Cp_2_Fe as an electrocatalyst.^33^

Building upon these precedents, we envisioned that electrochemical activation could be harnessed to achieve divergent oxidative cyclizations from a common intermediate, mimicking biosynthetic logic. Specifically, we demonstrate that iodide-mediated electrolysis affords mavacurane frameworks (Scheme 1D). In contrast, the use of Cp_2_Fe switches the selectivity, enabling access to akuammiline-type frameworks.

We began our investigation by exploring the anodic oxidation of substrate **3a** using iodide as a redox mediator (Table 1). Drawing upon our previous studies^19,20^ and the precedent established by Zhang and Huang,^30^ the reaction was conducted using Bu_4_NI and LiOMe in CH_3_CN/MeOH as the electrolyte components. Electrolysis with platinum electrodes smoothly delivered desired mavacurane-type product **4a** in an excellent 93% yield (entry 1). Substitution with LiI instead of Bu_4_NI afforded **4a** in only 20% yield, along with the iodinated side product **9** in 44% yield (entry 2). We next assessed the effect of the halide identity on this cyclization. Use of bromide as the mediator provided **4a** in a moderate yield (entry 3), while chloride failed to promote productive cyclization (entry 4). Electricity was essential for the cyclization to proceed, and the control experiment conducted without electricity resulted in the recovery of the starting material (entry 5).

**Table 1:**
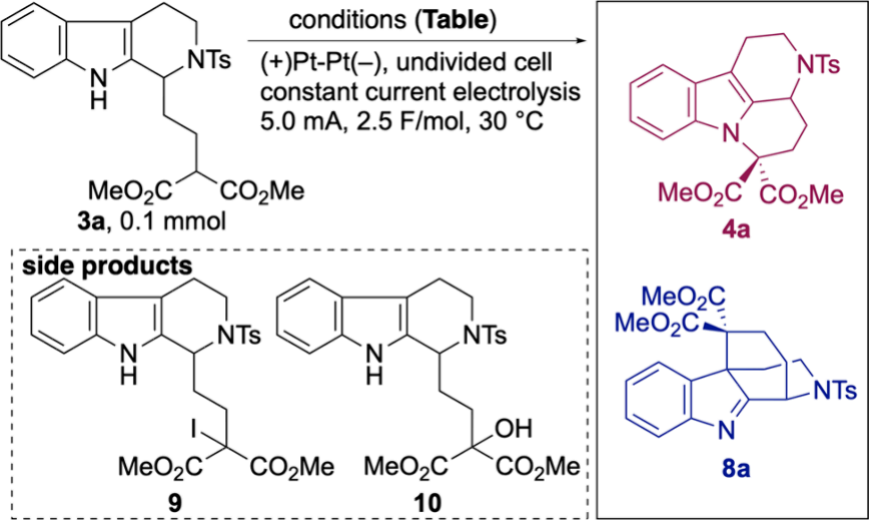
Anodic Cyclization toward Mavacurane and Akuammiline Skeleton

entry	conditions	result
1	Bu_4_NI, 0.1 mmol/LiOMe, 0.05 mmol	**4a**, 93%
	CH_3_CN/MeOH = 9:1 (5.0 mL)	
2	LiI, 0.1 mmol/LiOMe, 0.05 mmol	**4a**, 20%
	CH_3_CN/MeOH = 9:1 (5.0 mL)	**9**, 44%
3	Bu_4_NBr, 0.1 mmol/LiOMe, 0.05 mmol	**4a**, 68%
	CH_3_CN/MeOH = 9:1 (5.0 mL)	
4	Bu_4_NCl, 0.1 mmol/LiOMe, 0.05 mmol	**4a**, ND^a^
	CH_3_CN/MeOH = 9:1 (5.0 mL)	
5	Bu_4_NI, 0.1 mmol/LiOMe, 0.05 mmol	NR^b^
	CH_3_CN/MeOH = 9:1 (5.0 mL)	
	No electricity	
6	Cp_2_Fe, 0.1 mmol/LiOMe, 0.05 mmol	**8a**, trace
	Bu_4_NBF_4_, 0.5 mmol	
	THF/MeOH = 9:1 (5.0 mL)	
7	Cp_2_Fe, 0.1 mmol/LiO*t*-Bu, 0.05 mmol	**8a**, 57%
	Bu_4_NBF_4_, 0.5 mmol	
	THF/*t*-BuOH = 9:1 (5.0 mL)	
8	Cp_2_Fe, 0.1 mmol/LiO*t*-Bu, 0.05 mmol	**8a**, 94%
	Bu_4_NBF_4_, 0.5 mmol	
	THF/HFIP = 9:1 (5.0 mL)	
9	Cp_2_Fe, 0.1 mmol/LiO*t*-Bu, 0.05 mmol	**8a**, <9%
	Bu_4_NBF_4_, 0.5 mmol	**10**, <23%
	THF/HFIP = 9:1 (5.0 mL), under O_2_	
10	Cp_2_Fe, 0.1 mmol/LiO*t*-Bu, 0.05 mmol	NR^c^
	Bu_4_NBF_4_, 0.5 mmol	
	THF/HFIP = 9:1 (5.0 mL)	
	No electricity	

a Not detected.

b No reaction, 84% recovery.

c No reaction, 98% recovery.

Having established efficient conditions for the construction of the mavacurane framework, we next turned our attention to the synthesis of the akuammiline scaffold. Inspired by Vincent^9^ and Xu’s^31,32^ work, we carried out the anodic oxidation of **3a** using Cp_2_Fe and LiOMe in THF/MeOH (entry 6). Although ^1^H NMR analysis of the reaction mixture confirmed the formation of the desired C–C bond, numerous decomposition products were also observed. We hypothesized that methoxide may nucleophilically attack the C2 or C3 position of indole in the cation intermediates (*vide infra*) or the imine moiety in **8a**. Subsequent degradation of the methanol adduct would lower the isolated yield of **8a**.

To address this issue, we investigated the combination of less nucleophilic bases and alcohols. Gratifyingly, LiO*t*-Bu/*t*-BuOH afforded **8a** in 57% yield (entry 7). Furthermore, replacing *t*-BuOH with 1,1,1,3,3,3-hexafluoroisopropyl alcohol (HFIP) dramatically improved the yield, affording **8a** in 94% yield (entry 8). The radical-stabilization effect of HFIP has been well documented,^34–40^ and several radical-mediated transformations have been successfully carried out in HFIP.^41–43^ In our system, both the malonate radical and its intermediate adduct (*vide infra*) are likely stabilized by HFIP. When the reaction was conducted under an O_2_ atmosphere, the formation of the akuammiline skeleton was strongly inhibited (entry 9). As an alternative, alcohol **10** was obtained in 23% yield with several unidentified compounds. This result supports the generation of a malonate radical, which is likely trapped by O_2_ to form **10**. The cyclization also required electricity, and in its absence the starting material was recovered (entry 10).

With the optimized electrochemical conditions in hand, we next explored the substrate scope with respect to nitrogen protecting groups (Scheme 2). We found that common *N*-protecting groups were compatible with iodide-mediated N–C bond formation. For instance, replacement of the Ts group with either Boc or Cbz protection did not affect the cyclization efficiency, affording the corresponding products **4b** and **4c** in 86% and 80% yields, respectively. Similar to iodide-mediated N–C cyclization, both Boc and Cbz groups were well tolerated in place of Ts. Notably, Boc-protected substrate **3b** delivered the akuammiline-type product **8b** in 62% yield. Since anodic oxidation generates acidic species, which is called electro-generated acid,^44^ we hypothesized that acidic Boc deprotection could diminish the yield. Indeed, the addition of 0.2 mmol of *t*-BuOLi enhanced the yield of **8b** to 84%. The Cbz-protected analogue was also compatible with this reaction and gave **8c** in a 77% yield. In contrast, the use of a Ns group significantly hindered the cyclization. The iodide-mediated electrolysis of Ns precursor **3d** led to a complex mixture and furnished the desired product **4d** in only 9% yield with unidentified impurities. In addition, electrolysis with a Cp_2_Fe mediator also resulted in a highly complex reaction mixture, and only 1% of **8d** could be isolated.

**Scheme 2:**
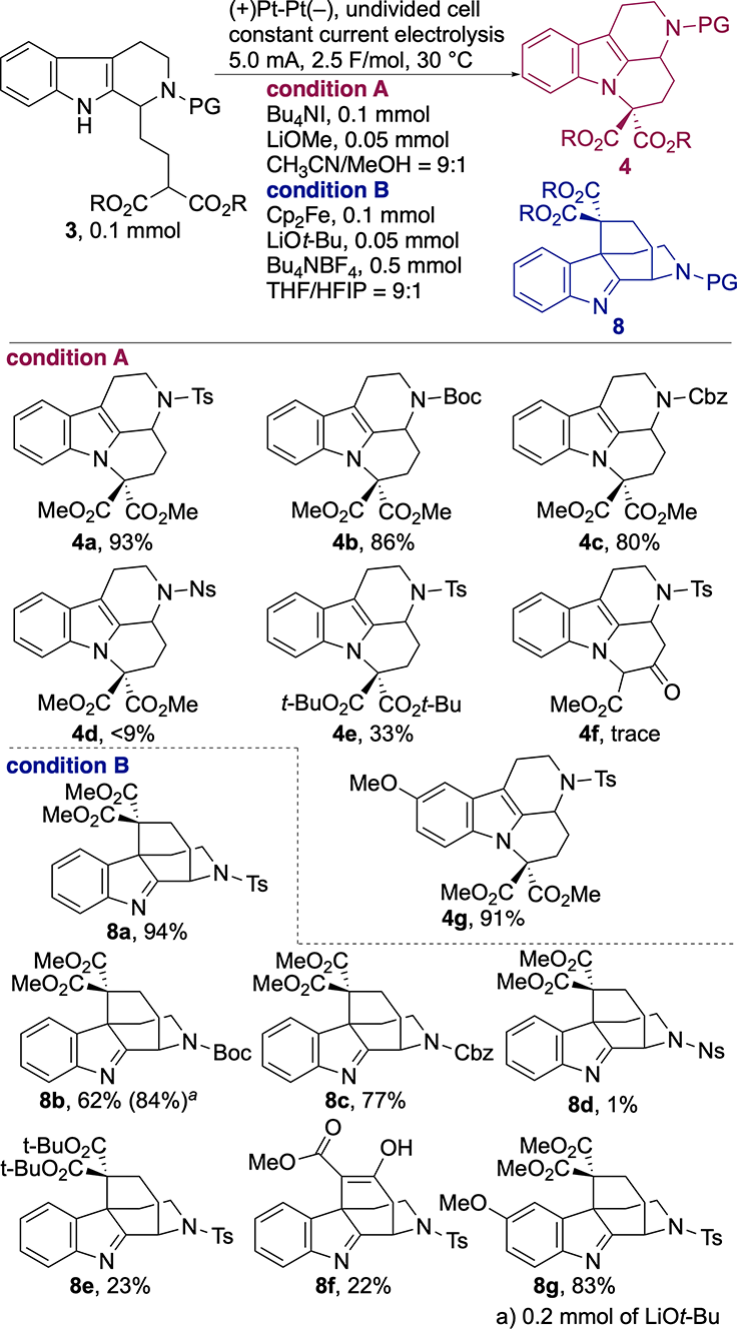
Substrate Scope

The reaction also exhibited a sensitivity to steric hindrance. Substrate **3e**, bearing a di-*tert*-butyl malonate moiety, underwent N–C bond formation to give **4e** in 33% yield. The Cp_2_Fe-mediated C–C bond-forming reaction was also sensitive to steric hindrance. The anodic oxidation of **3e** significantly decreases the efficiency, affording the desired akuammiline-type product **8e** in only a 23% yield.

To further expand the synthetic utility of this methodology, we examined β-ketoester substrate **3f** as an alternative to the malonate-type nucleophile. Under the iodide-mediated conditions, **3f** failed to undergo oxidation, and the starting material was recovered in 36% yield, suggesting a limitation in oxidizability or nucleophilicity of this substrate class. The Cp_2_Fe-mediated electrolysis provided the corresponding enol-type ketoester **8f** in 22% yield.

The electronic property of the indole ring did not significantly affect the cyclization. The presence of a methoxy substituent on the indole did not diminish the yield of the mavacurane-type skeleton, affording **4g** in 91% yield. Furthermore, the Cp_2_Fe-mediated cyclization of the same precursor **3g** furnished the akuammiline-type compound **8g** in 83% yield.

To elucidate the mechanism underlying the anodic cyclization, we conducted cyclic voltammetry (CV) experiments.^45^ The comparison of the voltammograms of substrate **3a**, products **4a** and **8a**, Bu_4_NI, and Cp_2_Fe revealed that either iodide or Cp_2_Fe is preferentially oxidized under the reaction conditions (Figures S8, S15). To determine which moiety of **3a**—the malonate or the indole unit—is more readily oxidized by the anodically generated I^+^ or ferrocenium species, we performed control CV experiments with model compounds. When dimethyl malonate was added to a Bu_4_NI or Cp_2_Fe solution under basic conditions, significant increases in anodic current attributed to I^–^ and Cp_2_Fe were observed (Figures S9, S19). These results indicate that the malonate anion, generated under basic conditions, undergoes indirect oxidation mediated by I^+^ or ferrocenium ions. In contrast, when *N*-Ts-1,2,3,4-tetrahydro-β-carboline—a model compound for the indole moiety—was added to a Bu_4_NI or Cp_2_Fe solution, no appreciable catalytic current was observed (Figures S11, S18). This finding suggests that the indole unit is not directly oxidized by I^+^ or ferrocenium species under these conditions.

Based on the insights gained from cyclic voltammetry and control experiments described in Supporting Information, we propose a reaction mechanism that underlies the formation of the mavacurane-type product **4a** (Figure 1). The sequence begins with deprotonation of the malonate moiety in substrate **3a**, generating the anionic intermediate **A**. The anodic oxidation of iodide generates electrophilic iodine species (I^+^), which are known to react with nucleophiles in solution.^46^ This nucleophilic species undergoes electrophilic iodination, affording iodinated intermediate **9**. Subsequently, a second deprotonation step is facilitated by methoxide, and the indole nitrogen attacks the iodinate malonate, leading to N–C bond formation and construction of the mavacurane core to yield **4a**. This proposed stepwise mechanism is further supported by DFT calculations (see Supporting Information). Notably, the calculated activation energy for the key cyclization step (from intermediate **B** to product **4a**) is only 1.3 kcal/mol, indicating that the transformation is kinetically accessible.

**Figure 1:**
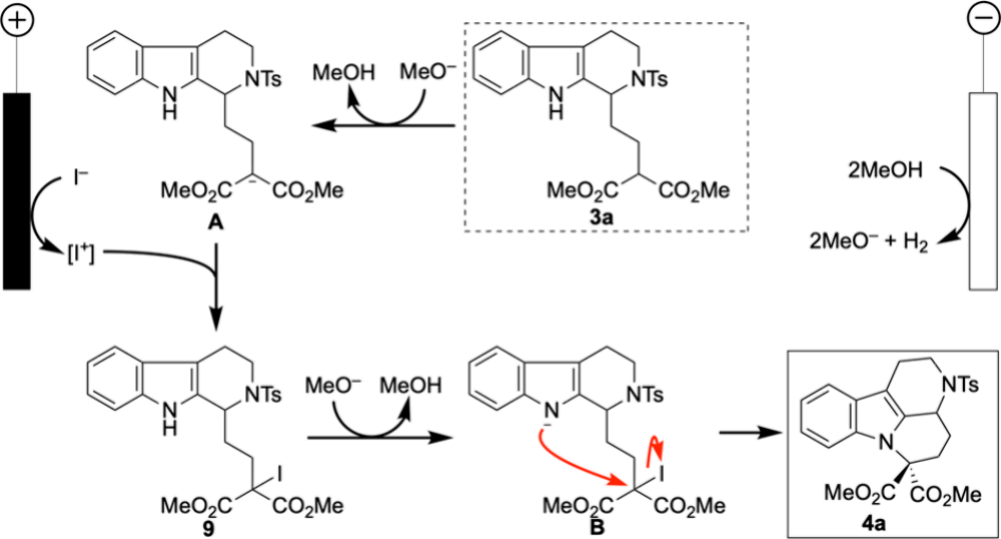
Plausible mechanism toward mavacurane skeleton.

To gain an understanding of the Cp_2_Fe-mediated C–C bond formation, we performed DFT calculations (Figure 2). After deprotonation of the malonate moiety, single-electron oxidation of intermediate **A** by the ferrocenium ion furnishes malonate radical **C**. The electrophilic nature of malonate radical **B** allows for cyclization.

**Figure 2:**
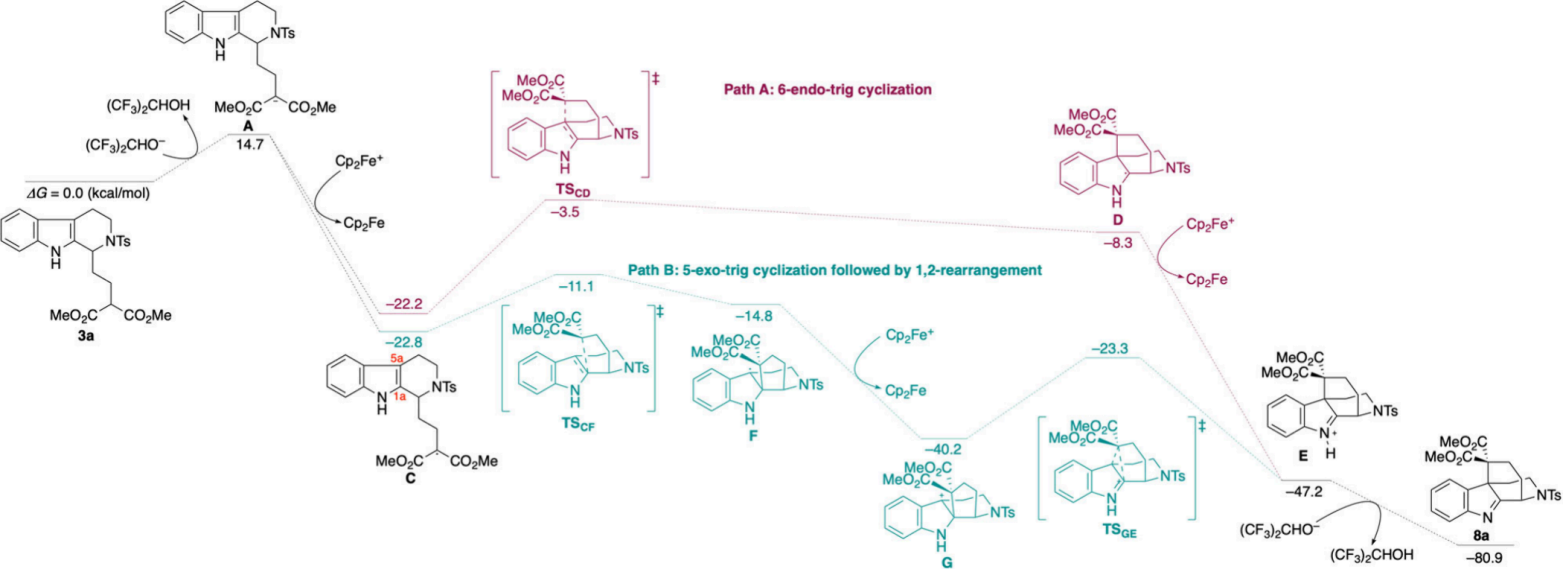
Energy profiles for the radical cyclization toward an akuammiline-type skeleton are calculated at (U)­B3LYP-D3/6-31+G­(d,p)_SMD(THF)_. Gibbs free energies (*ΔG*) are given in kcal/mol.

We first considered a 6-endo-trig cyclization pathway (Path A), in which the malonate radical adds to the C5a position, forming radical intermediate **D**. This species could then undergo oxidation to give iminium intermediate **E**, which upon deprotonation would yield the desired product **8a**. While this pathway is a simple explanation for construction of the akuammiline skeleton, DFT calculations revealed a relatively high activation barrier for the **C** to **D** transformation (*ΔG* ^*‡*^ = 18.7 kcal/mol), suggesting that this route may not be kinetically favorable.

We therefore considered an alternative and more favorable pathway: a 5-exo-trig cyclization, followed by a 1,2-rearrangement (Path B). In this scenario, the malonate radical **C** undergoes 5-exo-trig cyclization to form the benzylic radical **F**, proceeding via a significantly lower activation barrier (*ΔG* ^*‡*^ = 11.7 kcal/mol). Oxidation of **F** affords benzylic cation **G**. Semipinacol rearrangement generates the same iminium species **E**, which is subsequently deprotonated to furnish compound **8a**.

Importantly, all calculated energy barriers associated with Path B were markedly lower than those for Path A. These findings strongly support the conclusion that the anodic cyclization route to the akuammiline-type skeleton most likely proceeds via the 5-exo-trig cyclization/1,2-rearrangement pathway.

In summary, we have developed an electrochemical cyclization strategy to access both mavacurane- and akuammiline-type skeletons. A common intermediate bearing an indole core and a malonate moiety could be selectively oxidized by using either iodide or Cp_2_Fe as a redox mediator. The choice of mediator dramatically influenced the cyclization pathway, enabling the selective formation of both frameworks in high yields.^47^

Overall, our findings highlight the potential of electrochemical control in guiding complex skeletal transformations and pave the way for the rational design of oxidative cyclization strategies for alkaloid synthesis. Further studies toward the total synthesis of natural products using this electrochemical methodology are currently underway in our laboratory.

## Supplementary Material



## Data Availability

The data underlying this study are available in the published article and its Supporting Information.

## Notes

Experimental details, spectroscopic data, cyclic voltammograms, details of DFT calculations, and copies of ^1^H and ^13^C NMR spectra (PDF)
