# Supplementary material for: Bioinspired Electrochemical Cyclization toward the Divergent Synthesis of Mavacurane- and Akuammiline-Type Alkaloids
Source: Org Lett. 2025 Oct 14;27(42):11818–23. doi: 10.1021/acs.orglett.5c03645 (PMC12560074; doi:10.1021/acs.orglett.5c03645)

Supporting Information

Bioinspired Electrochemical Cyclization Toward the Divergent Synthesis of Mavacurane- and Akuammiline-type Alkaloids

Eisuke Sato*, Tomohiro Nakahama, Yuika Nomura, Koichi Mitsudo, and Seiji Suga*

Department of Applied Chemistry, Graduate School of Environmental, Life, Natural Science and Technology, Okayama University, 3-1-1 Tushima-naka, Kita-ku, Okayama 700-8530, Japan

E-mail addresses

Eisuke Sato: e-sato@okayama-u.ac.jp

Seiji Suga: suga@cc.okayama-u.ac.jp

Contents

1. General remarks S2
2. Synthesis of cyclization precursor S3
3. Anodic oxidation (mavacurane-type alkaloid skeleton) S23
4. Anodic oxidation (akuammiline-type alkaloid skeleton) S32
5. Control experiments S45
6. Cyclic voltammetry S47
7. DFT calculations S57
8. Reference S81
9. NMR spectra S82

1. General remarks

Nuclear magnetic resonance (NMR) spectra were recorded on JEOL JNM-ECZ600R (^{1}H 600 MHz, ^{13}C 150 MHz) and JEOL JNM-ECS400 (^{1}H 400 MHz, ^{13}C 100 MHz) spectrometers. Chemical shifts for ^{1}H NMR are expressed in parts per million (ppm) relative to TMS (δ 0.00 ppm) or residual $CHCl_3$ in $CDCl_3$ (δ 7.26 ppm). Chemical shifts for ^{13}C NMR are expressed in ppm relative to $CDCl_3$ (δ 77.16 ppm). Infrared (IR) spectra were recorded on SHIMADZU IRAffinity-1 spectrophotometer and JASCO FT/IR-4X with ATR Pro 4X. Cyclic voltammetry (CV) was recorded on Electrochemical Analyzer ALS-660E (BAS). Analytic thin layer chromatography (TLC) was performed on Merck, pre-coated plate silica gel 60 F_{254} (0.25 mm thickness). Column chromatography was performed on KANTO CHEMICAL silica gel 60N (40–50 μm). Gel permeation chromatography (GPC) was carried out on Japan Analytical Industry LC-5060 Plus II equipped with JAIGEL-2HR Plus using chloroform as an eluent. High-resolution mass spectrometry (HRMS) was performed on Bruker micrOTOF II-SKA (ESI-TOF) and Bruker compact (ESI-TOF). Melting points were measured on SANSYO SMP-300. Unless otherwise noted, all reactions were performed under argon atmosphere. All electrolysis was performed using PMX350-0.2A purchased from KIKUSUI ELECTRONIC COEPORATION. When heating was required, an oil bath was used, whereas for electrolysis performed at 30 °C, the temperature was maintained using a water bath.

Materials

Unless otherwise noted, materials were purchased from commercial suppliers and used without further purification. Dichloromethane (CH_2Cl_2) was dried over MS4A. Acetonitrile (CH_3CN) was distilled from CaH_2 and stored over MS3A. Methanol (MeOH) was dried over MS3A. 1,1,1,3,3,3-Hexafluoroisopropyl alcohol (HFIP) was dried over MS4A.

Electrochemical Equipment

Unless otherwise noted, all electrochemical reactions were carried out in an undivided cell equipped with platinum plate (1 cm × 1 cm, 0.20 mm thickness) electrodes (Figure S1).

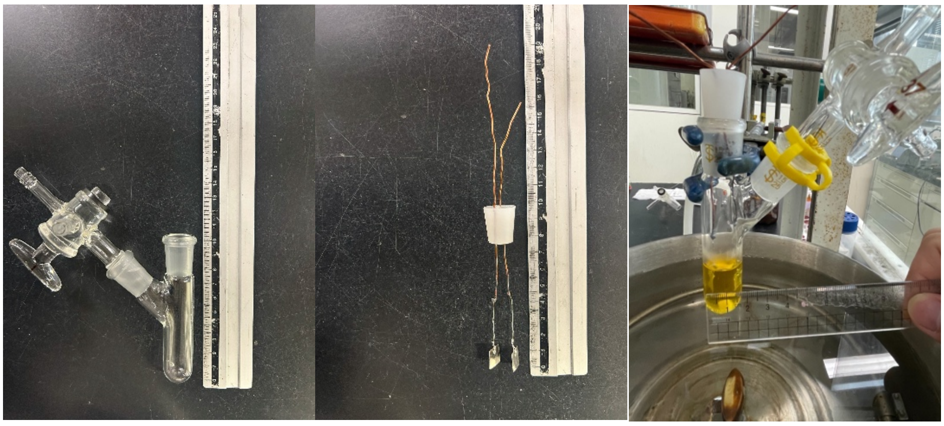

Figure S1. Electrochemical Equipment

2. Synthesis of cyclization precursor

Synthetic route of substrate 3a–3e

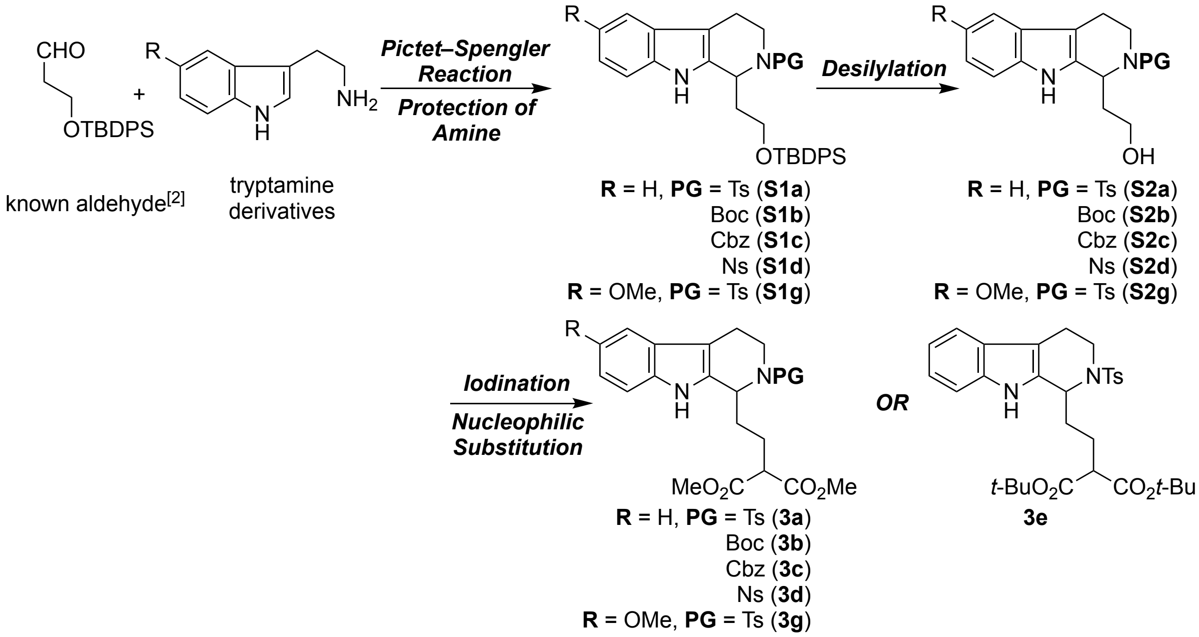

Synthetic route of 3f

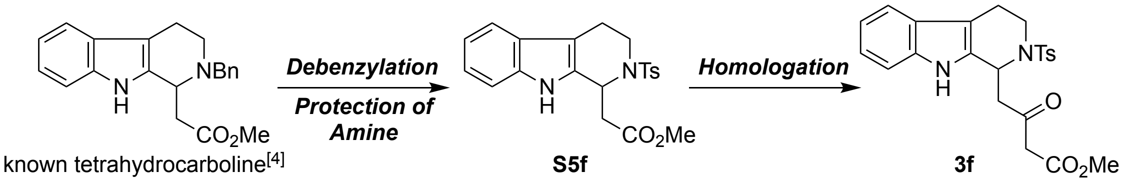

1-(2-((*tert*-Butyldiphenylsilyl)oxy)ethyl)- 2-tosyl-2,3,4,9-tetrahydro-1*H*-pyrido[3,4-*b*]indole (S1a).

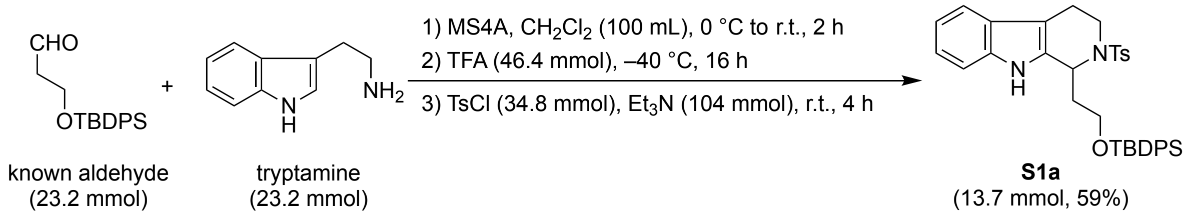

Compound **S1a** was prepared according to reported procedures.[1] To a solution of tryptamine (3.72 g, 23.2 mmol) and MS4A in CH_2Cl_2 (90 mL) was added a solution of known aldehyde[2] (7.26 g, 23.2 mmol) in CH_2Cl_2 (10 mL) dropwise at 0 °C. After stirring for 2 h at room temperature, trifluoroacetic acid (TFA, 3.60 mL, 46.4 mmol) was added at –40 °C. Then, the reaction mixture was stirred for 16 h at −40 °C, and triethylamine (15.0 mL, 104 mmol) and *p*-toluenesulfonyl chloride (6.64 g, 34.8 mmol) were added. After stirring for 4 h at room temperature, the resulting mixture was filtered through a Celite pad, diluted with H_2O (100 mL), and extracted with CH_2Cl_2 (3 × 100 mL). The combined organic layers were washed with brine, dried over $MgSO_4$ and concentrated *in vacuo*. The crude mixture was purified by column chromatography on silica gel (hexane/EtOAc = 10:1) to afford **S1a** as yellow liquid (8.34 g, 13.7 mmol, 59%).

^{1}H NMR (600 MHz, CDCl$_3$): δ 8.52 (brs, 1H, NH), 7.74–7.72 (m, 4H), 7.65 (d, *J* = 8.2 Hz, 2H), 7.51–7.48 (m, 2H), 7.45–7.43 (m, 4H), 7.34 (d, *J* = 8.2 Hz, 1H), 7.12–7.08 (m, 4H), 7.04 (dd, *J* = 1.4, 8.2 Hz, 1H), 5.38 (dd, *J* = 3.2, 5.0 Hz, 1H), 4.18 (ddd, *J* = 4.6, 13.7, 15.1 Hz, 1H), 4.01 (ddd, *J* = 5.0, 10.5, 15.1 Hz, 1H), 3.88 (ddd, *J* = 4.1, 8.2, 12.8 Hz, 1H), 3.32 (ddd, *J* = 5.0, 11.5, 12.8 Hz, 1H), 2.57–2.49 (m, 2H), 2.30 (s, 3H), 2.24 (m, 1H), 1.97 (m, 1H), 1.18 (s, 9H).

^{13}C NMR (150 MHz, CDCl$_3$): δ 143.3, 138.2, 135.8, 135.6, 133.4, 132.9, 132.7, 130.2, 129.7, 128.2, 126.9, 121.9, 119.4, 118.2, 111.0, 107.7, 61.9, 50.9, 40.1, 38.2, 27.2, 21.6, 20.5, 19.5.

IR (neat): 3400, 2950, 2400, 1150, 1100 cm^{-1}.

HRMS (ESI): Exact mass calculated for $C_{36}H_{40}N_2NaO_3SSi$ [M+Na]$^+$: 631.2421; found 631.2412.

***tert*-Butyl 1-(2-((*tert*-butyldiphenylsilyl)oxy)ethyl)-1,3,4,9-tetrahydro-2*H*-pyrido[3,4-*b*]indole-2-carboxylate (S1b)**

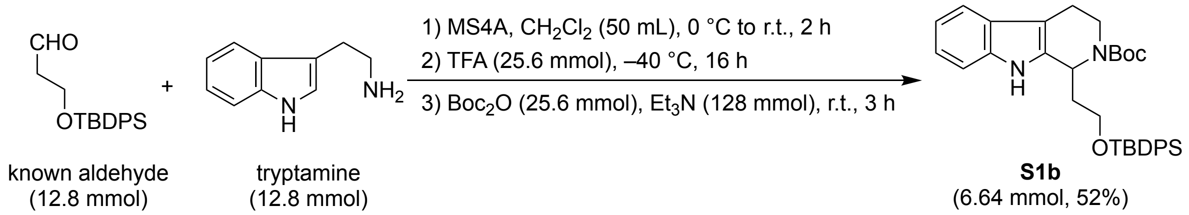

Compound **S1b** was prepared according to reported procedure.[1] To a solution of tryptamine (2.05 g, 12.8 mmol) and MS4A in CH_2Cl_2 (45 mL) was added a solution of known aldehyde[2] (4.00 g, 12.8 mmol) in CH_2Cl_2 (5 mL) dropwise at 0 °C. After stirring for 2 h at room temperature, TFA (2.00 mL, 25.6 mmol) was added at –40 °C. Then, the reaction mixture was stirred for 16 h at −40 °C, and triethylamine (17.8 mL, 128 mmol) and di-*tert*-butyl dicarbonate (Boc_2O, 5.59 g, 25.6 mmol) were added. After stirring for 3 h at room temperature, the resulting mixture was filtered through a Celite pad, diluted with H_2O (50 mL), and extracted with CH_2Cl_2 (3 × 100 mL). The combined organic layers were washed with brine, dried over $MgSO_4$ and concentrated *in vacuo*. The crude mixture was purified by column chromatography on silica gel (hexane/EtOAc = 10:1) to afford **S1b** (mixture of rotamers) as yellow liquid (3.69 g, 6.64 mmol, 52%).

^{1}H NMR (600 MHz, $CDCl_3$): δ 8.72–8.68 (m, 1H, NH), 7.73–7.71 (m, 9H), 7.49–7.48 (m, 2H), 7.12–7.08 (m, 3H), 5.49 (m, 0.4H), 5.39 (m, 0.6H), 4.49 (m, 0.6H), 4.28 (m, 0.4H), 4.04–3.92 (m, 1H), 3.08–3.01 (m, 1H), 2.83–2.82 (m, 1H), 2.72–2.70 (m, 1H), 2.22 (m, 1H), 2.18–2.11 (m, 1H), 2.05–1.98 (m, 1H), 1.45 (s, 9H), 1.18 (s, 4H), 1.07 (s, 5H).

^{13}C NMR (150 MHz, $CDCl_3$, Only major rotamer was assigned): δ 154.7, 135.7, 135.4, 134.9, 130.2, 129.7, 128.1, 127.8, 126.9, 121.7, 119.4, 118.3, 110.9, 62.6, 49.9, 38.1, 37.2, 28.6, 27.2, 26.7, 21.5, 19.4.

IR (neat): 3100, 2800, 1750, 1550, 950 cm^{-1}.

HRMS (ESI): Exact mass calculated for $C_{34}H_{42}N_2NaO_3Si$ $[M+Na]^+$: 577.2857; found 577.2874.

Benzyl 1-(2-((*tert*-butyldiphenylsilyl)oxy)ethyl)-1,3,4,9-tetrahydro-2*H*-pyrido[3,4-*b*]indole-2-carboxylate (S1c)

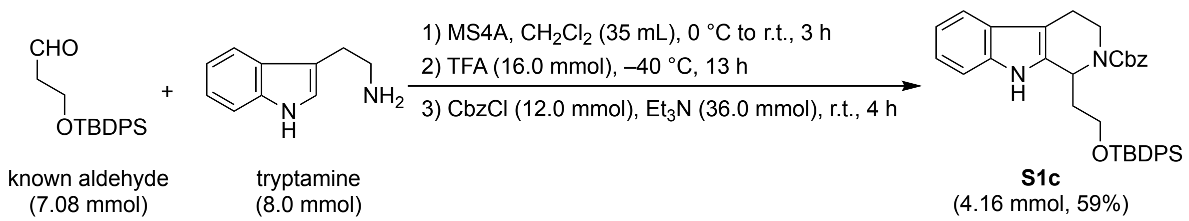

Compound **S1c** was prepared according to reported procedures.[1] To a solution of tryptamine (1.28 g, 8.0 mmol) and MS4A in CH_2Cl_2 (30 mL) was added a solution of known aldehyde[2] (2.21 g, 7.08 mmol) in CH_2Cl_2 (5 mL) dropwise at 0 °C. After stirring for 3 h at room temperature, TFA (1.2 mL, 16.0 mmol) was added at –40 °C. Then, the reaction mixture was stirred for 13 h at −40 °C, and triethylamine (5.0 mL, 36 mmol) and benzyl chloroformate (1.7 mL, 12.0 mmol) were added. After stirring for 3 h at room temperature, the resulting mixture was filtered through a Celite pad, diluted with H_2O (50 mL), and extracted with CH_2Cl_2 (3 × 100 mL). The combined organic layers were washed with brine, dried over $MgSO_4$ and concentrated *in vacuo*. The crude mixture was purified by column chromatography on silica gel (hexane/EtOAc = 10:1) to afford **S1c** (mixture of rotamers) as yellow liquid (2.45 g, 4.16 mmol, 57%).

^{1}H NMR (600 MHz, $CDCl_3$): δ 8.69 (brs, 0.4H, NH), 8.60 (brs, 0.6, NH), 7.74–7.64 (m, 5H), 7.49–7.45 (m, 4H), 7.43–7.28 (m, 6H), 7.14–7.07 (m, 4H), 5.58 (m, 0.4H), 5.52 (m, 0.6H), 5.20–5.13 (m, 2H), 4.55 (brdd, *J* = 5.0, 12.8 Hz, 0.6H), 4.41 (brdd, J = 5.0, 13.8 Hz, 0.4H), 4.45 (m, 0.4H), 3.94–3.84 (m 1.6H), 3.18–3.11 (m, 1H), 2.90–2.81 (m, 1H), 2.77–2.69 (m, 1H), 2.15–2.09 (m, 1H), 2.00 (m, 0.4H), 1.94 (m, 0.6H), 1.17 (s, 3.6H), 1.11 (s, 5.4H).

^{13}C NMR (150 MHz, $CDCl_3$, Only major rotamer was assigned): δ 155.4, 135.9, 135.8, 135.7, 135.6, 133.3, 132.9, 130.3, 130.2, 130.2, 128.7, 128.2, 128.1, 128.0, 121.9, 119.5, 119.4, 118.3, 111.0, 110.9, 108.7, 67.6, 62.2, 49.7, 38.9, 37.1, 27.1, 21.4, 19.4.

IR (neat): 2500, 2300, 1825, 1250, 800 cm^{-1}.

HRMS (ESI): Exact mass calculated for $C_{37}H_{40}N_2NaO_3Si$ $[M+Na]^+$: 611.2700; found 611.2699.

1-(2-((*tert*-Butyldiphenylsilyl)oxy)ethyl)-2-((2-nitrophenyl)sulfonyl)-2,3,4,9-tetrahydro-1*H*-pyrido[3,4-*b*]indole (S1d)

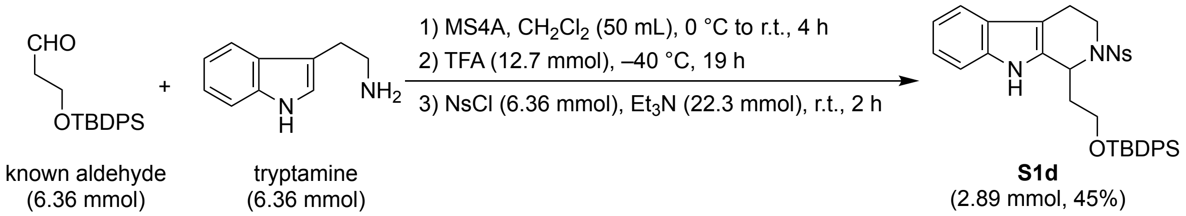

Compound **S1d** was prepared according to reported procedures.[1] To a solution of tryptamine (1.02 g, 6.36 mmol) and MS4A in CH_2Cl_2 (45 mL) was added a solution of known aldehyde[2] (2.03 g, 6.36 mmol) in CH_2Cl_2 (5 mL) dropwise at 0 °C. After stirring for 4 h at room temperature, TFA (1.00 mL, 12.7 mmol) was added at –40 °C. Then, the reaction mixture was stirred for 19 h at −40 °C, and triethylamine (3.1 mL, 22.3 mmol) and 2-nitrobenzenesulfonyl chloride (1.41 g, 6.36 mmol) were added. After stirring for 2 h at room temperature, the resulting mixture was filtered through a Celite pad, diluted with H_2O (50 mL), and extracted with CH_2Cl_2 (3 × 50 mL). The combined organic layers were washed with brine, dried over $MgSO_4$ and concentrated *in vacuo*. The crude mixture was purified by column chromatography on silica gel (hexane/EtOAc = 10:1) to afford **S1d** (mixture of rotamers) as yellow liquid (1.85 g, 2.89 mmol, 45%).

^{1}H NMR (600 MHz, $CDCl_3$): δ 8.54 (brs, 1H, NH), 7.95 (m, 1H), 7.72 (dd, *J* = 7.8, 7.8 Hz, 4H), 7.58 (dd, *J* = 8.2, 8.2 Hz, 1H), 7.55–7.46 (m, 4H), 7.44–7.41 (m, 4H), 7.38 (d, *J* = 7.8 Hz, 1H), 7.14–7.07 (m, 2H), 7.04 (dd, *J* = 7.8, 7.8 Hz, 1H), 5.47 (dd, *J* = 5.0, 8.2 Hz, 1H), 4.26 (dd, *J* = 5.0, 13.7 Hz, 1H), 3.96 (ddd, *J* = 5.0, 5.0, 13.7 Hz, 1H), 3.85 (ddd, *J* = 4.1, 8.2, 11.0 Hz, 1H), 3.45 (ddd, *J* = 5.5, 11.0, 14.7 Hz, 1H), 2.74–2.65 (m, 2H), 2.23 (m, 1H), 2.01 (m, 1H), 1.17 (s, 9H).

^{13}C NMR (150 MHz, $CDCl_3$): δ 148.1, 135.8, 135.6, 133.5, 132.8, 132.7, 131.8, 130.3, 130.3, 128.2, 128.1, 126.7, 124.4, 122.1, 119.5, 118.2, 111.1, 107.5, 61.8, 51.6, 40.5, 38.2, 27.2, 21.0, 19.5.

IR (neat): 3400, 2900, 2400, 1550, 1150 cm^{-1}.

HRMS (ESI): Exact mass calculated for $C_{35}H_{37}N_3NaO_5SSi$ $[M+Na]^+$: 662.2115; found 662.2136.

1-(2-((*tert*-Butyldiphenylsilyl)oxy)ethyl)- 6-methoxy-2-tosyl-2,3,4,9-tetrahydro-1*H*-pyrido[3,4-*b*]indole (S1g).

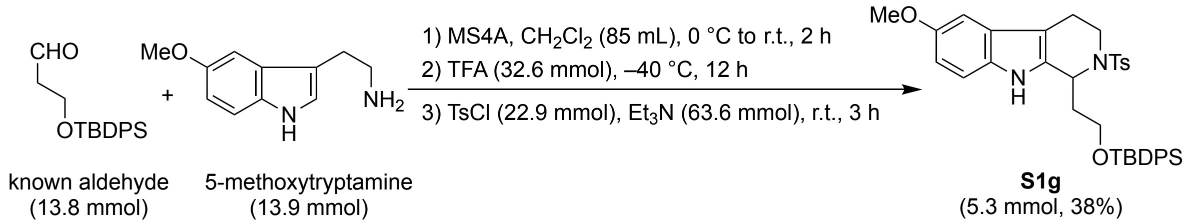

Compound **S1g** was prepared according to reported procedures.[1] To a solution of 5-methoxytryptamine (2.65 g, 13.9 mmol) and MS4A in CH_2Cl_2 (70 mL) was added a solution of known aldehyde[2] (4.31 g, 13.8 mmol) in CH_2Cl_2 (15 mL) dropwise at 0 °C. After stirring for 2 h at room temperature, trifluoroacetic acid (TFA, 2.50 mL, 32.6 mmol) was added at –40 °C. Then, the reaction mixture was stirred for 12 h at −40 °C, and triethylamine (9.0 mL, 63.6 mmol) and *p*-toluenesulfonyl chloride (4.37 g, 22.9 mmol) were added. After stirring for 3 h at room temperature, the resulting mixture was filtered through a Celite pad, diluted with H_2O (100 mL), and extracted with CH_2Cl_2 (3 × 100 mL). The combined organic layers were washed with brine, dried over $MgSO_4$ and concentrated *in vacuo*. The crude mixture was purified by column chromatography on silica gel (hexane/EtOAc = 7:1 to 4:1) to afford **S1g** as yellow liquid (3.34 g, 5.3 mmol, 38%).

^{1}H NMR (600 MHz, $CDCl_3$): δ 8.36 (s, 1H, NH), 7.73–7.71 (m, 4H), 7.66 (d, *J* = 8.3 Hz, 2H), 7.51–7.48 (m, 2H), 7.45–7.42 (m, 4H), 7.12 (d, *J* = 8.3 Hz, 2H), 6.97 (d, *J* = 8.9 Hz, 1H), 6.79–6.76 (m, 2H), 5.35 (dd, *J* = 4.8, 8.3 Hz, 1H), 4.17 (ddd, *J* = 1.4, 4.1, 13.8 Hz, 1H), 3.99 (m, 1H), 3.86 (m, 1H), 3.81 (s, 3H), 3.32 (m, 1H), 2.51–2.49 (m, 2H), 2.31 (s, 3H), 2.22 (m, 1H), 1.96 (ddd, *J* = 4.8, 9.6, 18.6 Hz, 1H), 1.17 (s, 9H).

^{13}C NMR (150 MHz, $CDCl_3$): δ 154.0, 143.2, 138.2, 135.7, 135.6, 133.5, 133.4, 132.9, 130.8, 130.2, 129.6 (2C), 128.14, 128.08,127.1, 126.9, 111.7, 111.6, 107.4, 100.4, 61.8, 56.0, 50.9, 40.0, 38.2, 27.1, 21.5, 20.5, 19.4.

IR (neat): 3400, 2900, 2850, 1450, 1150 cm^{-1}.

HRMS (ESI): Exact mass calculated for $C_{37}H_{42}N_2NaO_4SSi$ $[M+Na]^+$: 661.2527; found 661.2524.

2-(2-Tosyl-2,3,4,9-tetrahydro-1*H*-pyrido[3,4-*b*]indol-1-yl)ethan-1-ol (S2a)

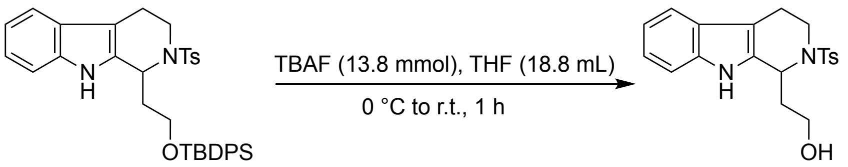

S1a (6.9 mmol) **S2a** (6.61 mmol, 96%)

To a solution of **S1a** (4.20 g, 6.89 mmol) in THF (5.0 mL) was added a solution of 1 M tetrabutylammonium fluoride (TBAF) in THF (13.8 mL, 13.8 mmol) dropwise at 0 °C, and stirred for 1 h at room temperature. The resulting mixture was diluted with saturated aqueous NH_4Cl (50 mL) and extracted with EtOAc (3 × 50 mL). The combined organic layers were washed with brine, dried over $MgSO_4$ and concentrated *in vacuo*. The crude mixture was purified by column chromatography on silica gel (hexane/EtOAc = 4:1 to 0:1) to afford **S2a** (2.45 g, 6.61 mmol, 96%) as yellow oil.

^{1}H NMR (600 MHz, $CDCl_3$): δ 8.57 (brs, 1H, NH), 7.65 (d, *J* = 10.5 Hz, 2H), 7.31–7.29 (m, 2H), 7.14 (dd, *J* = 6.9, 6.9 Hz, 1H), 7.09 (d, *J* = 10.5 Hz, 2H), 7.04 (dd, *J* = 6.9, 7.8 Hz, 1H), 5.40 (dd, *J* = 3.2, 10.1 Hz, 1H), 4.22 (dd, *J* = 5.5, 14.7 Hz, 1H), 4.13 (ddd, *J* = 2.3, 11.9, 11.9 Hz, 1H), 3.83 (ddd, *J* = 3.7, 7.3, 11.9 Hz, 1H), 3.37 (m, 1H), 3.28 (brs, 1H, OH), 2.51 (dd, *J* = 3.7, 15.6 Hz, 1H), 2.33 (m, 1H), 2.27 (s, 3H), 2.17 (m, 1H), 1.99 (m, 1H).

^{13}C NMR (150 MHz, $CDCl_3$): δ 143.6, 137.6, 136.1, 132.7, 129.7, 126.6, 126.5, 121.8, 119.1, 117.9, 111.3, 107.1, 58.5, 50.2, 39.7, 37.8, 21.4, 19.6.

IR (neat): 3400, 2950, 2370, 1300, 1150 cm^{-1}.

HRMS (ESI): Exact mass calculated for $C_{20}H_{22}N_2NaO_3S$ $[M+Na]^+$: 393.1243; found 393.1214.

***tert*-Butyl 1-(2-hydroxyethyl)-1,3,4,9-tetrahydro-2*H*-pyrido[3,4-*b*]indole-2-carboxylate (S2b)**

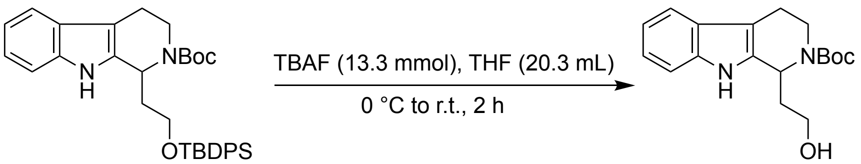

S1b (6.64 mmol) **S2b** (3.2 mmol, 48%)

To a solution of **S1b** (3.69 g, 6.64 mmol) in THF (7.0 mL) was added a solution of 1 M TBAF in THF (13.3 mL, 13.3 mmol) dropwise at 0 °C, and stirred for 2 h at room temperature. The resulting mixture was diluted with saturated aqueous NH_4Cl (50 mL) and extracted with EtOAc (3 × 50 mL). The combined organic layers were washed with brine, dried over $MgSO_4$ and concentrated *in vacuo*. The crude mixture was purified by column chromatography on silica gel (hexane/EtOAc = 4:1 to 0:1) to afford **S2b** (mixture of rotamers, 1.01 g, 3.20 mmol, 48%) as yellow oil.

^{1}H NMR (600 MHz, $CDCl_3$): δ 8.47–8.41 (m, 1H, NH), 7.47 (m, 1H), 7.34 (m, 1H), 7.17–7.08 (m, 2H), 5.46 (m, 0.8H), 5.31 (m, 0.2H), 4.50 (m, 0.2H), 4.33 (m, 0.8H), 3.90–3.85 (m, 1H), 3.75–3.66 (m, 2H), 3.11–3.07 (m, 1H), 2.86–2.81 (m, 1H), 2.73–2.70 (m, 1H, OH), 2.05–2.01 (m, 2H), 1.51 (s, 9H).

^{13}C NMR (150 MHz, $CDCl_3$, only major rotamer was assigned): δ 156.6, 136.2, 134.2, 126.8, 121.9, 119.6, 118.1, 111.2, 108.4, 81.0, 58.5, 47.7, 39.1, 36.7, 28.6, 21.8.

IR (neat): 3100, 2400, 1750, 1500, 1200 cm^{-1}.

HRMS (ESI): Exact mass calculated for $C_{18}H_{24}N_2NaO_3$ $[M+Na]^+$: 339.1679; found 339.1682.

Benzyl 1-(2-hydroxyethyl)-1,3,4,9-tetrahydro-2*H*-pyrido[3,4-*b*]indole-2-carboxylate (S2c)

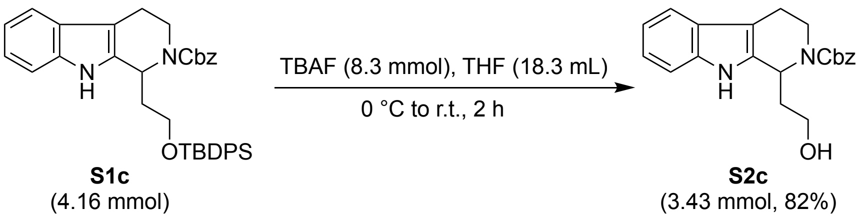

To a solution of **S1c** (3.69 g, 6.64 mmol) in THF (7.0 mL) was added a solution of 1 M TBAF in THF (8.3 mL, 8.3 mmol) dropwise at 0 °C, and stirred for 2 h at room temperature. The resulting mixture was diluted with saturated aqueous NH_4Cl (50 mL) and extracted with EtOAc (3 × 50 mL). The combined organic layers were washed with brine, dried over $MgSO_4$ and concentrated *in vacuo*. The crude mixture was purified by column chromatography on silica gel (hexane/EtOAc = 4:1 to 0:1) to afford **S2c** (mixture of rotamers, 1.20 g, 3.43 mmol, 82%) as yellow oil.

^{1}H NMR (600 MHz, $CDCl_3$): δ 8.44–8.39 (m, 1H, NH), 7.50–7.46 (m, 1H), 7.40–7.31 (m, 6H), 7.20–7.16 (m, 1H), 7.11–7.09 (m, 1H), 5.52 (dd, *J* = 3.2, 11.0 Hz, 0.8H), 5.40 (m, 0.2H), 5.27–5.21 (m, 1.8H), 5.11 (m, 0.2H), 5.56 (brdd, *J* = 4.1, 11.9 Hz, 0.2H), 4.45 (brdd, *J* = 5.0, 13.8 Hz, 0.8H), 3.86 (m, 0.4H), 3.78–3.68 (m, 1.6H), 3.18–3.14 (m, 1H), 2.86–2.83 (m, 1H), 2.76–2.73 (m, 1H), 2.18 (m, 0.8H), 2.03 (m, 0.2H), 1.91 (m, 0.8H), 1.85 (m, 0.2H), 1.61–1.60 (m, 1H, OH).

^{13}C NMR (150 MHz, $CDCl_3$, only major rotamer was assigned): δ 157.1, 136.5, 136.2, 133.8, 128.8, 128.4, 128.0, 126.7, 121.9, 119.5, 118.1, 111.3, 108.1, 67.9, 58.5, 48.6, 39.0, 36.7, 21.8.

IR (neat): 2950, 2500, 2280, 1900, 1750 cm^{-1}.

HRMS (ESI): Exact mass calculated for $C_{21}H_{22}N_2NaO_3$ $[M+Na]^+$: 373.1523; found 373.1520.

2-(2-((2-Nitrophenyl)sulfonyl)-2,3,4,9-tetrahydro-1*H*-pyrido[3,4-*b*]indol-1-yl)ethan-1-ol (S2d)

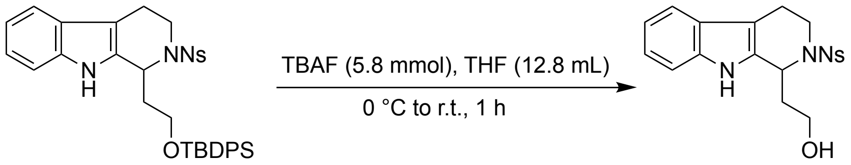

S1d
(2.89 mmol)

S2d
(2.33 mmol, 81%)

To a solution of **S1d** (1.85 g, 2.89 mmol) in THF (7.0 mL) was added a solution of 1 M TBAF in THF (5.8 mL, 5.8 mmol) dropwise at 0 °C, and stirred for 1 h at room temperature. The resulting mixture was diluted with saturated aqueous NH_4Cl (50 mL) and extracted with EtOAc (3 × 50 mL). The combined organic layers were washed with brine, dried over $MgSO_4$ and concentrated *in vacuo*. The crude mixture was purified by column chromatography on silica gel (hexane/EtOAc = 4:1 to 0:1) to afford **S2d** (936.0 mg, 2.33 mmol, 81%) as yellow oil.

^{1}H NMR (600 MHz, $CDCl_3$): δ 8.83 (brs, 1H, NH), 7.98 (dd, *J* = 0.9, 7.8 Hz, 1H), 7.59–7.49 (m, 3H), 7.36–7.32 (m, 2H), 7.15 (dd, *J* = 7.3, 7.3 Hz, 1H), 7.05 (dd, *J* = 7.3, 7.3 Hz, 1H), 5.50 (dd, *J* = 4.6, 9.2 Hz, 1H), 4.31 (dd, *J* = 5.5, 14.7 Hz, 1H), 4.04 (m, 1H), 3.83 (m, 1H), 3.52 (ddd, *J* = 4.6, 12.4, 15.1 Hz, 1H), 2.92 (brs, 1H, OH), 2.69–2.55 (m, 2H), 2.19 (m, 1H), 2.09 (m, 1H).

^{13}C NMR (150 MHz, $CDCl_3$): δ 148.1, 136.1, 134.0, 133.8, 132.9, 132.0, 130.1, 126.5, 124.3, 122.2, 119.5, 118.1, 111.4, 107.1, 58.8, 51.0, 40.2, 37.9, 20.4.

IR (neat): 3400, 2400, 1550, 1150, 750 cm^{-1}.

HRMS (ESI): Exact mass calculated for $C_{19}H_{19}N_3NaO_5S$ $[M+Na]^+$: 424.0938; found 424.0921.

2-(6-Methoxy-2-tosyl-2,3,4,9-tetrahydro-1*H*-pyrido[3,4-*b*]indol-1-yl)ethan-1-ol (S2g)

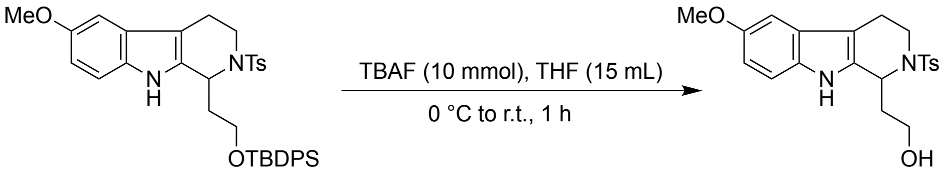

S1g
(4.8 mmol)

S2g
(4.79 mmol, quant)

To a solution of **S1g** (3.07 g, 4.8 mmol) in THF (5.0 mL) was added a solution of 1 M tetrabutylammonium fluoride (TBAF) in THF (10 mL, 10 mmol) dropwise at 0 °C, and stirred for 1.5 h at room temperature. The resulting mixture was diluted with saturated aqueous NH_4Cl (50 mL) and extracted with EtOAc (3 × 50 mL). The combined organic layers were washed with brine, dried over $MgSO_4$ and concentrated *in vacuo*. The crude mixture was purified by column chromatography on silica gel (hexane/EtOAc = 2:1 to 0:1) to afford **S2g** (1.92 g, 4.79 mmol, quant) as yellow solid.

^{1}H NMR (600 MHz, CDCl$_3$): δ 8.12 (brs, 1H, NH), 7.64 (d, *J* = 8.3 Hz, 2H), 7.18 (d, *J* = 8.3 Hz, 1H), 7.11 (d, *J* = 8.3 Hz, 2H), 6.79 (dd, *J* = 2.8, 8.3 Hz, 1H), 6.75 (d, *J* = 2.8 Hz, 1H), 5.33 (dd, *J* = 4.8, 10.3 Hz, 1H), 4.20 (brdd, *J* = 5.5, 15.2 Hz, 1H), 4.09 (ddd, *J* = 2.8, 10.3, 15.2 Hz, 1H), 3.79 (s, 3H), 3.36 (ddd, *J* = 4.1, 12.4, 14.5 Hz, 1H), 2.98 (brs, 1H, OH), 2.45 (brdd, *J* = 2.8, 15.2 Hz, 1H), 2.31 (m, 1H), 2.29 (s, 3H), 2.10 (m, 1H), 1.98 (m, 1H).

^{13}C NMR (150 MHz, CDCl$_3$): δ 154.1, 143.7, 137.8, 133.5, 131.1, 129.8, 127.0, 126.7, 111.82, 111.76, 107.4, 100.5, 58.5, 56.0, 50.2, 39.8, 38.0, 21.6, 19.8.

IR (neat): 3380, 2930, 2360, 1480, 1150 cm^{-1}.

HRMS (ESI): Exact mass calculated for $C_{21}H_{24}N_2NaO_4S$ $[M+Na]^+$: 423.1349; found 423.1344.

Melting Point: 66.3–72.2 °C

Dimethyl 2-(2-(2-tosyl-2,3,4,9-tetrahydro-1*H*-pyrido[3,4-*b*]indol-1-yl)ethyl)malonate (3a)[3]

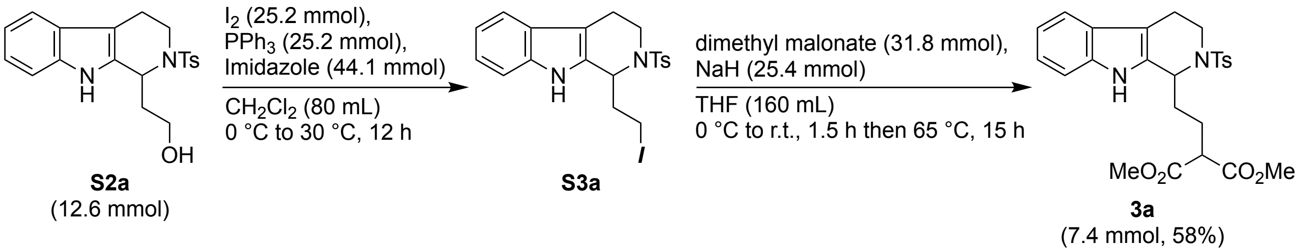

To a solution of **S2a** (4.67 g, 12.6 mmol) in CH_2Cl_2 (80 mL) was added imidazole (3.00 g, 44.1 mmol), triphenylphosphine (6.61 g, 25.2 mmol), and iodine (6.36 g, 25.2 mmol) at 0 °C. After stirring for 12 h at 30 °C in dark, the resulting mixture was diluted with saturated aqueous sodium thiosulfate (100 mL) and extracted with CH_2Cl_2 (3 × 100 mL). The combined organic layers were washed with brine, dried over $MgSO_4$ and concentrated *in vacuo*. The crude mixture was passed through a short pad column on silica gel (hexane/EtOAc = 5:1) and used for the next step.

To a solution of sodium hydride (1.02 g, 25.4 mmol, 60% dispersion in mineral oil) in THF (110 mL) was added dimethyl malonate (3.60 mL, 31.8 mmol) dropwise at 0 °C. After stirring for 1.5 h at room temperature, a solution of **S3a** (6.12 g, ca. 12.7 mmol) in THF (50 mL) was added at 0 °C. Then, the reaction mixture was stirred for 15 h at 65 °C. The resulting mixture was diluted with saturated aqueous NH_4Cl (100 mL) and extracted with EtOAc (3 × 100 mL). The combined organic layers were washed with brine, dried over $MgSO_4$ and concentrated *in vacuo*. The crude mixture was purified by column chromatography on silica gel (hexane/EtOAc = 10:1) to afford **3a**[3] (4.59 g, 7.40 mmol, 58%) as yellow oil.

^{1}H NMR (600 MHz, $CDCl_3$): δ. 8.06 (brs, 1H, NH), 7.61 (d, *J* = 8.7 Hz, 2H), 7.32–7.28 (m, 2H), 7.14 (dd, *J* = 6.9, 6.9 Hz, 1H), 7.09 (d, *J* = 8.7 Hz, 2H), 7.04 (dd, *J* = 6.9, 6.9 Hz, 1H), 5.13 (dd, *J* = 5.5, 7.8 Hz, 1H), 4.14 (dd, *J* = 5.9, 15.1 Hz, 1H), 3.78 (s, 3H), 3.75 (s, 3H), 3.56 (dd, *J* = 7.3, 7.3 Hz, 1H), 3.38 (m, 1H), 2.46(ddd, *J* = 3.7, 15.6, 17.4 Hz, 1H), 2.32 (m, 1H), 2.27 (s, 3H), 2.21–2.17 (m, 2H), 1.95–1.89 (m, 2H).

^{13}C NMR (150 MHz, $CDCl_3$): δ 170.1, 169.8, 143.5, 138.0, 136.0, 132.5, 129.7, 126.9, 126.7, 122.2, 119.6, 118.2, 111.1, 108.0, 52.9, 52.8, 52.5, 50.9, 39.8, 33.3, 25.3, 21.5, 19.7.

IR (neat): 3400, 2950, 2350, 1750, 1350 cm^{-1}.

Dimethyl 2-(2-(2-(*tert*-butoxycarbonyl)-2,3,4,9-tetrahydro-1*H*-pyrido[3,4-*b*]indol-1-yl)ethyl)malonate (3b)

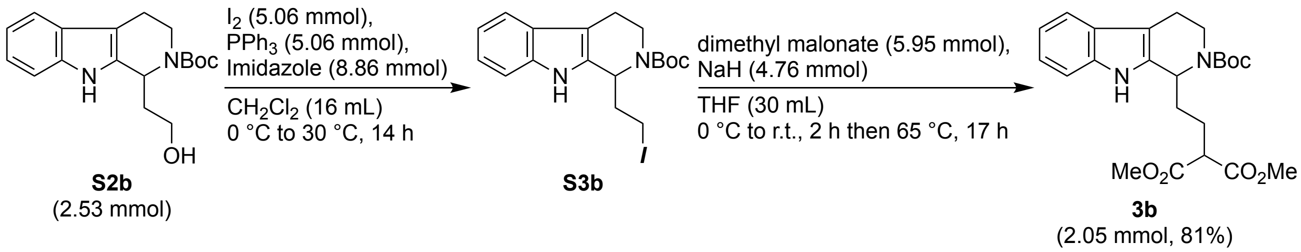

To a solution of **S2b** (0.80 g, 2.53 mmol) in CH_2Cl_2 (16 mL) was added imidazole (0.603 g, 8.86 mmol), triphenylphosphine (1.33 g, 5.06 mmol), and iodine (1.28 g, 5.06 mmol) at 0 °C. After stirring for 14 h at 30 °C in dark, the resulting mixture was diluted with saturated aqueous sodium thiosulfate (20 mL) and extracted with CH_2Cl_2 (3 × 20 mL). The combined organic layers were washed with brine, dried over $MgSO_4$ and concentrated *in vacuo*. The crude mixture was passed through a short pad column on silica gel (hexane/EtOAc = 5:1) and used for the next step.

To a solution of sodium hydride (0.19 g, 4.76 mmol, 60% dispersion in mineral oil) in THF (20 mL) was added dimethyl malonate (0.68 mL, 5.95 mmol) dropwise at 0 °C. After stirring for 2 h at room temperature, a solution of **S3b** (1.02 g, ca. 2.48 mmol) in THF (10 mL) was added at 0 °C. Then, the reaction mixture was stirred for 17 h at 65 °C. The resulting mixture was diluted with saturated aqueous NH_4Cl (30 mL) and extracted with EtOAc (3 × 30 mL). The combined organic layers were washed with brine, dried over $MgSO_4$ and concentrated *in vacuo*. The crude mixture was purified by column chromatography on silica gel (hexane/EtOAc = 5:1) to afford **3b** (mixture of rotamers, 880 mg, 2.05 mmol, 81%) as yellow oil.

^{1}H NMR (600 MHz, $CDCl_3$): δ 7.95 (brs, 1H, NH), 7.46–7.43 (m, 1H), 7.33–7.32 (m, 1H), 7.17–7.08 (m, 2H), 5.33 (m, 0.5H), 5.16 (m, 0.5H), 4.50 (m, 0.5H), 4.30 (m, 0.5H), 3.77–3.75 (m, 6H), 3.64–3.58 (m, 0.5H), 3.49–3.47 (m, 0.5H), 3.13–3.08 (m, 1H), 2.82 (m, 1H), 2.70–2.67 (m, 1H), 2.17–2.10 (m, 2H), 1.87–1.81 (m, 2H), 1.48 (s, 9H).

^{13}C NMR (150 MHz, $CDCl_3$, only major rotamer was assigned): δ 169.9, 169.8 155.5, 136.1, 134.3, 126.8, 121.7, 119.3, 118.0, 111.1, 108.2, 80.1, 52.6, 51.1, 50.2, 38.8, 37.5, 32.0, 28.5, 25.4, 21.5.

IR (neat): 3200, 2800, 1800, 1600, 1150 cm^{-1}.

HRMS (ESI): Exact mass calculated for $C_{23}H_{30}N_2NaO_6$ $[M+Na]^+$: 453.1996; found 453.1986.

Dimethyl 2-(2-(2-((benzyloxy)carbonyl)-2,3,4,9-tetrahydro-1*H*-pyrido[3,4-*b*]indol-1-yl)ethyl)malonate (3c)

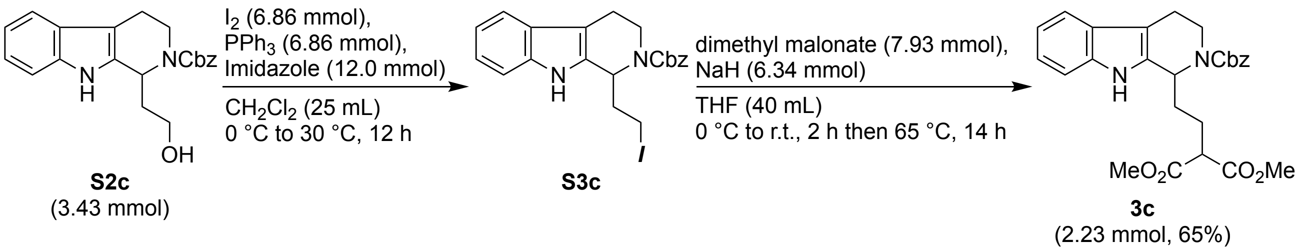

To a solution of **S2c** (1.20 g, 3.43 mmol) in CH_2Cl_2 (25 mL) was added imidazole (817.3 mg, 12.0 mmol), triphenylphosphine (1.80 g, 6.86 mmol), and iodine (1.74 g, 6.86 mmol) at 0 °C. After stirring for 14 h at 30 °C in dark, the resulting mixture was diluted with saturated aqueous sodium thiosulfate (25 mL) and extracted with CH_2Cl_2 (3 × 25 mL). The combined organic layers were washed with brine, dried over $MgSO_4$ and concentrated *in vacuo*. The crude mixture was passed through a short pad column on silica gel (hexane/EtOAc = 5:1) and used for the next step.

To a solution of sodium hydride (253.6 mg, 6.34 mmol, 60% dispersion in mineral oil) in THF (30 mL) was added dimethyl malonate (0.91 mL, 7.93 mmol) dropwise at 0 °C. After stirring for 2 h at room temperature, a solution of **S3c** (1.46 g, ca. 3.17 mmol) in THF (10 mL) was added at 0 °C. Then, the reaction mixture was stirred for 14 h at 65 °C. The resulting mixture was diluted with saturated aqueous NH_4Cl (40 mL) and extracted with EtOAc (3 × 40 mL). The combined organic layers were washed with brine, dried over $MgSO_4$ and concentrated *in vacuo*. The crude mixture was purified by column chromatography on silica gel (hexane/EtOAc = 5:1) to afford **3c** (mixture of rotamers, 1.04 g, 2.23 mmol, 65%) as yellow oil.

^{1}H NMR (600 MHz, $CDCl_3$): δ 8.01 (brs, 0.6H, NH), 7.95 (brs, 0.4H, NH), 7.46–7.44 (m, 1H), 7.37–7.32 (m, 6H), 7.18–7.15 (m, 1H), 7.11–7.08 (m, 1H), 5.38 (m, 0.6H), 5.23–5.11 (m, 2.4H), 4.56 (m, 0.4H), 4.42 (m, 0.6H), 3.76–3.72 (m, 6H), 3.56 (m, 0.6H), 3.39 (m, 0.4H), 3.23–3.15 (m, 1H), 2.91–2.80 (m, 1H), 2.74–2.69 (m, 1H), 2.12–2.04 (m, 2H), 1.91–1.88 (m, 2H).

^{13}C NMR (150 MHz, $CDCl_3$, only major rotamer was assigned): δ 170.1, 169.8, 156.1, 136.8, 136.1, 133.8, 128.7, 128.4, 127.9, 126.8, 122.1, 119.7, 118.2, 111.1, 108.6, 67.8, 67.5, 52.8, 51.2, 51.0, 38.8, 32.3, 25.4, 21.7.

IR (neat): 3100, 2810, 2600, 1780, 1600 cm^{-1}.

HRMS (ESI): Exact mass calculated for $C_{26}H_{28}N_2NaO_6$ $[M+Na]^+$: 487.1840; found 487.1839.

2-(2-((2-Nitrophenyl)sulfonyl)-2,3,4,9-tetrahydro-1*H*-pyrido[3,4-*b*]indol-1-yl)ethyl-4-methylbenzenesulfonate (S4d)

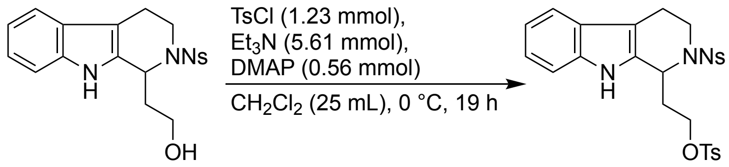

S2d
(1.12 mmol)

S4d
(0.94 mmol, 84%)

To a solution of **S2d** (451 mg, 1.12 mmol) in CH_2Cl_2 (25 mL) was added *p*-toluenesulfonyl chloride (236 mg, 1.23 mmol), triethylamine (0.78 mL, 5.61 mmol), and 4-dimethylaminopyridine (73 mg, 0.56 mmol) at 0 °C and stirred for 18 h at 0 °C. The resulting mixture was diluted with H_2O (30 mL) and extracted with CH_2Cl_2 (3 × 30 mL). The combined organic layers were washed with brine, dried over $MgSO_4$ and concentrated *in vacuo*. The crude mixture was purified by column chromatography on silica gel (hexane/EtOAc = 4:1) to afford **S4d** (520 mg, 0.94 mmol, 84%) as yellow oil.

^{1}H NMR (600 MHz, CDCl$_3$): δ 8.37 (brs, 1H, NH), 7.96 (dd, *J* = 1.4, 8.2 Hz, 1H), 7.86 (d, *J* = 8.2 Hz, 2H), 7.59 (m, 1H), 7.54 (dd, *J* = 1.4, 7.8 Hz, 1H), 7.52 (dd, *J* = 1.4, 7.8 Hz, 1H), 7.39–7.33 (m, 4H), 7.18 (ddd, *J* = 0.9, 6.9, 8.2 Hz, 1H), 7.07 (dd, *J* = 7.8, 7.8 Hz, 1H), 5.35 (dd, *J* = 6.4, 6.4 Hz, 1H), 4.38 (dd, *J* = 6.4, 11.0, 1H), 4.30–4.23 (m, 2H), 3.46 (m, 1H), 2.69–2.62 (m, 2H), 2.47 (s, 3H), 2.32–2.25 (m, 2H).

^{13}C NMR (150 MHz, CDCl$_3$): δ 148.1, 145.5, 136.2, 133.9, 133.8, 132.6, 132.0, 131.6, 130.5 130.2, 128.2, 126.5, 124.4, 122.6, 119.8, 118.3, 111.5, 108.0, 67.4, 50.4, 40.1, 35.6, 21.9, 20.6.

IR (neat): 3400, 2400, 1600, 1350, 1200 cm^{-1}.

HRMS (ESI): Exact mass calculated for $C_{26}H_{25}N_3NaO_7S_2$ $[M+Na]^+$: 578.1026; found 578.1033.

Dimethyl 2-(2-(2-((2-nitrophenyl)sulfonyl)-2,3,4,9-tetrahydro-1*H*-pyrido[3,4-*b*]indol-1-yl)ethyl)malonate (3d)

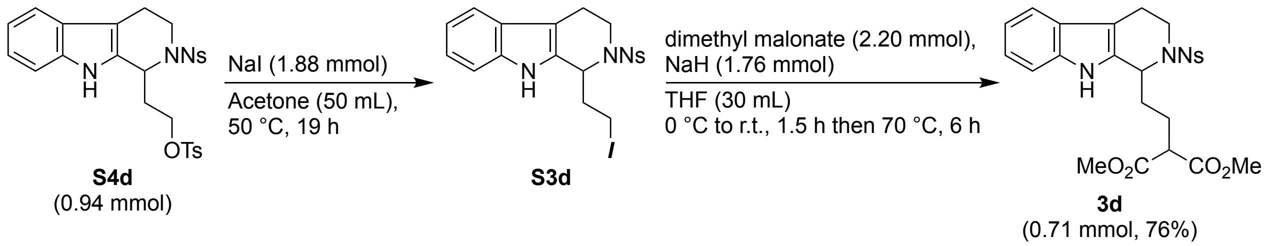

To a solution of **S4d** (524 mg, 0.94 mmol) in acetone (50 mL) was added sodium iodide (284 mg, 1.88 mmol) at room temperature. After stirring for 19 h at 50 °C, the resulting mixture was added H_2O (30 mL) and extracted with diethyl ether (3 × 30 mL). The combined organic layers were washed with brine, dried over $MgSO_4$ and concentrated *in vacuo* to afford **S3d** (465 mg, ca. 0.882 mmol). **S3d** was used for the next step without further purification.

To a solution of sodium hydride (70.9 mg, 1.76 mmol, 60% dispersion in mineral oil) in THF (10 mL) was added dimethyl malonate (0.25 mL, 2.20 mmol) dropwise at 0 °C. After stirring for 1.5 h at room temperature, a solution of **S3d** (465 mg, ca. 0.882 mmol) in THF (20 mL) was added at 0 °C. Then, the reaction mixture was stirred for 6 h at 70 °C. The resulting mixture was diluted with saturated aqueous NH_4Cl (30 mL) and extracted with EtOAc (3 × 30 mL). The combined organic layers were washed with brine, dried over $MgSO_4$ and concentrated *in vacuo*. The crude mixture was purified by column chromatography on silica gel (hexane/EtOAc = 4:1) to afford **3d** (367 mg, 0.71 mmol, 76%) as yellow oil.

^{1}H NMR (600 MHz, $CDCl_3$): δ 8.25 (brs, 1H, NH), 7.99 (dd, *J* = 1.4, 7.8 Hz, 1H), 7.60–7.53 (m, 2H), 7.49 (dd, *J* = 1.4, 7.8 Hz, 1H), 7.34 (m, 1H), 7.33 (m, 1H), 7.16 (dd, *J* = 7.8, 7.8 Hz, 1H), 7.05 (dd, *J* = 7.8, 7.8 Hz, 1H), 5.20 (dd, *J* = 6.9, 6.9 Hz, 1H), 4.27 (dd, *J* = 5.5, 14.7 Hz, 1H), 3.78 (s, 3H), 3.74 (s 3H), 3.56–3.50 (m, 2H), 2.64 (dd, *J* = 4.1, 15.6 Hz, 1H), 2.54 (m, 1H), 2.20–2.09 (m, 2H), 2.01–1.93 (m, 2H).

^{13}C NMR (150 MHz, $CDCl_3$): δ 170.1, 169.7, 148.1, 136.1, 134.1, 133.7, 132.9, 131.8, 130.4, 126.6, 124.3, 122.3, 119.7, 118.2, 111.3, 107.3, 3.4, 52.9, 52.8, 51.0, 40.1, 33.3, 25.3, 20.4.

IR (neat): 3300, 2800, 1800, 1650, 1200 cm^{-1}.

HRMS (ESI): Exact mass calculated for $C_{24}H_{25}N_3NaO_8S$ $[M+Na]^+$: 538.1255; found 538.1254.

Di-*tert*-butyl 2-(2-(2-tosyl-2,3,4,9-tetrahydro-1*H*-pyrido[3,4-*b*]indol-1-yl)ethyl)malonate (3e)

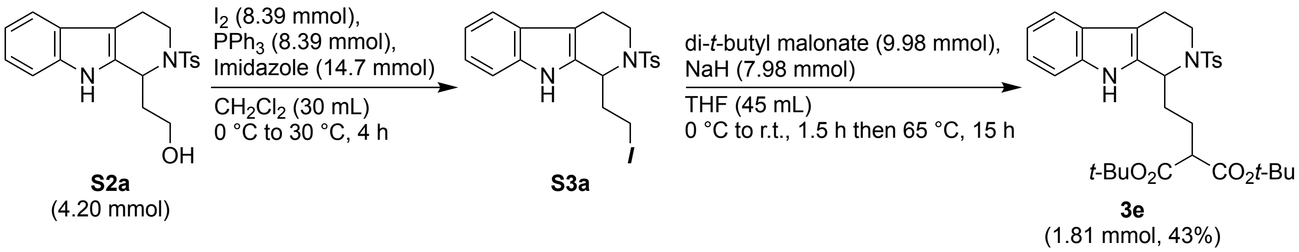

To a solution of **S2a** (1.55 g, 4.20 mmol) in CH_2Cl_2 (30 mL) was added imidazole (1.00 g, 14.69 mmol), triphenylphosphine (2.20 g, 8.39 mmol), and iodine (2.13 g, 8.39 mmol) at 0 °C. After stirring for 4 h at 30 °C in dark, the resulting mixture was diluted with saturated aqueous sodium thiosulfate (30 mL) and extracted with CH_2Cl_2 (3 × 30 mL). The combined organic layers were washed with brine, dried over $MgSO_4$ and concentrated *in vacuo*. The crude mixture was passed through a short pad column (hexane/EtOAc = 5:1) and used for the next step.

To a solution of sodium hydride (319.2 mg, 7.98 mmol, 60% dispersion in mineral oil) in THF (35 mL) was added di-*tert*-butyl malonate (2.2 mL, 9.98 mmol) dropwise at 0 °C. After stirring for 1.5 h at room temperature, a solution of **S3a** (1.92 g, ca. 3.99 mmol) in THF (10 mL) was added at 0 °C. Then, the reaction mixture was stirred for 15 h at 65 °C. The resulting mixture was diluted with saturated aqueous NH_4Cl (45 mL) and extracted with EtOAc (3 × 45 mL). The combined organic layers were washed with brine, dried over $MgSO_4$ and concentrated *in vacuo*. The crude mixture was purified by column chromatography on silica gel (hexane/EtOAc = 10:1) to afford **3e** (1.03 g, 1.81 mmol, 43%) as yellow oil.

^{1}H NMR (600 MHz, $CDCl_3$): δ. 8.21 (brs, 1H, NH), 7.62 (d, *J* = 8.3 Hz, 2H), 7.32 (brd, *J* = 8.3 Hz, 1H), 7.31 (brd, *J* = 8.3 Hz, 1H), 7.14 (ddd, *J* = 0.9, 8.3, 8.3 Hz, 1H), 7.09 (d, *J* = 8.3 Hz, 2H), 7.04 (ddd, *J* = 0.9, 8.3, 8.3 Hz, 1H), 5.11 (dd, *J* = 6.9, 6.9 Hz, 1H), 4.13 (brdd, *J* = 5.5, 14.7 Hz, 1H), 3.39 (ddd, *J* = 4.1, 11.9, 14.7 Hz, 1H), 3.33 (dd, *J* = 7.8, 7.8 Hz, 1H), 2.49 (brdd, *J* = 4.1, 15.6 Hz, 1H), 2.37 (ddd, *J* = 5.5, 11.9, 15.6, 1H), 2.27 (s, 3H), 2.10–2.06 (m, 2H), 1.98–1.90 (m, 2H), 1.49 (s, 9H), 1.46 (s, 9H).

^{13}C NMR (150 MHz, $CDCl_3$): δ 169.6, 168.7, 143.4, 138.1, 136.0, 132.8, 129.6, 126.9, 126.7, 122.1, 119.5, 118.2, 111.1, 107.9, 82.1, 81.8, 53.3, 52.4, 39.9, 33.7, 28.1 (6C), 24.9, 21.6, 19.9.

IR (neat): 3220, 2500, 1775, 1650, 1200 cm^{-1}.

HRMS (ESI): Exact mass calculated for $C_{31}H_{40}N_2NaO_6S$ [M+Na]$^+$: 591.2499; found 591.2497.

Methyl 2-(2-tosyl-2,3,4,9-tetrahydro-1*H*-pyrido[3,4-*b*]indol-1-yl)acetate (S5f)

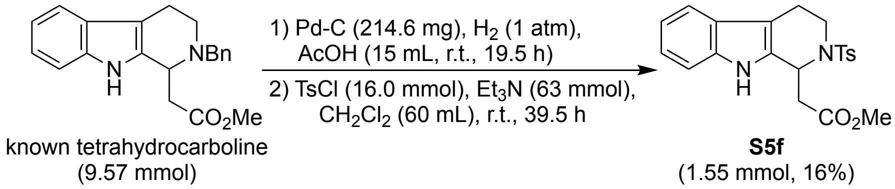

To a solution of known tetrahydrocarboline[4] (618.2 mg, 9.57 mmol) in acetic acid (15 mL) was added Pd/C (10 wt%, 214.6 mg). The reaction mixture was stirred under H_2 atmosphere (1 atm) for 19.5 h at room temperature. The resulting mixture was filtered through a Celite pad, diluted with H_2O (50 mL), and extracted with CH_2Cl_2 (3 × 50 mL). The combined organic layers were washed with brine, dried over $MgSO_4$ and concentrated *in vacuo*. The crude mixture was used without further purification.

To a solution of the crude amine in CH_2Cl_2 (60 mL) was added triethylamine (8.5 mL, 63.0 mmol) and *p*-toluenesulfonyl chloride (3.00 g, 16.0 mmol). After stirring for 19.5 h at room temperature, the resulting mixture was diluted with H_2O (60 mL), and extracted with CH_2Cl_2 (3 × 60 mL). The combined organic layers were washed with brine, dried over $MgSO_4$ and concentrated *in vacuo*. The crude mixture was purified by column chromatography on silica gel (hexane/EtOAc = 7:1) to afford **S5f** as yellow liquid (618.2 mg, 1.55 mmol, 16%).

^{1}H NMR (600 MHz, $CDCl_3$): δ 8.75 (brs, 1H, NH), 7.65 (d, *J* = 6.4 Hz, 2H), 7.34 (brd, *J* = 7.8 Hz, 1H), 7.30 (brd, *J* = 8.3 Hz, 1H), 7.15 (ddd, *J* = 0.9, 7.8, 8.3 Hz, 1H), 7.14 (d, *J* = 6.4 Hz, 2H), 7.05 (ddd, *J* = 0.9, 7.8, 7.8 Hz, 1H), 5.49 (dd, *J* = 3.2, 11.0 Hz, 1H), 4.24 (brdd, *J* = 5.0, 14.2 Hz, 1H), 3.77 (s, 3H), 3.35 (ddd, *J* = 4.1, 11.9, 14.2 Hz, 1H), 3.15 (brdd, *J* = 4.1, 17.4 Hz, 1H), 3.02 (brdd, *J* = 11.9, 17.4 Hz, 1H), 2.57 (brdd, *J* = 3.2, 15.6 Hz, 1H), 2.48 (m, 1H), 2.30 (s, 3H).

^{13}C NMR (150 MHz, $CDCl_3$): δ 173.5, 143.6, 137.9, 135.7, 132.0, 129.8, 126.8, 126.3, 122.4, 119.5, 118.3, 111.2, 107.8, 52.4, 48.9, 41.1, 40.4, 21.6, 20.3.

IR (neat): 3300, 2550, 1775, 1600, 1300 cm^{-1}.

HRMS (ESI): Exact mass calculated for $C_{21}H_{22}N_2NaO_4S$ $[M+Na]^+$: 421.1192; found 421.1190.

Methyl 3-oxo-4-(2-tosyl-2,3,4,9-tetrahydro-1*H*-pyrido[3,4-*b*]indol-1-yl)butanoate (3f)

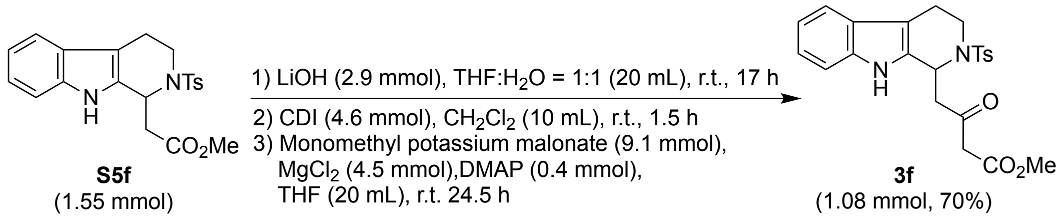

Compound **3f** was prepared according to the reported procedures.[3] To a solution of **S5f** (618.2 mg, 1.55 mmol) in THF/H_2O = 1:1 (20 mL) was added lithium hydroxide (69.0 mg, 2.9 mmol) at 0 °C. After stirring for 17 h at room temperature, the reaction mixture was diluted with 3 M HClaq (20 mL) and extracted with EtOAc (3 × 20 mL). The combined organic layers were washed with brine, dried over $MgSO_4$ and concentrated *in vacuo*. The crude mixture was used without further purification.

To a solution of the carboxylic acid (574 mg, ca. 1.5 mmol) in CH_2Cl_2 (10 mL) was added 1,1'-carbonyldiimidazole (747.7 mg, 4.5 mmol) at room temperature. After stirring for 1.5 h, the reaction mixture was concentrated *in vacuo* and dissolved in THF (20 mL). To the solution of activate ester was added magnesium chloride (430.8 mg, 4.5 mmol), monomethyl potassium malonate (1.43 g, 9.1 mmol), and 4-dimethylaminopyridine (51.7 mg, 0.4 mmol) at 0 °C. After stirring for 24.5 h at room temperature, the resulting mixture was diluted with saturated aqueous NH_4Cl (20 mL) and extracted with EtOAc (3 × 20 mL). The combined organic layers were washed with brine, dried over $MgSO_4$ and concentrated *in vacuo*. The crude mixture was purified by column chromatography on silica gel (hexane/EtOAc = 4:1) to afford **3e** (475.5 mg, 1.08 mmol, 70% in 3 steps) as yellow oil.

^{1}H NMR (600 MHz, $CDCl_3$): δ. 8.55 (brs, 1H, NH), 7.64 (d, *J* = 8.3 Hz, 2H), 7.33 (brd, *J* = 8.3 Hz, 1H), 7.30 (brd, *J* = 8.3 Hz, 1H), 7.15 (ddd, *J* = 0.9, 6.9, 8.3 Hz, 1H), 7.14 (d, *J* = 8.3 Hz, 2H), 7.04 (ddd, *J* = 0.9, 6.9, 8.3 Hz, 1H), 5.51 (dd, *J* = 1.4, 9.6 Hz, 1H), 4.24 (brdd, *J* = 5.5, 14.2 Hz, 1H), 3.74 (s, 3H), 3.58 (d, *J* = 16.1 Hz, 1H), 3.53 (d, *J* = 16.1 Hz, 1H), 3.41 (ddd, *J* = 2.8, 14.2, 14.2 Hz, 1H), 3.36–3.31 (m, 2H), 2.57 (brdd, *J* = 4.6, 15.6 Hz, 1H), 2.46 (m, 1H), 2.30 (s, 3H).

^{13}C NMR (150 MHz, $CDCl_3$): δ 203.6, 167.0, 143.7, 137.7, 135.7, 131.9, 129.9, 126.8, 126.3, 122.4, 119.6, 118.2, 111.3, 107.8, 52.8, 50.8, 49.3, 48.3, 40.6, 21.6, 20.3.

IR (neat): 3380, 3330, 2475, 2300, 1800 cm^{-1}.

HRMS (ESI): Exact mass calculated for $C_{23}H_{24}N_2NaO_5S$ $[M+Na]^+$: 463.1298; found 463.1297.

Dimethyl 2-(2-(6-methoxy-2-tosyl-2,3,4,9-tetrahydro-1*H*-pyrido[3,4-*b*]indol-1-yl)ethyl)malonate (3g)

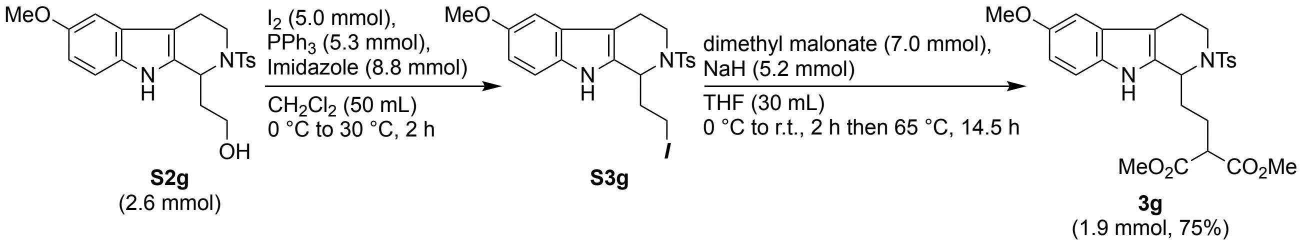

To a solution of imidazole (602 g, 8.85 mmol), triphenylphosphine (1.38 g, 5.26 mmol), and iodine (1.28 g, 5.04 mmol) in CH_2Cl_2 (30 mL) was added a solution of **S2g** (1.04 g, 2.60 mmol) in CH_2Cl_2 (20 mL) at 0 °C. After stirring for 2 h at 30 °C in dark, the resulting mixture was diluted with saturated aqueous sodium thiosulfate (100 mL) and extracted with CH2Cl2 (3 × 100 mL). The combined organic layers were washed with brine, dried over MgSo4 and concentrated *in vacuo*. The crude mixture was passed through a short pad column on silica gel (hexane/EtOAc = 2:1) and used for the next step.

To a solution of sodium hydride (206 mg, 5.15 mmol, 60% dispersion in mineral oil) in THF (20 mL) was added dimethyl malonate (0.8 mL, 7.0 mmol) dropwise at 0 °C. After stirring for 2 h at 30 °C, a solution of **S3g** (1.27 g, ca. 2.49 mmol) in THF (10 mL) was added at 0 °C. Then, the reaction mixture was stirred for 14.5 h at 65 °C. The resulting mixture was diluted with saturated aqueous NH_4Cl (50 mL) and extracted with EtOAc (3 × 50 mL). The combined organic layers were washed with brine, dried over $MgSO_4$ and concentrated *in vacuo*. The crude mixture was purified by column chromatography on silica gel (hexane/EtOAc = 4:1, 3:1, 2:1 to 1:1) to afford **3g** (993 mg, 1.93 mmol, 75%) as colorless solid.

^{1}H NMR (600 MHz, $CDCl_3$): δ 7.94 (brs, 1H, NH), 7.62 (d, *J* = 8.3 Hz, 2H), 7.19 (d, *J* = 9.0 Hz, 1H), 7.09 (d, *J* = 8.3 Hz, 2H), 6.79 (dd, *J* = 2.8, 9.0 Hz, 1H), 6.73 (d, *J* = 2.8 Hz, 1H), 5.11 (dd, *J* = 7.6, 7.6 Hz, 1H), 4.13 (brdd, *J* = 5.5, 15.2 Hz, 1H), 3.80 (s, 3H), 3.77 (s, 3H), 3.74 (s, 3H), 3.54 (dd, *J* = 7.6, 7.6 Hz, 1H), 3.37 (ddd, *J* = 4.8, 12.4, 15.2 Hz, 1H), 2.43 (brdd, *J* = 4.8, 15.2 Hz, 1H), 2.31 (m, 1H), 2.28 (s, 3H), 2.19–2.15 (m, 2H), 1.93–1.87 (m, 2H).

^{13}C NMR (150 MHz, $CDCl_3$): δ 170.1, 169.8, 154.1, 143.5, 138.0, 133.4, 131.1, 129.7, 127.1, 126.9, 111.9, 111.8, 107.8, 100.6, 56.0, 52.9, 52.8, 52.6, 50.9, 39.8, 33.3, 25.2, 21.6, 19.8.

IR (neat): 3380, 2950, 1730, 1430, 1150 cm^{-1}.

HRMS (ESI): Exact mass calculated for $C_{26}H_{30}N_2NaO_7S$ $[M+Na]^+$: 537.1666; found 537.1667.

Melting Point: 64.8–67.3 °C

3. Anodic oxidation (mavacurane-type alkaloid skeleton)

General procedure of anodic oxidation for the mavacurane skeleton

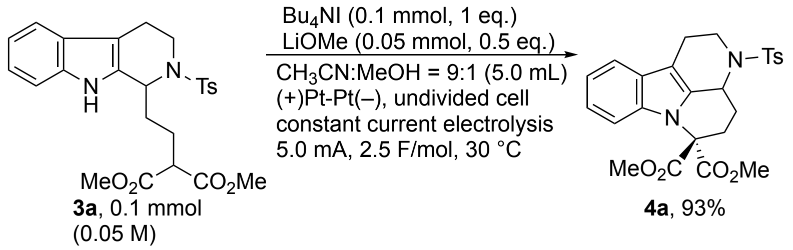

The undivided cell was charged with cyclization precursor **3a** (48.5 mg, 0.1 mmol), tetrabutylammonium iodide (38.2 mg, 0.1 mmol), and lithium methoxide (1.9 mg, 0.05 mmol) in $CH_3CN/MeOH$ = 9:1 (5.0 mL). A constant current (5.0 mA, 2.5 F/mol) was supplied at 30 °C with vigorous stirring using platinum anode and cathode. After the electrolysis, the reaction mixture was diluted with saturated aqueous NH_4Cl (10 mL) and extracted with EtOAc (3 × 10 mL). The combined organic layers were washed with brine, dried over Na_2SO_4 and concentrated *in vacuo*. The crude mixture was purified by column chromatography on silica gel (hexane/EtOAc = 4:1) to afford product **4a**[3] (45.2 mg, 93 μmol, 93%) as yellow oil.

Dimethyl 3-tosyl-1,2,3,3a,4,5-hexahydro-6*H*-indolo[3,2,1-*de*][1,5]naphthyridine-6,6-dicarboxylate (4a)[3]

^{1}H NMR (600 MHz, $CDCl_3$): δ 7.74 (d, *J* = 8.2 Hz, 2H), 7.37 (d, *J* = 7.3 Hz, 1H), 7.31 (d, *J* = 8.2 Hz, 2H), 7.13–7.08 (m, 3H), 4.48 (dd, *J* = 4.6, 11.9 Hz, 1H), 3.90 (s, 3H), 3.78 (m, 1H), 3.61 (s, 3H), 3.53 (ddd, *J* = 3.7, 8.7, 12.6 Hz, 1H), 2.91 (ddd, *J* = 2.8, 4.1, 14.7 Hz, 1H), 2.72(ddd, *J* = 3.7, 4.1, 14.7 Hz, 1H), 2.64(m, 1H), 2.47 (ddd, *J* = 2.8, 12.6, 16.5 Hz, 1H), 2.41 (s, 3H), 2.35 (m, 1H), 2.00 (m, 1H).

^{13}C NMR (150 MHz, $CDCl_3$): δ 169.5, 168.0, 143.9, 137.9, 136.7, 131.7, 130.1, 127.6, 127.3, 122.4, 120.7, 118.4, 112.6, 109.9, 68.4, 53.7, 53.3, 53.1, 44.7, 32.1, 27.4, 21.7, 21.2.

IR (neat): 2925, 2350, 1750, 1400, 1150 cm^{-1}.

3-(*tert*-Butyl) 6,6-dimethyl 1,3a,4,5-tetrahydro-3*H*-indolo[3,2,1-*de*][1,5]naphthyridine-3,6,6(2*H*)-tricarboxylate (4b)

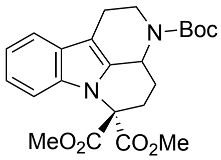

4b

Compound **4b** was prepared according to the general procedure from **3b** (43.5 mg, 0.1 mmol). The crude was purified by column chromatography on silica gel (hexane/EtOAc = 4:1) to afford **4b** (mixture of rotamers, 37.2 mg, 86 μmol, 86%) as yellow oil.

^{1}H NMR (600 MHz, $CDCl_3$): δ 7.48 (m, 1H), 7.18–7.12 (m, 3H), 4.81 (dd, *J* = 2.4, 12.1 Hz, 1H), 4.40 (dd, *J* = 11.0, 11.0 Hz, 1H), 3.91 (s, 3H), 3.61 (s, 3H), 2.93–2.86 (m, 2H), 2.80 (dd, *J* = 2.4, 12.8 Hz, 1H), 2.71 (m, 1H), 2.53–2.45 (m, 2H), 2.74 (m, 1H), 1.52 (s, 9H).

^{13}C NMR (150 MHz, $CDCl_3$, only major rotamer was assigned): δ 169.7, 168.3, 155.3, 138.0, 133.5, 128.1, 121.9, 120.5, 118.3, 112.5, 110.7, 80.3, 68.6, 53.6, 53.1, 50.8, 41.1, 32.4, 28.7, 25.3, 22.4.

IR (neat): 2800, 2000, 1750, 1650, 950 cm^{-1}.

HRMS (ESI): Exact mass calculated for $C_{23}H_{28}N_2NaO_6$ $[M+Na]^+$: 451.1840; found 451.1833.

3-Benzyl 6,6-dimethyl 1,3a,4,5-tetrahydro-3*H*-indolo[3,2,1-de][1,5]naphthyridine (4c)

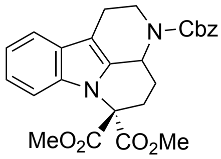

4c

Compound **4c** was prepared according to the general procedure from **3c** (46.5 mg, 0.1 mmol). The crude was purified by column chromatography on silica gel (hexane/EtOAc = 4:1) to afford **4c** (mixture of rotamers, 37.0 mg, 80 μmol, 80%) as yellow oil and recovery of **3c** (5.4 mg, 12 μmol, 12%).

^{1}H NMR (600 MHz, $CDCl_3$): δ 7.46 (dd, J = 4.6, 4.6 Hz, 1H), 7.40–7.37 (m, 4H), 7.34 (m, 1H), 7.17–7.13 (m, 3H), 5.25 (d, J = 13.4 Hz, 1H), 5.22 (d, J = 13.4 Hz, 1H), 4.89 (brd, J = 9.6 Hz, 1H), 4.49 (brd, J = 11.0 Hz, 1H), 3.90 (s, 3H), 3.60 (s, 3H), 3.00 (brdd, J = 11.0, 12.4 Hz,), 2.87 (brd, J = 12.4 Hz, 1H), 2.82 (brd, J = 15.1 Hz, 1H), 2.74 (brdd, J = 12.4, 15.1 Hz, 1H), 2.53–2.49 (m, 2H), 1.76 (brddd, J = 10.6, 12.8, 12.8 Hz, 1H).

^{13}C NMR (150 MHz, $CDCl_3$, only major rotamer was assigned): δ 169.7, 168.2, 156.0, 138.1, 136.8, 133.1, 128.7, 128.3, 128.1, 128.0, 122.1, 120.6, 118.4, 112.6, 110.6, 68.6, 67.5, 53.7, 53.2, 51.0, 41.6, 32.2, 25.2, 22.4.

IR (neat): 1760, 1700, 1650, 1150, 800 cm^{-1}.

HRMS (ESI): Exact mass calculated for $C_{26}H_{26}N_2NaO_6$ $[M+Na]^+$: 485.1683; found 485.1680.

Di-*tert*-butyl 3-tosyl-1,2,3,3a,4,5-hexahydro-6*H*-indolo[3,2,1-*de*] [1,5]naphthyridine-6,6-dicarboxylate (4e)

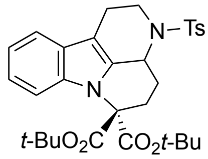

4e

Compound **4e** was prepared according to the general procedure from **3e** (56.9 mg, 0.1 mmol). The crude was purified by column chromatography on silica gel (hexane/EtOAc = 7:1) to afford **4e** (yellow oil, 18.5 mg, 33 μmol, 33%), **9e** (yellow oil, 18.2 mg, 18 μmol, 18%), and recovery of **3e** (25.9 mg, 46 μmol, 46%).

Di-*tert*-butyl 3-tosyl-1,2,3,3a,4,5-hexahydro-6*H*-indolo[3,2,1-*de*] [1,5]naphthyridine-6,6-dicarboxylate (4e)

^{1}H NMR (600 MHz, CDCl$_3$): δ 7.75 (d, *J* = 8.7 Hz, 2H), 7.33 (d, *J* = 7.8 Hz, 1H), 7.29 (d, *J* = 8.7 Hz, 2H), 7.29 (d, *J* = 7.8 Hz, 1H), 7.10 (dd, *J* = 7.8, 7.8 Hz, 1H), 7.05 (dd, *J* = 7.8, 7.8 Hz, 1H), 4.56 (dd, *J* = 4.1, 11.9 Hz, 1H), 3.86 (ddd, *J* = 4.1, 4.1, 13.3 Hz, 1H), 3.44 (ddd, *J* = 3.7, 9.1, 13.3 Hz, 1H), 2.81 (ddd, *J* = 4.1, 9.1, 14.7 Hz, 1H), 2.67 (ddd, *J* = 3.7, 4.1, 14.7 Hz, 1H), 2.55 (m, 1H), 2.40 (s, 3H), 2.38 (m, 1H), 2.30 (m, 1H), 2.05 (m, 1H), 1.58 (s, 9H), 1.17 (s, 9H).

^{13}C NMR (150 MHz, CDCl$_3$): δ 168.1, 166.4, 143.7, 138.5, 137.1, 132.4, 130.1, 128.0, 127.2, 121.8, 120.4, 117.9, 114.1, 109.8, 83.7, 83.3, 69.6, 53.0, 44.2, 32.3, 28.1, 27.7, 27.6, 21.7, 21.2.

IR (neat): 2400, 2350, 1200, 800, 725 cm^{-1}.

HRMS (ESI): Exact mass calculated for $C_{31}H_{38}N_2NaO_6S$ [M+Na]$^+$: 589.2343; found 589.2339.

Di-*tert*-butyl 2-iodo-2-(2-(2-tosyl-2,3,4,9-tetrahydro-1*H*-pyrido[3,4-b]indol-1-yl)ethyl)malonate (9e)

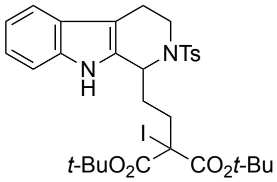

9e

^{1}H NMR (600 MHz, CDCl$_3$): δ 7.84 (brs, 1H, NH), 7.64 (d, *J* = 8.3 Hz, 2H), 7.33–7.31 (m, 2H), 7.16 (dd, *J* = 7.8, 7.8 Hz, 1H), 7.11 (d, *J* = 8.3 Hz, 2H), 7.05 (dd, *J* = 7.8, 7.8 Hz, 1H), 5.14 (dd, *J* = 4.6, 7.8 Hz, 1H), 4.17 (dd, *J* = 5.5, 14.7 Hz, 1H), 3.37 (ddd, *J* = 4.6, 11.9, 14.7 Hz, 1H), 2.52 (dd, *J* = 3.7, 15.6 Hz, 1H), 2.40 (ddd, *J* = 4.6, 10.6, 15.6 Hz, 1H), 2.36–2.30 (m, 2H), 2.29 (s, 3H), 2.07 (m, 1H), 1.96 (m, 1H), 1.47 (s, 9H), 1.46 (s, 9H).

^{13}C NMR (150 MHz, CDCl$_3$): δ 167.1, 166.8, 143.5, 138.0, 136.1, 132.2, 129.7, 127.0, 126.7, 122.3, 119.7, 118.3, 111.1, 108.6, 83.7, 83.7, 52.7, 49.7, 40.2, 36.6, 34.5, 28.1, 27.8, 21.6, 20.0.

IR (neat): 2400, 2300, 1575, 800, 725 cm^{-1}.

HRMS (ESI): Exact mass calculated for $C_{31}H_{39}IN_2NaO_6S$ [M+Na]$^+$: 717.1466; found 717.1460.

Dimethyl 10-methoxy-3-tosyl-1,2,3,3a,4,5-hexahydro-6*H*-indolo[3,2,1-*de*][1,5]naphthyridine-6,6-dicarboxylate (4g)

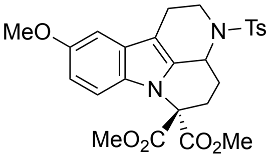

4g

Compound **4g** was prepared according to the general procedure from **3g** (51.1 mg, 0.1 mmol). The crude was purified by column chromatography on silica gel (hexane/EtOAc = 4:1 to 2:1) to afford **4g** (46.6 mg, 90.9 μmol, 91%) as yellow solid.

^{1}H NMR (600 MHz, $CDCl_3$): δ 7.74 (d, *J* = 8.3 Hz, 2H), 7.30 (d, *J* = 8.3 Hz, 2H), 7.03 (d, *J* = 9.0 Hz, 1H), 6.81 (d, *J* = 2.8 Hz, 1H), 6.76 (dd, *J* = 2.8, 9.0 Hz, 1H), 4.47 (dd, *J* = 4.8, 16.5 Hz, 1H), 3.90 (s, 3H). 3.80 (s, 3H), 3.78 (m, 1H), 3.61 (s, 3H), 3.52 (ddd, *J* = 3.4, 8.3, 12.4 Hz, 1H). 2.88 (ddd, *J* = 2.8, 4.1, 14.5 Hz, 1H), 2.68 (ddd, *J* = 4.1, 4.1, 14.5 Hz, 1H), 2.61 (m, 1H), 2.45 (ddd, *J* = 4.8, 4.8, 14.5 Hz, 1H), 2.41 (s, 3H), 2.32 (m, 1H), 2.00 (ddd, *J* = 2.1, 14.5, 14.5 Hz, 1H).

^{13}C NMR (150 MHz, $CDCl_3$): δ 169.6, 168.0, 154.7, 143.8, 136.6, 132.8, 132.5, 130.1, 128.3, 127.2, 113.4, 111.8, 109.8, 100.6, 68.4, 55.8, 53.7, 53.2, 53.0, 44.6, 31.9, 27.3, 21.6, 21.3.

IR (neat): 2952, 1737, 1159, 1033, 800 cm^{-1}.

HRMS (ESI): Exact mass calculated for $C_{26}H_{28}N_2NaO_7S$ $[M+Na]^+$: 535.1509; found 535.1495.

Melting Point: 80.3–84.2 °C

Scale-up synthesis (mavacurane-type alkaloid skeleton)

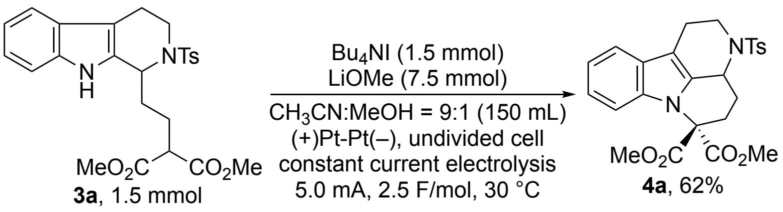

The anodic oxidation was carried out in a 200 mL round bottom flask equipped with a Pt anode (1 cm × 1 cm, 0.20 mm thickness) and a Pt cathode (1 cm × 1 cm, 0.20 mm thickness). The flask was charged with cyclization precursor **3a** (727.7 mg, 1.5 mmol), tetrabutylammonium iodide (554.1 mg, 1.5 mmol), and lithium methoxide (28.5 mg, 0.75 mmol) in CH_3CN/MeOH = 9:1 (150 mL). A constant current (5.0 mA, 2.5 F/mol) was supplied at 30 °C with vigorous stirring. After the electrolysis, the resulting mixture was diluted with saturated aqueous NH_4Cl (150 mL) and extracted with EtOAc (3 × 100 mL). The combined organic layers were washed with brine, dried over Na_2SO_4 and concentrated *in vacuo*. The crude mixture was purified by column chromatography on silica gel (hexane/EtOAc = 4:1) to afford product **4a** (446.8 mg, 0.93 mmol, 62%) as yellow oil with a recovery of **3a** (12 mg, 25 μmol, 2%).

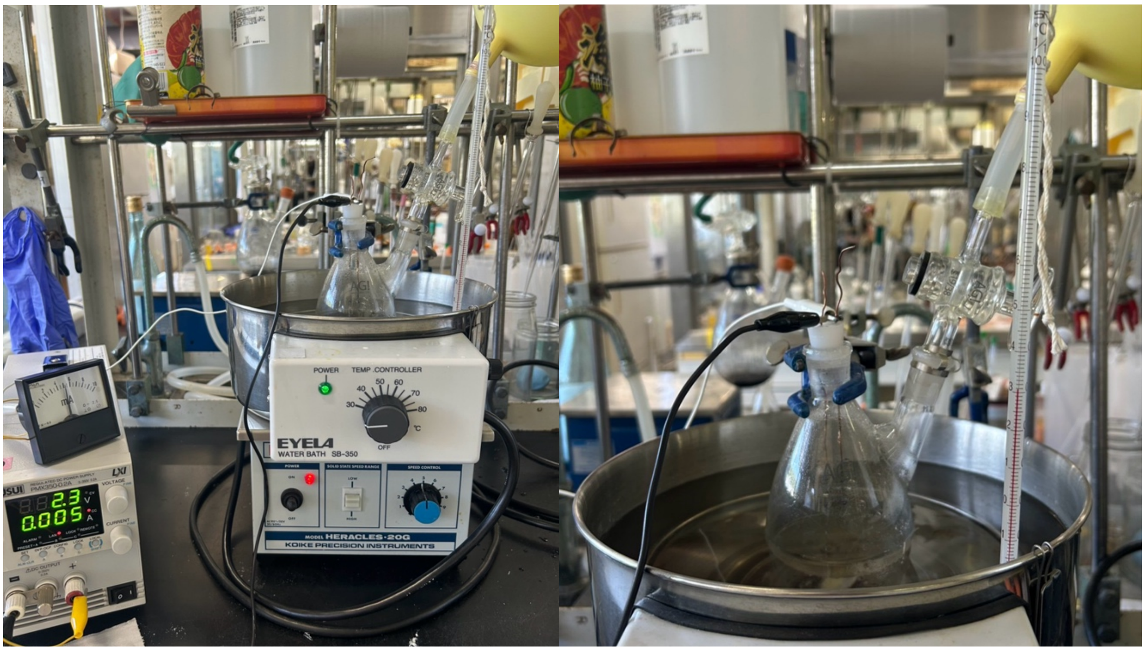

Figure S2. Scale-up synthesis (mavacurane skeleton)

Miscellaneous optimization of the reaction conditions (mavacurane-type alkaloid skeleton)

Table S1. Amount of Bu_4NI

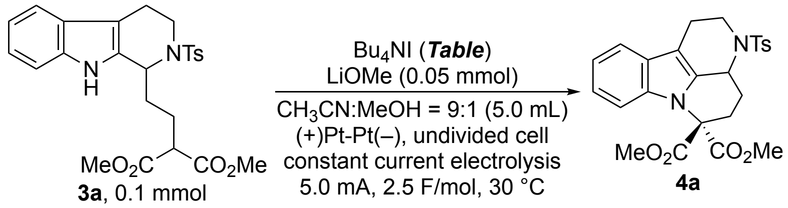

Entry	Bu_4NI	Yield of **4a** (%)	Recovery of **3a** (%)
1	0.5 mmol	88	trace
2	0.1 mmol	93	Not Detected
3	0.05 mmol	66	22
4	0.01 mmol	17	14

Table S2. Screening of mediator

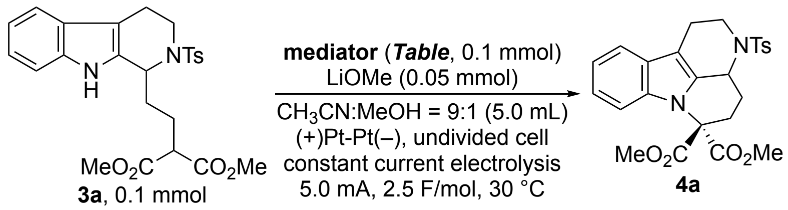

Entry	Mediator	Yield of **4a** (%)	Recovery of **3a** (%)
1	Bu_4NI	93	Not Detected
2	Bu_4NBr	68	trace
3[a]	Bu_4NCl	Not Detected	22
4	NaI	69	Not Detected
5[b]	LiI	20	Not Detected

[a] Product **8a** was detected. (**Figure S3.**) [b] Iodinated side product **9** was obtained in 44%.

Table S3. Screening of base

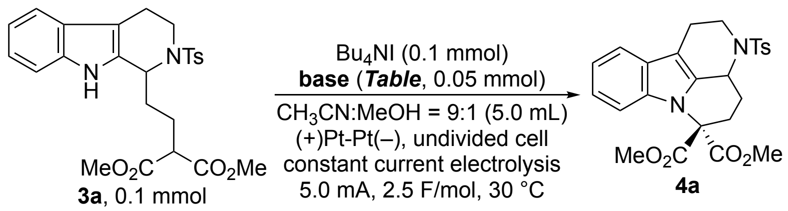

Entry	base (0.05 mmol)	Yield of **4a** (%)	Recovery of **3a** (%)
1	LiOMe	93	Not Detected
2	Li_2CO_3	57	29
3	none	85	13

Table S4. Screening of amount of electricity

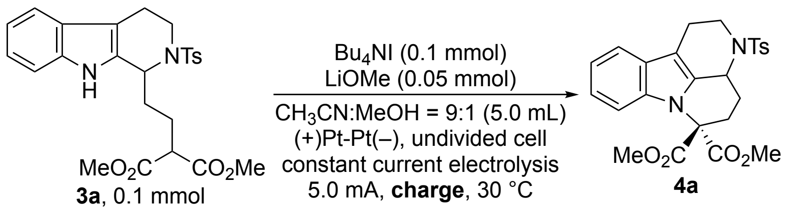

Entry	Charge (F/mol)	Yield of **4a** (%)	Recovery of **3a** (%)
1	2.5	93	Not Detected
2	2.0	66	33

Table S5. Screening of solvent

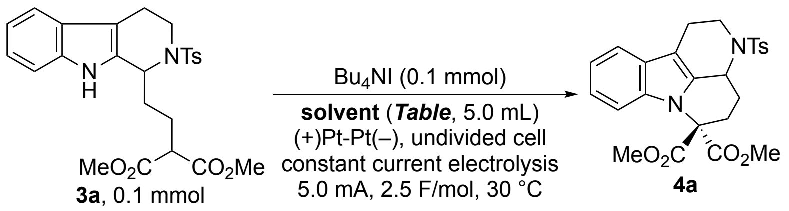

Entry	Solvent	Yield of **4a** (%)	Recovery of **3a** (%)
1	CH_3CN:MeOH = 9:1	85	13
2	CH_3CN	71	Not Detected

Electrolysis with Bu_4NCl as mediator

When tetrabutylammonium chloride (Bu_4NCl) instead of Bu_4NI was used as a mediator, mavacurane-type skeleton **4a** was not obtained. The NMR analysis suggests that trace amount of akuammiline-type product **8a** was obtained (Figure S3).

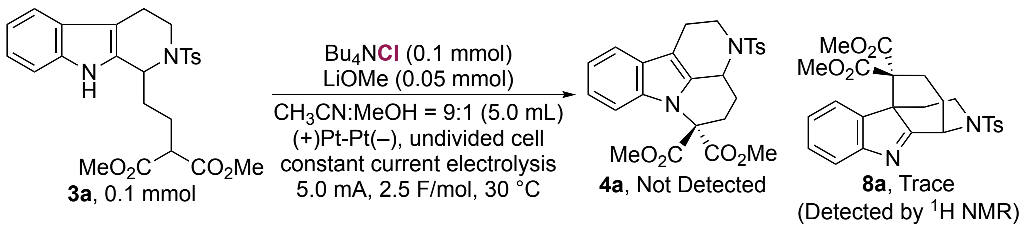

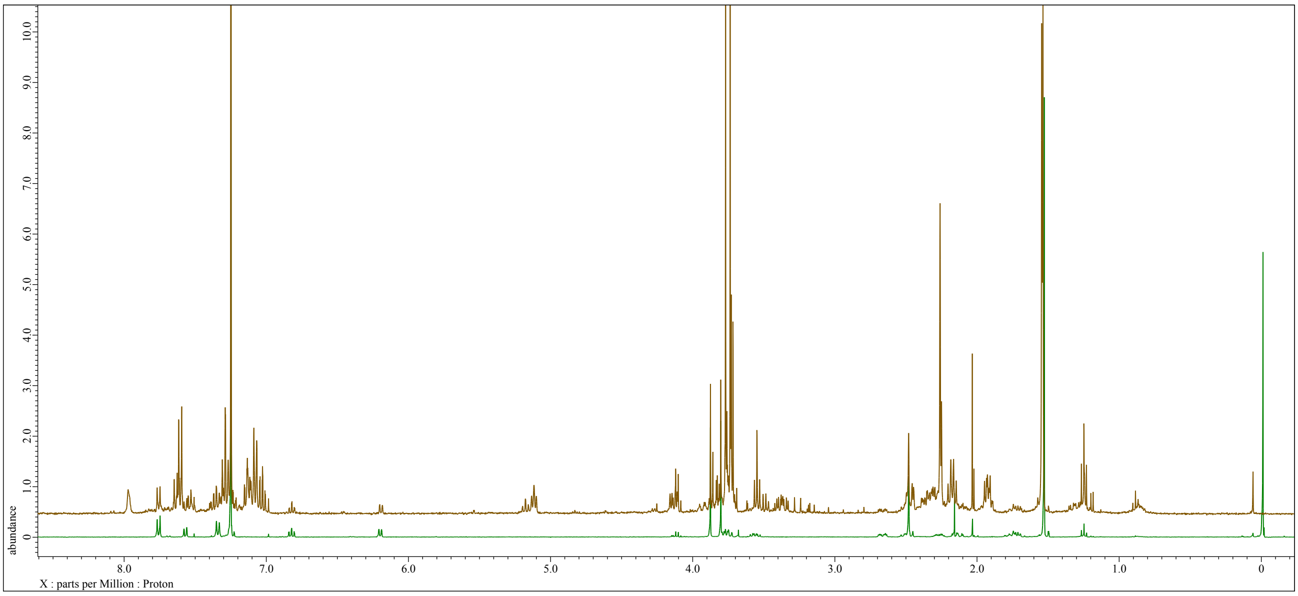

Figure S3. ^{1}H NMR of crude mixture (top) and **8a** (bottom)

4. Anodic oxidation (akuammiline-type alkaloid skeleton)

General procedure of anodic oxidation

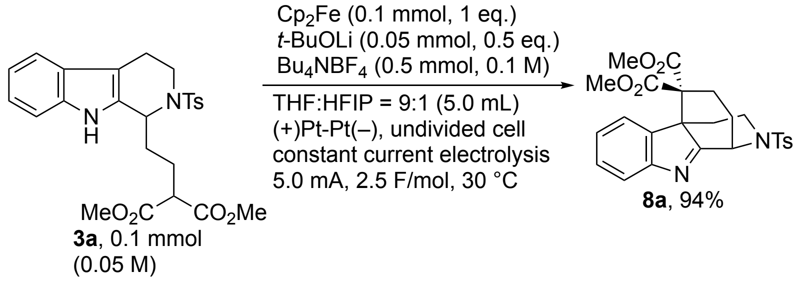

An undivided cell was charged with cyclization precursor **3a** (48.5 mg, 0.1 mmol), tetrabutylammonium tetrafluoroborate (164.6 mg, 0.1 mmol), ferrocene (18.6 mg, 0.1 mmol), and lithium *tert*-butoxide (4.0 mg, 0.05 mmol) in THF/HFIP = 9:1 (5.0 mL). A constant current (5.0 mA, 2.5 F/mol) was supplied at 30 °C with vigorous stirring. After the electrolysis, the reaction mixture was diluted with saturated aqueous NH_4Cl (10 mL) and extracted with EtOAc (3 × 10 mL). The combined organic layers were washed with brine, dried over Na_2SO_4 and concentrated *in vacuo*. The crude mixture was purified by column chromatography on silica gel (hexane/EtOAc = 4:1) to afford product **8a** (45.1 mg, 94 μmol, 94%) as yellow oil.

Dimethyl 12-tosyl-2,3-dihydro-1,4a-(epiminoethano)carbazole-4,4(1*H*)-dicarboxylate (8a)

^{1}H NMR (600 MHz, CDCl$_3$): δ 7.77 (d, *J* = 8.2 Hz, 2H), 7.58 (d, *J* = 7.3 Hz, 1H), 7.36 (d, *J* = 8.2 Hz, 2H), 7.25 (dd, *J* = 7.3, 7.3 Hz, 1H), 6.83 (dd, *J* = 7.3, 7.3 Hz, 1H), 6.18 (d, *J* = 7.3 Hz, 1H), 3.88 (s, 3H), 3.81 (s, 3H), 3.79–3.74 (m, 2H), 3.56 (m, 1H), 2.68 (m, 1H), 2.50 (m, 1H), 2.49 (s, 3H), 2.28 (m, 1H), 2.15 (m, 1H), 1.80–1.71 (m, 2H).

^{13}C NMR (150 MHz, CDCl$_3$): δ 176.8, 169.0, 168.8, 152.9, 144.0, 142.9, 135.7, 130.1, 128.4, 127.5, 126.2, 121.8, 121.1, 65.6, 62.6, 61.3, 53.6, 53.4, 46.2, 32.9, 30.2, 28.8, 21.7.

IR (neat): 3600, 2900, 2350, 1750, 1210 cm^{-1}.

HRMS (ESI): Exact mass calculated for $C_{25}H_{26}N_2NaO_6S$ [M+Na]$^+$: 505.1404; found 505.1397.

Dimethyl 2-hydroxy-2-(2-(2-tosyl-2,3,4,9-tetrahydro-1*H*-pyrido[3,4-*b*]indol-1-yl)ethyl)malonate (10)

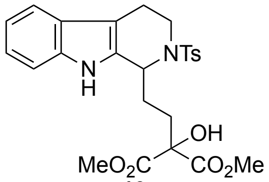

10

^{1}H NMR (600 MHz, CDCl$_3$): δ 8.18 (brs, 1H, NH), 7.61 (d, *J* = 8.7 Hz, 2H), 7.30–7.29 (m, 2H), 7.14 (ddd, *J* = 0.9, 7.8, 8.3 Hz, 1H), 7.08 (d, *J* = 8.7 Hz, 2H), 7.03 (ddd, *J* = 0.9, 7.8, 7.8 Hz, 1H), 5.18 (dd, *J* = 6.0, 7.8 Hz, 1H), 4.15 (brdd, *J* = 5.5, 14.7 Hz, 1H), 3.99 (brs, 1H, OH), 3.87 (s, 3H), 3.81 (s, 3H), 3.39 (ddd, *J* = 4.6, 12.4, 14.7 Hz, 1H), 2.49 (brdd, *J* = 4.6, 15.6 Hz, 1H), 2.41–2.32 (m, 3H), 2.26 (s, 3H), 2.00 (m, 1H), 1.94 (m, 1H).

^{13}C NMR (150 MHz, CDCl$_3$): 170.9, 170.7, 143.3, 137.8, 135.9, 132.6, 129.4, 126.7, 126.4, 121.8, 119.1, 117.9, 111.2, 107.1, 79.1, 53.6, 53.5, 53.0, 39.5, 31.7, 29.5, 21.3, 19.5.

IR (neat): 3800, 3150, 2300, 1550, 775 cm^{-1}.

HRMS (ESI): Exact mass calculated for $C_{25}H_{28}N_2NaO_7S^+$ [M+Na]$^+$: 523.1509; found 523.1506.

12-(*tert*-Butyl) 4,4-dimethyl 2,3-dihydro-1,4a-(epiminoethano)carbazole-4,4,12(1*H*)-tricarboxylate (8b)

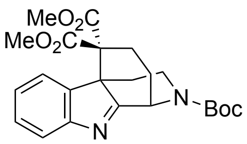

8b

Compound **8b** was prepared according to the general procedure from **3b** (43.0 mg, 0.1 mmol) with 0.2 mmol of *t*-BuOLi instead of 0.05 mmol. The crude was purified by column chromatography on silica gel (hexane/EtOAc = 4:1) to afford **8b** (mixture of rotamers, 36.2 mg, 84 μmol, 84%) as yellow oil.

^{1}H NMR (600 MHz, CDCl$_3$): δ 7.68–7.66 (m, 1H), 7.39–7.35 (m, 1H), 7.24–7.22 (m, 1.7 H), 7.18 (d, *J* = 7.23 Hz, 0.3H), 3.92 (m, 3H), 3.89–3.85 (m, 1H), 3.83 (m, 3H), 3.80–3.66 (m, 2H), 2.74–2.89 (m, 1H), 2.49–2.32 (m, 1H), 2.20–2.17 (m, 1H), 2.14–2.07 (m, 1H), 1.81–1.76 (m, 1H), 1.55–1.49 (m, 3.7H), 1.44 (s, 6.3H).

^{13}C NMR (150 MHz, CDCl$_3$, only major rotamer was assigned): δ 177.4, 169.0, 168.8, 154.2, 152.8, 143.7, 128.4, 126.7, 121.8, 121.4, 80.3, 64.2, 62.8, 61.5, 53.6, 53.4, 44.0, 31.6, 31.2, 28.5, 26.3.

IR (neat): 3800, 1850, 1720, 1650, 1300 cm^{-1}.

HRMS (ESI): Exact mass calculated for $C_{23}H_{28}N_2O_6Na$ [M+Na]$^+$: 451.1840; found 451.1841.

12-Benzyl 4,4-dimethyl 2,3-dihydro-1,4a-(epiminoethano)carbazole-4,4,12(1*H*)-tricarboxylate (8c)

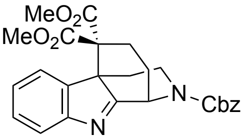

8c

Compound **8c** was prepared according to the general procedure from **3c** (46.4 mg, 0.1 mmol). The crude was purified by column chromatography on silica gel (hexane/EtOAc = 4:1) to afford **8c** (mixture of rotamers, 36 mg, 77 μmol, 77%) as yellow oil.

^{1}H NMR (600 MHz, CDCl$_3$): δ 7.66 (d, J = 6.9 Hz, 1H), 7.43–7.28 (m, 1.7H), 7.37–7.34 (m, 1.5H), 7.33–7.27 (m, 2.8H), 7.23–7.17 (m, 1.6H), 7.13 (d, J = 7.3 Hz, 0.4H), 5.21 (s, 0.8H), 5.18 (d, J = 12.4 Hz, 0.6H), 5.13 (d, J = 12.4 Hz, 0.6H), 3.96–3.89 (m, 4H), 3.88–3.83 (m, 4H), 3.77 (dd, J = 10.1, 10.1 Hz, 1H), 2.71 (dddd, J = 3.7, 10.6, 14.2, 14.2 Hz, 1H), 2.46 (m, 1H), 2.35 (m, 0.4H), 2.18 (m, 0.6H), 2.10 (m, 1H), 1.82 (ddd, J = 7.3, 13.3, 13.3 Hz, 1H), 1.54 (m, 1H).

^{13}C NMR (150 MHz, CDCl$_3$, only major rotamer was assigned): δ 177.2, 168.9, 168.7, 154.6, 152.8, 143.4, 136.5, 128.6, 128.5, 128.2, 127.8, 126.7, 121.9, 121.4, 67.2, 64.0, 62.8, 61.5, 55.6, 53.4, 44.5, 32.4, 31.1, 26.4.

IR (neat): 3125, 2800, 1780, 1625, 1520 cm^{-1}.

HRMS (ESI): Exact mass calculated for $C_{26}H_{26}N_2NaO_6$ $[M+Na]^+$: 485.1683; found 485.1681.

Dimethyl 12-((4-nitrophenyl)sulfonyl)-2,3-dihydro-1,4a-(epiminoethano)carbazole-4,4(1*H*)-dicarboxylate (8d)

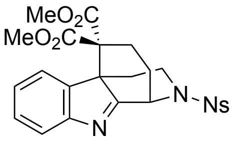

8d

Compound **8d** was prepared according to the general procedure from **3d** (51.5 mg, 0.1 mmol). The crude was purified by column chromatography on silica gel (hexane/EtOAc = 4:1). Further purification using GPC afforded **8d** (yellow oil, 0.7 mg, 1.4 μmol, 1%) and recovery of **3d** (3.7 mg, 7 μmol, 7%).

^{1}H NMR (600 MHz, CDCl$_3$): δ 8.01 (dd, J = 1.4, 7.8 Hz, 1H), 7.73 (ddd, J = 1.4, 7.8, 9.2 Hz, 1H), 7.65 (m, 2H), 7.60 (brd, J = 7.8 Hz, 1H), 7.28 (ddd, J = 1.4, 7.8, 8.7 Hz, 1H), 6.94 (ddd, J = 1.4, 7.8, 8.7 Hz, 1H), 6.82 (brd, J = 7.8 Hz, 1H), 4.00 (ddd, J = 9.6,10.6, 10.6 Hz, 1H), 3.96 (brdd, J = 5.5, 10.6 Hz, 1H), 3.92 (brdd, J = 9.6, 10.6 Hz, 1H), 3.89 (s, 3H), 3.82 (s, 3H), 2.68 (ddd, J = 3.7, 3.7, 14.7 Hz, 1H), 2.55 (ddd, J = 9.6, 9.6, 13.3 Hz, 1H), 2.25 (dddd, J = 3.7, 3.7, 5.5, 14.2 Hz, 1H), 2.12 (ddd, J = 3.7, 3.7, 14.7 Hz, 1H), 1.87 (brdd, J = 10.6, 13.3 Hz, 1H), 1.71 (dddd, J = 3.7, 3.7, 10.6, 14.2 Hz, 1H).

^{13}C NMR (150 MHz, CDCl$_3$): δ 176.1, 168.8, 168.6, 152.8, 148.3, 142.4, 134.0, 133.0, 131.9, 130.6, 128.7, 126.5, 124.1, 122.0, 121.2, 66.3, 62.7, 61.5, 53.7, 53.5, 46.3, 32.7, 30.7, 28.1.

IR (neat): 3800, 2820, 2250, 1625, 1550 cm^{-1}.

HRMS (ESI): Exact mass calculated for $C_{24}H_{23}N_3O_8SNa$ [M+Na]$^+$: 536.1098; found 536.1077.

Di-*tert*-butyl 12-tosyl-2,3-dihydro-1,4a-(epiminoethano)carbazole-4,4(1*H*)-dicarboxylate (8e)

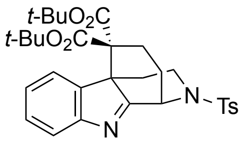

8e

Compound **8e** was prepared according to the general procedure from **3e** (56.8 mg, 0.1 mmol). The crude was purified by column chromatography on silica gel (hexane/EtOAc = 7:1) to afford **8e** (yellow oil, 10.7 mg, 22 µmol, 23%) with a recovery of **3e** (14.2 mg, 26 µmol, 26%).

^{1}H NMR (600 MHz, CDCl$_3$): δ 7.76 (d, *J* = 8.3 Hz, 2H), 7.52 (brd, *J* = 7.3 Hz, 1H), 7.35 (d, *J* = 8.3, 2H), 7.21 (ddd, *J* = 0.9, 7.3, 7.3 Hz, 1H), 6.74 (ddd, *J* = 0.9, 7.3, 7.3 Hz, 1H), 6.00 (brd, *J* = 7.3 Hz, 1H), 3.79 (dd, *J* = 9.2, 9.2 Hz, 1H), 3.70 (brdd, *J* = 5.5, 11.0 Hz, 1H), 3.51 (ddd, *J* = 6.9, 9.6, 11.0 Hz, 1H), 2.77 (ddd, *J* = 9.2, 11.5, 13.3 Hz, 1H), 2.55 (ddd, *J* = 3.2, 6.0, 13.3 Hz, 1H), 2.49 (s, 3H), 2.26 (dddd, *J* = 3.2, 9.2, 11.5, 14.2 Hz, 1H), 2.05 (ddd, *J* = 5.5, 9.6, 15.6 Hz, 1H), 1.78 (dddd, *J* = 6.0, 9.2, 9.2, 14.2 Hz, 1H), 1.70 (brdd, *J* = 6.9, 15.6 Hz, 1H), 1.55 (s, 9H), 1.50 (s, 9H).

^{13}C NMR (150 MHz, CDCl$_3$): δ 177.6, 167.9, 167.6, 153.3, 144.0, 143.2, 135.5, 130.1, 128.1, 127.6, 125.8, 121.7, 120.9, 83.2, 83.0, 65.9, 63.0, 62.8, 46.5, 33.0, 30.4, 29.3, 28.1, 28.0, 21.7.

IR (neat): 3150, 2800, 1775, 1570, 1200 cm^{-1}.

HRMS (ESI): Exact mass calculated for $C_{31}H_{38}N_2NaO_6S$ $[M+Na]^+$: 589.2343; found 589.2339.

4-(Hydroxy(methoxy)methylene)-12-tosyl-1,2-dihydro-1,4a-(epiminoethano)carbazol-3(4*H*)-one (8f)

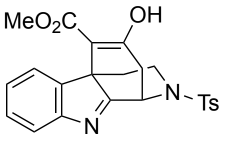

8f

Compound **8f** was prepared according to the general procedure from **3f** (44.0 mg, 0.1 mmol). The crude was purified by column chromatography on silica gel (hexane/EtOAc = 1:1 to 1:2) to afford **8f** (yellow oil, 9.0 mg, 21 μmol, 22%) with a recovery of **3f** (13.4 mg, 32 μmol, 32%).

^{1}H NMR (600 MHz, CDCl$_3$): δ 10.90 (brs, 1H, OH), 7.82 (d, *J* = 8.3 Hz, 2H), 7.40 (d, *J* = 8.3 Hz, 2H), 7.31 (brdd, *J* = 7.8, 7.8 Hz, 1H), 7.16 (brd, *J* = 7.8 Hz, 1H), 6.96 (brdd, *J* = 7.3, 7.8 Hz, 1H), 6.81 (brd, *J* = 7.3 Hz, 1H), 4.50 (dd, *J* = 7.3, 7.3 Hz, 1H), 3.90 (s, 3H), 3.70–3.63 (m, 2H), 3.13 (dd, *J* = 7.3, 18.8 Hz, 1H), 2.52 (m, 1H), 2.49 (s, 3H), 2.39 (brdd, *J* = 10.1, 12.4 Hz, 1H), 2.11 (brdd, *J* = 6.2, 12.4 Hz, 1H).

^{13}C NMR (150 MHz, CDCl$_3$): δ 189.5, 175.1, 167.9, 144.4, 141.2, 136.4, 134.5, 130.3, 129.5, 127.3, 124.6, 122.7, 112.4, 99.8, 57.8, 55.9, 52.5, 44.2, 42.3, 41.7, 21.8.

IR (neat): 3100, 2380, 2250, 1870, 1500 cm^{-1}.

HRMS (ESI): Exact mass calculated for $C_{23}H_{22}N_2NaO_5S$ [M+Na]$^+$: 461.1142; found 461.1137.

Dimethyl 6-methoxy-12-tosyl-2,3-dihydro-1,4a-(epiminoethano)carbazole-4,4(1*H*)-dicarboxylate (8g)

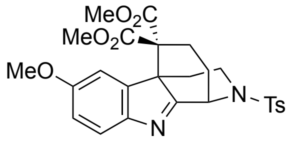

8g

Compound **8g** was prepared according to the general procedure from **3g** (52.1 mg, 0.1 mmol). The crude was purified by column chromatography on silica gel (hexane/EtOAc = 2:1 to 1:1) to afford **8g** (43.2 mg, 84.3 μmol, 83%) as yellow solid.

^{1}H NMR (600 MHz, CDCl$_3$): δ 7.77 (d, *J* = 8.3 Hz, 2H), 7.48 (d, *J* = 8.3 Hz, 1H), 7.35 (d, *J* = 8.3 Hz, 2H), 6.76 (dd, *J* = 2.1, 8.3 Hz, 1H), 5.86 (d, *J* = 2.1 Hz, 1H), 3.88 (s, 3H), 3.81 (m, 1H), 3.81 (s, 3H), 3.72 (brdd, *J* = 9.0, 9.0 Hz, 1H), 3.68 (s, 3H), 3.53 (ddd, *J* = 7.6, 11.0, 11.0 Hz, 1H), 2.66 (ddd, *J* = 2.8, 7.6, 14.5 Hz, 1H), 2.48 (m, 1H), 2.47 (s, 3H), 2.26 (m, 1H), 2.17 (m, 1H), 1.79 (m, 1H), 1.72 (brdd, *J* = 6.9, 13.1 Hz, 1H).

^{13}C NMR (150 MHz, CDCl$_3$): δ 174.4, 169.1, 168.9, 158.7, 146.6, 144.7, 144.3, 135.3, 130.1, 127.4, 122.0, 112.1, 108.8, 65.4, 62.6, 61.0, 55.7, 53.5, 53.4, 46.1, 32.9, 29.9, 28.8, 21.7.

IR (neat): 2950, 2350, 1730, 1460, 1160 cm^{-1}.

HRMS (ESI): Exact mass calculated for $C_{26}H_{28}N_2NaO_7S$ [M+Na]$^+$: 535.1509; found 535.1492.

Melting Point: 149.3–159.0 °C

Scale-up synthesis (akuammiline-type alkaloid skeleton)

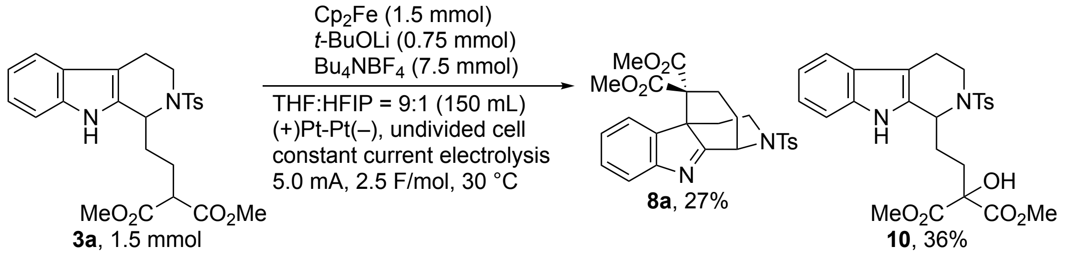

The anodic oxidation was carried out in a 200 mL round bottom flask equipped with a Pt anode (1 cm × 1 cm, 0.20 mm thickness) and a Pt cathode (1 cm × 1 cm, 0.20 mm thickness). The flask was charged with cyclization precursor **3a** (726.9 mg, 1.5 mmol), tetrabutylammonium tetrafluoroborate (2.47 g, 7.5 mmol), ferrocene (279.0 mg, 1.5 mmol), and lithium *tert*-butoxide (60.0 mg, 0.75 mmol) in THF/HFIP = 9:1 (150 mL). A constant current (5.0 mA, 2.5 F/mol,) was supplied at 30 °C with vigorous stirring. After the electrolysis, the reaction mixture was diluted with saturated aqueous NH_4Cl (100 mL) and extracted with EtOAc (3 × 100 mL). The combined organic layers were washed with brine, dried over Na_2SO_4 and concentrated *in vacuo*. The crude mixture was purified by column chromatography on silica gel (hexane/EtOAc = 4:1) to afford product **8a** (193 mg, 0.4 mmol, 27%) as yellow oil and compound **10** (272 mg, 0.5 mmol, 36%) as yellow oil.

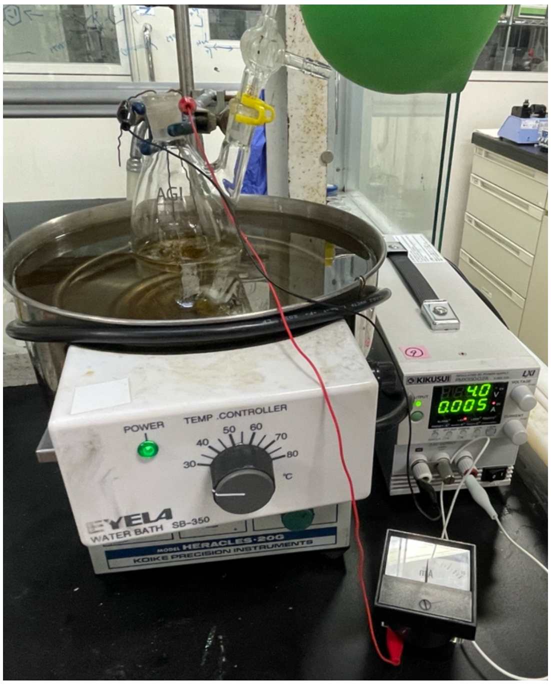

Figure S4. Scale-up synthesis (akuammiline-type alkaloid skeleton)

Miscellaneous optimization of the reaction conditions (akuammiline-type alkaloid skeleton)

Table S6. Screening of solvent

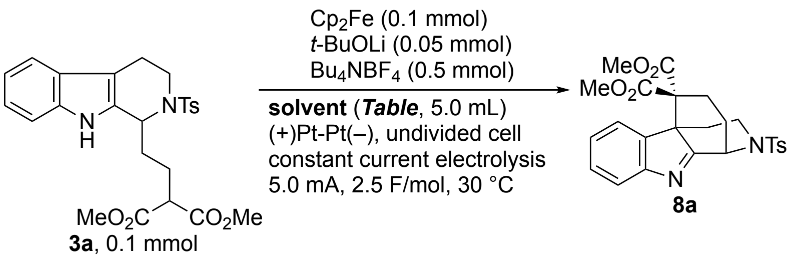

Entry	Solvent	Yield of **8a** (%)	Recovery of **3a** (%)
1	THF:HFIP = 9:1	94	Not Detected
2	THF:*t*-BuOH = 9:1	57	Not Detected
3[a]	THF:MeOH = 9:1	Trace	Trace
4	CH_3CN:HFIP = 9:1	<7	75
5	THF (5.0 mL), HFIP (50 μL)	Not Detected	46

[a] MeOLi was used instead of *t*-BuOLi.

Table S7. Amount of ferrocene and current value

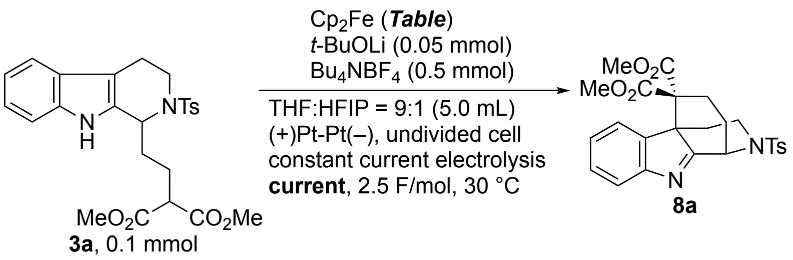

Entry	Cp_2Fe (mmol)	Current (mA)	Yield of **8a** (%)	Recovery of **3a** (%)
1	0.1	5.0	94	Not Detected
2	0.01	5.0	<7	<67
3	0.01	0.5	<25	<11
4	none	5.0	Not Detected	82

Table S8. Screening of base

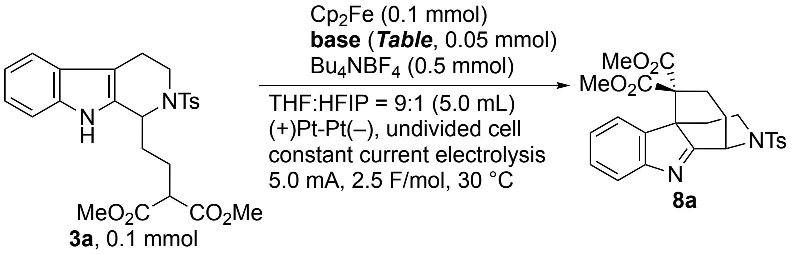

Entry	Base (0.05 mmol)	Yield of **8a** (%)	Recovery of **3a** (%)
1	*t*-BuOLi	94	Not Detected
2	*t*-BuONa	57	11
3	MeOLi	83	Not Detected
4	none	<29	28

Table S9. Screening of mediator

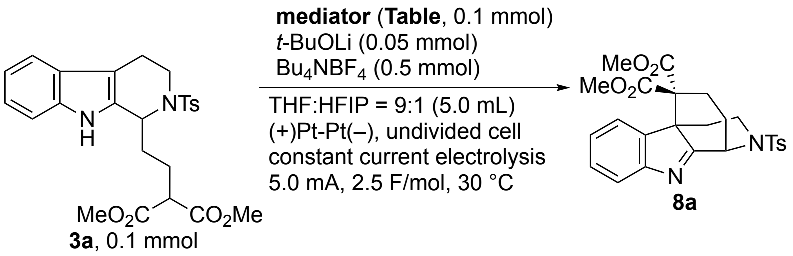

Entry	Mediator (0.1 mmol)	Oxidation potential ($E_{1/2}$ V vs. Fc/Fc^{+})	Yield of **8a** (%)	Recovery of **3a** (%)
1	ferrocene	0.00	94	Not Detected
2	1,1'-dimethylferrocene	–0.09	<37	Not Detected
3	acetylferrocene	+0.23	Not Detected	71%

Table S10. Co(salen) catalyst as a redox mediator

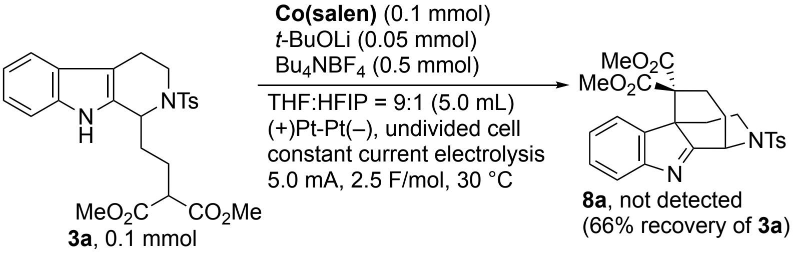

Table S11. Screening of anode material

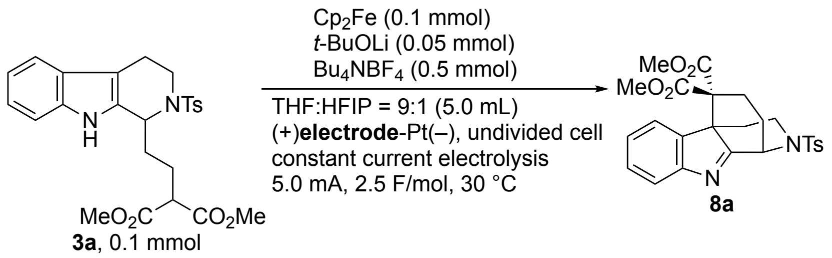

Entry	Electrode	Yield of **8a** (%)	Recovery of **3a** (%)
1	Pt	94	Not Detected
2	Graphite	57	Not Detected

Table S12. *t*-BuOH as co-solvent

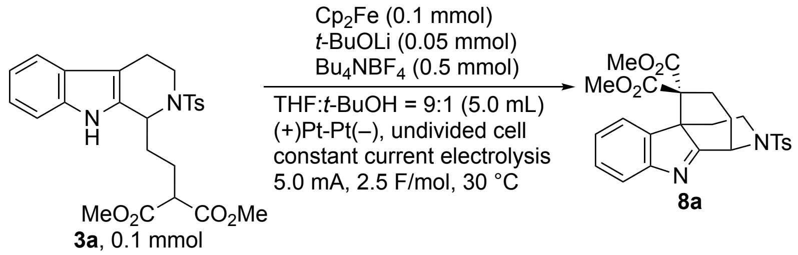

Entry	Deviation from standard condition	Yield of **8a** (%)	Recovery of **3a** (%)
1	none	57	Not Detected
2	2.5 mA instead of 5.0 mA	54	Not Detected
3	10.0 mA instead of 5.0 mA	Not Detected	79
4	2.0 F/mol instead of 2.5 F/mol	40	27
5	*t*-BuONa instead of *t*-BuOLi from entry 4	31	Not Detected

Electrolysis with THF/MeOH = 9:1

When MeOLi and MeOH was used instead of *t*-BuOLi and HFIP, the NMR analysis indicated the formation of cyclization product **8a** with several decomposed compounds, though the structures of these compounds could not be completely determined. One of the side products would be the methanol adduct into the imine moiety, and the HRMS analysis suggests the structure.

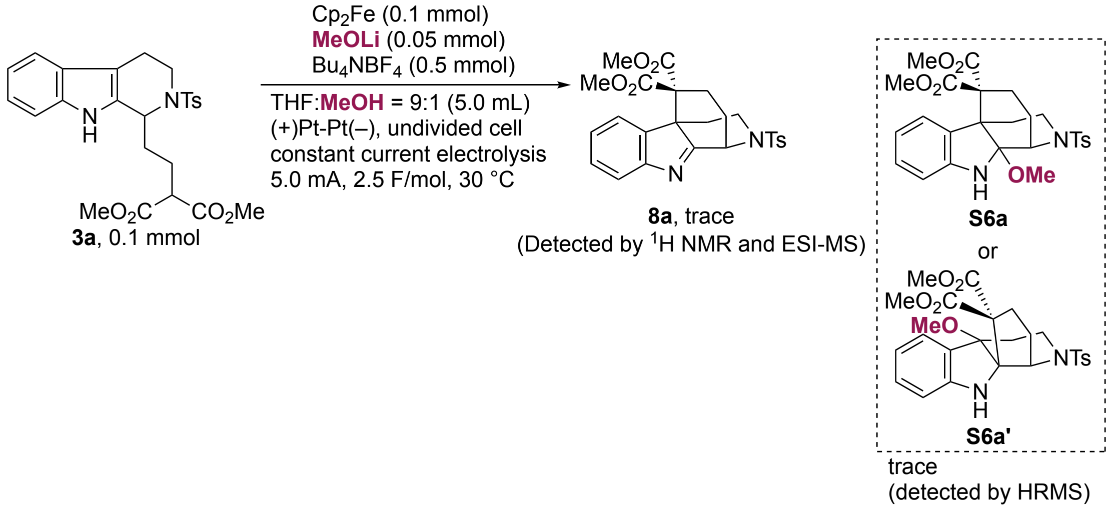

HRMS analysis

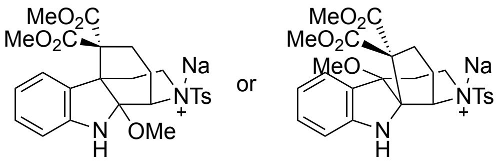

Exact mass calcd for $C_{26}H_{30}N_2NaO_7S$ [M+Na]$^+$: 537.1666; found 537.1673.

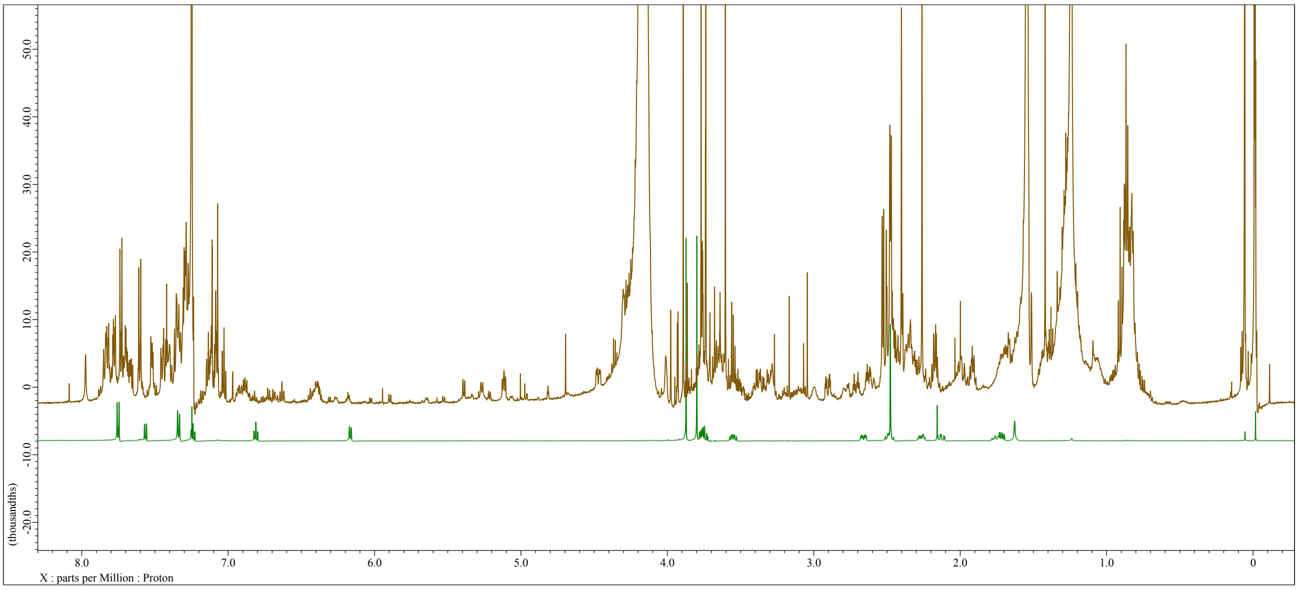

Figure S5. ^{1}H NMR of crude mixture (upper) and **8a** (lower)

5. Control experiments

Electrolysis in a divided cell

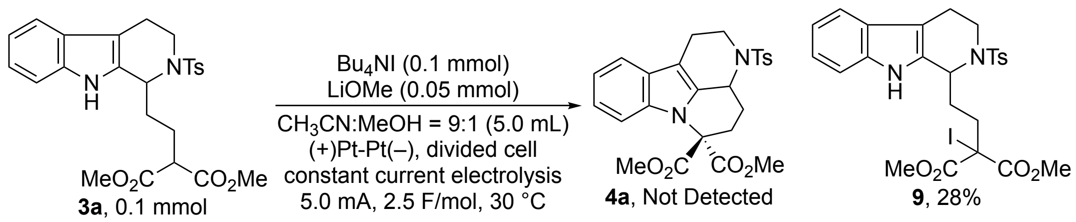

Cyclization precursor **3a** (48.5 mg, 0.1 mmol), tetrabutylammonium iodide (38.2 mg, 0.1 mmol), and lithium methoxide (1.9 mg, 0.05 mmol) in CH_3CN/MeOH = 9:1 (5.0 mL) were charged in a anodic chamber, and tetrabutylammonium iodide (38.2 mg, 0.1 mmol), and lithium methoxide (1.9 mg, 0.05 mmol) in CH_3CN/MeOH = 9:1 (5.0 mL) were charged in a cathodic chamber. A constant current (5.0 mA, 2.5 F/mol) was supplied at 30 °C with vigorous stirring. After the electrolysis, the reaction mixture was diluted with saturated aqueous NH_4Cl (10 mL) and extracted with EtOAc (3 × 10 mL). The combined organic layers were washed with brine, dried over Na_2SO_4 and concentrated *in vacuo*. The crude mixture was purified by column chromatography on silica gel (hexane/EtOAc = 4:1) to afford product **9**[3] (13 mg, 28 μmol, 28%) as yellow oil.

Although methoxide could not be transported from the cathodic to the anodic chamber, the addition of 0.05 mmol of LiOMe promoted deprotonation followed by the iodination, resulting in the formation of 28% of iodinated product **9**.

Dimethyl 2-iodo-2-(2-(2-tosyl-2,3,4,9- tetrahydro-1*H*-pyrido[3,4-*b*]indol-1-yl)ethyl)malonate (9)[3]

^{1}H NMR (600 MHz, $CDCl_3$): δ 7.85 (brs, 1H, NH), 7.63 (d, *J* = 8.3 Hz, 2H), 7.32 (d, *J* = 7.8 Hz, 1H), 7.30 (d, *J* = 7.8 Hz, 1H), 7.16 (dd, *J* = 7.8, 7.8 Hz, 1H), 7.10 (d, *J* = 8.3 Hz, 2H), 7.05 (dd, *J* = 7.8, 7.8 Hz, 1H), 5.15 (dd, *J* = 4.1, 9.2 Hz, 1H), 4.16 (dd, *J* = 5.5, 14.7 Hz, 1H), 3.81 (s, 3H), 3.81 (s, 3H), 3.37 (ddd, *J* = 4.6, 11.9, 14.7 Hz, 1H), 2.51 (dd, *J* = 4.6, 16.1 Hz, 1H), 2.45 (dd, *J* = 5.1, 10.1 Hz, 2H), 2.39 (m, 1H), 2.28 (s, 3H), 2.05–1.94 (m, 2H).
^{13}C NMR (150 MHz, $CDCl_3$): δ 168.9, 168.8, 143.6, 137.9, 136.0, 132.1, 129.7, 127.0, 126.7, 122.4, 119.8, 118.4, 111.1, 108.4, 54.2 (2C), 52.6, 42.3, 39.9, 36.9, 34.4, 21.6, 19.8.
IR (neat): 2400, 2300, 1800, 1540, 1300 cm^{-1}.

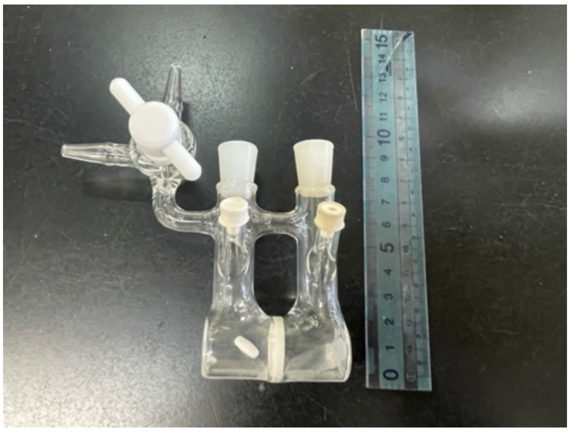

Figure S6. Divided cell. Two chambers were separated by 4G glass flit.

I_2 and Bu_4NI_3

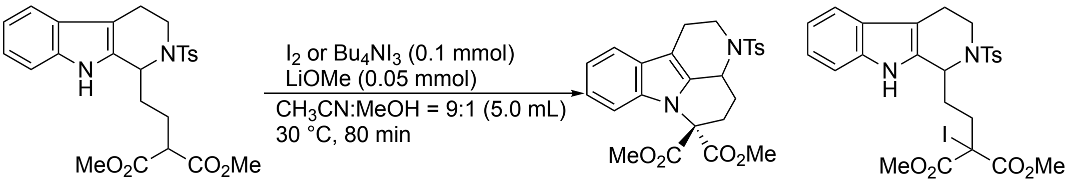

3a, 0.1 mmol **4a**, Not Detected **9**, 12% (I_2) or 39% (Bu_4NI_3)

To a solution of **3a** (48.5 mg, 0.1 mmol) in CH_3CN:MeOH = 9:1 (5.0 mL) was added lithium methoxide (1.9 mg, 0.05 mmol) and iodine (25.3 mg, 0.1 mmol) or tetrabutylammonium triiodide (62.3 mg, 0.1 mmol) at 30 °C, and stirred for 80 min. The resulting mixture was diluted with saturated aqueous NH_4Cl (10 mL) and extracted with EtOAc (3 × 10 mL). The combined organic layers were washed with brine, dried over $MgSO_4$ and concentrated *in vacuo*. The crude mixture was purified by column chromatography on silica gel (hexane/EtOAc = 4:1 to 0:1) to afford **9** (7.1 mg, 12 μmol, 12% (I_2 as an oxidant), or 24.0 mg, 39 μmol, 39% (Bu_4NI_3 as an oxidant)) as yellow oil.

Cyclization of 9 under basic conditions

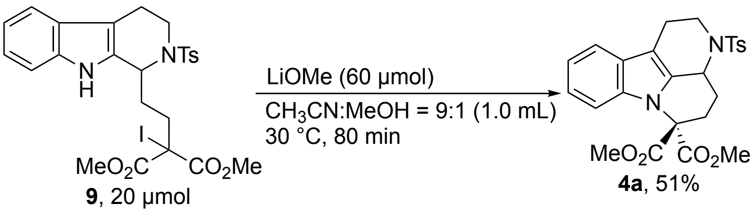

9, 20 μmol **4a**, 51%

To a solution of **9** (12.3 mg, 20 μmol) in CH_3CN:MeOH = 9:1 (1.0 mL) was added lithium methoxide (2.3 mg, 60 μmol) at 30 °C After stirring for 80 min, the resulting mixture was diluted with saturated aqueous NH_4Cl (10 mL) and extracted with EtOAc (3 × 10 mL). The combined organic layers were washed with brine, dried over $MgSO_4$ and concentrated *in vacuo*. The crude mixture was purified by column chromatography on silica gel (hexane/EtOAc = 4:1 to 0:1) to afford **4a** (4.9 mg, 10.2 μmol, 51%) as yellow oil.

Electrochemical cyclization of 9

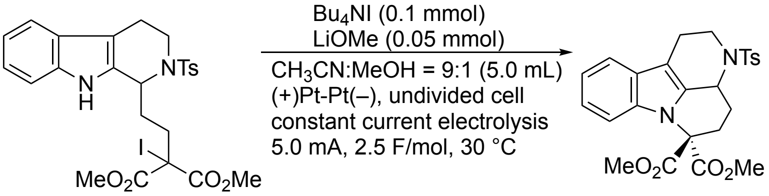

9, 0.1 mmol **4a**, 45%

The undivided cell was charged with iodide **9** (61.1 mg, 0.1 mmol), tetrabutylammonium iodide (37.0 mg, 0.1 mmol), and lithium methoxide (2.0 mg, 0.05 mmol) in CH_3CN/MeOH = 9:1 (5.0 mL). A constant current (5.0 mA, 2.5 F/mol) was supplied at 30 °C with vigorous stirring using platinum anode and cathode. After the electrolysis, the reaction mixture was diluted with saturated aqueous NH_4Cl (10 mL) and extracted with EtOAc (3 × 10 mL). The combined organic layers were washed with brine, dried over Na_2SO_4 and concentrated *in vacuo*. The crude mixture was purified by column chromatography on silica gel (hexane/EtOAc = 4:1) to afford product **4a** (21.5 mg, 44.6 μmol, 45%) as yellow oil, and iodide **9** was recovered in 11% (6.8 mg, 11.4 μmol) yield.

6. Cyclic voltammetry

A Pt electrode (surface area: 0.071 cm^2, BAS), Pt coil electrode, and an Ag/Ag^+ electrode (Ag wire in 0.01 M $AgNO_3$/0.10 M Bu_4NPF_6/CH_3CN or THF) were used as working, counter, and reference electrodes, respectively. The working electrode was polished with 5 μm diamond and 0.5 μm alumina slurry. After polishing, it was washed with deionized water and acetone and dried in an oven. CH_3CN or THF solutions of each sample (5 mM) and Bu_4NPF_6 (0.1 M) were prepared as an electrochemical solution and bubbled with argon for 2 min. Using the electrodes and the solutions, beaker-typed electrochemical cells were constructed, and were connected with a potentiostat to perform cyclic voltammetry. The redox potentials were calibrated with ferrocene as a standard. Cyclic voltammetry was performed at a scan rate of 30–100 mV/s. The cyclic voltammograms were plotted in IUPAC convention.

Construction of mavacurane-type skeleton using Bu_4NI

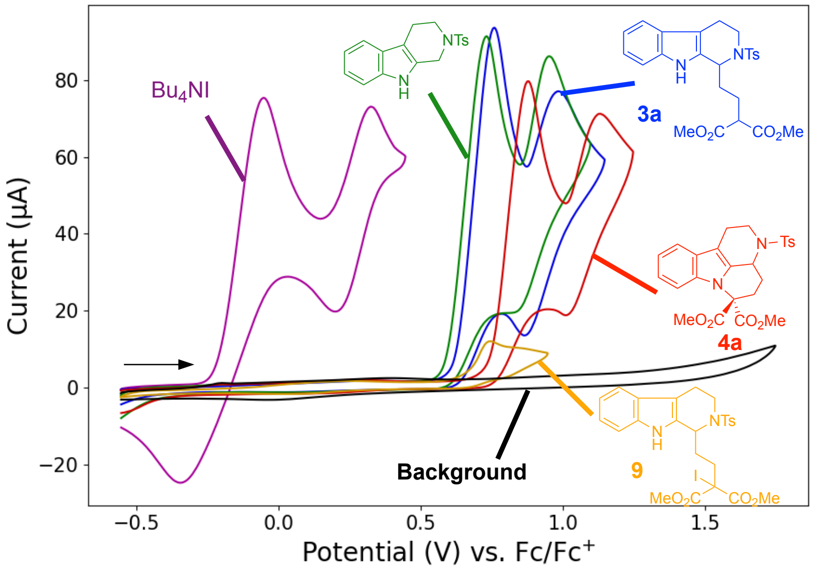

Figure S7. Cyclic voltammograms of Bu_4NI, *N*-Ts-1,2,3,4-tetrahydro-β-carboline, **3a**, **4a,** and **9**

solvent: CH_3CN, supporting electrolyte: Bu_4NPF_6 (0.1 M), substrate concentration: 5 mM (**9**: 0.7 mM),

working electrode: Pt, counter electrode: Pt coil, reference electrode: Ag/Ag^+, reference: Fc/Fc^+,

scan rate: 100 mV/s, temperature: room temperature

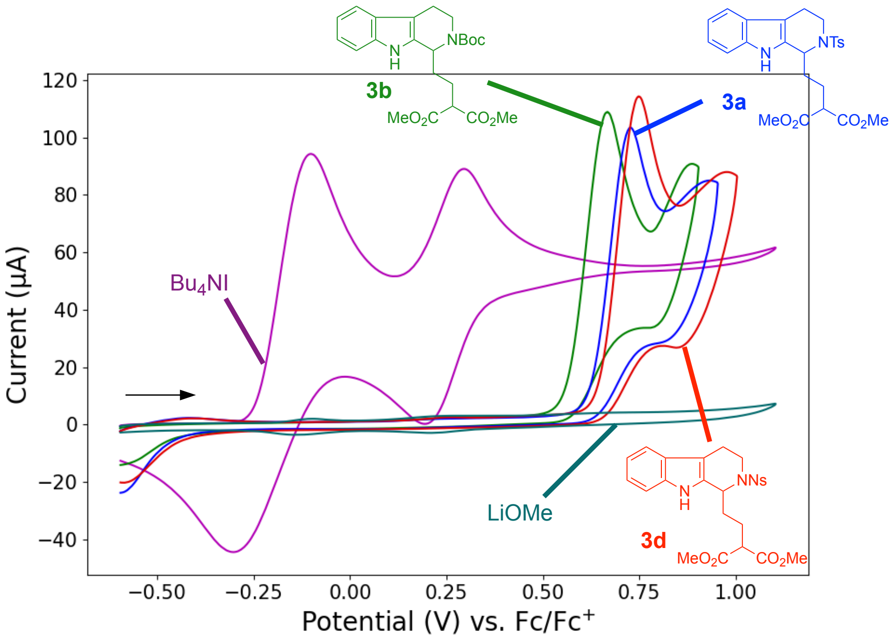

Figure S8. Cyclic voltammograms of Bu_4NI, LiOMe, **3a**, **3b**, and **3d**

solvent: CH_3CN, supporting electrolyte: Bu_4NPF_6 (0.1 M), substrate concentration: 5 mM, working electrode: Pt, counter electrode: Pt coil, reference electrode: Ag/Ag^+, reference: Fc/Fc^+, scan rate: 100 mV/s, temperature: room temperature

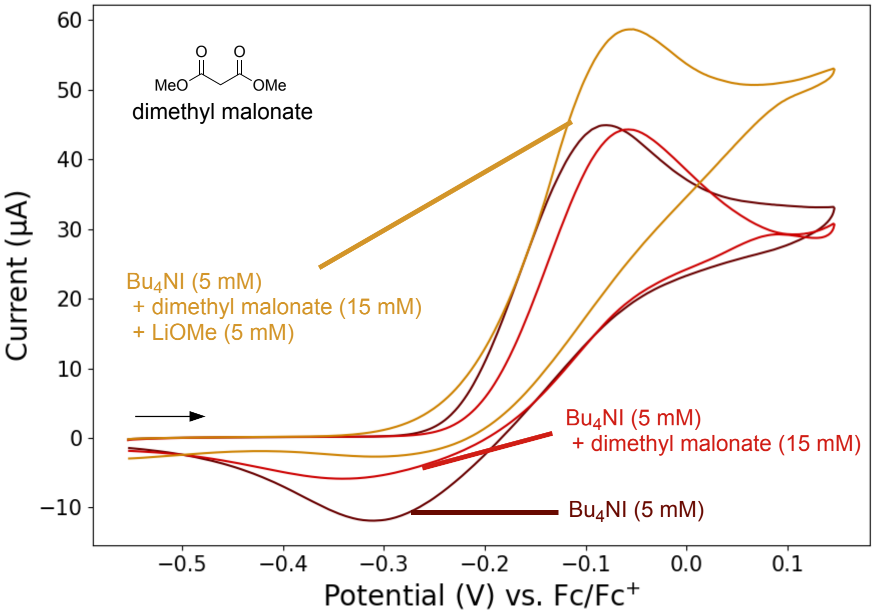

Figure S9. Catalytic current with dimethyl malonate at 30 mV/s

solvent: CH_3CN, supporting electrolyte: Bu_4NPF_6 (0.1 M), substrate concentration: 5–15 mM, working electrode: Pt, counter electrode: Pt coil, reference electrode: Ag/Ag^+, reference: Fc/Fc^+, scan rate: 30 mV/s, temperature: room temperature

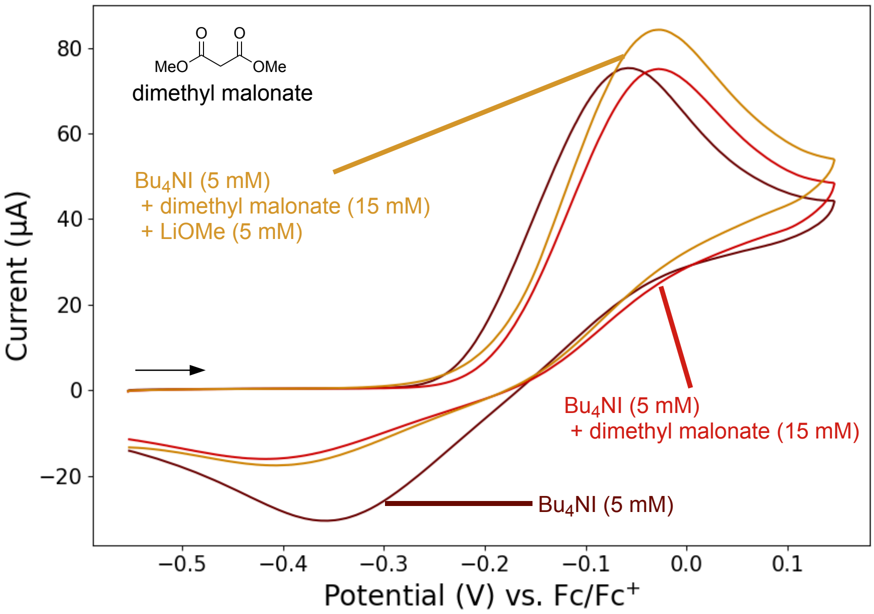

Figure S10. Catalytic current with dimethyl malonate at 100 mV/s

solvent: CH_3CN, supporting electrolyte: Bu_4NPF_6 (0.1 M), substrate concentration: 5–15 mM, working electrode: Pt, counter electrode: Pt coil, reference electrode: Ag/Ag^+, reference: Fc/Fc^+, scan rate: 100 mV/s, temperature: room temperature

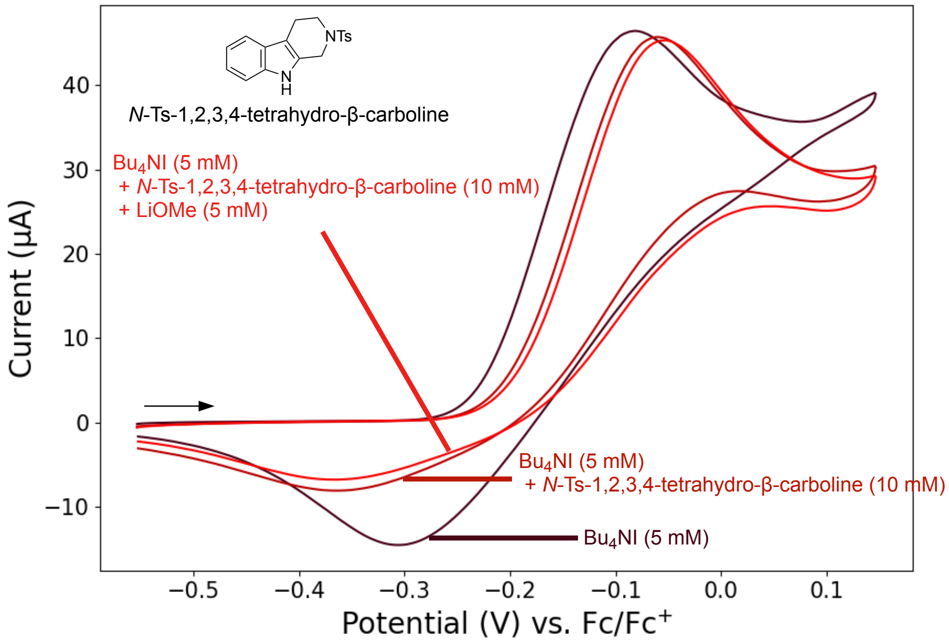

Figure S11. Catalytic current with *N*-Ts-1,2,3,4-tetrahydro-β-carboline at 30 mV/s

solvent: CH_3CN, supporting electrolyte: Bu_4NPF_6 (0.1 M), substrate concentration: 5–10 mM, working electrode: Pt, counter electrode: Pt coil, reference electrode: Ag/Ag^+, reference: Fc/Fc^+, scan rate: 30 mV/s, temperature: room temperature

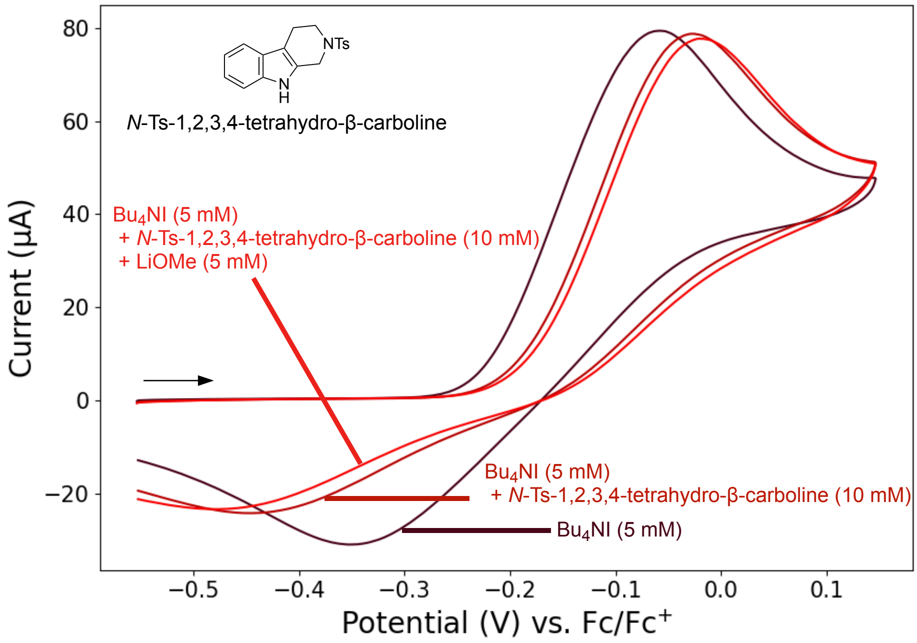

Figure S12. Catalytic current with *N*-Ts-1,2,3,4-tetrahydro-β-carboline at 100 mV/s

solvent: CH_3CN, supporting electrolyte: Bu_4NPF_6 (0.1 M), substrate concentration: 5–10 mM, working electrode: Pt, counter electrode: Pt coil, reference electrode: Ag/Ag^+, reference: Fc/Fc^+, scan rate: 100 mV/s, temperature: room temperature

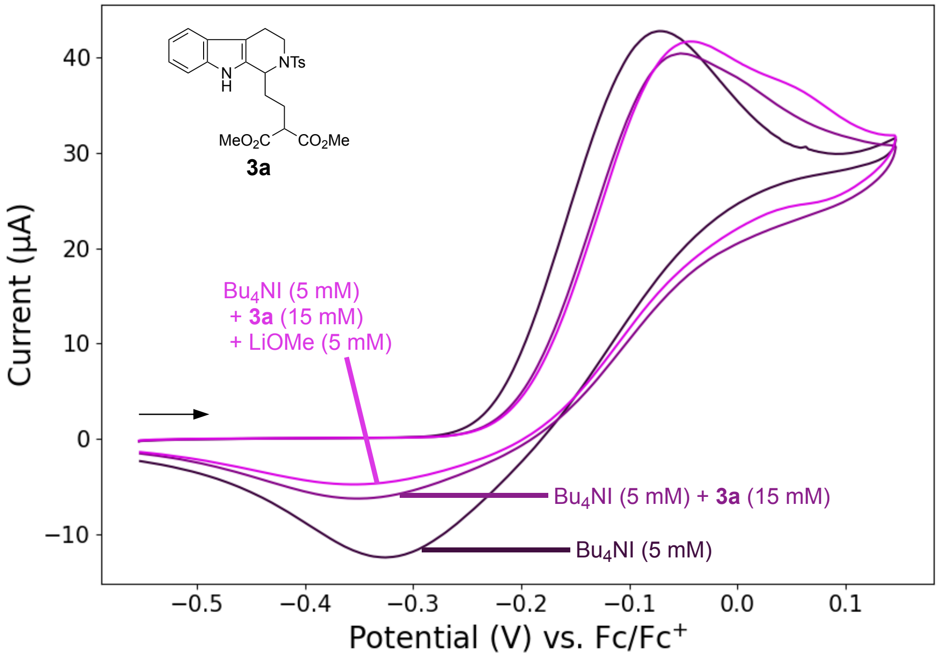

Figure S13. Catalytic current with **3a** at 30 mV/s

solvent: CH_3CN, supporting electrolyte: Bu_4NPF_6 (0.1 M), substrate concentration: 5–15 mM, working electrode: Pt, counter electrode: Pt coil, reference electrode: Ag/Ag^+, reference: Fc/Fc^+, scan rate: 30 mV/s, temperature: room temperature

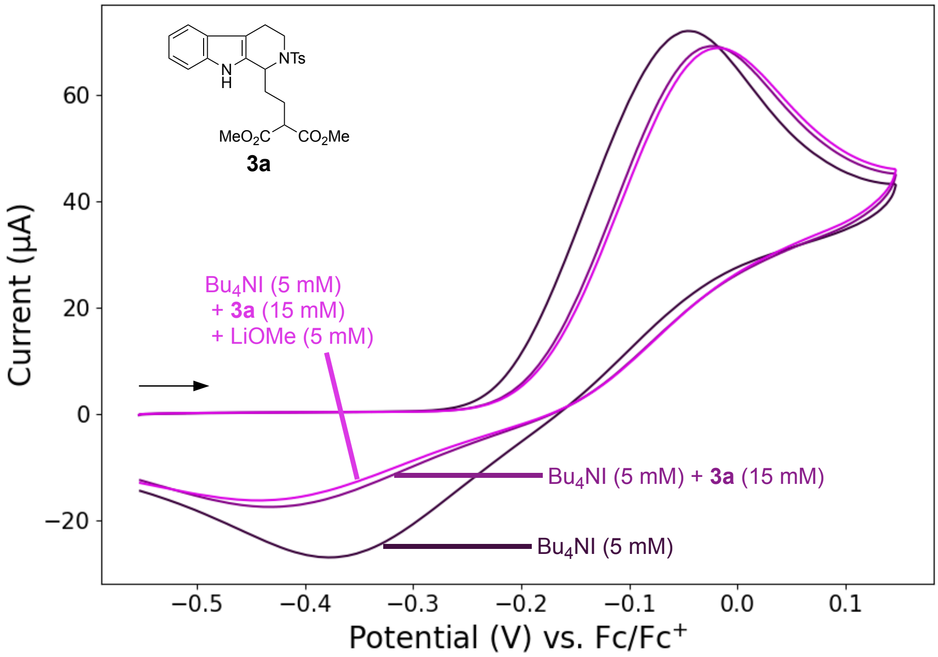

Figure S14. Catalytic current with **3a** at 100 mV/s

solvent: CH_3CN, supporting electrolyte: Bu_4NPF_6 (0.1 M), substrate concentration: 5–15 mM, working electrode: Pt, counter electrode: Pt coil, reference electrode: Ag/Ag^+, reference: Fc/Fc^+, scan rate: 100 mV/s, temperature: room temperature

Construction of akuammiline-type skeleton using ferrocene

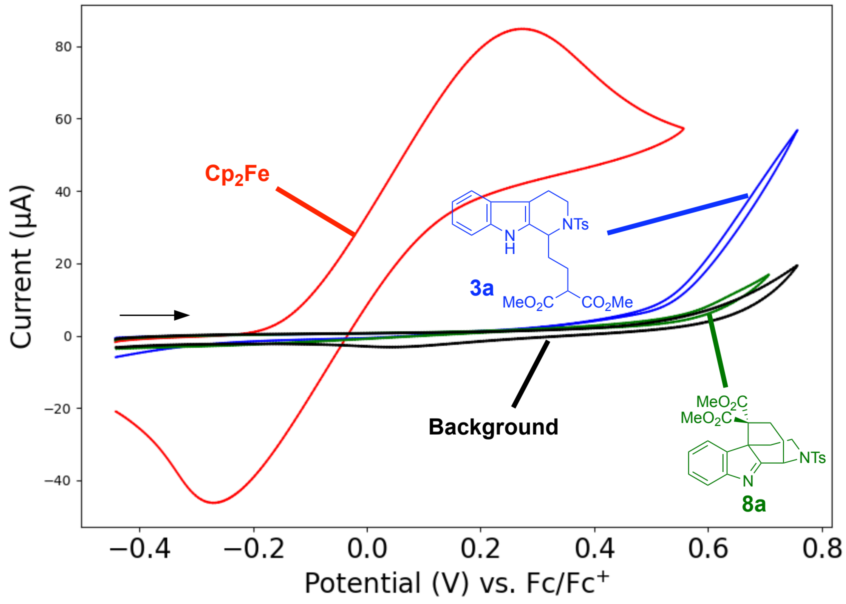

Figure S15. Cyclic voltammograms of Cp_2Fe, **3a** and **8a**

solvent: THF, supporting electrolyte: Bu_4NPF_6 (0.1 M), substrate concentration: 5 mM, working electrode: Pt, counter electrode: Pt coil, reference electrode: Ag/Ag^+, reference: Fc/Fc^+, scan rate: 100 mV/s, temperature: room temperature

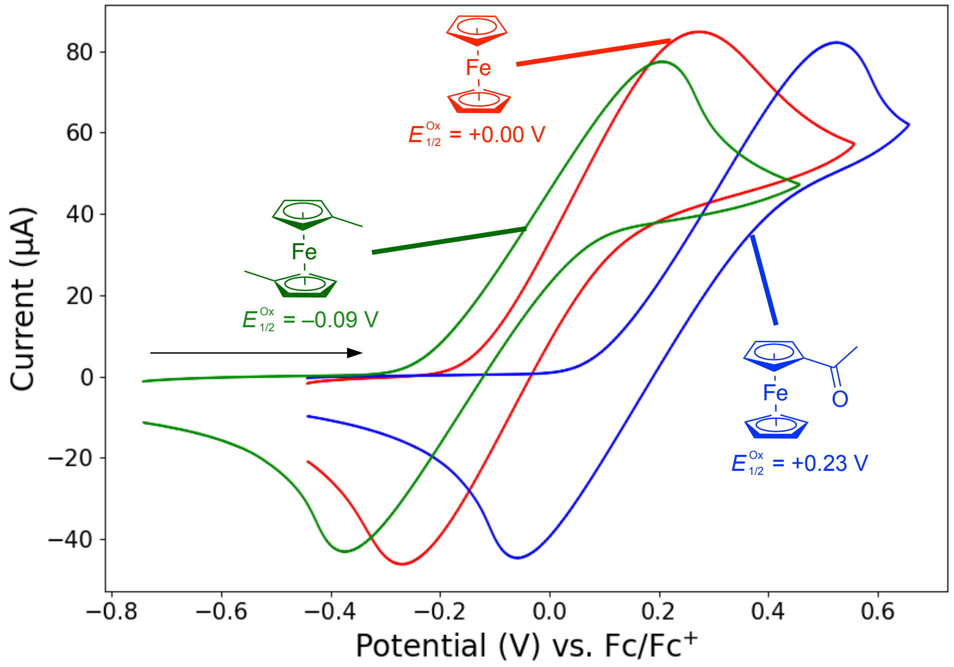

Figure S16. Cyclic voltammograms of ferrocene derivatives

solvent: THF, supporting electrolyte: Bu_4NPF_6 (0.1 M), substrate concentration: 5 mM, working electrode: Pt, counter electrode: Pt coil, reference electrode: Ag/Ag^+, reference: Fc/Fc^+, scan rate: 100 mV/s, temperature: room temperature

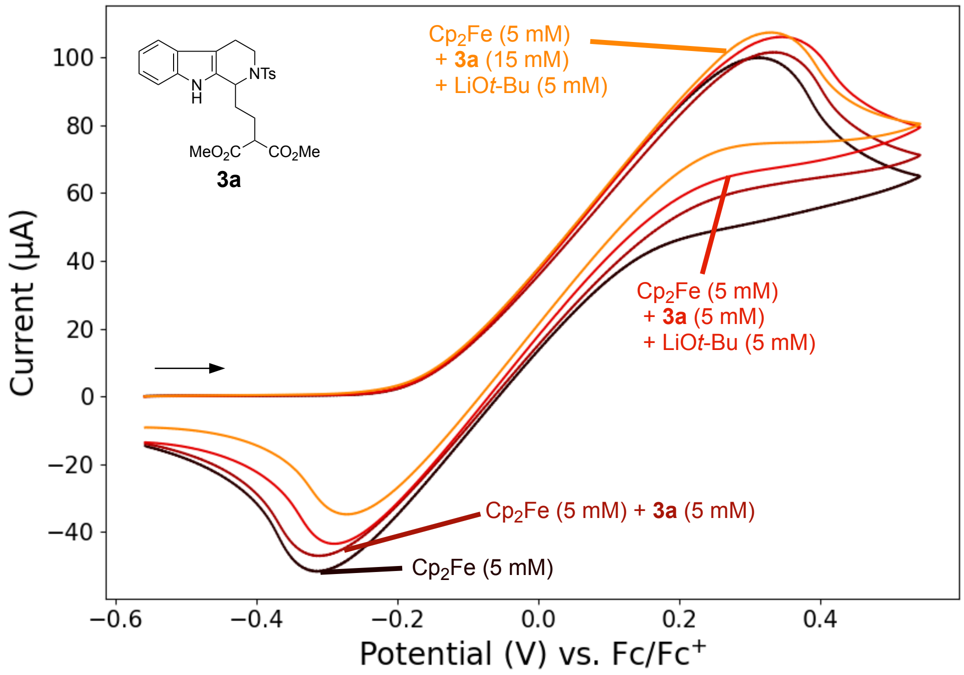

Figure S17. Catalytic current with **3a**

solvent: THF, supporting electrolyte: Bu_4NPF_6 (0.1 M), substrate concentration: 5–15 mM,

working electrode: Pt, counter electrode: Pt coil, reference electrode: Ag/Ag^+, reference: Fc/Fc^+,

scan rate: 100 mV/s, temperature: room temperature

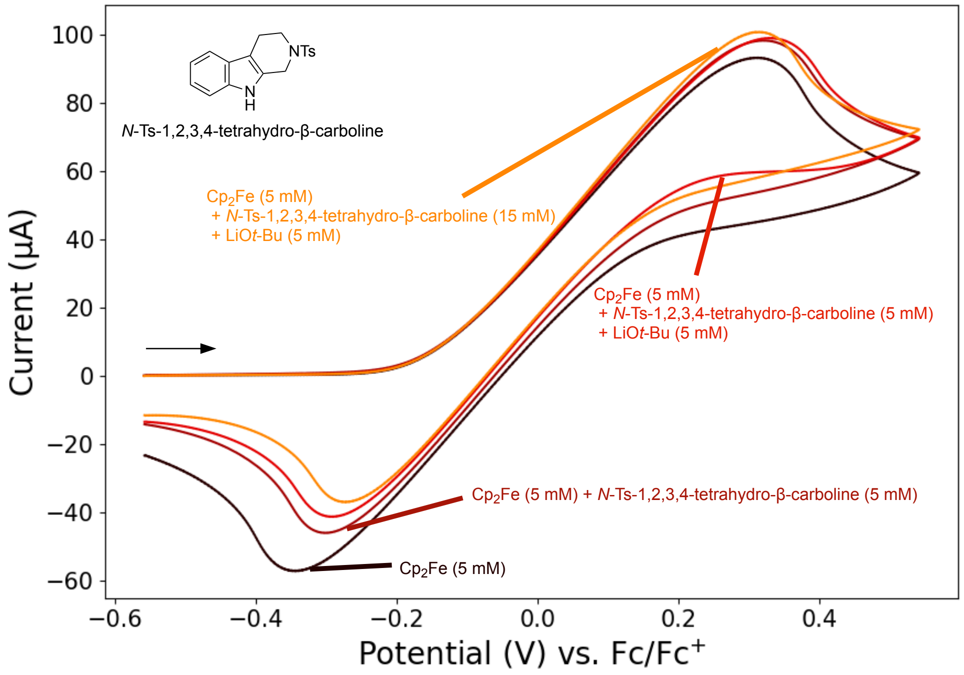

Figure S18. Catalytic current with *N*-Ts-1,2,3,4-tetrahydro-β-carboline

solvent: THF, supporting electrolyte: Bu_4NPF_6 (0.1 M), substrate concentration: 5–15 mM,

working electrode: Pt, counter electrode: Pt coil, reference electrode: Ag/Ag^+, reference: Fc/Fc^+,

scan rate: 100 mV/s, temperature: room temperature

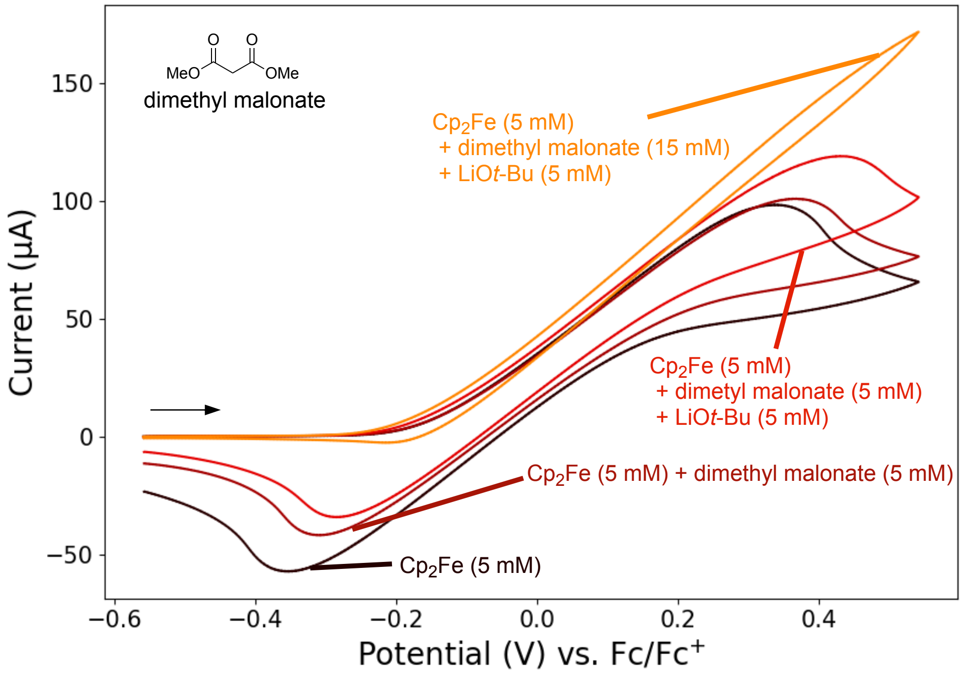

Figure S19. Catalytic current with dimethyl malonate

solvent: THF, supporting electrolyte: Bu_4NPF_6 (0.1 M), substrate concentration: 5–15 mM, working electrode: Pt, counter electrode: Pt coil, reference electrode: Ag/Ag^+, reference: Fc/Fc^+, scan rate: 100 mV/s, temperature: room temperature

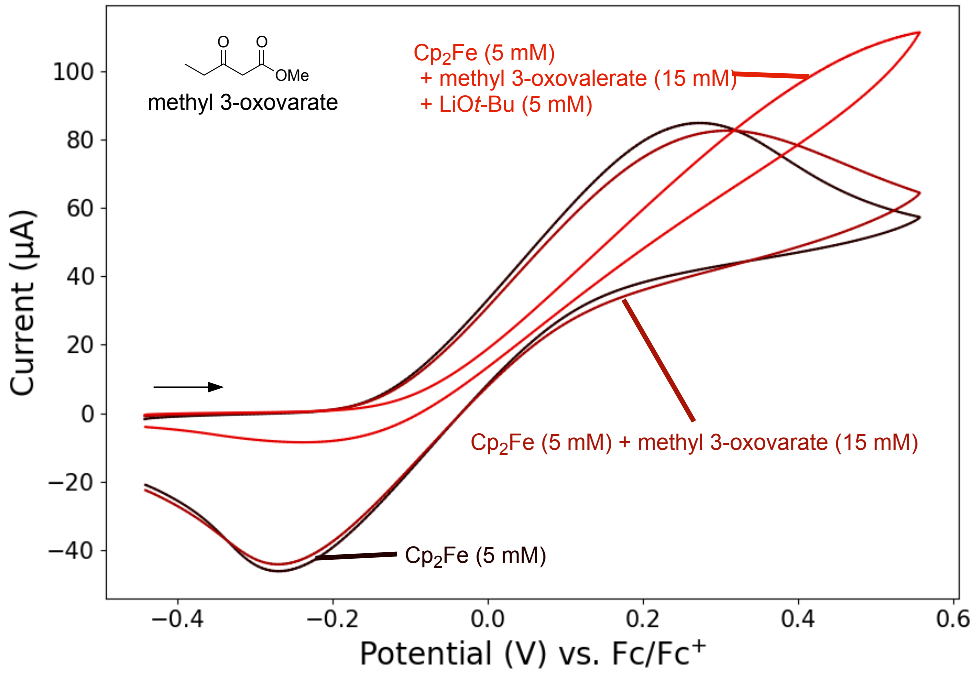

Figure S20. Catalytic current with methyl 3-oxovarate

solvent: THF, supporting electrolyte: Bu_4NPF_6 (0.1 M), substrate concentration: 5–15 mM, working electrode: Pt, counter electrode: Pt coil, reference electrode: Ag/Ag^+, reference: Fc/Fc^+, scan rate: 100 mV/s, temperature: room temperature

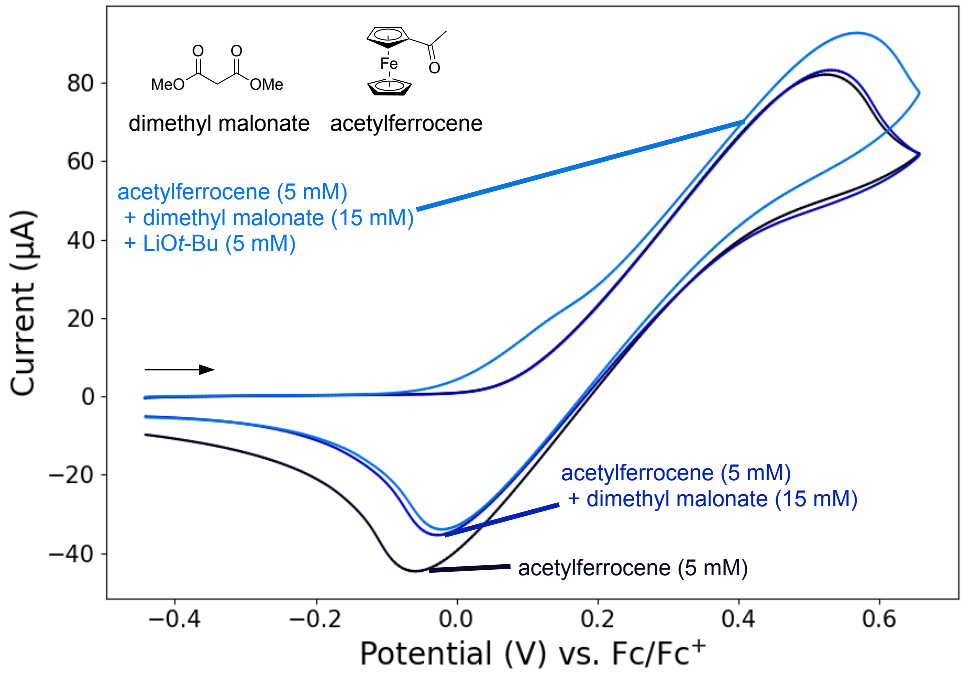

Figure S21. Catalytic current of acetylferrocene with dimethyl malonate

solvent: THF, supporting electrolyte: Bu_4NPF_6 (0.1 M), substrate concentration: 5–15 mM,

working electrode: Pt, counter electrode: Pt coil, reference electrode: Ag/Ag^+, reference: Fc/Fc^+,

scan rate: 100 mV/s, temperature: room temperature

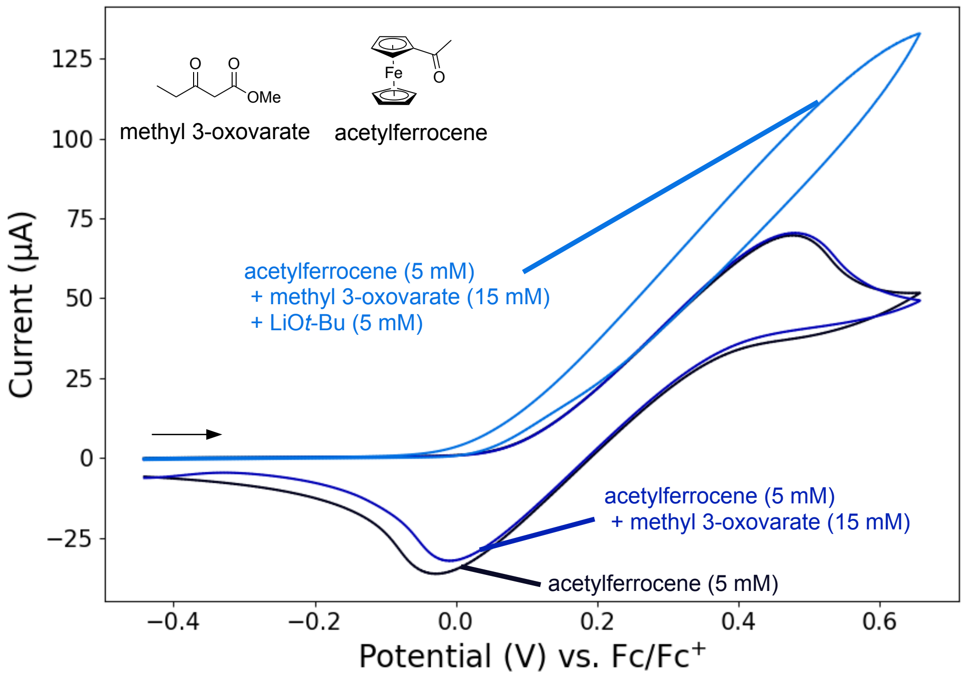

Figure S22. Catalytic current of acetylferrocene with methyl 3-oxovarate

solvent: THF, supporting electrolyte: Bu_4NPF_6 (0.1 M), substrate concentration: 5–15 mM,

working electrode: Pt, counter electrode: Pt coil, reference electrode: Ag/Ag^+, reference: Fc/Fc^+,

scan rate: 100 mV/s, temperature: room temperature

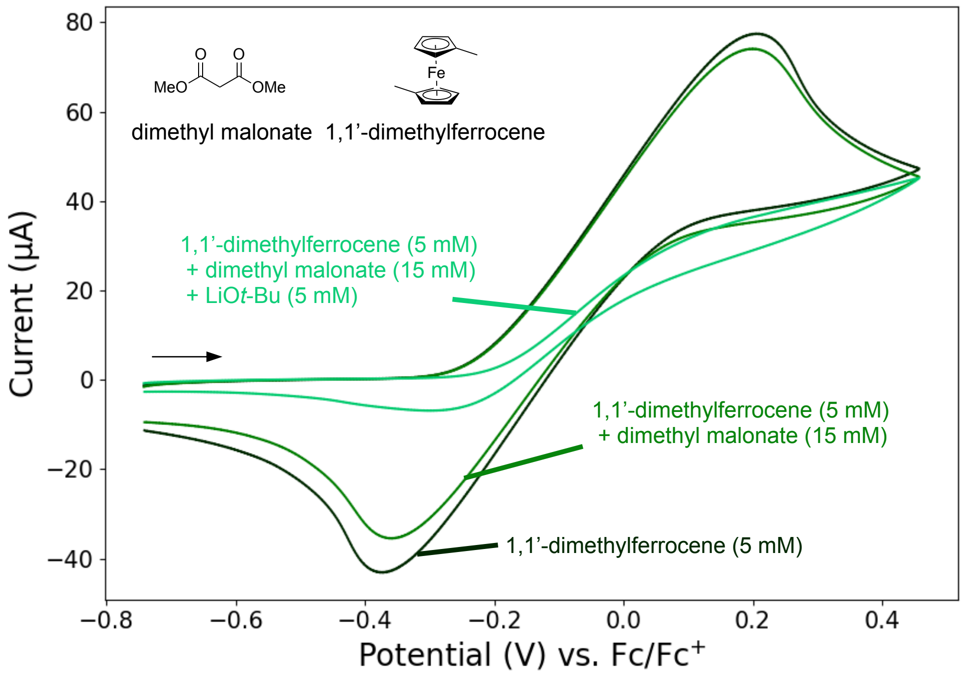

Figure S23. Catalytic current of 1,1'-dimethylferrocene with dimethyl malonate

solvent: THF, supporting electrolyte: Bu_4NPF_6 (0.1 M), substrate concentration: 5–15 mM, working electrode: Pt, counter electrode: Pt coil, reference electrode: Ag/Ag^+, reference: Fc/Fc^+, scan rate: 100 mV/s, temperature: room temperature

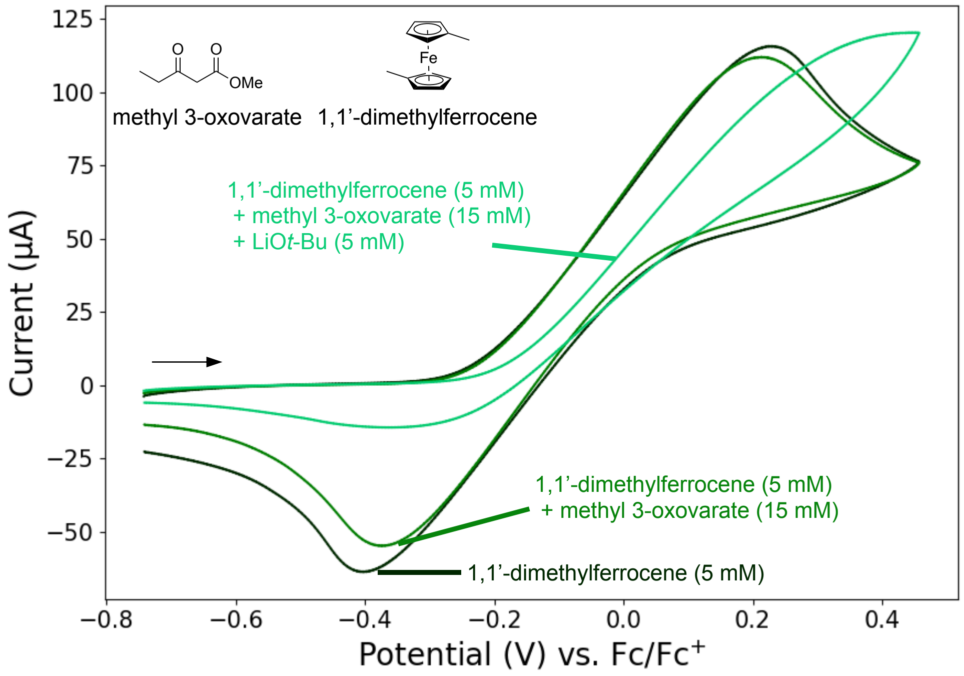

Figure S24. Catalytic current of 1,1'-dimethylferrocene with methyl 3-oxovarate

solvent: THF, supporting electrolyte: Bu_4NPF_6 (0.1 M), substrate concentration: 5–15 mM, working electrode: Pt, counter electrode: Pt coil, reference electrode: Ag/Ag^+, reference: Fc/Fc^+, scan rate: 100 mV/s, temperature: room temperature

7. DFT calculations

Density functional theory (DFT) calculations were performed using Gaussian 16 program[5]. Geometries were optimized at the (U)B3LYP functional[6,7] with 6-31+G(d,p) basis set for C, H, N, O, S, F and the Lanl2dz effective core potential (ECP)[8–10] for Fe, I with SMD solvation model[11] in THF (**Scheme S1**) or CH_3CN (**Scheme S2**). Dispersion interactions were included using Grimme's DFT-D3 correction with Becke-Johnson damping.[12] Thermochemical corrections were obtained from frequency calculations at the same level of theory. The connectivity of the transition states and their adjacent minima was confirmed by intrinsic reaction coordinate (IRC) calculations. The energy profile was illustrated in **Scheme S1** and **Scheme S2**. Calculated structures are illustrated using ChemDraw and CYLView.[13]

Underneath the Cartesian coordinates for the optimized geometries are listed the following energies:

Electronic energy (E)

Enthalpy at 298.15 K (H)

Gibbs free energy at 298.15 K and 1 mol/L (G)

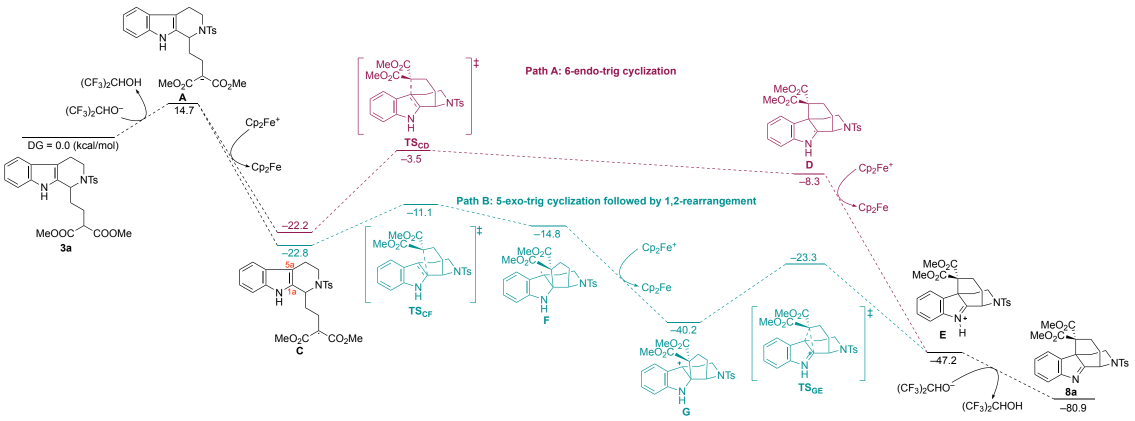

Scheme S1. DFT calculations for akuammiline skeleton ((U)B3LYP-D3/6-31G(d,p)$_{SMD(THF)}$)

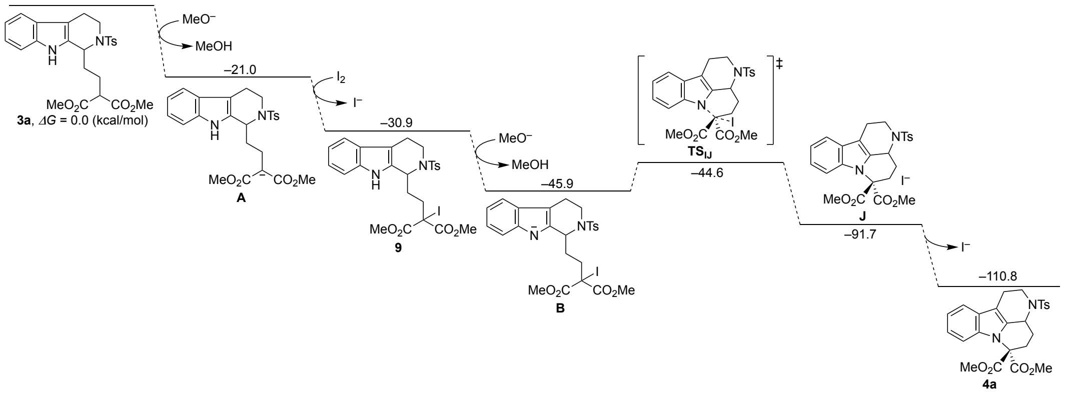

Scheme S2. DFT calculations for mavacurane skeleton (B3LYP-D3/6-31G(d,p)$_{SMD(CH3CN)}$)

Akuammiline skeleton (Scheme S1)

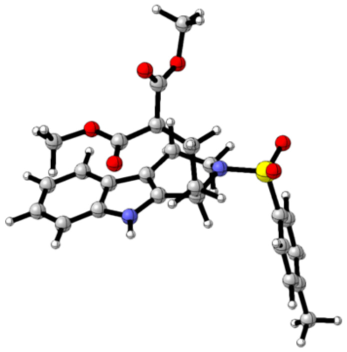

3a

C	1.7578200	2.4279220	-1.0214740
C	2.5641370	3.0959760	-1.9608080
C	3.4319110	4.0919150	-1.5164770
C	3.5076750	4.4371050	-0.1481570
C	2.7210030	3.7912580	0.8052900
C	1.8566580	2.7877880	0.3555780
H	2.5130790	2.8347970	-3.0144900
H	4.0621320	4.6140910	-2.2312160
H	4.1935250	5.2181850	0.1679110
H	2.7823770	4.0491930	1.8588760
C	0.7848440	1.3728670	-1.1008430
C	0.3497220	1.1238420	0.1760680
C	0.2313210	0.6037200	-2.2547600
H	0.1235200	1.2364860	-3.1433620
H	0.8797420	-0.2384650	-2.5343900
C	-1.1526100	0.0870680	-1.8613540
H	-1.8413020	0.9370620	-1.7509740
H	-1.5378070	-0.5805540	-2.6299480
C	-0.6382660	0.0796090	0.6274220
H	-1.4994030	0.6102990	1.0659700
N	-1.0855470	-0.6899870	-0.5855710
C	-3.8307110	-0.5507000	-0.0210020
C	-4.1469370	-0.2337580	1.3029080
C	-4.5248070	0.0297200	-1.0885230
C	-5.1741370	0.6773320	1.5545410
H	-3.6099400	-0.7011990	2.1211660
C	-5.5466160	0.9373910	-0.8163640
H	-4.2776160	-0.2336310	-2.1110650
C	-5.8880510	1.2753410	0.5046590
H	-5.4262260	0.9221280	2.5827460
H	-6.0916450	1.3875130	-1.6418980
C	-7.0172650	2.2357320	0.7796360
H	-6.9956910	3.0825270	0.0855830
H	-7.9856410	1.7355440	0.6517660
H	-6.9746840	2.6229430	1.8017480
O	-2.7221530	-2.3249630	-1.6514720
C	-0.0408160	-0.8252600	1.7402090
H	0.4840630	-0.1755380	2.4460620
H	-0.8614620	-1.2844000	2.2923710
C	0.8914670	-1.9585710	1.2910410
H	1.2302590	-2.4882100	2.1874470
H	0.3208890	-2.6676370	0.6873520
C	3.0748250	-0.6168290	1.2396650
C	2.8949000	-2.7821470	0.0503930
O	3.9264580	-0.0158550	0.3943500
O	2.4450050	-3.2440860	-1.1263850
O	3.0759850	-0.4461630	2.4437090
O	3.7697850	-3.3214190	0.7034240
C	3.0293370	-4.4824180	-1.5985710
H	4.1052190	-4.3614070	-1.7480940
H	2.8430650	-5.2869880	-0.8826450
H	2.5328250	-4.6925090	-2.5458480
C	4.9114430	0.8650260	0.9814810
H	4.4264040	1.6218240	1.5998620
H	5.6179420	0.2870150	1.5838240

H	5.4197470	1.3338930	0.1394720
S	-2.4680760	-1.6653700	-0.3604750
O	-2.2481020	-2.4883250	0.8375860
N	0.9915490	1.9746640	1.0603200
H	0.8561720	2.0013850	2.0610290
C	2.1302660	-1.5345010	0.4742570
H	1.8191530	-1.0172510	-0.4334870

Imaginary frequency = 0

E = -1928.205245

H = -1928.204300

G = -1928.303939

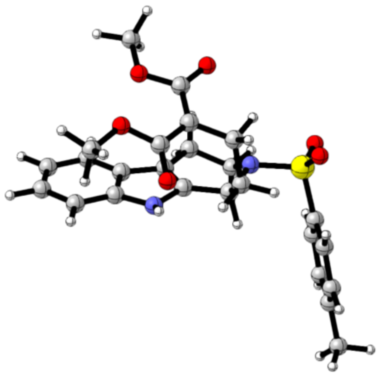

A

C	2.0428000	1.9897760	-1.0975420
C	2.9391350	2.6215220	-1.9777350
C	4.0294370	3.3128380	-1.4488340
C	4.2386180	3.3861890	-0.0541670
C	3.3556050	2.7798570	0.8415780
C	2.2634520	2.0903280	0.3085140
H	2.7911150	2.5603690	-3.0529570
H	4.7346350	3.7984790	-2.1183560
H	5.1016660	3.9237820	0.3294330
H	3.5121330	2.8363680	1.9148930
C	0.8631880	1.1836190	-1.2662820
C	0.4358760	0.8281240	-0.0136860
C	0.1244820	0.7399370	-2.4862840
H	0.0667980	1.5387530	-3.2364530
H	0.6134600	-0.1190490	-2.9644090
C	-1.3008170	0.3640090	-2.0779970
H	-1.8494950	1.2701540	-1.7782120
H	-1.8199320	-0.0996480	-2.9139220
C	-0.7609350	-0.0157490	0.3396440
H	-1.5351160	0.6640540	0.7375980
N	-1.2797770	-0.6100280	-0.9450300
C	-3.9465370	-0.3937630	-0.1206800
C	-4.1756720	-0.4048660	1.2574060
C	-4.6055370	0.5185500	-0.9518290
C	-5.0787760	0.5082680	1.8037690
H	-3.6631950	-1.1200640	1.8909420
C	-5.5029300	1.4245450	-0.3876990
H	-4.4277160	0.5129300	-2.0216340
C	-5.7546940	1.4347020	0.9945980
H	-5.2600710	0.4984180	2.8752530
H	-6.0182140	2.1327620	-1.0313590
C	-6.7527850	2.3977410	1.5869570
H	-6.7482290	3.3542080	1.0544760
H	-7.7690650	1.9889280	1.5166750
H	-6.5464840	2.5881140	2.6445300
O	-3.1394210	-1.8076310	-2.2129450
C	-0.4364600	-1.0271430	1.4582910
H	-0.0875870	-0.4375410	2.3105020
H	-1.3654440	-1.4991190	1.7808340
C	0.5883320	-2.1410290	1.1554180
H	0.5632250	-2.7999150	2.0377530
H	0.2152320	-2.7300700	0.3162240
C	2.0265660	-1.7435930	0.8532500

C	2.7377410	-1.0804310	1.8869620
C	2.5295740	-2.0967180	-0.4420640
O	4.0994990	-0.9265510	1.7334480
O	3.7785710	-1.6016950	-0.7624180
O	2.2069240	-0.6355570	2.9389520
O	1.9232450	-2.7764670	-1.2932160
C	4.2354560	-1.8804710	-2.0851500
H	4.3158980	-2.9573160	-2.2715500
H	3.5720780	-1.4442700	-2.8409670
H	5.2241180	-1.4189660	-2.1565070
C	4.7725770	-0.2279440	2.7798880
H	4.3602630	0.7744130	2.9331690
H	4.7227560	-0.7739160	3.7292030
H	5.8138910	-0.1495040	2.4562050
S	-2.7244090	-1.5029090	-0.8317440
O	-2.5211370	-2.6144970	0.1071370
N	1.2405570	1.4140570	0.9533990
H	1.3607480	1.0153770	1.8823370

Imaginary frequency = 0

E = -1927.709243

H = -1927.708299

G = -1927.802231

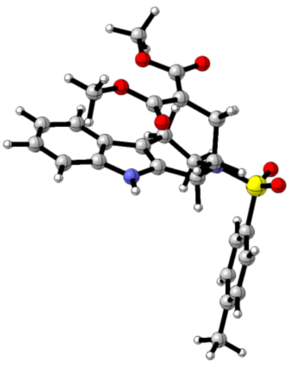

C

C	1.9462970	1.6802800	-1.0351670
C	2.8508150	2.1596820	-1.9989260
C	3.7776010	3.1297090	-1.6251660
C	3.8140720	3.6321170	-0.3045810
C	2.9281850	3.1752370	0.6712680
C	2.0042130	2.1974510	0.2927880
H	2.8310110	1.7701300	-3.0130090
H	4.4869210	3.5075590	-2.3562270
H	4.5487340	4.3885270	-0.0430380
H	2.9598290	3.5566930	1.6876100
C	0.9017520	0.6976490	-1.0490110
C	0.3841100	0.6383360	0.2279620
C	0.2909670	-0.1056490	-2.1482200
H	0.3089130	0.4459690	-3.0950370
H	0.8174620	-1.0532240	-2.3130360
C	-1.1669080	-0.3837450	-1.7786820
H	-1.7293910	0.5609770	-1.7847130
H	-1.6134940	-1.0644230	-2.5014400
C	-0.6983390	-0.2699870	0.7281620
H	-1.4820780	0.3579520	1.1816280
N	-1.2617020	-1.0343540	-0.4373330
C	-3.9504680	-0.3705670	-0.0014190
C	-4.2345120	0.1392780	1.2689720
C	-4.5137440	0.1979200	-1.1491890
C	-5.0940410	1.2328450	1.3840150
H	-3.8034300	-0.3197100	2.1521110
C	-5.3699310	1.2894840	-1.0131430
H	-4.2967170	-0.2142950	-2.1284610
C	-5.6743960	1.8234480	0.2505660
H	-5.3207910	1.6282440	2.3704750
H	-5.8136530	1.7305880	-1.9017690
C	-6.6308610	2.9814860	0.3824020
H	-6.5065990	3.6957390	-0.4379560

H	-7.6687010	2.6259160	0.3509770
H	-6.4910370	3.5114200	1.3292180
O	-3.1303640	-2.4718920	-1.3954150
C	-0.1427370	-1.1934250	1.8397250
H	0.2822370	-0.5516590	2.6156720
H	-0.9651630	-1.7468040	2.2907010
C	0.9174590	-2.2083990	1.3599660
H	1.1761670	-2.8222080	2.2341620
H	0.4754440	-2.8652580	0.6110830
C	2.1809240	-1.6197800	0.8067770
C	2.9974070	-0.8198140	1.7369540
C	2.5798000	-2.0264030	-0.5543630
O	4.0820810	-0.2296740	1.2084030
O	3.5905550	-1.3256910	-1.0946690
O	2.7039320	-0.7079660	2.9274920
O	2.0187420	-2.9387880	-1.1596970
C	3.9615850	-1.6771190	-2.4411090
H	4.3039720	-2.7146500	-2.4916870
H	3.1183810	-1.5402600	-3.1236930
H	4.7725010	-0.9960950	-2.7010580
C	4.8759810	0.5619350	2.1124140
H	4.2720340	1.3473660	2.5726940
H	5.3113470	-0.0679520	2.8938710
H	5.6602000	1.0013770	1.4958220
S	-2.7928360	-1.7308540	-0.1698440
O	-2.7460970	-2.4417940	1.1164400
N	1.0337550	1.5496550	1.0366650
H	0.8847340	1.6692730	2.0288470

Imaginary frequency = 0

E = -1927.567535

H = -1927.566591

G = -1927.664763

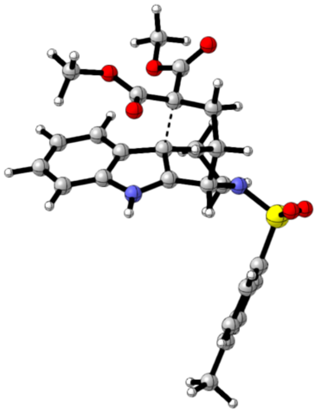

TS$_{CD}$

C	1.8132070	1.4170710	-0.7042630
C	2.7818390	1.8416560	-1.6128010
C	3.2941180	3.1395280	-1.5068840
C	2.8480140	4.0088200	-0.4945940
C	1.8644020	3.6144910	0.4159240
C	1.3515970	2.3219250	0.2857180
H	3.1471280	1.1654620	-2.3763220
H	4.0512070	3.4779720	-2.2082840
H	3.2676610	5.0084200	-0.4226130
H	1.5049060	4.2864460	1.1895870
C	1.1160160	0.1170870	-0.4861230
C	0.1116130	0.4570830	0.4941070
C	0.5504660	-0.7353880	-1.6379060
H	0.7639510	-0.2477440	-2.5937040
H	0.9878960	-1.7352230	-1.6843760
C	-0.9716720	-0.8664670	-1.4912600
H	-1.4481780	0.1078700	-1.6689220
H	-1.3535770	-1.5815300	-2.2203840
C	-0.7705630	-0.6000060	1.0375390
H	-1.5861900	-0.1624880	1.6227440
N	-1.3156050	-1.3759900	-0.1382270
C	-3.9620280	-0.5033140	-0.0481250

C	-4.3563140	0.1504650	1.1226120
C	-4.3350290	-0.0162780	-1.3060480
C	-5.1314780	1.3073130	1.0252380
H	-4.0754930	-0.2472490	2.0916980
C	-5.1085690	1.1407250	-1.3823470
H	-4.0356530	-0.5397160	-2.2075830
C	-5.5181000	1.8206700	-0.2224450
H	-5.4445380	1.8145420	1.9339260
H	-5.4034250	1.5197730	-2.3573610
C	-6.3819170	3.0525580	-0.3218580
H	-6.0812080	3.6817620	-1.1659860
H	-7.4311360	2.7730960	-0.4817880
H	-6.3341760	3.6512440	0.5925130
O	-3.1750240	-2.8032810	-1.1017210
C	0.0811560	-1.5255290	1.9325020
H	0.3968240	-0.9469360	2.8039470
H	-0.5472980	-2.3525990	2.2751540
C	1.3013150	-2.1049400	1.1979650
H	1.8802250	-2.7056240	1.9088520
H	0.9427990	-2.8041600	0.4386300
C	2.2598300	-1.1160570	0.5022970
C	3.0096720	-0.2392740	1.4491230
C	3.1213780	-1.8412360	-0.4988700
O	4.1822890	0.2150310	0.9619980
O	3.6674040	-1.0396830	-1.4291320
O	2.5942650	0.0756900	2.5582200
O	3.2768680	-3.0558880	-0.5066840
C	4.5174320	-1.6730850	-2.4050400
H	5.3616510	-2.1675150	-1.9167850
H	3.9539540	-2.4051460	-2.9905130
H	4.8729770	-0.8666480	-3.0475950
C	4.8687190	1.1952060	1.7644580
H	4.2352160	2.0727800	1.9180490
H	5.1546890	0.7743180	2.7324810
H	5.7562550	1.4663700	1.1918770
S	-2.8887270	-1.9371540	0.0538940
O	-2.9769620	-2.4699090	1.4234330
N	0.3364970	1.7039520	1.0065990
H	-0.1444330	2.1074210	1.7994210

Imaginary frequency = 1

E = -1927.539667

H = -1927.538723

G = -1927.634962

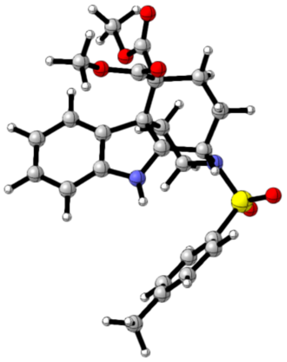

D

C	1.3732820	1.4157520	-0.6078730
C	2.3007790	2.1542370	-1.3327260
C	2.0991300	3.5322650	-1.5147130
C	0.9647400	4.1615480	-0.9834950
C	0.0109410	3.4351000	-0.2616350
C	0.2318360	2.0668240	-0.0873650
H	3.1800070	1.6737730	-1.7445780
H	2.8290570	4.1132210	-2.0710300
H	0.8200400	5.2281650	-1.1325820
H	-0.8724910	3.9137690	0.1510430
C	1.2492110	-0.0810670	-0.3108540
C	0.0873230	-0.0750870	0.6674400

C	0.7894710	-0.8553950	-1.6198580
H	1.0446050	-0.2615960	-2.4996090
H	1.3098180	-1.8075420	-1.7234990
C	-0.7175110	-1.1053920	-1.6302700
H	-1.2460580	-0.1436520	-1.6663660
H	-0.9910300	-1.6797890	-2.5158720
C	-0.5591730	-1.3829400	0.8937870
H	-1.3778240	-1.3081060	1.6106280
N	-1.1279820	-1.8921630	-0.4324120
C	-3.6110230	-0.6904630	-0.0920770
C	-3.8614200	-0.2712460	1.2182020
C	-3.8870050	0.1473560	-1.1784930
C	-4.3723730	1.0090700	1.4365730
H	-3.6663170	-0.9354700	2.0528600
C	-4.4003210	1.4216380	-0.9405640
H	-3.7078310	-0.1929660	-2.1923950
C	-4.6441980	1.8761710	0.3661880
H	-4.5682660	1.3353570	2.4545420
H	-4.6141550	2.0741760	-1.7830090
C	-5.2185300	3.2497830	0.6049980
H	-4.7979990	3.9828780	-0.0911330
H	-6.3052910	3.2426790	0.4524210
H	-5.0300990	3.5920360	1.6268680
O	-3.1530230	-2.7427580	-1.7157730
C	0.4958740	-2.3461730	1.4700500
H	0.6234510	-2.0737820	2.5211190
H	0.1100710	-3.3694590	1.4318810
C	1.8722890	-2.3032930	0.7771410
H	2.6105560	-2.7814190	1.4244920
H	1.8374510	-2.8956790	-0.1406880
C	2.4033120	-0.8739620	0.4182120
C	2.8161490	-0.1388110	1.7112610
C	3.7051710	-1.0761850	-0.3703260
O	3.4407640	1.0125670	1.4249380
O	3.6488150	-0.6449930	-1.6352120
O	2.6253990	-0.5384080	2.8427830
O	4.6874680	-1.6053100	0.1188460
C	4.8473540	-0.8128690	-2.4312590
H	5.6807060	-0.2705330	-1.9779910
H	5.0985260	-1.8725960	-2.5184020
H	4.6034740	-0.3958920	-3.4082240
C	3.8643370	1.8194430	2.5471050
H	3.0023720	2.0990540	3.1581630
H	4.5894360	1.2730280	3.1557740
H	4.3229860	2.7053170	2.1080330
S	-2.7750770	-2.2546030	-0.3778840
O	-2.9805090	-3.1025330	0.8089550
N	-0.5692650	1.1431110	0.5751390
H	-1.3924180	1.3856780	1.1116110

Imaginary frequency = 0

E = -1927.548324

H = -1927.547380

G = -1927.642637

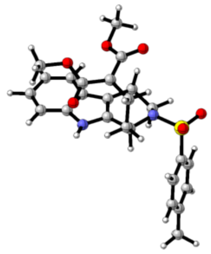

C

C	-2.1395060	1.7219300	1.1489690
C	-3.1093200	2.1026380	2.0939820
C	-4.1008810	3.0037860	1.7127600
C	-4.1400160	3.5339970	0.4032670

C	-3.1864430	3.1800750	-0.5518340
C	-2.1917160	2.2779060	-0.1633700
H	-3.0891730	1.6912090	3.0995070
H	-4.8606710	3.3035190	2.4291520
H	-4.9292340	4.2303750	0.1340400
H	-3.2157360	3.5850130	-1.5592910
C	-1.0231700	0.8215170	1.1677420
C	-0.4604160	0.8437270	-0.0899670
C	-0.4081100	0.0169660	2.2625660
H	-0.4619620	0.5456990	3.2213770
H	-0.9172740	-0.9454530	2.3958910
C	1.0628830	-0.2182420	1.9165780
H	1.6111440	0.7346280	1.9530500
H	1.5092970	-0.9085060	2.6290210
C	0.7315170	0.0626390	-0.5692990
H	1.5314130	0.7816440	-0.8130810
N	1.1783930	-0.8251430	0.5576990
C	3.8932100	-0.3624360	0.0489440
C	4.1919870	0.0453030	-1.2546100
C	4.4906660	0.2534030	1.1533520
C	5.1053050	1.0822430	-1.4470200
H	3.7258900	-0.4451630	-2.1022930
C	5.4002250	1.2886220	0.9400960
H	4.2586830	-0.0786860	2.1594020
C	5.7226350	1.7184870	-0.3579440
H	5.3433020	1.3990830	-2.4589240
H	5.8707460	1.7667660	1.7950430
C	6.7343220	2.8145790	-0.5771330
H	6.7440090	3.5222570	0.2575590
H	7.7437630	2.3918760	-0.6616880
H	6.5315240	3.3666450	-1.5000280
O	2.9697800	-2.3192450	1.5853760
C	0.3864770	-0.7067610	-1.8642140
H	-0.0404710	0.0096290	-2.5693370
H	1.2972450	-1.0949060	-2.3190970
C	-0.5873540	-1.8897230	-1.6670430
H	-0.7776110	-2.3048760	-2.6666590
H	-0.0940090	-2.6603800	-1.0764490
C	-1.9028890	-1.5577900	-1.0298470
C	-2.8355810	-0.7177960	-1.7934960
C	-2.2566420	-2.2679970	0.2188410
O	-4.0831920	-0.6481100	-1.2943480
O	-3.1364730	-1.6269080	1.0033310
O	-2.5100150	-0.1494850	-2.8361220
O	-1.7702840	-3.3551880	0.5159020
C	-3.5401390	-2.3118440	2.2057160
H	-4.0700900	-3.2366130	1.9595300
H	-2.6752790	-2.5460640	2.8318870
H	-4.2057190	-1.6185620	2.7205930
C	-5.0125770	0.1781570	-2.0236280
H	-4.6589420	1.2116920	-2.0574470
H	-5.1487750	-0.1987740	-3.0412690
H	-5.9479910	0.1183280	-1.4667860
S	2.6610210	-1.6364570	0.3187280
O	2.5491330	-2.4300930	-0.9132570
N	-1.1360590	1.7516170	-0.8852330
H	-1.0451570	1.8295280	-1.8881150

Imaginary frequency = 0

E = -1927.567607

H = -1927.566663

G = -1927.665693

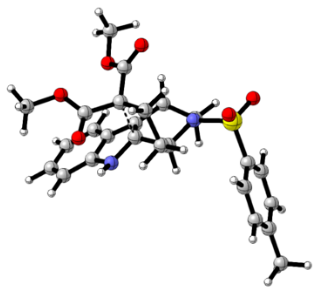

TS$_{CF}$

C	-2.1100300	1.7849720	1.0792360
C	-2.8950690	2.4403470	2.0525530
C	-3.7534260	3.4577720	1.6450040

C	-3.8391730	3.8331090	0.2860700
C	-3.0589110	3.2109770	-0.6975720
C	-2.1908450	2.2016730	-0.2873750
H	-2.8382730	2.1402440	3.0949500
H	-4.3731310	3.9673110	2.3771740
H	-4.5234800	4.6252800	-0.0042160
H	-3.1206380	3.5101590	-1.7397130
C	-1.1797070	0.7242330	1.1323870
C	-0.7849280	0.4014400	-0.2236480
C	-0.5065870	0.0696540	2.2834960
H	-0.6031760	0.6773480	3.1889680
H	-0.9370290	-0.9150770	2.5025800
C	0.9788690	-0.1034690	1.9461680
H	1.4623880	0.8807370	1.8599600
H	1.4711860	-0.6701890	2.7332560
C	0.6478340	-0.0622970	-0.5184420
H	1.2745260	0.8348340	-0.6247960
N	1.1174750	-0.8542910	0.6658260
C	3.8128510	-0.3538150	0.0983840
C	4.1564370	-0.1371990	-1.2384190
C	4.3325450	0.4527300	1.1168540
C	5.0317370	0.9029160	-1.5529220
H	3.7585770	-0.7760580	-2.0185470
C	5.2040040	1.4881390	0.7819930
H	4.0706570	0.2675810	2.1528290
C	5.5680010	1.7299350	-0.5533700
H	5.3045310	1.0703710	-2.5914300
H	5.6124200	2.1145120	1.5705950
C	6.5404490	2.8293260	-0.8979090
H	6.4413160	3.6782410	-0.2141060
H	7.5727070	2.4645200	-0.8196200
H	6.3946980	3.1873210	-1.9215150
O	2.9611210	-2.1417460	1.8653170
C	0.5620700	-0.8391210	-1.8346540
H	0.2470030	-0.1267740	-2.6044790
H	1.5099930	-1.2630890	-2.1563220
C	-0.5064190	-1.9172400	-1.6298350
H	-0.8297710	-2.3247300	-2.5921230
H	-0.0874030	-2.7321960	-1.0407360
C	-1.6879340	-1.3135430	-0.8694110
C	-2.7465540	-0.7882080	-1.7759030
C	-2.1506180	-2.1395740	0.2980290
O	-3.9952450	-0.8039010	-1.2801570
O	-2.9928420	-1.4874450	1.1167860
O	-2.4938900	-0.3453590	-2.8951140
O	-1.7922650	-3.2910950	0.5063470
C	-3.5059060	-2.2332170	2.2371350
H	-4.1021120	-3.0825900	1.8913920
H	-2.6913640	-2.5966240	2.8700120
H	-4.1316290	-1.5311860	2.7892210
C	-5.0187980	-0.2128200	-2.1082750
H	-4.8020510	0.8444860	-2.2841560
H	-5.0942760	-0.7370980	-3.0645010
H	-5.9437610	-0.3198220	-1.5410850
S	2.6317390	-1.6356980	0.5225230
O	2.5542770	-2.5859140	-0.5951970
N	-1.2750600	1.4683930	-1.0241380
H	-1.3752350	1.3682960	-2.0270820

Imaginary frequency = 1

E = -1927.552855

H = -1927.551911

G = -1927.647065

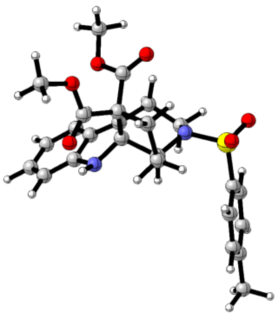

F

C	-1.7200380	2.2233350	0.8192260
C	-2.1673590	3.2477490	1.6864620

C	-2.7249580	4.4017340	1.1352150
C	-2.8363400	4.5542610	-0.2610340
C	-2.3746310	3.5612700	-1.1444480
C	-1.8117070	2.4130560	-0.5976550
H	-2.0938390	3.1237120	2.7631610
H	-3.0844470	5.1917110	1.7887640
H	-3.2809980	5.4588870	-0.6663110
H	-2.4420720	3.6928320	-2.2206700
C	-1.1540860	0.9590470	1.0434440
C	-0.9347070	0.2710100	-0.2792620
C	-0.5303850	0.4140810	2.2750650
H	-0.5734900	1.1443290	3.0892230
H	-1.0186150	-0.5026870	2.6297990
C	0.9385520	0.0890390	1.9713260
H	1.4859960	1.0142070	1.7379680
H	1.3972360	-0.3865810	2.8356040
C	0.5489230	-0.2269610	-0.4623260
H	1.1796270	0.6399100	-0.6931040
N	1.0061170	-0.8525940	0.8210190
C	3.7068450	-0.6038360	0.1297740
C	4.0187730	-0.6215270	-1.2319170
C	4.2955820	0.3270410	0.9927990
C	4.9323090	0.3080650	-1.7300810
H	3.5659740	-1.3543560	-1.8898810
C	5.2046160	1.2487680	0.4755130
H	4.0574770	0.3236830	2.0507420
C	5.5375790	1.2554750	-0.8893910
H	5.1795980	0.2938830	-2.7881700
H	5.6658310	1.9715600	1.1432330
C	6.5479670	2.2355890	-1.4293170
H	6.5123120	3.1850710	-0.8860080
H	7.5643160	1.8349150	-1.3230070
H	6.3825670	2.4359560	-2.4922180
O	2.8155640	-2.0300220	2.1830600
C	0.4053390	-1.2012700	-1.6316960
H	0.1506040	-0.6068200	-2.5162660
H	1.2955020	-1.7770900	-1.8678990
C	-0.7656590	-2.0856560	-1.2076720
H	-1.1920530	-2.6519130	-2.0397680
H	-0.4453810	-2.7983150	-0.4475740
C	-1.8017940	-1.1045620	-0.5683940
C	-2.9065030	-0.9576270	-1.6201650
C	-2.4098670	-1.6734960	0.7189830
O	-3.9828560	-1.6941630	-1.3271390
O	-3.3012990	-0.8232310	1.2467230
O	-2.7877590	-0.3553460	-2.6767840
O	-2.1297590	-2.7492200	1.2118460
C	-3.9634470	-1.2481160	2.4569890
H	-4.5473840	-2.1535900	2.2707670
H	-3.2328890	-1.4383140	3.2476100
H	-4.6178190	-0.4216450	2.7338700
C	-5.0345180	-1.7379840	-2.3238220
H	-5.4192540	-0.7325260	-2.5095730
H	-4.6557760	-2.1708590	-3.2529550
H	-5.8090980	-2.3716690	-1.8928970
S	2.4740160	-1.7317150	0.7819700
O	2.3168220	-2.8489720	-0.1603550
N	-1.2188810	1.3397510	-1.2419080
H	-1.5674680	1.0697620	-2.1542420

Imaginary frequency = 0

E = -1927.558241

H = -1927.557297

G = -1927.653029

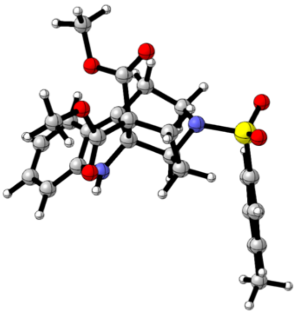

G

C	1.4494480	2.2762810	-0.8105070

C	1.6573800	3.4001080	-1.6720550
C	1.9441280	4.6071850	-1.0957800
C	2.0207620	4.7428620	0.3343830
C	1.8095600	3.6957630	1.2039990
C	1.5099880	2.4409690	0.6258990
H	1.6020050	3.2772040	-2.7482450
H	2.1217240	5.4830170	-1.7105660
H	2.2538500	5.7221770	0.7424640
H	1.8645790	3.8193390	2.2797240
C	1.1522130	0.9585850	-1.0669730
C	1.0013920	0.2226200	0.2411960
C	0.6518000	0.3672710	-2.3225790
H	0.7375220	1.0697290	-3.1544000
H	1.1591110	-0.5639550	-2.5879160
C	-0.8371930	0.0488590	-2.0594500
H	-1.3868620	0.9769330	-1.8501880
H	-1.2544550	-0.4213080	-2.9464390
C	-0.4955810	-0.3153840	0.3785660
H	-1.1433320	0.5245400	0.6553270
N	-0.9245030	-0.8952090	-0.9258930
C	-3.6111460	-0.7437810	-0.1654310
C	-3.9342870	-0.9051860	1.1845710
C	-4.1871140	0.2745420	-0.9333640
C	-4.8473190	-0.0272950	1.7685280
H	-3.4936660	-1.7074800	1.7655150
C	-5.0956080	1.1421460	-0.3292950
H	-3.9434600	0.3783680	-1.9852880
C	-5.4401180	1.0069050	1.0264830
H	-5.1069070	-0.1525390	2.8161800
H	-5.5511430	1.9308730	-0.9217050
C	-6.4530050	1.9260910	1.6592140
H	-6.4446850	2.9144820	1.1899150
H	-7.4640220	1.5152840	1.5423250
H	-6.2698340	2.0463700	2.7312870
O	-2.7375930	-2.0009920	-2.3403520
C	-0.3116870	-1.3550060	1.4900890
H	-0.0927500	-0.8183610	2.4190070
H	-1.1830670	-1.9774370	1.6699110

C	0.8956130	-2.1589670	1.0155890
H	1.3227960	-2.7930200	1.7955780
H	0.6256300	-2.7991130	0.1753450
C	1.9098500	-1.0758060	0.5231390
C	2.9030730	-0.8998440	1.6923340
C	2.7013530	-1.5266620	-0.7170370
O	4.0573820	-1.5085370	1.4440970
O	3.4460270	-0.5212770	-1.1948290
O	2.6246070	-0.3785310	2.7598190
O	2.6665020	-2.6432780	-1.1867120
C	4.2791260	-0.8165920	-2.3441770
H	5.0015100	-1.5955910	-2.0897080
H	3.6622110	-1.1408620	-3.1856030
H	4.7877030	0.1180860	-2.5776710
C	5.0271130	-1.5563140	2.5276650
H	5.3046190	-0.5425790	2.8235060
H	4.6077130	-2.0976920	3.3784100
H	5.8837530	-2.0875390	2.1155140
S	-2.3900800	-1.8088710	-0.9253260
O	-2.1844910	-2.9849790	-0.0722730
N	1.2216840	1.2816700	1.2192970
H	1.3825640	1.0824420	2.2010680

Imaginary frequency = 0

E = -1927.404553

H = -1927.403609

G = -1927.497265

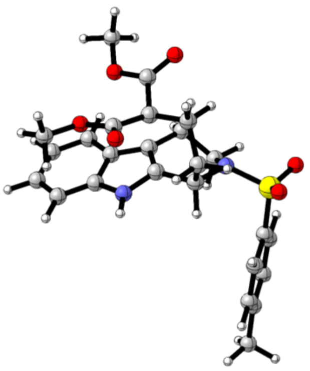

TS_{GE}

C	1.8228120	1.5282750	-0.9436240
C	2.6100610	2.1329700	-1.9480250
C	3.2740920	3.3056210	-1.6347580
C	3.1634000	3.8798240	-0.3426490
C	2.3988140	3.3030100	0.6696040
C	1.7329930	2.1213390	0.3519790
H	2.6896720	1.6787260	-2.9302020
H	3.8895970	3.7982020	-2.3801870
H	3.6967900	4.8024920	-0.1338560
H	2.3277610	3.7489630	1.6554690
C	1.0208290	0.3678310	-0.9247100
C	0.5017270	0.2403130	0.4117870
C	0.4170140	-0.4322840	-2.0236230
H	0.5449530	0.0849520	-2.9777410
H	0.8651570	-1.4267030	-2.1241340
C	-1.0759210	-0.5819360	-1.6989780
H	-1.5527660	0.4079490	-1.6920120
H	-1.5494770	-1.1940560	-2.4648230
C	-0.6506250	-0.6317550	0.8193530
H	-1.3783840	0.0128840	1.3297000
N	-1.2544720	-1.2715710	-0.3935780
C	-3.8898950	-0.3884700	-0.0398400
C	-4.1498310	0.1569560	1.2221830
C	-4.3831370	0.2103760	-1.2039200
C	-4.9109170	1.3225260	1.3096560
H	-3.7817320	-0.3313700	2.1181660
C	-5.1426810	1.3740760	-1.0934450
H	-4.1901160	-0.2318480	-2.1751780
C	-5.4185060	1.9480500	0.1589130
H	-5.1222680	1.7467650	2.2874700
H	-5.5341950	1.8397080	-1.9937260
C	-6.2741650	3.1840270	0.2667430
H	-6.1845070	3.8116080	-0.6251470
H	-7.3311900	2.9065910	0.3684020
H	-6.0064200	3.7809730	1.1438940
O	-3.1963710	-2.5557140	-1.4088780
C	-0.0855790	-1.6838110	1.8027750
H	0.2446120	-1.1622960	2.7052830
H	-0.8527880	-2.4015150	2.0837980
C	1.0859740	-2.3627990	1.0981220
H	1.5966660	-3.0418420	1.7938510
H	0.7284730	-2.9596020	0.2596210
C	2.1002360	-1.3686540	0.6042700
C	2.8839970	-0.6686840	1.6931780
C	2.8446470	-1.8064340	-0.6440280
O	3.6944960	0.2856280	1.2425920
O	3.7334060	-0.9298580	-1.1040810
O	2.7534950	-0.9593260	2.8702490
O	2.6201230	-2.8862360	-1.1669640
C	4.4566860	-1.3147870	-2.3001270
H	5.0335630	-2.2242120	-2.1166500
H	3.7607690	-1.4774860	-3.1265740
H	5.1178240	-0.4756380	-2.5127510
C	4.4571240	1.0084120	2.2414420
H	3.7880870	1.4666120	2.9732930
H	5.1454850	0.3265650	2.7460700
H	5.0047340	1.7681600	1.6865870
S	-2.8559560	-1.8432440	-0.1707030
O	-2.8857700	-2.5436470	1.1200360
N	0.9076760	1.3275440	1.1345190
H	0.6826550	1.4947940	2.1087340

Imaginary frequency = 1

E = -1927.377894

H = -1927.376949

G = -1927.470228

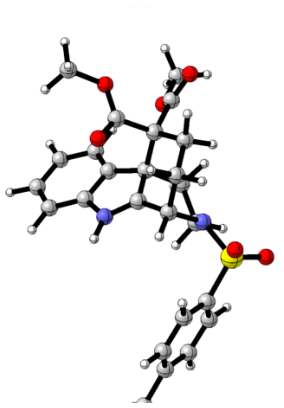

E

C	1.8352270	1.5121000	-0.5874960
C	2.8678540	2.0464700	-1.3514850
C	3.0923470	3.4310760	-1.3053440
C	2.3027590	4.2776240	-0.5172690
C	1.2531650	3.7620430	0.2511950
C	1.0599310	2.3910580	0.1846550
H	3.4916610	1.4134510	-1.9663480
H	3.8989140	3.8533450	-1.8966880
H	2.5022670	5.3442920	-0.5027330
H	0.6235420	4.3957620	0.8668620
C	1.2920720	0.0970930	-0.3878250
C	0.1682590	0.3664050	0.5758780
C	0.6015230	-0.5506070	-1.6571820
H	0.8255710	0.0619310	-2.5311840
H	1.0092720	-1.5466590	-1.8310970
C	-0.9184670	-0.6618040	-1.4954100
H	-1.3889480	0.3322350	-1.5137750
H	-1.3222650	-1.2482280	-2.3206580
C	-0.6971080	-0.7478280	1.0214430
H	-1.4981110	-0.3784750	1.6678040
N	-1.2315580	-1.3733850	-0.2315350
C	-3.9219380	-0.6284090	-0.0633310
C	-4.3332780	-0.1025730	1.1654190
C	-4.3329680	-0.0540090	-1.2716490
C	-5.1677170	1.0156830	1.1756430
H	-4.0258890	-0.5718490	2.0936270
C	-5.1655320	1.0630070	-1.2386740
H	-4.0210250	-0.4804880	-2.2187140
C	-5.5967360	1.6145430	-0.0197870
H	-5.4991430	1.4212040	2.1275450
H	-5.4936640	1.5082170	-2.1739620
C	-6.5325530	2.7957350	-0.0021510
H	-6.3165030	3.4877160	-0.8223280
H	-7.5701310	2.4595000	-0.1238010
H	-6.4713370	3.3427560	0.9432660
O	-3.0206820	-2.7761720	-1.3312980
C	0.2225450	-1.7309380	1.7827350
H	0.5229000	-1.2627590	2.7226900

H	-0.3656320	-2.6213940	2.0149410
C	1.4371770	-2.1270420	0.9398880
H	2.1164610	-2.7406560	1.5347300
H	1.1022710	-2.7512290	0.1071230
C	2.2641210	-0.9441130	0.3328230
C	2.9776320	-0.1735980	1.4587800
C	3.3128370	-1.5731290	-0.6081600
O	4.2971360	-0.1244100	1.3068120
O	3.5528170	-0.8401180	-1.6974720
O	2.3659050	0.3502190	2.3730440
O	3.8529240	-2.6324920	-0.3665460
C	4.5515430	-1.3531090	-2.6176570
H	5.5150850	-1.4412990	-2.1112340
H	4.2400690	-2.3264010	-3.0030880
H	4.6039680	-0.6205950	-3.4227380
C	5.0435850	0.6178060	2.3105140
H	4.7266130	1.6631210	2.3092160
H	4.8850830	0.1758360	3.2963550
H	6.0869490	0.5298070	2.0110670
S	-2.7960660	-2.0173900	-0.0940590
O	-2.8342850	-2.6753570	1.2199050
N	0.0608010	1.6277290	0.8462270
H	-0.6538130	2.0244410	1.4563470

Imaginary frequency = 0

E = -1927.416981

H = -1927.416037

G = -1927.508295

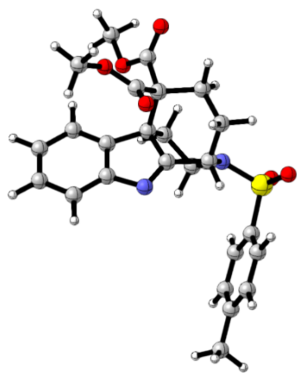

8a

C	1.8316780	1.4451360	-0.6056620	H	-6.1729030	3.5987780	-0.8272340
C	2.8773200	1.9485890	-1.3711920	H	-7.4651640	2.6242240	-0.1200770
C	3.1214800	3.3313950	-1.3548850	H	-6.3221930	3.4613430	0.9380060
C	2.3317450	4.1958380	-0.5861990	O	-3.0634880	-2.7586950	-1.3494380
C	1.2699750	3.6994490	0.1811960	C	0.1520590	-1.6911950	1.8288720
C	1.0365570	2.3273990	0.1557110	H	0.4281170	-1.1926770	2.7610190
H	3.5017860	1.2906750	-1.9616030	H	-0.4347190	-2.5800240	2.0746790
H	3.9382590	3.7343750	-1.9471450	C	1.4035560	-2.1133330	1.0487150
H	2.5419050	5.2616800	-0.5876370	H	2.0649900	-2.6941160	1.6948760
H	0.6431200	4.3554630	0.7776540	H	1.1020600	-2.7792380	0.2347990
C	1.2818220	0.0444300	-0.3719020	C	2.9623240	-0.1389280	1.4985290
C	0.1326870	0.3999100	0.5679350	C	3.3205510	-1.6210690	-0.4636180
C	0.6299760	-0.6514810	-1.6247080	O	4.1989570	0.2123720	1.1228150
H	0.8840190	-0.0915670	-2.5267490	O	3.5206550	-1.0099860	-1.6354650
H	1.0211740	-1.6626960	-1.7479070	O	2.4562440	0.1870860	2.5540950
C	-0.8953950	-0.7363780	-1.5049540	O	3.9264580	-2.6142290	-0.1090500
H	-1.3472030	0.2638370	-1.5664570	C	4.5581960	-1.5596750	-2.4824210
H	-1.2894190	-1.3434940	-2.3202880	H	5.5238810	-1.5093540	-1.9735130
C	-0.7325160	-0.7249800	1.0191510	H	4.3276960	-2.5959730	-2.7407650
H	-1.5534820	-0.3413610	1.6297760	H	4.5621940	-0.9353430	-3.3757470
N	-1.2506650	-1.3981010	-0.2251390	C	4.9179570	1.0987400	2.0143780
C	-3.9207900	-0.5978540	-0.0745230	H	4.3765130	2.0428310	2.1141800
C	-4.3348510	-0.0735040	1.1532720	H	5.0433890	0.6331680	2.9947310
C	-4.2956790	0.0049680	-1.2804040	H	5.8855260	1.2603520	1.5400800
C	-5.1340230	1.0701470	1.1658800	S	-2.8159840	-2.0084840	-0.1071330
H	-4.0471730	-0.5572550	2.0802010	O	-2.8951490	-2.6808380	1.1997370
C	-5.0934170	1.1475110	-1.2461510	N	-0.0093020	1.6475200	0.8431970
H	-3.9785050	-0.4170660	-2.2278180	C	2.2394900	-0.9557780	0.4114470
C	-5.5241840	1.6982430	-0.0271930	Imaginary frequency = 0			
H	-5.4594430	1.4789180	2.1186000	E = -1926.990138			
H	-5.3885740	1.6176600	-2.1804810	H = -1926.989194			
C	-6.4131470	2.9159180	-0.0061090	G = -1927.083799			

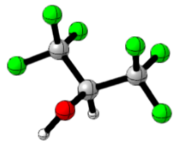

1,1,1,3,3,3-hexafluoroisopropyl alcohol (HFIP)

C	-1.7073120	0.3737710	0.0444600
H	-1.2689740	-0.6309060	0.0612590
C	-1.2300920	1.0392220	-1.2627110
C	-1.2103940	1.0899760	1.3103900
F	-1.6102440	2.3284350	-1.3670380
F	-1.7692710	0.3711250	-2.3118640
F	0.1126240	0.9908460	-1.3975800
F	-1.7282350	2.3283200	1.4413370
F	0.1361000	1.2019550	1.3209750
F	-1.5692620	0.3813050	2.4033160
O	-3.1083180	0.3770880	0.0984910
H	-3.4494730	-0.4166250	-0.3411350

Imaginary frequency = 0

E = -789.791416

H = -789.790472

G = -789.836161

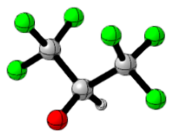

HFIP_anion

C	-1.8131750	0.3761190	0.0004550
H	-1.2797150	-0.6126160	-0.0074320
C	-1.2371540	1.0789190	-1.2720010
C	-1.2199920	1.0646540	1.2740880
F	-1.6835620	2.3514720	-1.4415760
F	-1.6112850	0.3903880	-2.3861970
F	0.1326880	1.1478830	-1.3269120
F	-1.5541740	2.3783520	1.3964030
F	0.1483040	1.0128630	1.3664840
F	-1.6831380	0.4459350	2.3935820
O	-3.1356830	0.3498030	0.0031050

Imaginary frequency = 0

E = -789.313782

H = -789.312838

G = -789.357939

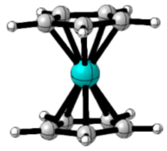

Ferrocene (Cp_2Fe)

Fe	-0.0000000	0.0007590	-0.0005320
C	1.6835050	-0.1886000	-1.2020830
C	1.6825790	-1.2019810	-0.1922280
C	1.6834370	1.0850120	-0.5503400
H	1.6514370	-0.3558480	-2.2706400
C	1.6819900	-0.5547080	1.0836120
H	1.6495210	-2.2698680	-0.3634570
C	1.6822470	0.8587070	0.8623370
H	1.6517750	2.0495560	-1.0396560
H	1.6483650	-1.0474860	2.0463280
H	1.6491430	1.6221600	1.6284430
C	-1.6819910	-0.5547230	1.0836040
C	-1.6825790	-1.2019780	-0.1922460
C	-1.6822470	0.8586940	0.8623500
H	-1.6483650	-1.0475140	2.0463140
C	-1.6835050	-0.1885830	-1.2020870
H	-1.6495220	-2.2698630	-0.3634890

C	-1.6834370	1.0850190	-0.5503250
H	-1.6491440	1.6221370	1.6284660
H	-1.6514360	-0.3558160	-2.2706480
H	-1.6517760	2.0495710	-1.0396270

Imaginary frequency = 0

E = -510.395470

H = -510.394526

G = -510.437990

H	1.6969830	1.9245070	1.2430880
H	1.6643430	1.7774140	-1.4528310

Imaginary frequency = 0

E = -510.197272

H = -510.196327

G = -510.241730

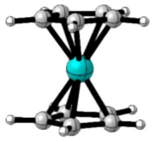

Cp_2Fe^+

Fe	-0.0006170	0.0082690	0.0101480
C	-1.7309180	1.0974160	0.5116400
C	-1.7218230	-0.1562570	1.2015970
C	-1.7083690	0.8290740	-0.8935740
H	-1.6959680	2.0753170	0.9718790
C	-1.7043000	-1.1998280	0.2229930
H	-1.6841550	-0.2911740	2.2741210
C	-1.6943720	-0.5908890	-1.0717890
H	-1.6575140	1.5693500	-1.6808700
H	-1.6467010	-2.2606080	0.4268220
H	-1.6318140	-1.1108910	-2.0177760
C	1.6987640	-1.2207580	0.0632970
C	1.7226750	-0.3173550	1.1726340
C	1.6939210	-0.4443870	-1.1396840
H	1.6407640	-2.2989790	0.1238090
C	1.7314210	1.0162690	0.6567780
H	1.6821460	-0.5934950	2.2175810
C	1.7096280	0.9384170	-0.7716230
H	1.6303510	-0.8325730	-2.1472730

Mavacurane skeleton (Scheme S2)

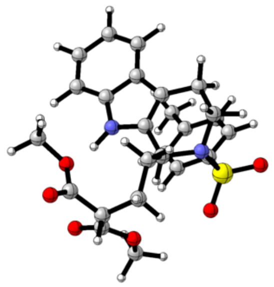

3a

C	-1.5941050	2.1583890	-1.0186140
C	-2.6940660	2.9912370	-1.2956040
C	-2.8368570	4.1811640	-0.5824320
C	-1.8969170	4.5583920	0.4037640
C	-0.7976070	3.7517780	0.7018450
C	-0.6618140	2.5550690	-0.0108020
H	-3.4210460	2.7079600	-2.0522870
H	-3.6831770	4.8317640	-0.7858620
H	-2.0337890	5.4923610	0.9419030
H	-0.0760410	4.0354090	1.4629400
C	-1.1392210	0.8912140	-1.5292290
C	-0.0013500	0.5689290	-0.8291200
C	-1.7458890	-0.0245990	-2.5478250
H	-2.7206550	-0.3883440	-2.2017880
H	-1.9315670	0.4968160	-3.4952350
C	-0.7955890	-1.2046540	-2.8294910
H	-1.3204290	-2.0378890	-3.2960680
H	-0.0078410	-0.8864620	-3.5182130
C	0.7793710	-0.7063240	-0.9474650
H	0.9821420	-1.0906430	0.0524760
N	-0.0791830	-1.7195760	-1.6308790
S	-0.9328000	-2.7678590	-0.6142720
C	-2.1193390	-1.8258740	0.3498700
C	-1.6769950	-1.1195320	1.4733840
C	-3.4498490	-1.7561680	-0.0674350
C	-2.5777830	-0.3120750	2.1638770
H	-0.6480590	-1.1959100	1.8025480
C	-4.3393340	-0.9455530	0.6396400
H	-3.7827540	-2.3198770	-0.9320790
C	-3.9167980	-0.1996040	1.7512700
H	-2.2349700	0.2445560	3.0319430
H	-5.3755140	-0.8879890	0.3175140
C	-4.8644570	0.7261580	2.4700030
H	-5.9070900	0.4352540	2.3113750
H	-4.6645770	0.7447280	3.5461150
H	-4.7448810	1.7518960	2.0975870
O	-1.6825870	-3.6748870	-1.5030640
O	0.0583170	-3.3188970	0.3273660
C	2.1361200	-0.5479500	-1.6963330
H	2.0239240	-0.9785760	-2.6956860
H	2.3405330	0.5150980	-1.8475520
C	3.3434930	-1.2321200	-1.0346960
H	3.0905670	-2.2608940	-0.7627360
H	4.1444210	-1.2904110	-1.7789470
C	3.9727130	-0.5519650	0.2187680
C	3.2236090	-0.6776860	1.5425390
C	4.3667660	0.8892270	-0.0708900
O	2.5500880	-1.8297680	1.6209660
O	3.3331800	1.7360760	0.0370740
O	3.3006790	0.1275620	2.4544730
O	5.4818030	1.2282000	-0.4261290
C	3.5751160	3.1230440	-0.3028720
H	4.3309890	3.5471370	0.3622930
H	3.9019440	3.2043750	-1.3422630
H	2.6180010	3.6247610	-0.1637790
C	1.8817020	-2.1189310	2.8727600
H	2.6228840	-2.3158110	3.6518960
H	1.2495530	-1.2795250	3.1698790
H	1.2796140	-3.0035830	2.6746080
H	4.9160990	-1.0785950	0.4082480
N	0.2928890	1.5625330	0.0842320
H	1.1232120	1.5917210	0.6611090

Imaginary frequency = 0

E = -1928.209886

H = -1928.208942

G = -1928.306909

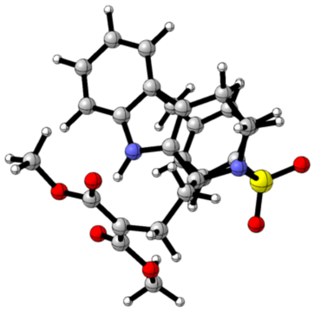

H

C	-0.7001230	2.1077840	-1.2498280
C	-1.4238530	3.2458530	-1.6533780
C	-1.1902590	4.4591260	-1.0060550
C	-0.2400120	4.5600440	0.0368740
C	0.4917520	3.4488280	0.4578380
C	0.2455760	2.2302140	-0.1865240
H	-2.1557690	3.1775960	-2.4543080
H	-1.7447870	5.3443930	-1.3061990
H	-0.0772890	5.5200170	0.5194580
H	1.2225970	3.5193240	1.2586830
C	-0.6867900	0.7275500	-1.6521540
C	0.2249700	0.0815920	-0.8428520
C	-1.5601710	0.0158400	-2.6405040
H	-2.6118870	0.1004570	-2.3370590
H	-1.4928280	0.4642660	-3.6399110
C	-1.1522780	-1.4651680	-2.7468800
H	-1.9596760	-2.0741140	-3.1531760
H	-0.2920950	-1.5688170	-3.4146120
C	0.4890610	-1.4062410	-0.8632110
H	0.5792690	-1.7809050	0.1585880
N	-0.7109710	-2.0666910	-1.4637960
S	-1.8953280	-2.5653650	-0.3723810
C	-2.5305170	-1.1412450	0.5183960
C	-1.7948130	-0.6302400	1.5925690
C	-3.7029640	-0.5181150	0.0860980
C	-2.2294790	0.5365010	2.2174350
H	-0.8932940	-1.1287670	1.9287570
C	-4.1273540	0.6455050	0.7293630
H	-4.2706170	-0.9320340	-0.7401300
C	-3.3915930	1.2002850	1.7888130
H	-1.6520200	0.9430860	3.0433180
H	-5.0387070	1.1345660	0.3958730
C	-3.8118830	2.4995400	2.4269370
H	-4.8847420	2.6773680	2.3065750
H	-3.5700010	2.5173810	3.4943280
H	-3.2813310	3.3377770	1.9567740
O	-2.9920700	-3.1290650	-1.1828880
O	-1.2039520	-3.4173010	0.6146380
C	1.7668270	-1.8400100	-1.6418680
H	1.5270550	-2.7555700	-2.1956440
H	2.0255550	-1.0687540	-2.3726510
C	2.9848570	-2.1023280	-0.7241360
H	2.8030760	-3.0149520	-0.1518280
H	3.8481710	-2.2945110	-1.3725680
C	3.2724010	-0.9473180	0.2190450
C	2.7514800	-0.9549180	1.5436070
C	3.8092460	0.2230830	-0.4062410
O	2.3426050	-2.2202990	1.9566000
O	3.9467550	1.3371800	0.3916520
O	2.5770830	0.0055000	2.3240220
O	4.1511170	0.2853990	-1.6039750
C	4.3857630	2.5295000	-0.2687950
H	5.3956040	2.4161430	-0.6775900
H	3.7055950	2.8104600	-1.0795480
H	4.3845450	3.3064120	0.4992230
C	1.7081460	-2.2927990	3.2373710
H	2.4089980	-2.0514270	4.0440520
H	0.8514330	-1.6152360	3.3026610
H	1.3708040	-3.3266160	3.3405140
N	0.7910290	0.9863610	0.0358370
H	1.4669150	0.7821290	0.7683260

Imaginary frequency = 0

E = -1927.736117

H = -1927.735173

G = -1927.832115

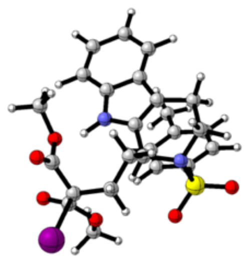

9

C	-3.0038670	1.8879880	-0.9098460
C	-4.2490790	2.5092410	-1.1206970
C	-4.5878600	3.6214830	-0.3502210
C	-3.7018090	4.1300920	0.6275560
C	-2.4614230	3.5338250	0.8604430
C	-2.1296900	2.4122250	0.0915530
H	-4.9358770	2.1235630	-1.8698130
H	-5.5477180	4.1084200	-0.5012800
H	-3.9927450	4.9996030	1.2108740
H	-1.7795500	3.9176490	1.6144240
C	-2.3411170	0.7452300	-1.4827830
C	-1.1417820	0.6128080	-0.8241540
C	-2.8159920	-0.2433410	-2.5038670
H	-3.7150630	-0.7563370	-2.1421920
H	-3.1008680	0.2513510	-3.4410150
C	-1.7009350	-1.2608810	-2.8208430
H	-2.1048540	-2.1688230	-3.2685790
H	-0.9994480	-0.8265550	-3.5388340
C	-0.1608450	-0.5090690	-1.0001780
H	0.1602240	-0.8564930	-0.0190960
N	-0.8681230	-1.6503630	-1.6510700
S	-1.5030180	-2.8172960	-0.6043010
C	-2.8198980	-2.0844850	0.3700790
C	-2.4896790	-1.2736490	1.4616060
C	-4.1493450	-2.2711190	-0.0138960
C	-3.5077590	-0.6214340	2.1531060
H	-1.4567640	-1.1451710	1.7618910
C	-5.1570010	-1.6137530	0.6949120
H	-4.3913390	-2.9105670	-0.8559720
C	-4.8538080	-0.7682280	1.7737650
H	-3.2538040	0.0180420	2.9943500
H	-6.1930210	-1.7546810	0.3984850
C	-5.9378210	-0.0047020	2.4908130
H	-6.9184710	-0.4673760	2.3450230
H	-5.7359450	0.0602590	3.5650200
H	-5.9925010	1.0223050	2.1064440
O	-2.0959160	-3.8561280	-1.4665120
O	-0.4101670	-3.1689630	0.3213820
C	1.1030380	-0.1238000	-1.8248670
H	1.0298810	-0.6072850	-2.8028640
H	1.0977900	0.9504510	-2.0221860
C	2.4624970	-0.5631440	-1.2382770
H	2.4121770	-1.6049270	-0.9132740
H	3.1930760	-0.5096760	-2.0497110
C	3.0352420	0.2861550	-0.0711480
C	2.3965160	0.1073360	1.3140210
C	3.0942400	1.7659880	-0.4900900
O	1.8416960	-1.0976200	1.4418370
O	1.9357000	2.3642970	-0.2023440
O	2.4402950	0.9457720	2.1947730
O	4.0060420	2.2916020	-1.0938230
C	1.7550790	3.7213820	-0.6851690
H	2.4989370	4.3792420	-0.2301050
H	1.8438190	3.7452930	-1.7738010
H	0.7493500	4.0022500	-0.3751610
C	1.2541450	-1.4204180	2.7275590
H	2.0400060	-1.4885720	3.4842780
H	0.5271950	-0.6566980	3.0123080
H	0.7674510	-2.3825170	2.5793670
I	5.1333080	-0.4366730	0.2920170
N	-1.0045710	1.6130680	0.1187570
H	-0.1975830	1.7526500	0.7107760

Imaginary frequency = 0

E = -1938.961749

H = -1938.960805

G = -1939.061965

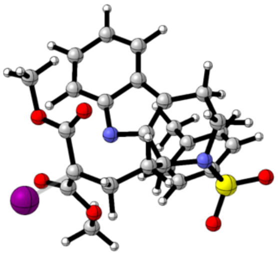

I

C	-1.5326560	2.1673960	-1.1109410
C	-2.1475340	3.3836490	-1.4723030
C	-1.7076870	4.5714880	-0.8812400
C	-0.6595740	4.5660770	0.0723220
C	-0.0383360	3.3738020	0.4537770
C	-0.4685570	2.1672170	-0.1265370
H	-2.9558130	3.4013520	-2.2019280
H	-2.1752360	5.5150160	-1.1546810
H	-0.3339360	5.5065850	0.5116290
H	0.7728090	3.3686780	1.1772160
C	-1.6957750	0.7957660	-1.4867670
C	-0.7700370	0.0979740	-0.7106200
N	-0.0204640	0.8882560	0.1079830
C	-2.6328320	0.1355020	-2.4543300
H	-3.6700920	0.1583620	-2.0920610
H	-2.6420110	0.6466690	-3.4272440
C	-2.1891030	-1.3203400	-2.6903550
H	-2.9807670	-1.9168550	-3.1449660
H	-1.3308640	-1.3407290	-3.3684360
C	-0.5541060	-1.3866860	-0.7799480
H	-0.4927780	-1.8100960	0.2230640
N	-1.7247910	-2.0240720	-1.4644300
S	-2.8988140	-2.6561360	-0.4411210
C	-3.6277330	-1.3256560	0.5211650
C	-2.9372480	-0.8301720	1.6325060
C	-4.8342700	-0.7549960	0.1102100
C	-3.4513690	0.2699820	2.3154580
H	-2.0119040	-1.2901620	1.9569580
C	-5.3383030	0.3426300	0.8108530
H	-5.3666450	-1.1572460	-0.7448960
C	-4.6498760	0.8822870	1.9091960
H	-2.9108750	0.6632530	3.1725030
H	-6.2770830	0.7895620	0.4940940
C	-5.1651390	2.1062740	2.6232160
H	-6.2410290	2.2362250	2.4718070
H	-4.9658020	2.0540600	3.6985560
H	-4.6646890	3.0059910	2.2420040
O	-3.9526030	-3.2202050	-1.3076690
O	-2.1899480	-3.5404040	0.5060100
C	0.7407870	-1.7593630	-1.5295350
H	0.6511410	-2.8049300	-1.8474690
H	0.8250060	-1.1458980	-2.4285360
C	2.0740760	-1.6955670	-0.7457680
H	2.0871300	-2.4854250	0.0073640
H	2.8405830	-1.9444610	-1.4825110
C	2.5257270	-0.3942350	-0.0493670
C	1.9770620	-0.1141260	1.3622490
C	2.5861790	0.8047570	-0.9948890
O	1.2410680	-1.1577520	1.7885890
O	2.8677250	1.9645750	-0.4061740
O	2.2856710	0.8211260	2.0727780
O	2.4641660	0.6699930	-2.2028730
C	2.9612410	3.1095900	-1.2809570
H	3.7900840	2.9828070	-1.9837190
H	2.0271680	3.2504600	-1.8289070
H	3.1464780	3.9590050	-0.6237520
C	0.6203690	-0.9982340	3.0787100
H	1.3774890	-0.8739000	3.8579340
H	-0.0449020	-0.1305950	3.0672240
H	0.0527250	-1.9142680	3.2450210
I	4.7896450	-0.7876100	0.4093370

Imaginary frequency = 0

E = -1938.478302

H = -1938.477358

G = -1938.577657

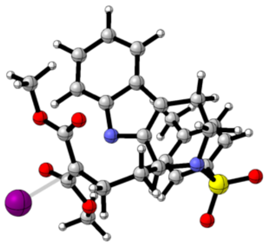

TS$_{IJ}$

C	-1.3661910	2.2048260	-1.1427920
C	-1.8682850	3.4726070	-1.4822120
C	-1.2952550	4.6073540	-0.8960810
C	-0.2383190	4.4910290	0.0339390
C	0.2654660	3.2390300	0.4022230
C	-0.2941240	2.0959580	-0.1848760
H	-2.6934240	3.5731520	-2.1844760
H	-1.6720140	5.5940530	-1.1552120
H	0.1890030	5.3902830	0.4712630
H	1.0820160	3.1383030	1.1093480
C	-1.6874440	0.8514330	-1.5181950
C	-0.8364110	0.0561790	-0.7703300
N	-0.0030160	0.7625930	0.0514660
C	-2.7093560	0.2911560	-2.4599290
H	-3.7256740	0.3933720	-2.0563110
H	-2.7078890	0.8276560	-3.4182510
C	-2.3911120	-1.1904130	-2.7420530
H	-3.2410990	-1.7070530	-3.1882430
H	-1.5563460	-1.2600200	-3.4455930
C	-0.7398390	-1.4324910	-0.8637760
H	-0.6962790	-1.8780040	0.1320200
N	-1.9524460	-1.9678780	-1.5507640
S	-3.1455800	-2.5419530	-0.5068480
C	-3.6840350	-1.2019930	0.5617930
C	-2.9181870	-0.8672080	1.6847260
C	-4.8209570	-0.4658260	0.2207860
C	-3.2845020	0.2384010	2.4493350
H	-2.0489480	-1.4537000	1.9588500
C	-5.1765400	0.6343840	1.0030490
H	-5.4158950	-0.7454910	-0.6419620

C	-4.4084220	1.0139300	2.1155550
H	-2.6852730	0.5055700	3.3158990
H	-6.0615260	1.2082960	0.7410300
C	-4.7627210	2.2364010	2.9238880
H	-5.8127090	2.5141560	2.7916650
H	-4.5736250	2.0768350	3.9905450
H	-4.1497160	3.0907900	2.6087050
O	-4.2854720	-2.9436090	-1.3532920
O	-2.4892870	-3.5462920	0.3534350
C	0.5442080	-1.8365980	-1.6037200
H	0.4794890	-2.8972540	-1.8715420
H	0.6339700	-1.2575940	-2.5259660
C	1.8403830	-1.6873360	-0.7702930
H	1.8513300	-2.4511150	0.0071840
H	2.6529540	-1.9124500	-1.4622290
C	2.1854400	-0.3534410	-0.1087710
C	1.8494140	-0.1015060	1.3466350
C	2.5044250	0.7726700	-1.0515720
O	1.1907830	-1.1694610	1.8497100
O	2.8799920	1.9131340	-0.4741900
O	2.1732510	0.8585980	2.0190110
O	2.4342530	0.6172360	-2.2649530
C	3.1798140	3.0037690	-1.3698180
H	4.0386910	2.7524760	-1.9989770
H	2.3166520	3.2368590	-1.9975560
H	3.4170710	3.8491490	-0.7238860
C	0.6731280	-1.0139120	3.1834900
H	1.4850420	-0.8600130	3.8998970
H	-0.0182340	-0.1670580	3.2222860
H	0.1470690	-1.9436610	3.4031810
I	4.7960610	-0.9565390	0.4305510

Imaginary frequency = 1

E = -1938.476144

H = -1938.475200

G = -1938.575577

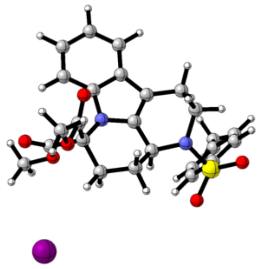

J

C	-1.5226410	3.0547400	0.0637040
C	-2.1483910	4.2882950	0.3149070
C	-1.3977940	5.3265220	0.8649410
C	-0.0298310	5.1489310	1.1633700
C	0.6151980	3.9337410	0.9245260
C	-0.1404660	2.8887880	0.3806850
H	-3.2008350	4.4251650	0.0806010
H	-1.8677920	6.2856140	1.0654480
H	0.5377720	5.9737140	1.5854400
H	1.6719680	3.8290600	1.1433610
C	-1.9779340	1.8085530	-0.4952140
C	-0.9124590	0.9568570	-0.4762670
N	0.2204820	1.5789680	0.0526290
C	-3.2546000	1.3952440	-1.1616880
H	-3.9691960	0.9502210	-0.4595470
H	-3.7503300	2.2573430	-1.6228110
C	-2.8869440	0.3893140	-2.2693700
H	-3.7738240	-0.0203980	-2.7537850
H	-2.2974270	0.9000200	-3.0373600
C	-0.9106450	-0.4796810	-0.8818430
H	-1.0468050	-1.0708280	0.0325240
N	-2.0633500	-0.7547300	-1.7885160
S	-2.8711640	-2.2008970	-1.4981670
C	-3.9371690	-1.9619480	-0.0776710
C	-3.4274760	-2.1640940	1.2090510
C	-5.2457240	-1.5075320	-0.2737770
C	-4.2421440	-1.8930630	2.3094820
H	-2.4196710	-2.5394550	1.3505890
C	-6.0446570	-1.2456110	0.8385120
H	-5.6324460	-1.3650100	-1.2770780
C	-5.5560240	-1.4244860	2.1444140
H	-3.8518020	-2.0540960	3.3108770
H	-7.0643190	-0.8995390	0.6905900
C	-6.4184380	-1.1009590	3.3383220
H	-7.4742680	-1.3049050	3.1336770
H	-6.1146360	-1.6750600	4.2189200
H	-6.3336980	-0.0362830	3.5923380
O	-3.7325470	-2.4460480	-2.6688920
O	-1.8349040	-3.1834190	-1.1320600
C	0.4572670	-0.8673360	-1.4534040
H	0.4947460	-1.9467220	-1.6172820
H	0.6202920	-0.3692380	-2.4168000
C	1.5164750	-0.4609840	-0.4197420
H	1.2734630	-0.9153290	0.5452770
H	2.5108720	-0.8114890	-0.6958320
C	1.5667790	1.0801270	-0.2182240
C	2.5119800	1.3960260	0.9645950
C	2.1314470	1.7698600	-1.4944490
O	2.0145520	0.9333430	2.1101070
O	3.2063930	1.1328760	-1.9570160
O	3.5706680	1.9851040	0.8557440
O	1.6375050	2.7516170	-2.0128600
C	3.8467390	1.7041310	-3.1246560
H	3.1507060	1.7185130	-3.9671000
H	4.1904770	2.7182110	-2.9051600
H	4.6922250	1.0504460	-3.3370650
C	2.8202030	1.1496890	3.2976880
H	3.7879520	0.6548930	3.1870000
H	2.9602230	2.2205000	3.4649310
H	2.2519810	0.7041800	4.1135430
I	4.8702490	-2.5987430	0.6477180

Imaginary frequency = 0

E = -1938.544832

H = -1938.543888

G = -1938.650681

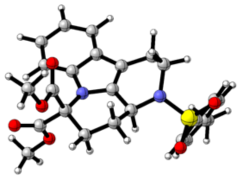

4a

C	0.8199080	1.9938110	-1.4152270
C	0.8879610	3.2091140	-2.1185980
C	2.0258120	4.0031220	-1.9860110
C	3.0989300	3.5970100	-1.1643420
C	3.0568790	2.3951790	-0.4559070
C	1.9101620	1.6036970	-0.5806240
H	0.0636190	3.5191820	-2.7551420
H	2.0920720	4.9456070	-2.5225230
H	3.9792570	4.2282260	-1.0822280
H	3.9028480	2.0925280	0.1514500
C	-0.1674040	0.9494190	-1.3312730
C	0.3228700	0.0142010	-0.4676090
N	1.5826980	0.3802140	0.0076280
C	-1.4393070	0.6824400	-2.0766700
H	-2.3194450	1.0864920	-1.5628380
H	-1.4107320	1.1381560	-3.0730450
C	-1.5622740	-0.8445640	-2.2394190
H	-2.5065810	-1.1259230	-2.7063170
H	-0.7545910	-1.2034020	-2.8849360
C	-0.3733410	-1.2177450	0.0042120
H	-0.8444760	-0.9727790	0.9645290
N	-1.4495640	-1.5954460	-0.9572320
S	-2.8881230	-2.1060930	-0.2485010
C	-3.7746130	-0.6538550	0.3150580
C	-3.5074480	-0.1318280	1.5851560
C	-4.6731870	-0.0231270	-0.5507850
C	-4.1447450	1.0443660	1.9799480
H	-2.8275290	-0.6405240	2.2597860
C	-5.3025590	1.1501750	-0.1358560
H	-4.8798240	-0.4438280	-1.5287820
C	-5.0441370	1.7067520	1.1278610
H	-3.9453350	1.4498290	2.9682810
H	-6.0073400	1.6388670	-0.8031390
C	-5.7002580	2.9954460	1.5539080
H	-6.6518130	3.1493250	1.0363100
H	-5.8810520	3.0125350	2.6332090
H	-5.0527750	3.8494320	1.3164570
O	-3.6857590	-2.7315640	-1.3181440
O	-2.5030930	-2.8943810	0.9363630
C	0.6478780	-2.3243150	0.2864030
H	0.1483110	-3.1760910	0.7528870
H	1.1051640	-2.6652490	-0.6501270
C	1.6959260	-1.7461590	1.2481160
H	1.1925970	-1.3518840	2.1363400
H	2.3999200	-2.5113120	1.5753180
C	2.5042200	-0.5829120	0.6003240
C	3.3549890	0.0871790	1.7053970
C	3.4571890	-1.1399900	-0.4990910
O	2.5825670	0.7687340	2.5504250
O	4.0996890	-2.2315310	-0.0838290
O	4.5629790	-0.0147630	1.7980160
O	3.5786780	-0.6502590	-1.6036720
C	5.0434700	-2.8199230	-1.0149240
H	4.5294010	-3.1247490	-1.9294790
H	5.8336310	-2.1025850	-1.2496840
H	5.4536410	-3.6867840	-0.4979340
C	3.2577730	1.4387760	3.6472800
H	3.7727970	0.7068140	4.2738550
H	3.9706950	2.1684940	3.2564790
H	2.4669170	1.9362400	4.2072830

Imaginary frequency = 0

E = -1927.006736

H = -1927.005792

G = -1927.101576

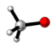

CH_3O^-

C	-0.0000110	0.0000740	-0.5596660
H	0.0001620	1.0223560	-1.0322110
H	-0.8850160	-0.5108050	-1.0331130
H	0.8850450	-0.5109830	-1.0328430
O	-0.0001800	-0.0006420	0.8071400

Imaginary frequency = 0

E = -115.180609

H = -115.179665

G = -115.205789

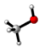

CH_3OH

C	-1.3224400	2.5068830	0.0027490
H	-0.9465510	1.4804600	0.0239910
H	-0.9604470	2.9947740	-0.9124240
H	-2.4203340	2.4786470	-0.0184330
O	-0.8294880	3.1559480	1.1824620
H	-1.1517720	4.0695130	1.1836000

Imaginary frequency = 0

E = -115.687908

H = -115.686964

G = -115.713993

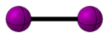

I_2

I	-1.7961670	4.2152580	0.0000000
I	-4.6620570	4.2152580	-0.0000000

Imaginary frequency = 0

E = -22.764655

H = -22.763711

G = -22.793573

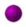

I^-

I	-1.8991120	4.2152580	0.0000000

Imaginary frequency = 0

E = -11.561285

H = -11.560341

G = -11.579549

8. Reference

[1] Bailey, P. D.; Beard, M. A.; Phillips, T. R. “Unexpected cis selectivity in the Pictet–Spengler reaction” *Tetrahedron Lett.* **2009**, *50*, 3645–3647.

[2] Wilson, D. M.; Britton, R. “Enantioselective Total Synthesis of the Marine Macrolides Salarins A and C” *J. Am. Chem. Soc.* **2024**, *146*, 8456–8463.

[3] Ren, W.; Tappin, N.; Wang, Q.; Zhu, J. “Synthetic Study towards Strictamine: The Oxidative Coupling Approach” *Synlett* **2013**, *24*, 1941–1944.

[4] Boumendjel, A.; Nuzillard, J.-M.; Massiot, G. “Synthesis of ajmalicine derivatives using Wittig–Horner and Knoevenagel reactions” *Tetrahedron Lett.* **1999**, *40*, 9033–9036.

[5] Gaussian 16, Revision C.01, Frisch, M. J.; Trucks, G. W.; Schlegel, H. B.; Scuseria, G. E.; Robb, M. A.; Cheeseman, J. R.; Scalmani, G.; Barone, V.; Petersson, G. A.; Nakatsuji, H.; Li, X.; Caricato, M.; Marenich, A. V.; Bloino, J.; Janesko, B. G.; Gomperts, R.; Mennucci, B.; Hratchian, H. P.; Ortiz, J. V.; Izmaylov, A. F.; Sonnenberg, J. L.; Williams-Young, D.; Ding, F.; Lipparini, F.; Egidi, F.; Goings, J.; Peng, B.; Petrone, A.; Henderson, T.; Ranasinghe, D.; Zakrzewski, V. G.; Gao, J.; Rega, N.; Zheng, G.; Liang, W.; Hada, M.; Ehara, M.; Toyota, K.; Fukuda, R.; Hasegawa, J.; Ishida, M.; Nakajima, T.; Honda, Y.; Kitao, O.; Nakai, H.; Vreven, T.; Throssell, K.; Montgomery, J. A., Jr.; Peralta, J. E.; Ogliaro, F.; Bearpark, M. J.; Heyd, J. J.; Brothers, E. N.; Kudin, K. N.; Staroverov, V. N.; Keith, T. A.; Kobayashi, R.; Normand, J.; Raghavachari, K.; Rendell, A. P.; Burant, J. C.; Iyengar, S. S.; Tomasi, J.; Cossi, M.; Millam, J. M.; Klene, M.; Adamo, C.; Cammi, R.; Ochterski, J. W.; Martin, R. L.; Morokuma, K.; Farkas, O.; Foresman, J. B.; Fox, D. J. Gaussian, Inc., Wallingford CT, **2016**.

[6] Becke, A. D. “Density-functional thermochemistry. III. The role of exact exchange” *J. Chem. Phys.* **1993**, *98*, 5648−5652.

[7] Lee, C.; Yang, W.; Parr, R. G. “Development of the Colle-Salvetti correlation-energy formula into a functional of the electron density” *Phys. Rev. B* **1988**, *37*, 785−789.

[8] Hay, P. J.; Wadt, W. R. “*Ab initio* effective core potential for molecular calculations. Potentials for the transition metal atoms Sc to Hg” *J. Chem. Phys.* **1985**, *82*, 270−283.

[9] Wadt, W. R.; Hay, P. J. “*Ab initio* effective core potential for molecular calculations. Potentials for main group elements Na to Bi” *J. Chem. Phys.* **1985**, *82*, 284−298.

[10] Hay, P. J.; Wadt, W. R. “*Ab initio* effective core potential for molecular calculations. Potentials for K to Au including the outermost core orbitals” *J. Chem. Phys.* **1985**, *82*, 299−310.

[11] Marenich, A. V.; Cramer, C. J.; Truhlar, D. G. “Universal Solvation Model Based on Solute Electron Density and on a Continuum Model of the Solvent Defined by the Bulk Dielectric Constant and Atomic Surface Tensions” *J. Phys. Chem. B* **2009**, *113*, 6378–6396.

[12] Grimme, S.; Antony, J.; Ehrlich, S.; Krieg, H. “A consistent and accurate *ab initio* parametrization of density functional dispersion correction (DFT-D) for the 94 elements H-Pu” *J. Chem. Phys.* **2010**, *132*, 154104.

[13] CYLView20; Legault, C. Y. Université de Sherbrooke, Canada, **2020** (http://www.cylview.org).

9. NMR spectra

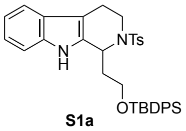

S1a

^{1}H NMR ($CDCl_3$, 600 MHz)

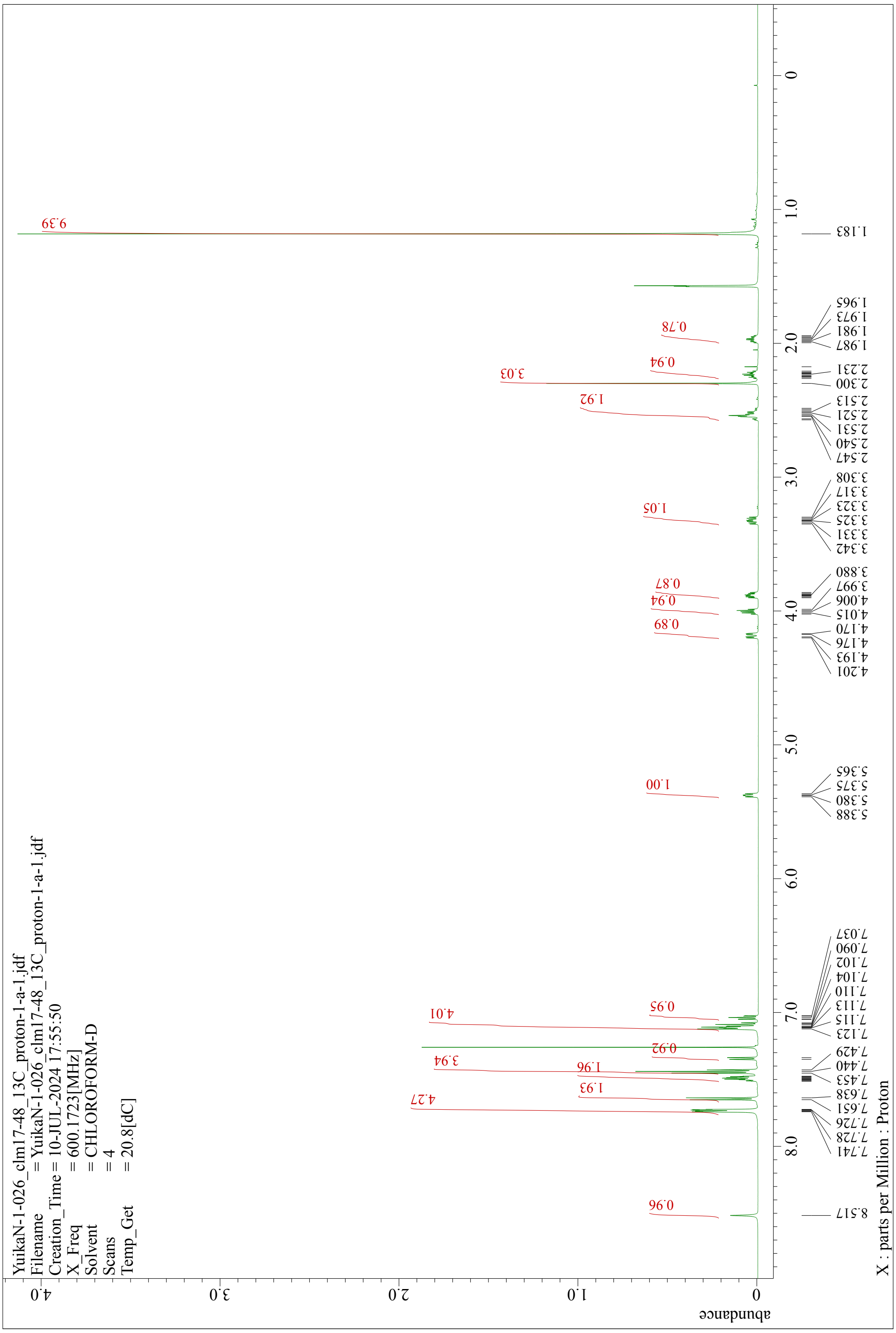

$^{13}C\{^{1}H\}$ NMR ($CDCl_3$, 150 MHz)

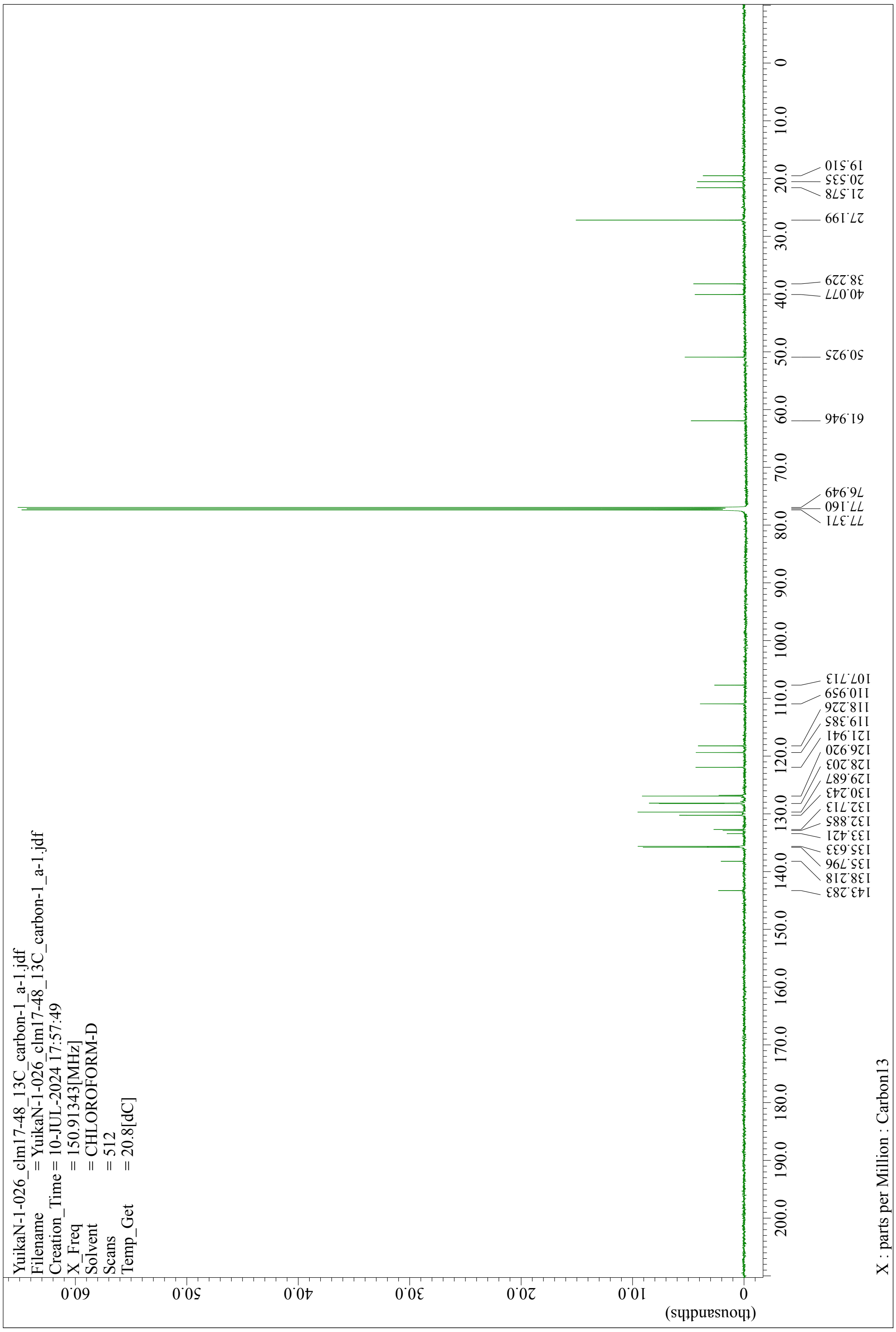

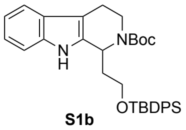

S1b

^{1}H NMR ($CDCl_3$, 600 MHz)

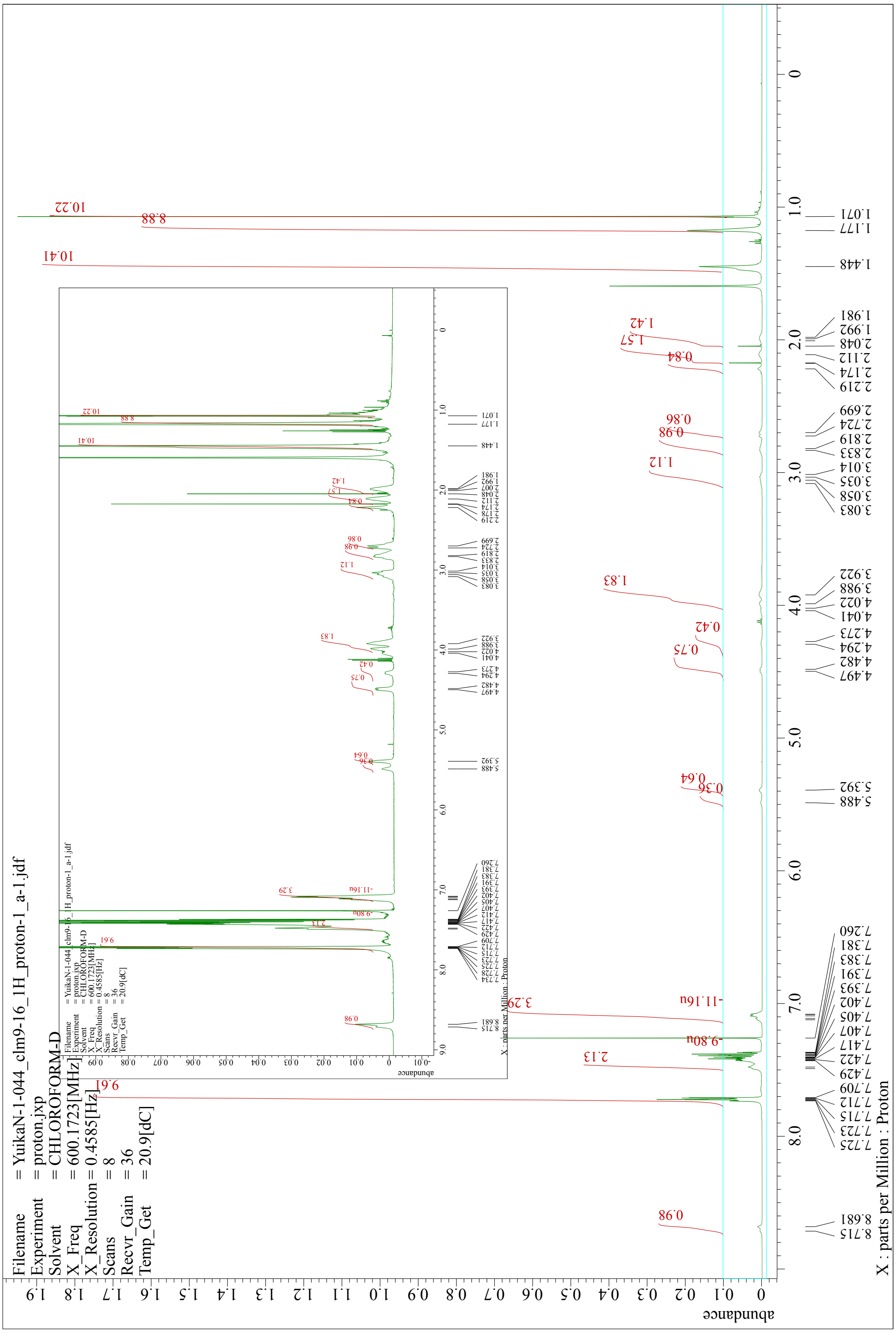

$^{13}C\{^{1}H\}$ NMR ($CDCl_3$, 150 MHz)

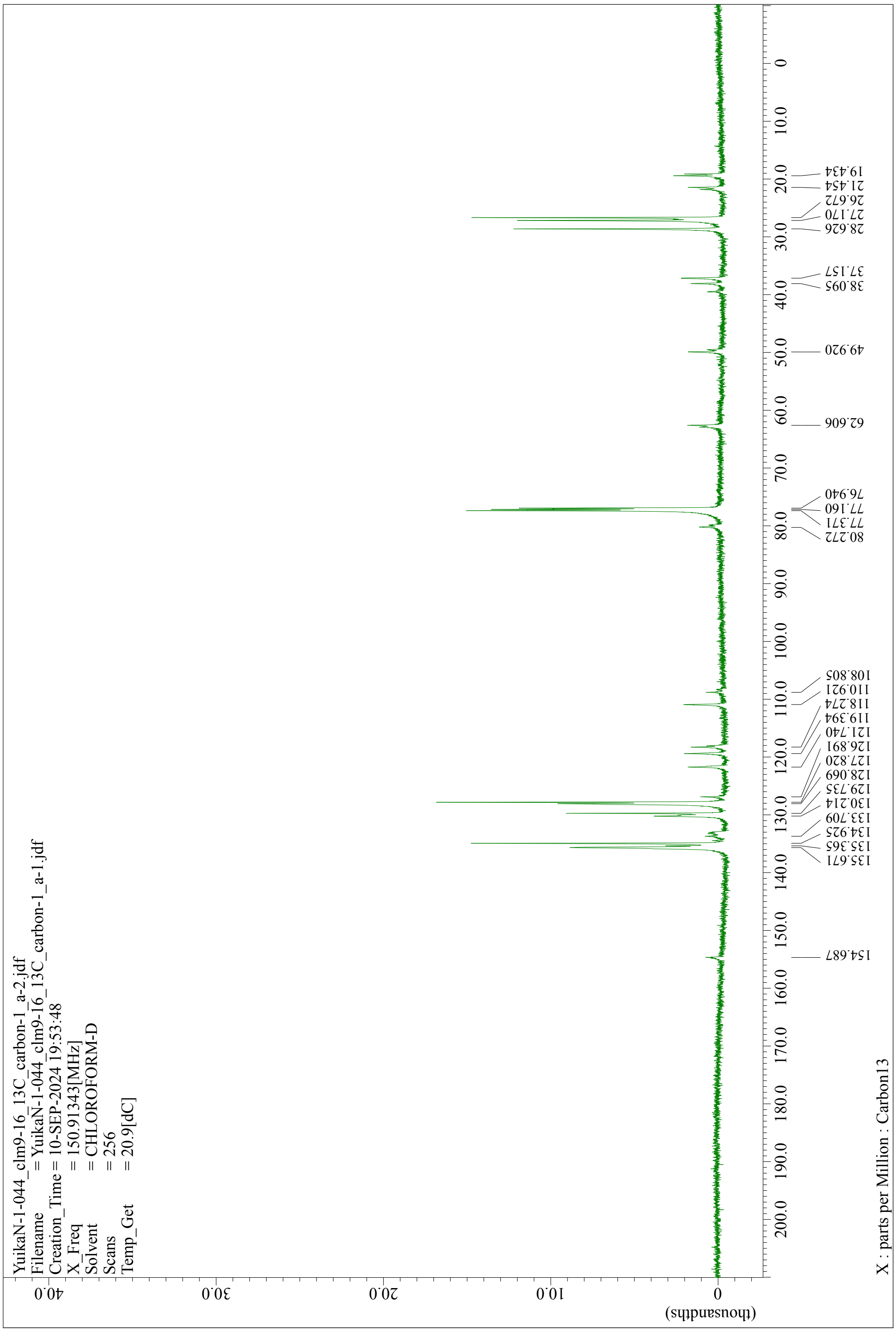

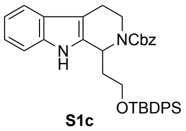

S1c

^{1}H NMR ($CDCl_3$, 600 MHz)

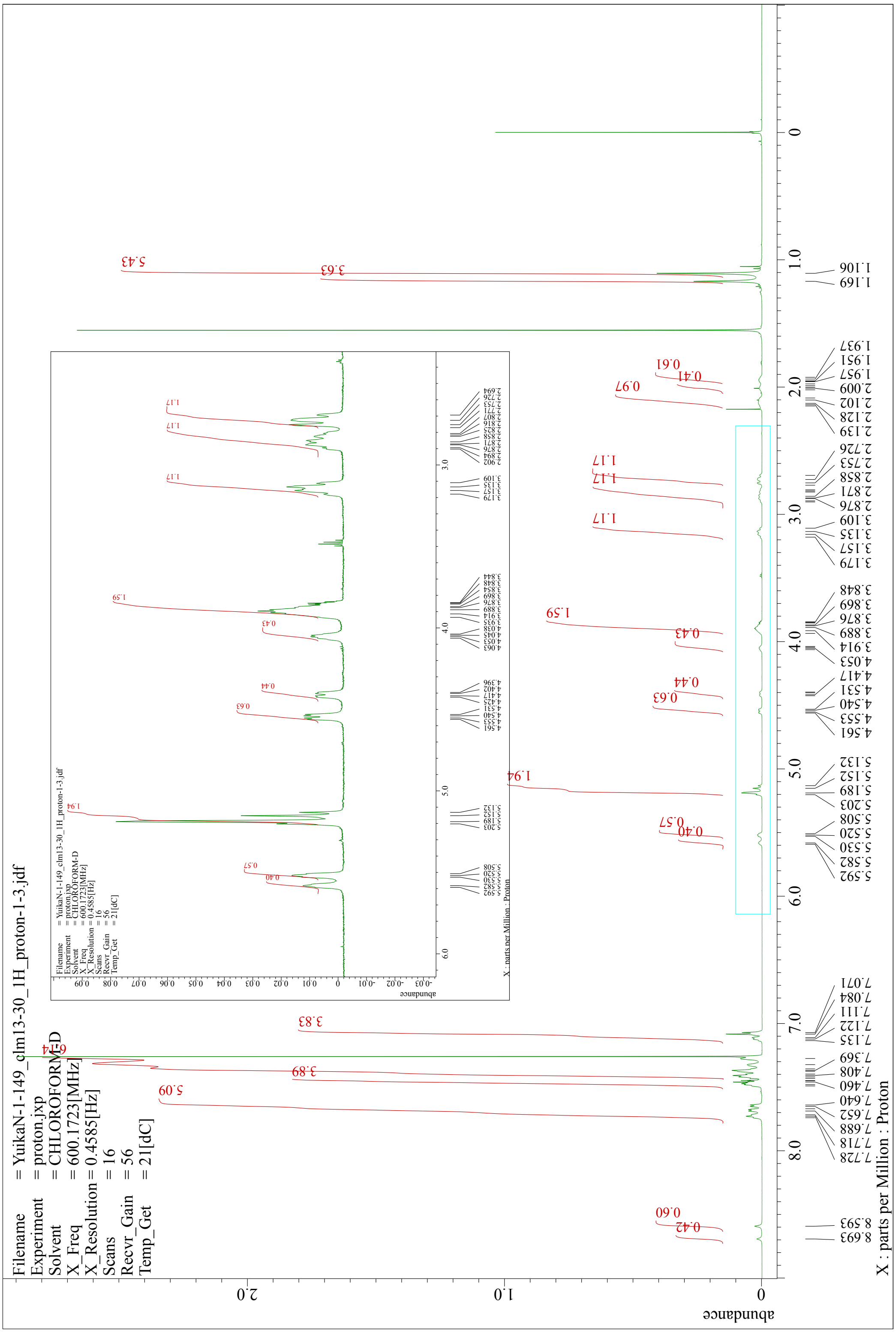

$^{13}C\{^{1}H\}$ NMR ($CDCl_3$, 150 MHz)

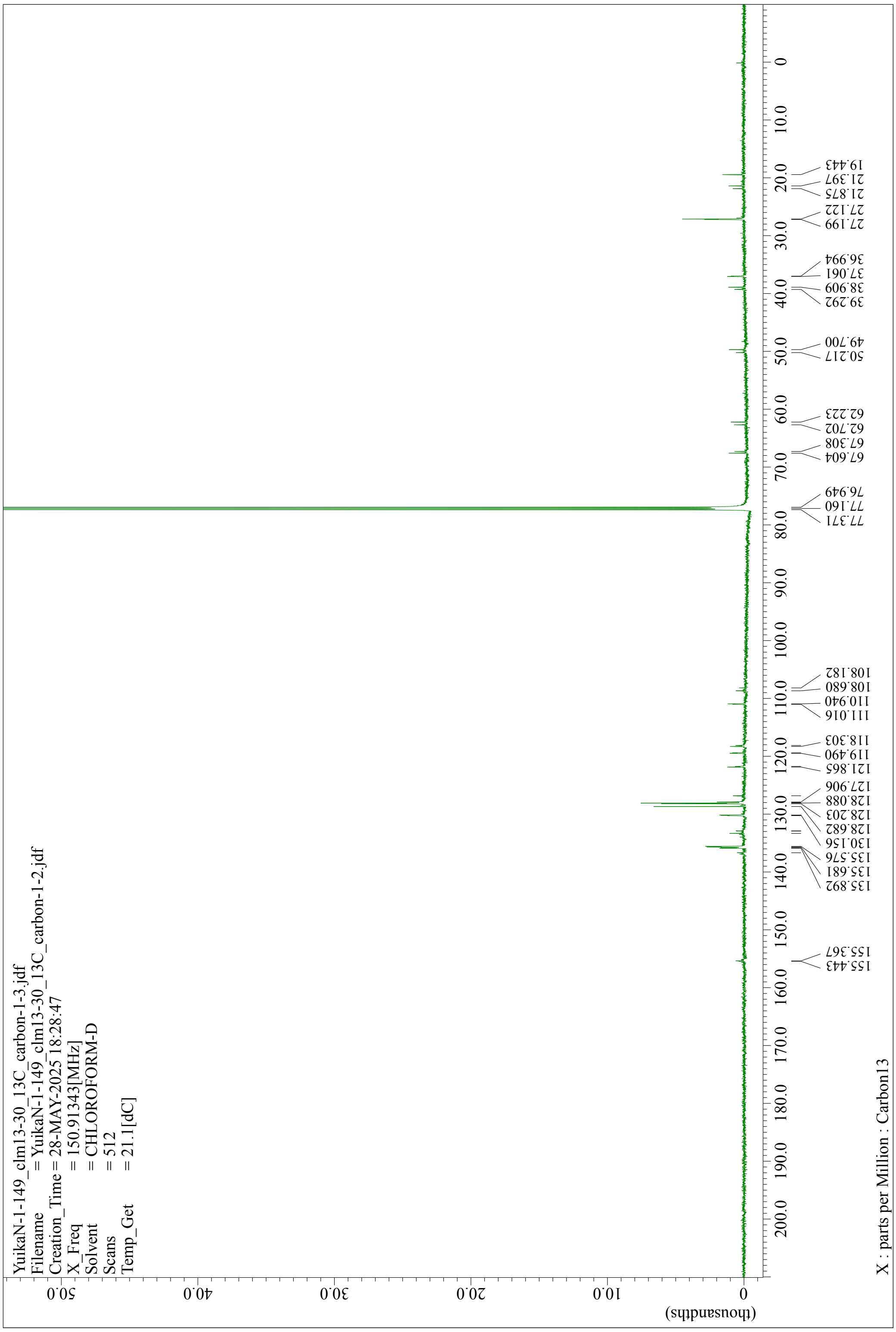

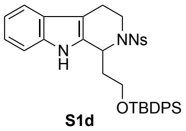

S1d

^{1}H NMR ($CDCl_3$, 600 MHz)

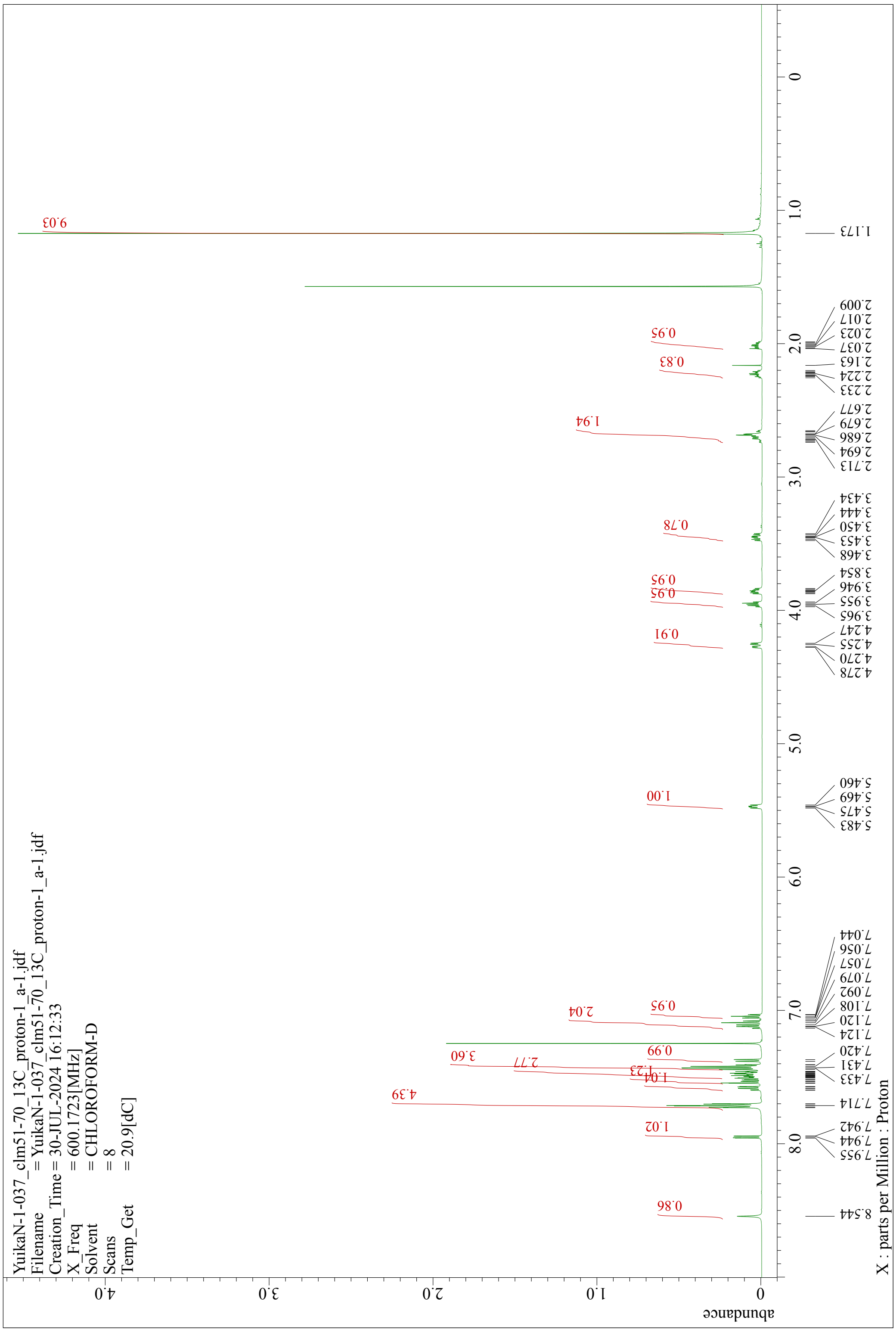

$^{13}C\{^{1}H\}$ NMR ($CDCl_3$, 150 MHz)

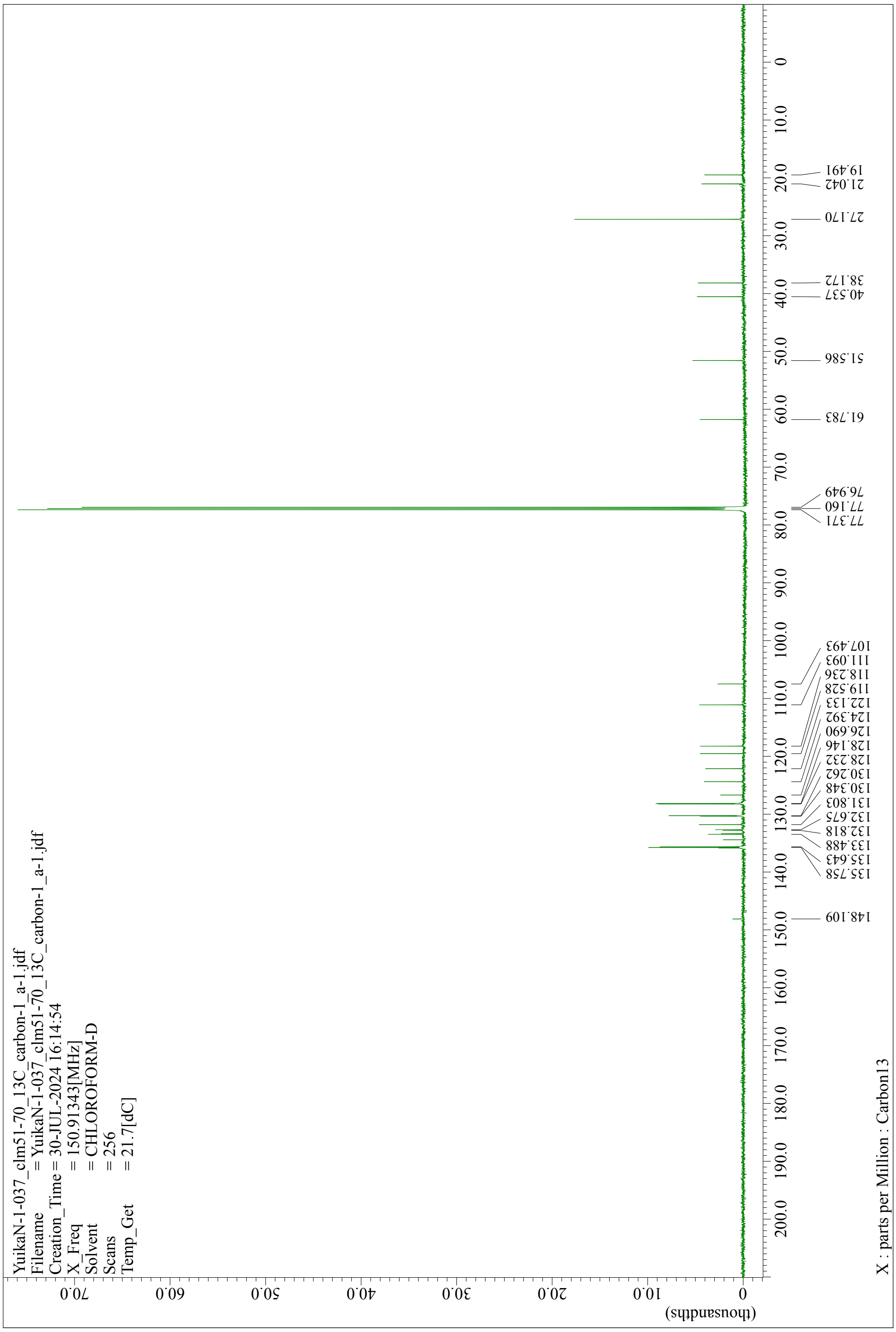

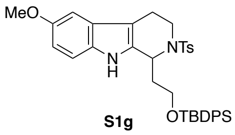

S1g

^{1}H NMR ($CDCl_3$, 600 MHz)

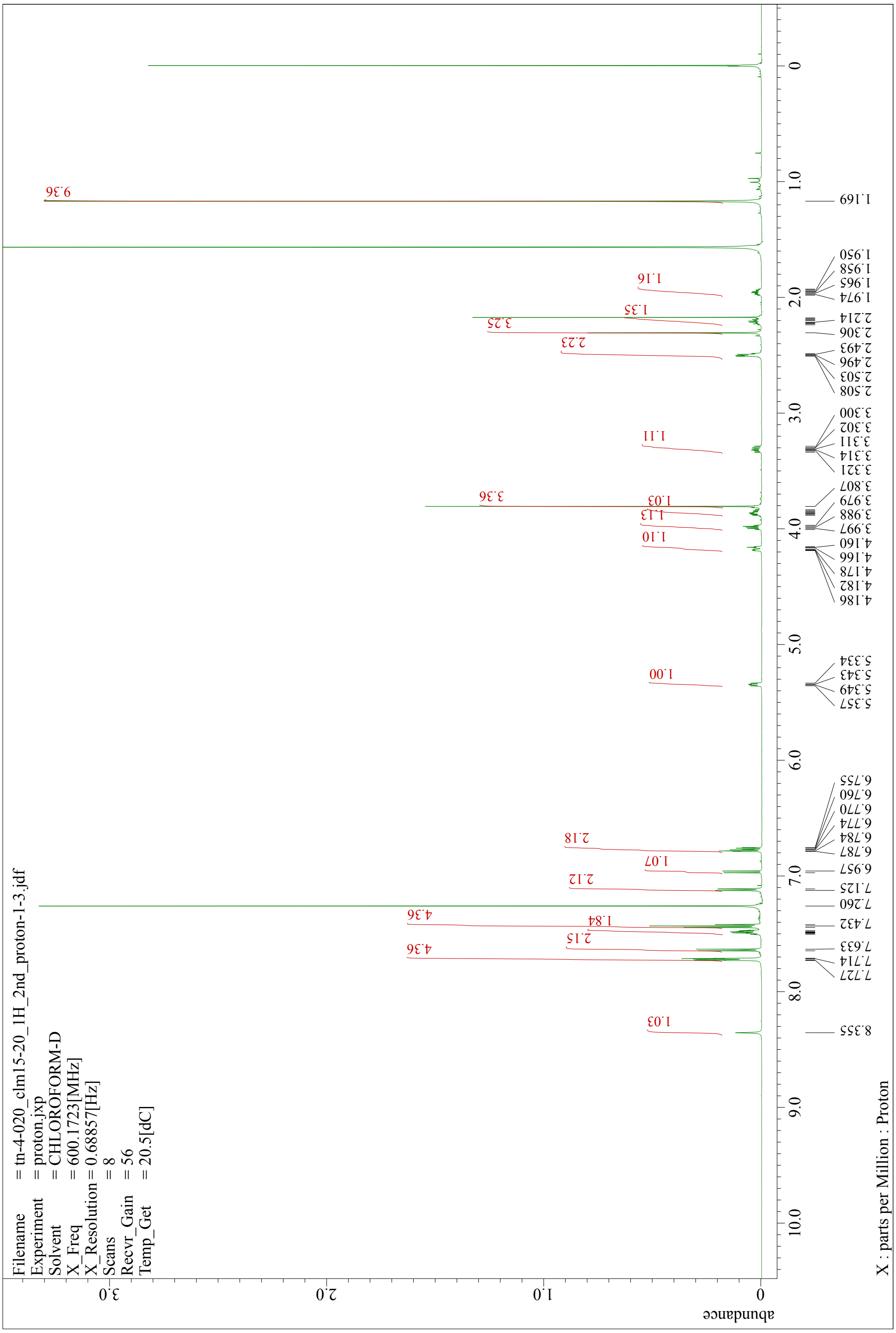

$^{13}C\{^{1}H\}$ NMR ($CDCl_3$, 150 MHz)

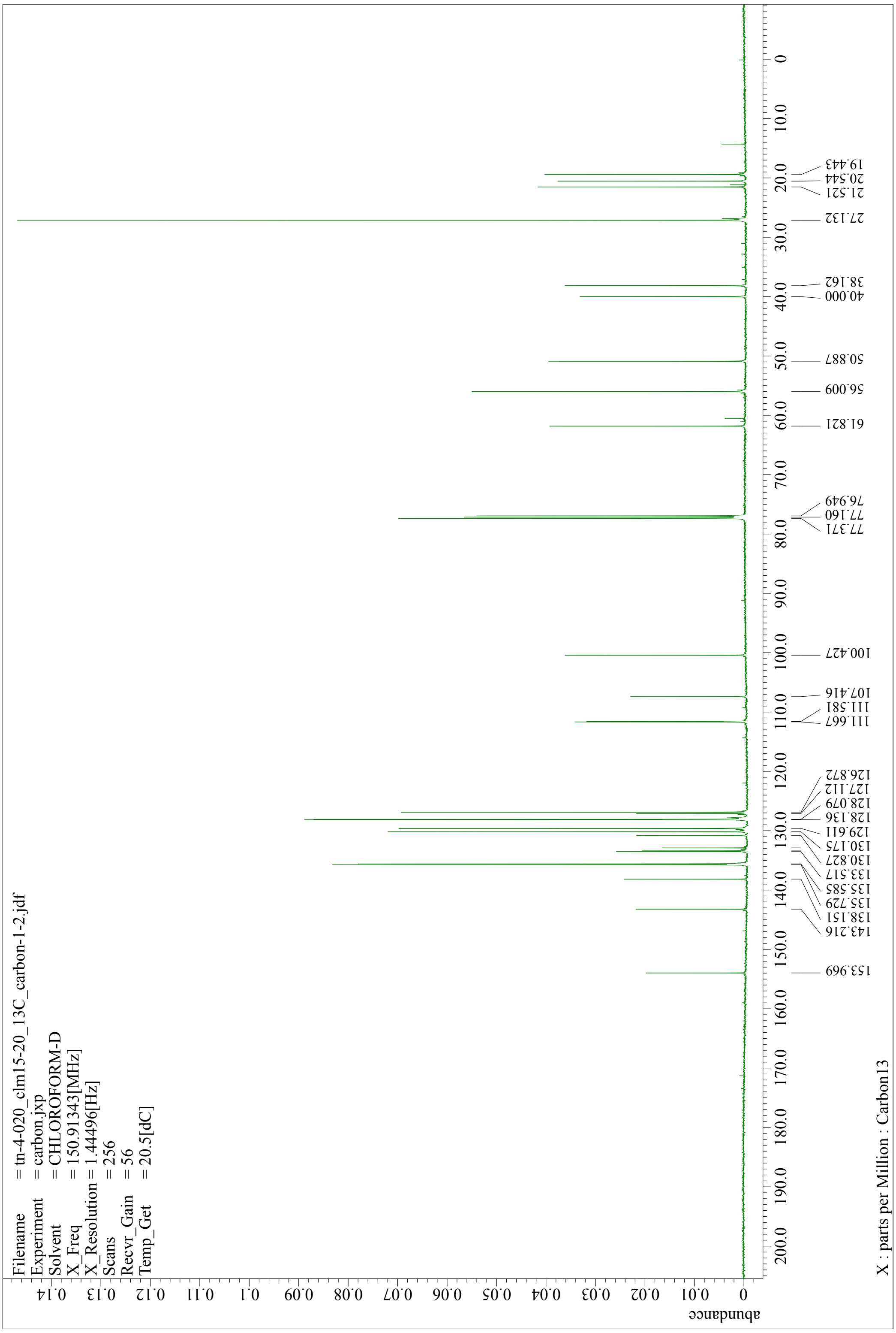

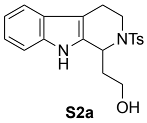

S2a

^{1}H NMR ($CDCl_3$, 600 MHz)

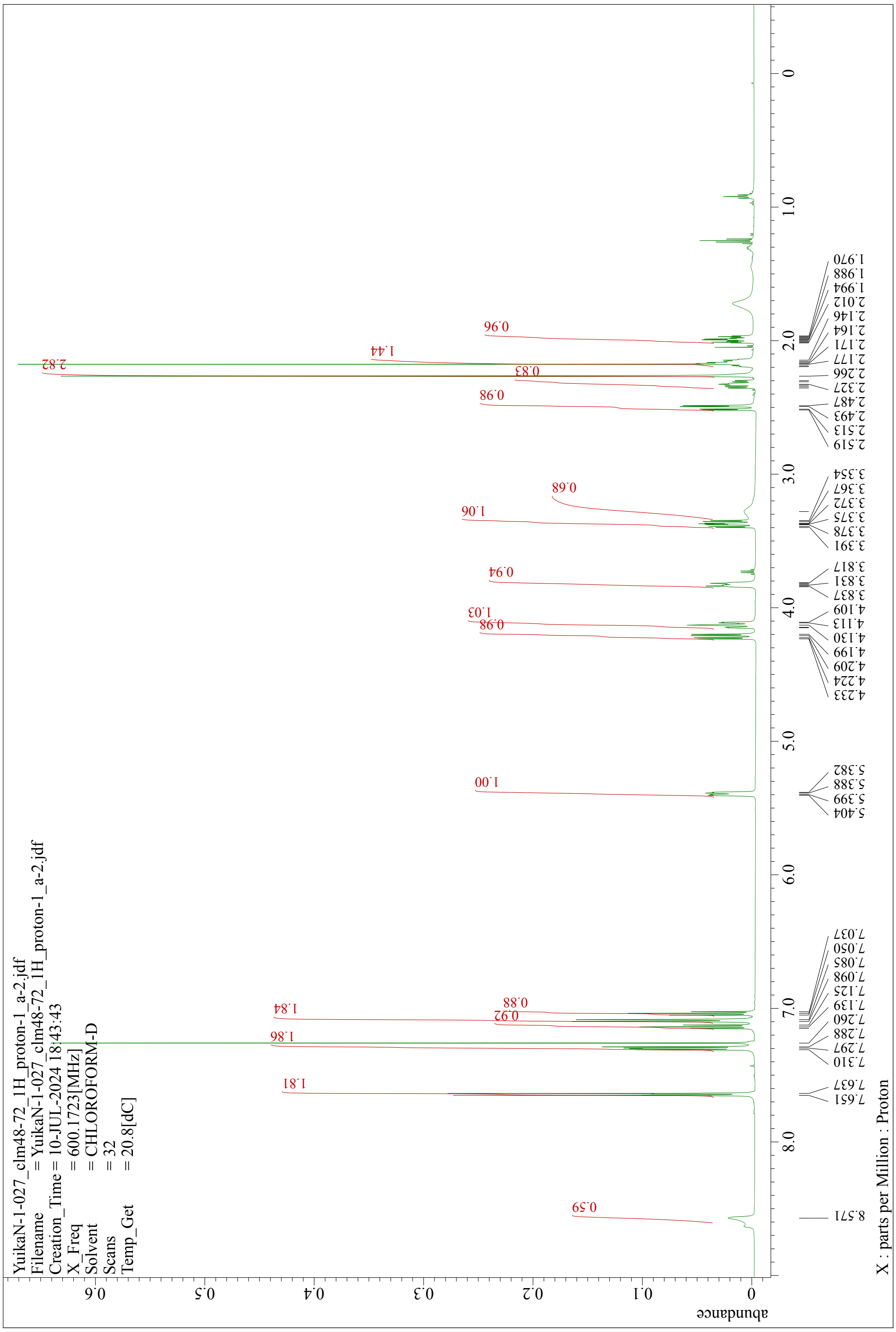

$^{13}C\{^1H\}$ NMR ($CDCl_3$, 150 MHz)

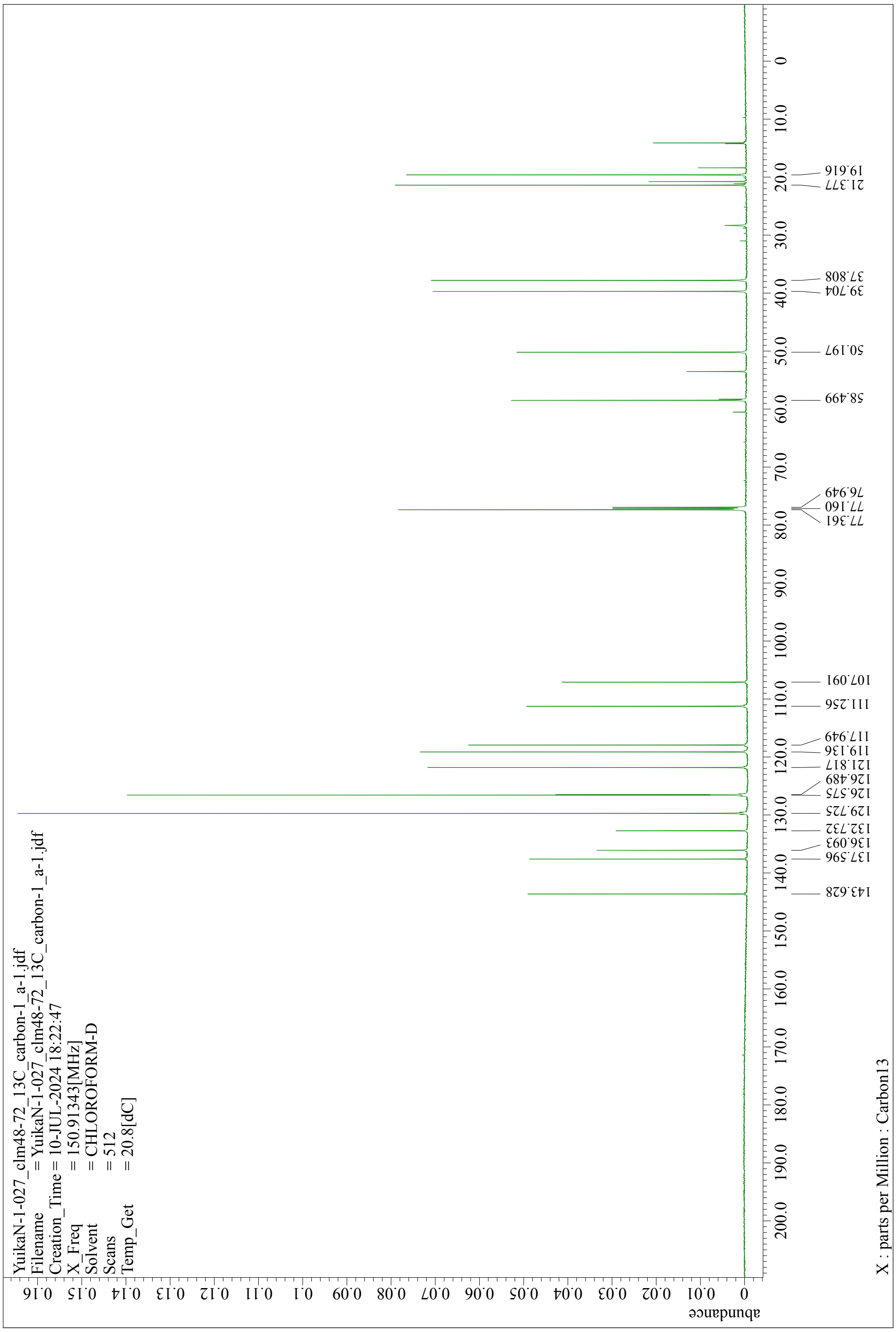

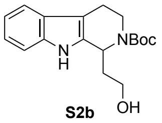

S2b

^{1}H NMR ($CDCl_3$, 600 MHz)

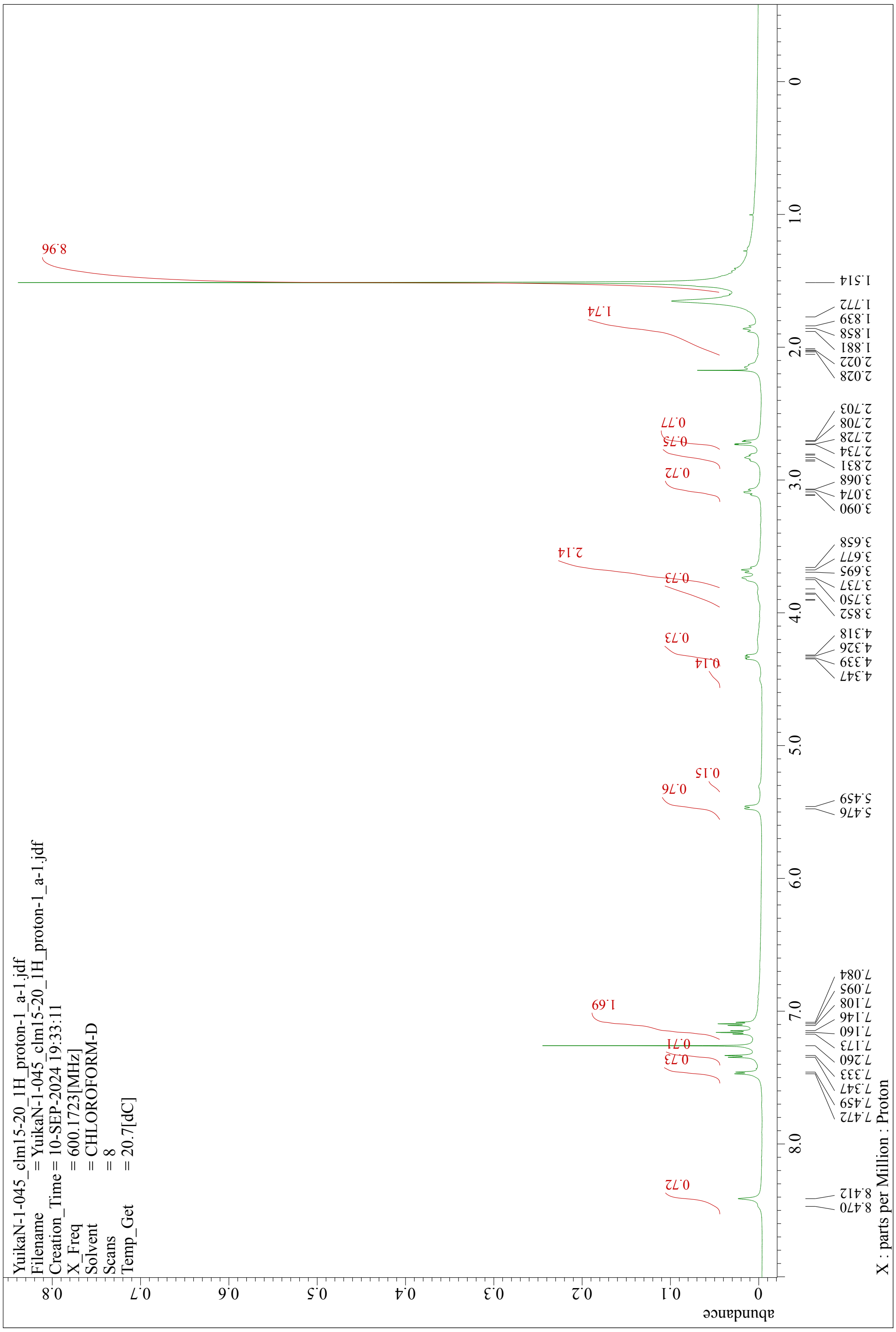

$^{13}C\{^1H\}$ NMR ($CDCl_3$, 150 MHz)

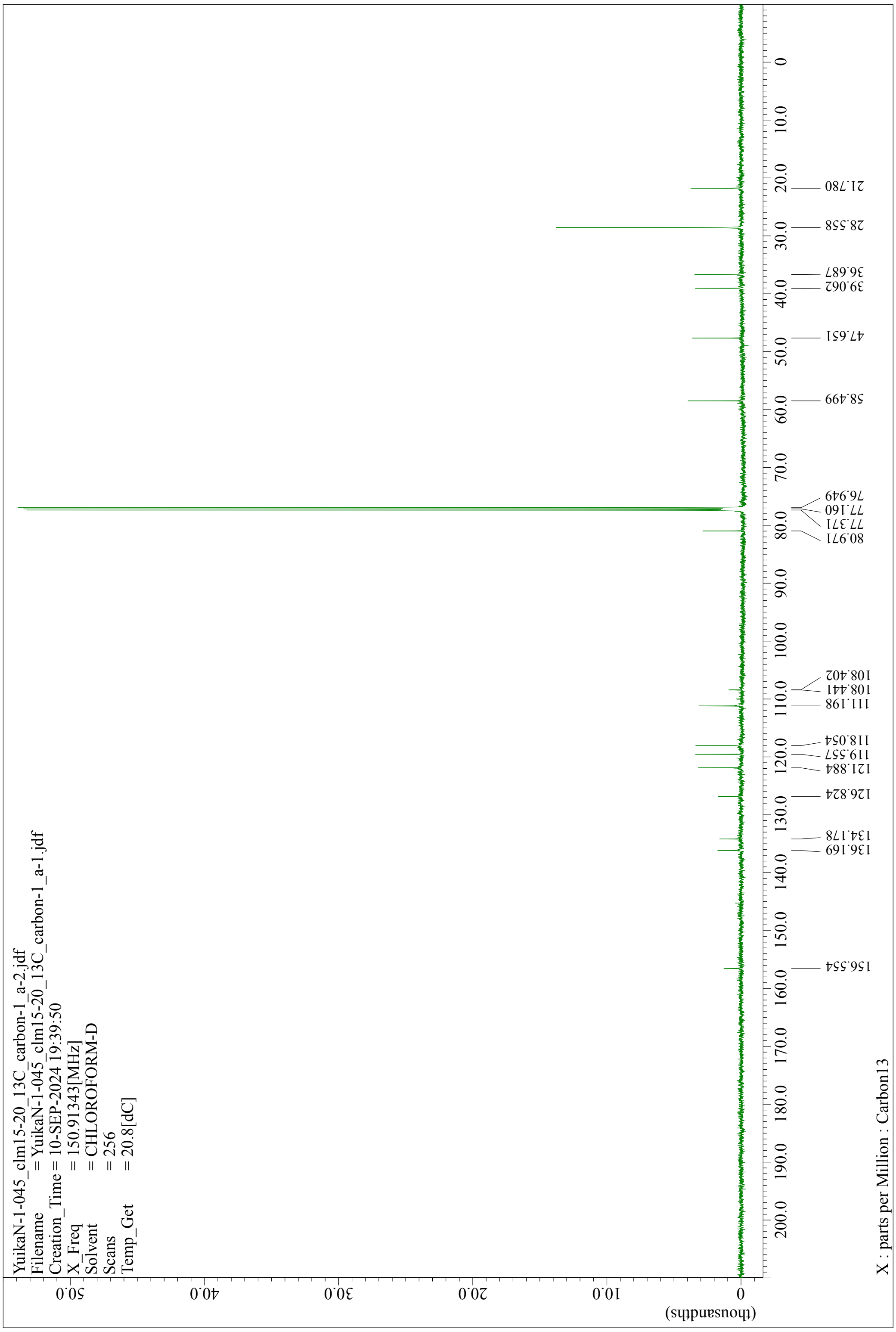

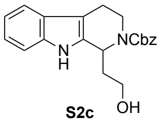

S2c

^{1}H NMR ($CDCl_3$, 600 MHz)

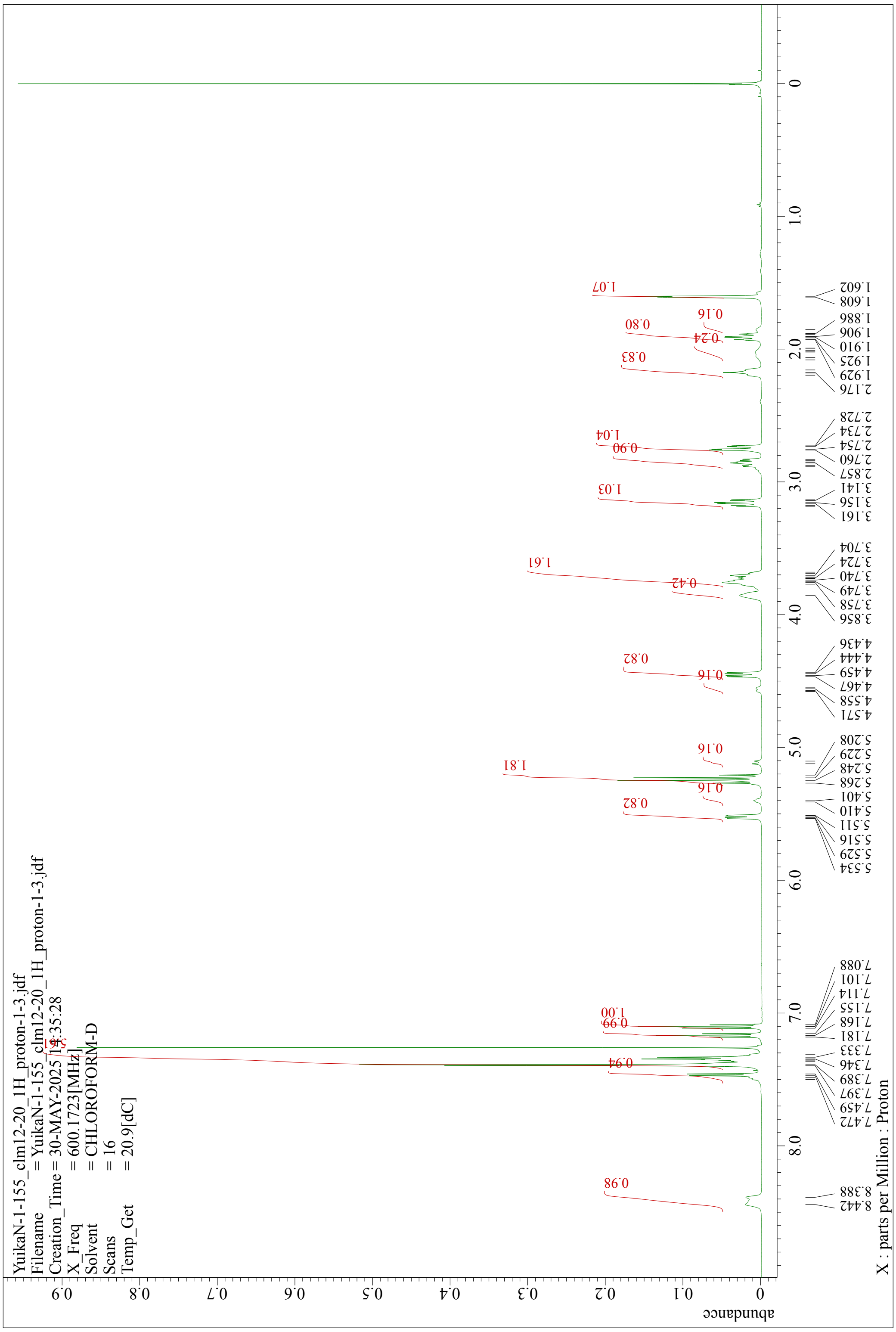

$^{13}C\{^{1}H\}$ NMR ($CDCl_3$, 150 MHz)

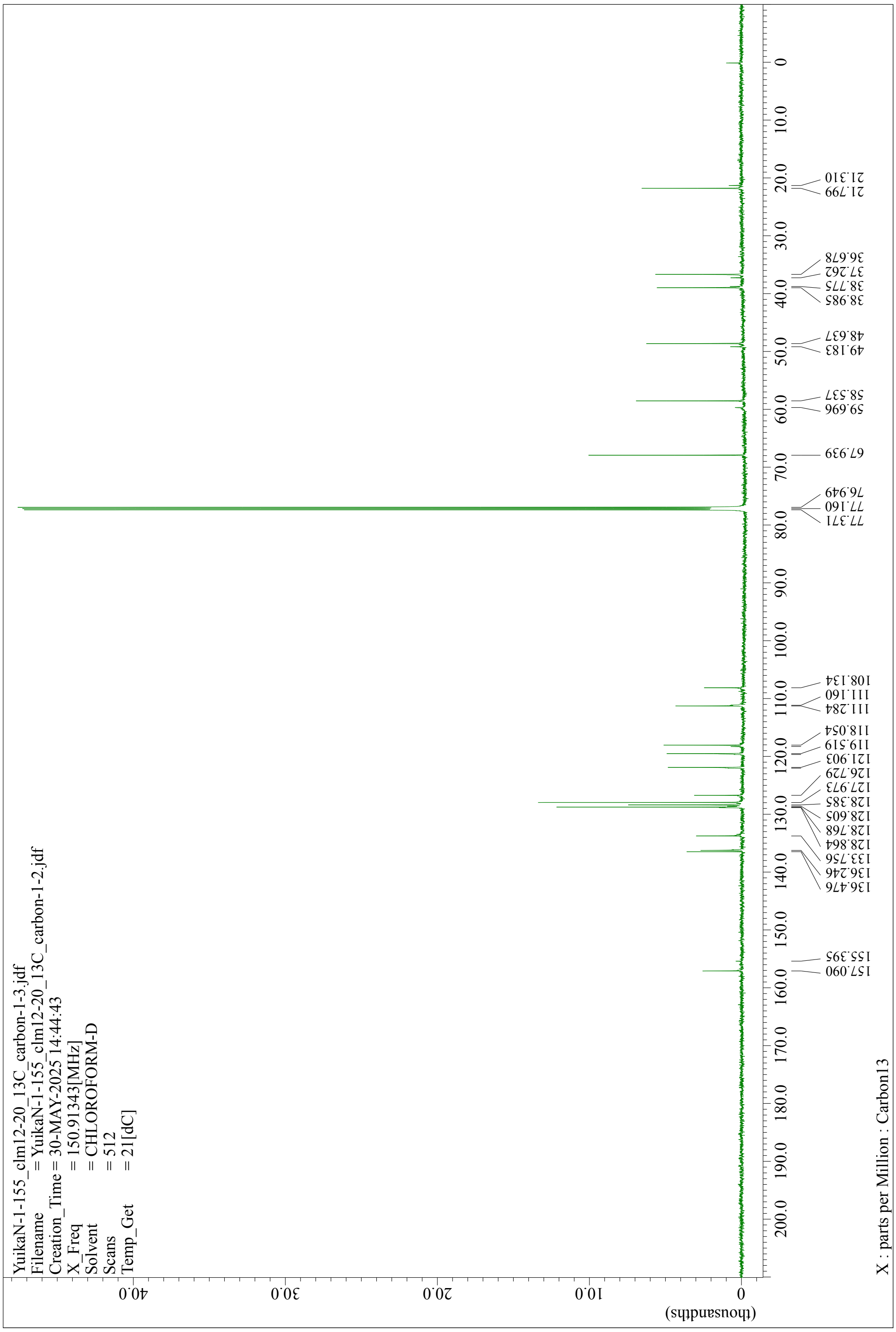

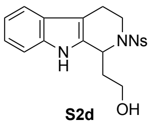

S2d

^{1}H NMR ($CDCl_3$, 600 MHz)

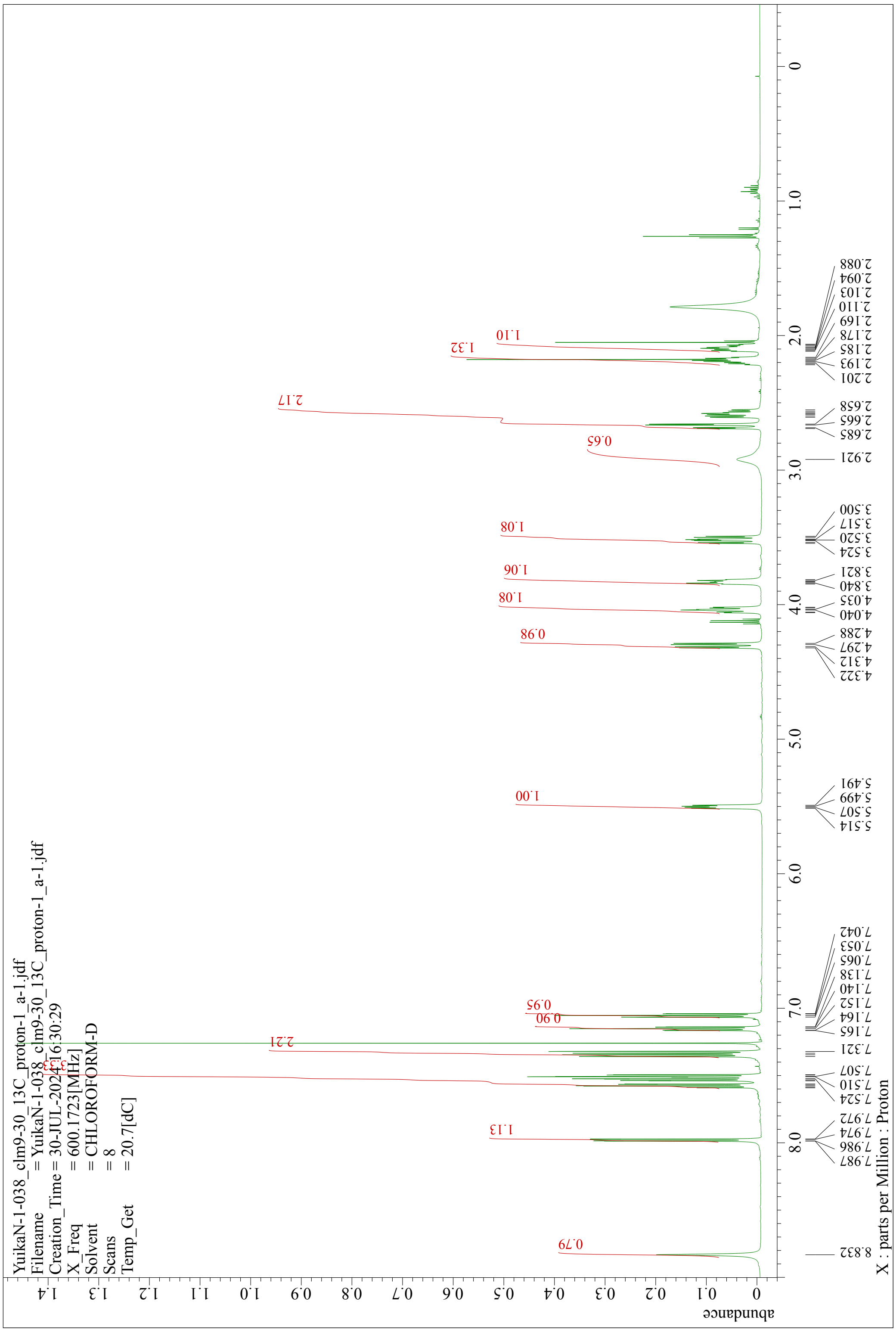

$^{13}C\{^{1}H\}$ NMR ($CDCl_3$, 150 MHz)

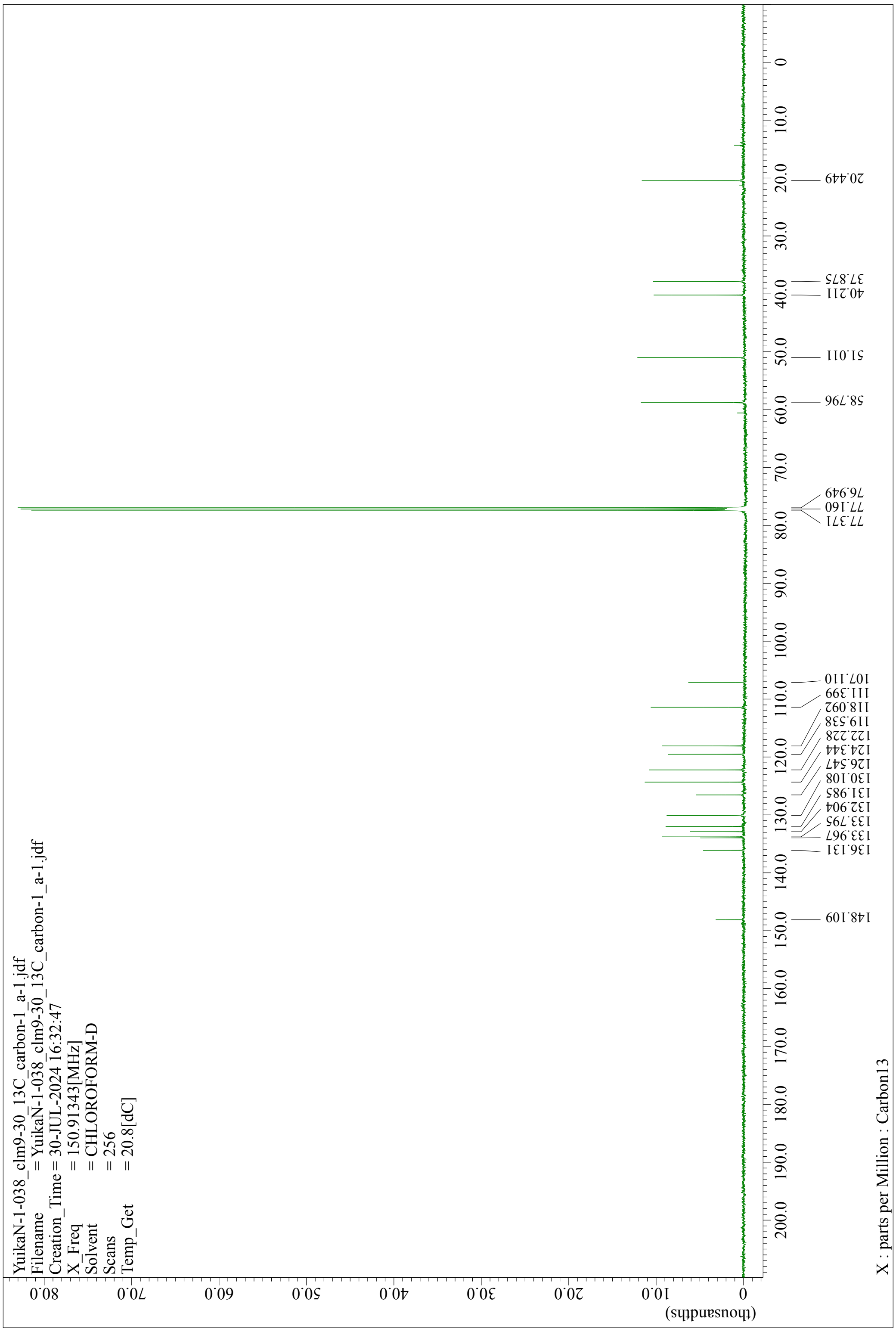

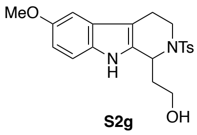

S2g

^{1}H NMR ($CDCl_3$, 600 MHz)

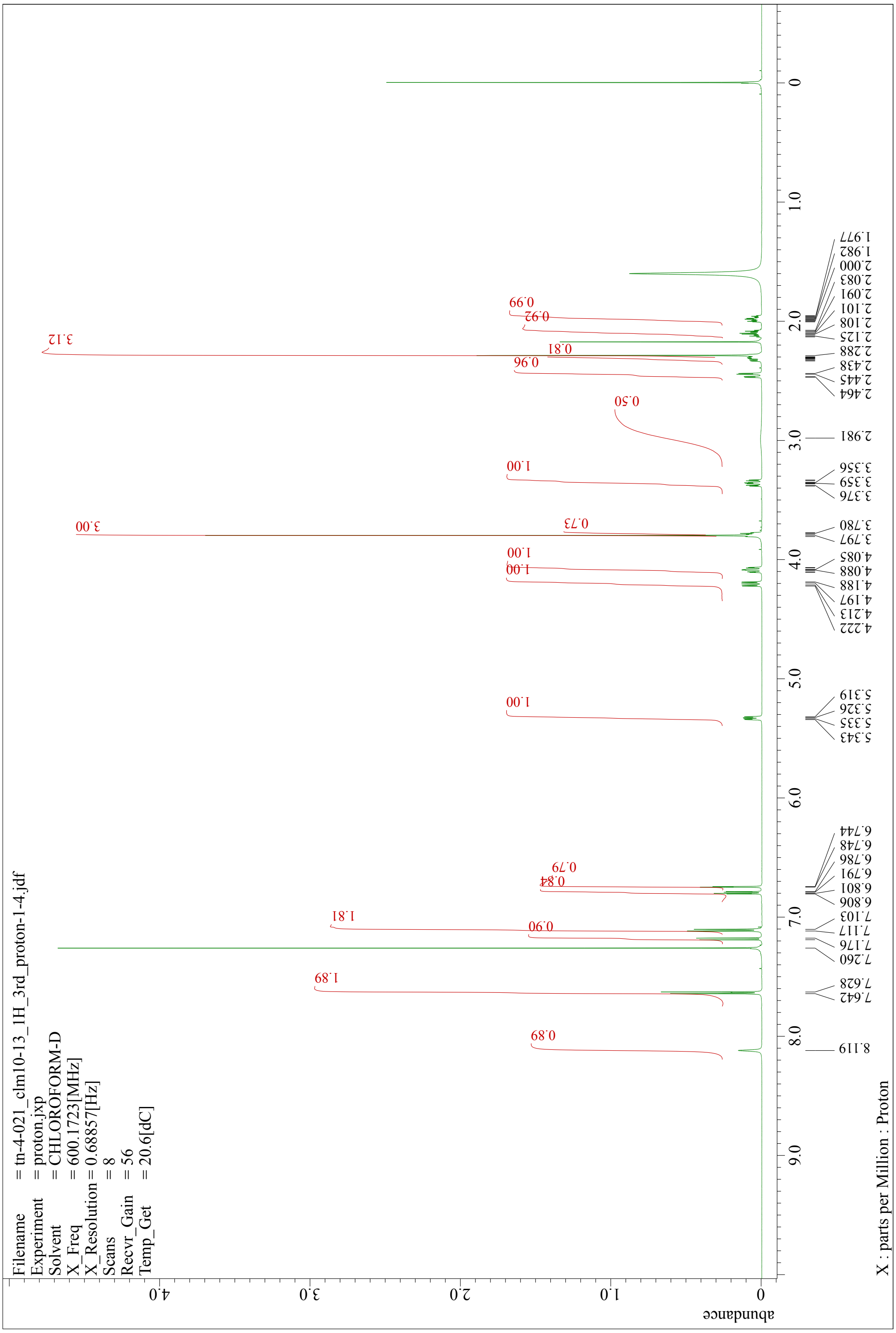

$^{13}C\{^{1}H\}$ NMR ($CDCl_3$, 150 MHz)

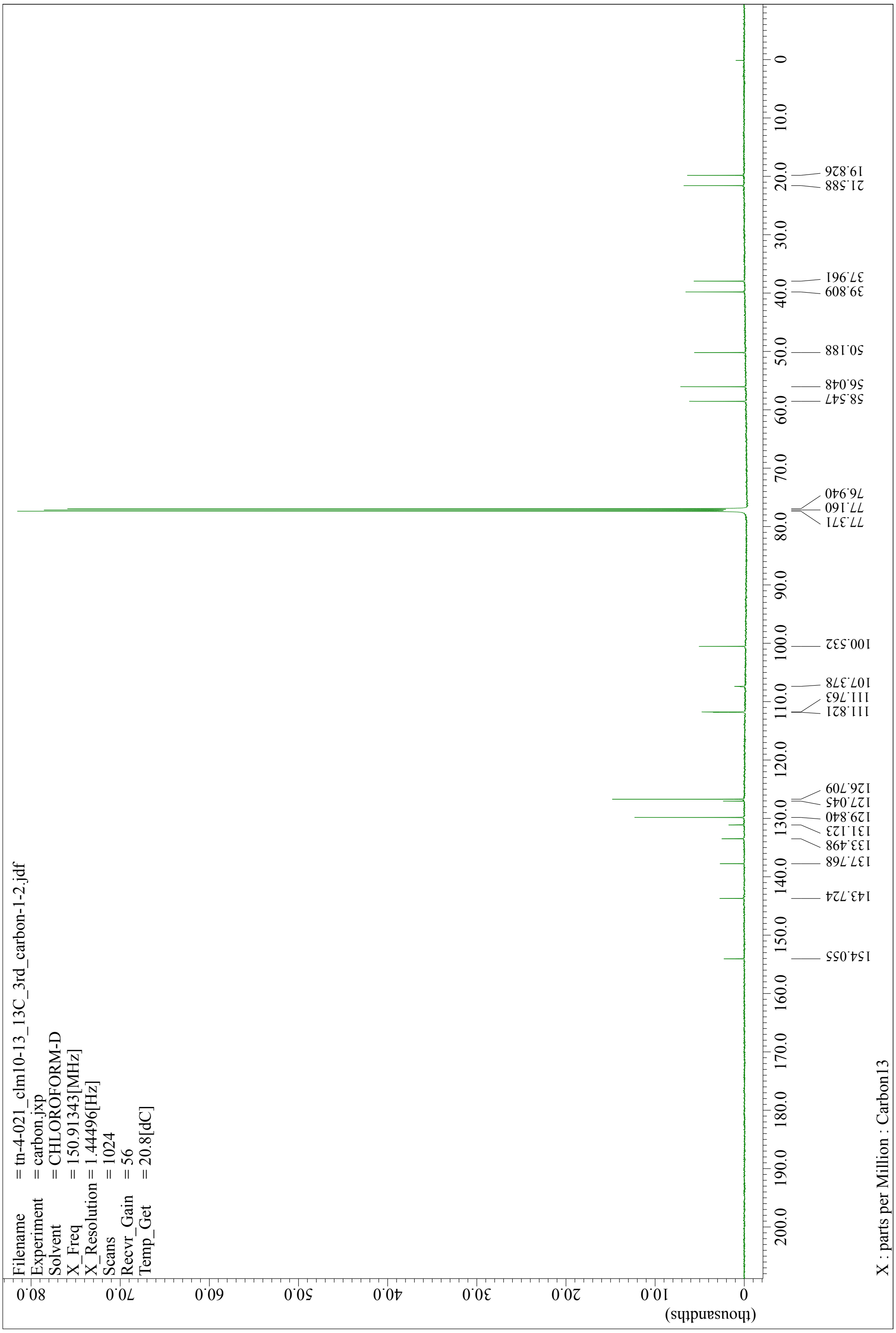

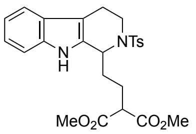

3a

^{1}H NMR ($CDCl_3$, 600 MHz)

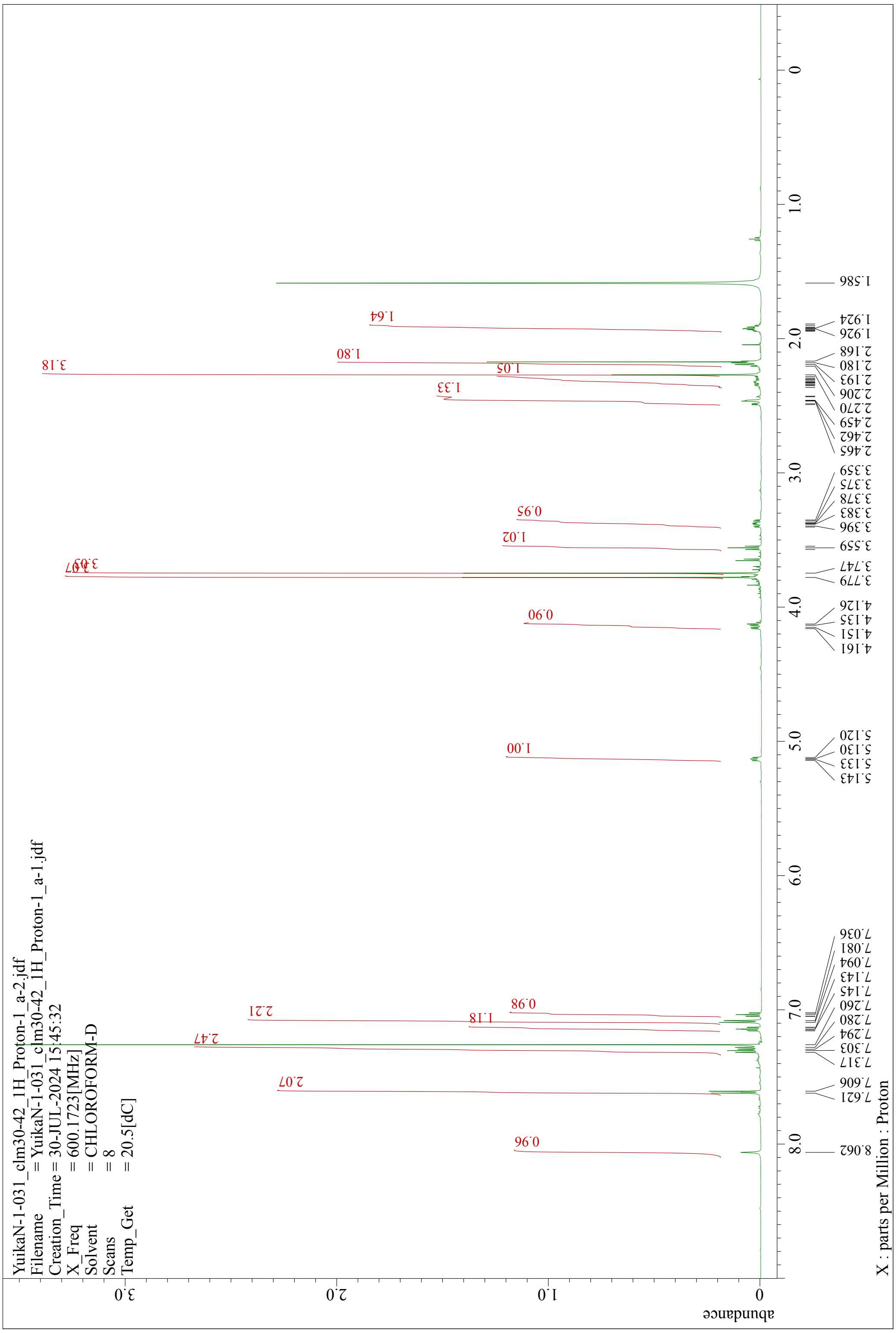

$^{13}C\{^{1}H\}$ NMR ($CDCl_3$, 150 MHz)

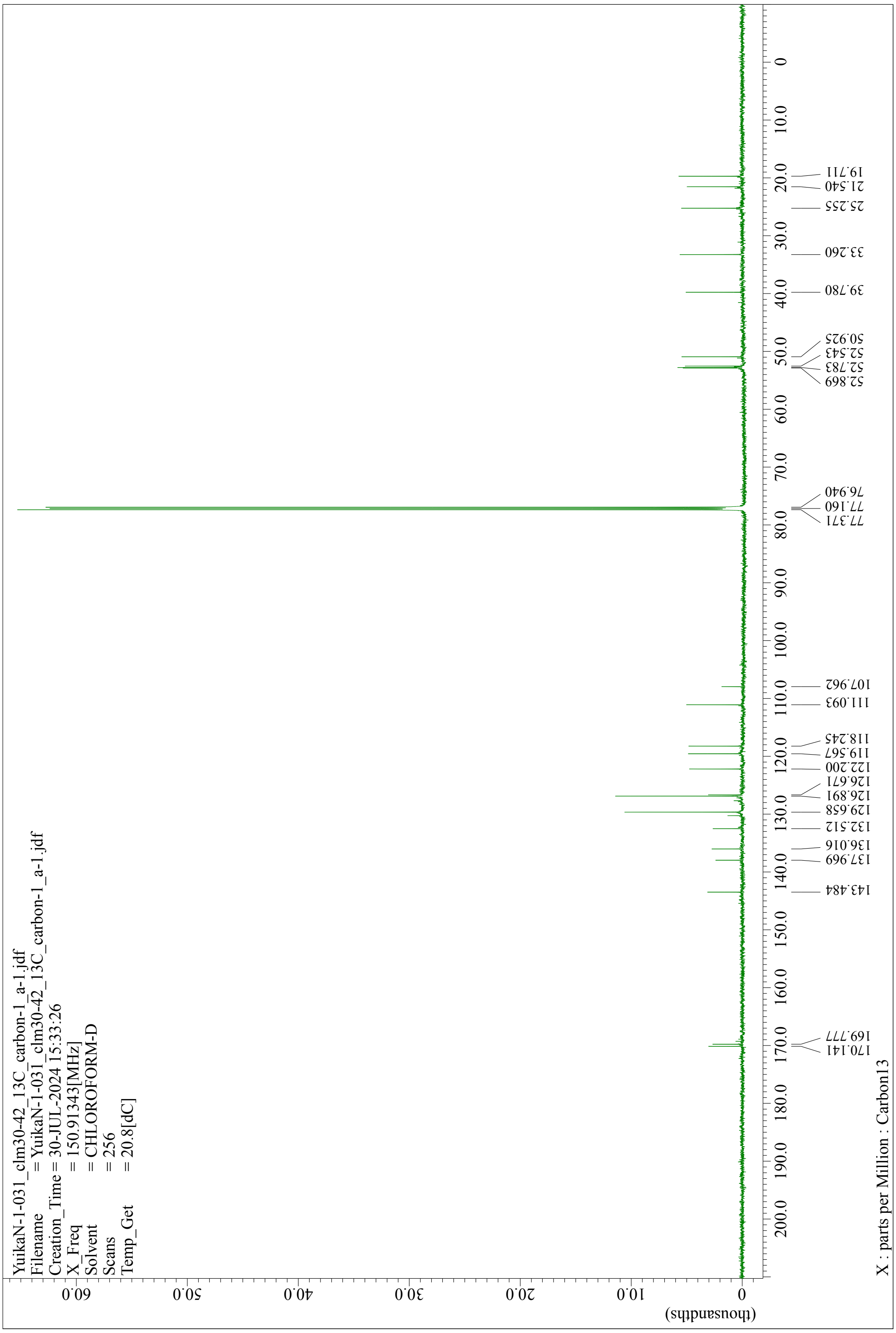

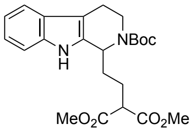

3b

^{1}H NMR ($CDCl_3$, 600 MHz)

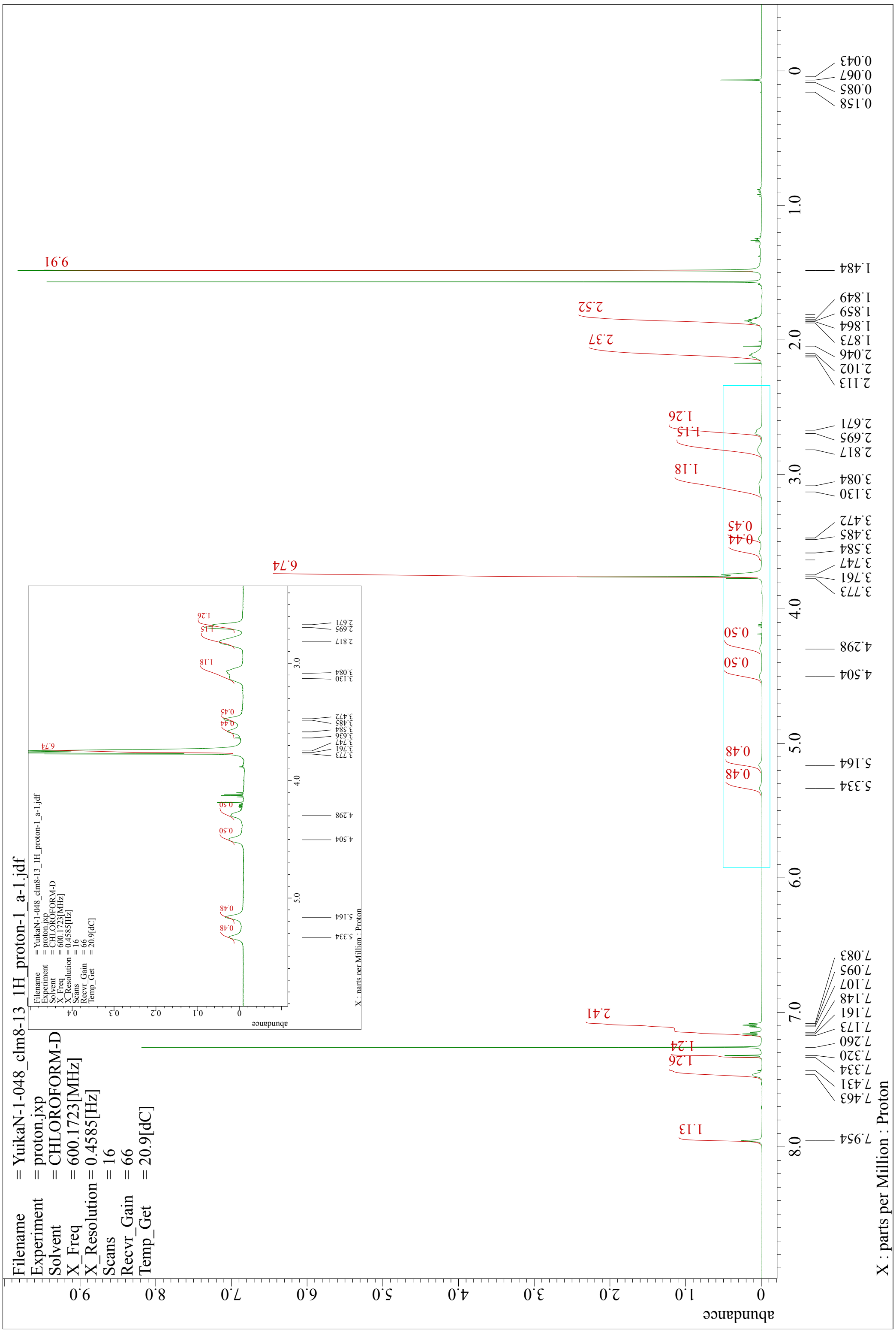

$^{13}C\{^1H\}$ NMR ($CDCl_3$, 150 MHz)

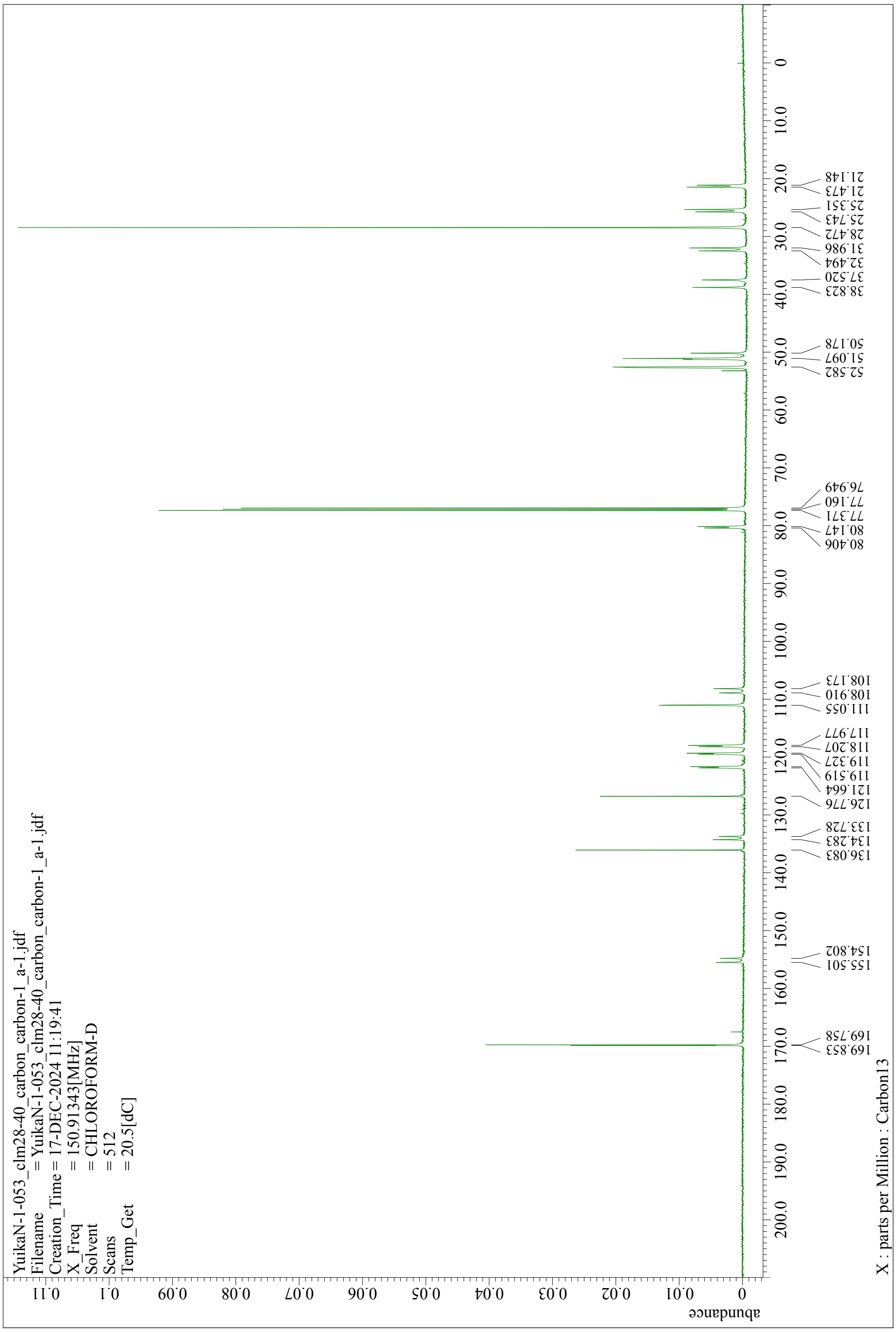

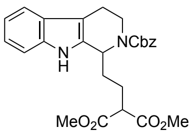

3c

^{1}H NMR ($CDCl_3$, 600 MHz)

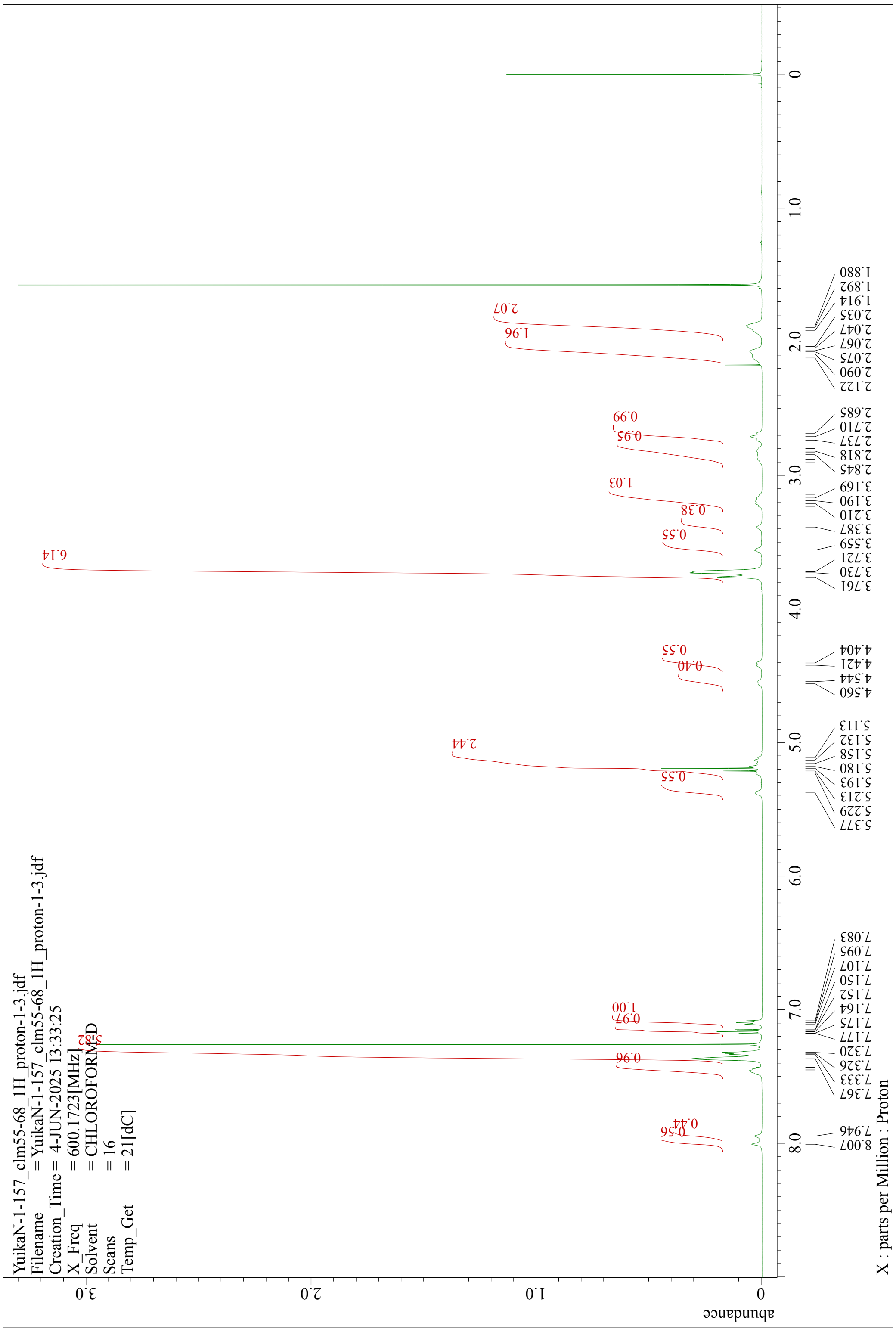

$^{13}C\{^1H\}$ NMR ($CDCl_3$, 150 MHz)

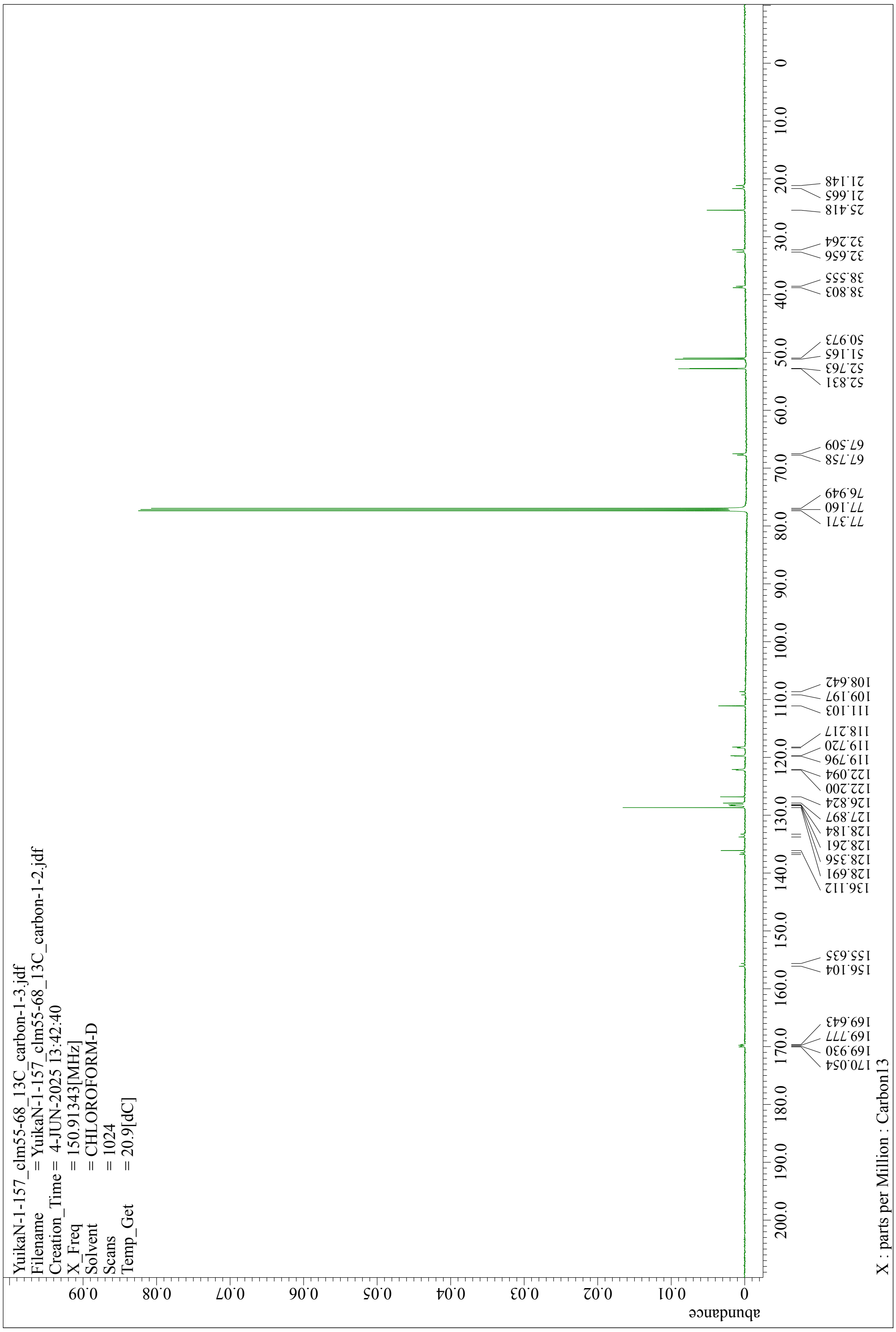

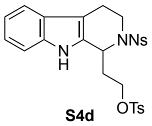

S4d

^{1}H NMR (CDCl$_3$, 600 MHz)

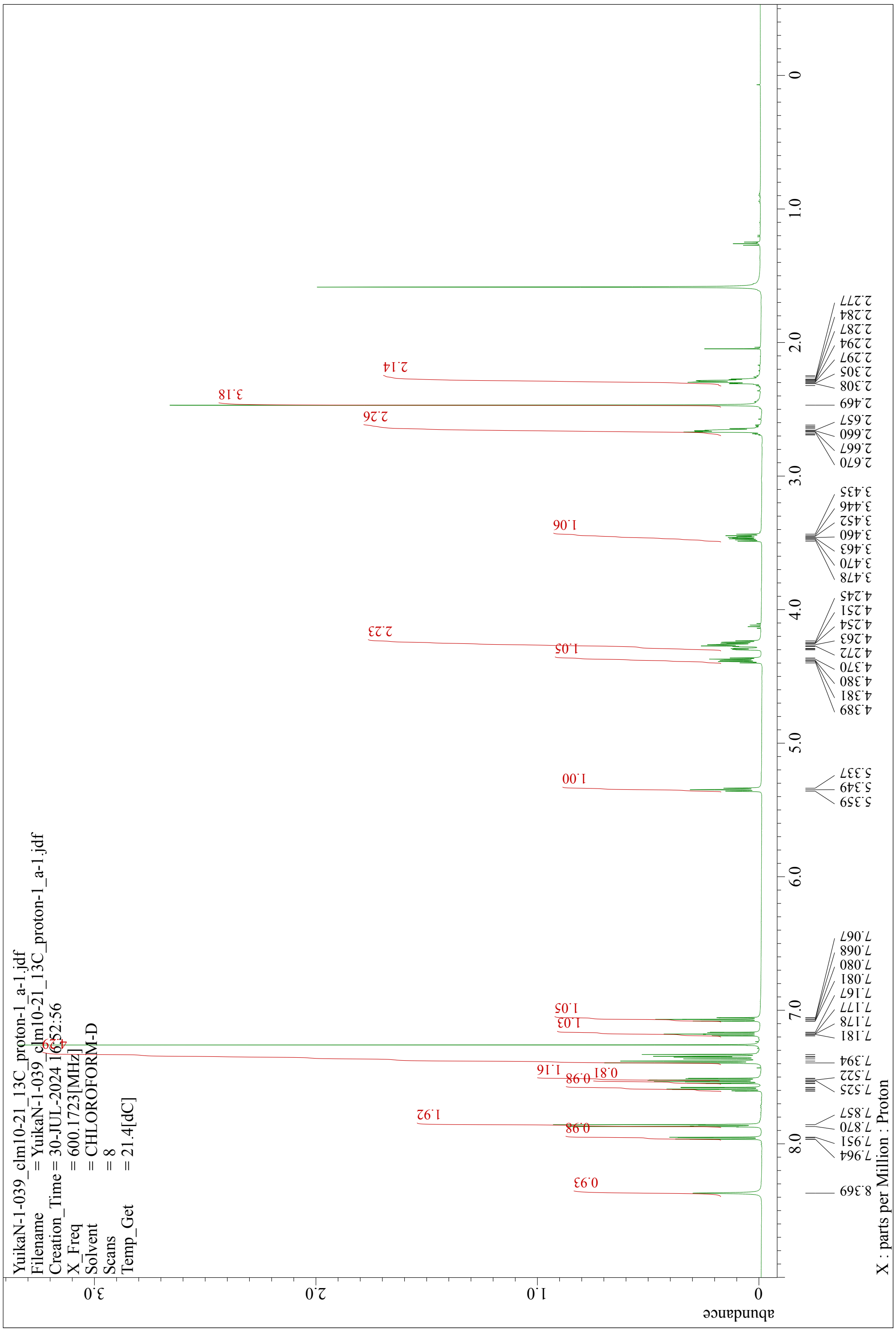

$^{13}C\{^{1}H\}$ NMR ($CDCl_3$, 150 MHz)

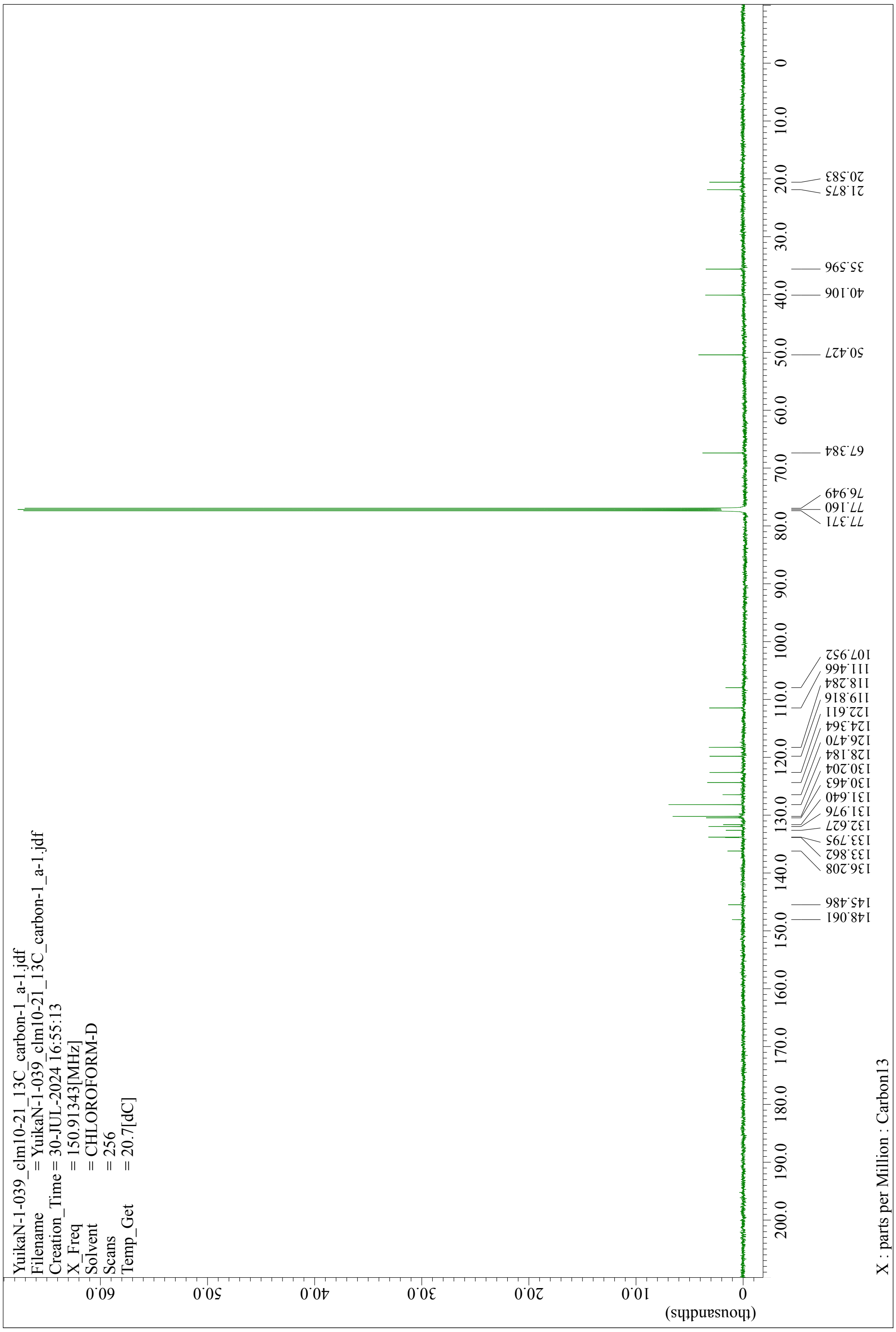

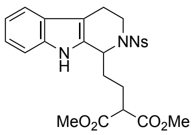

3d

^{1}H NMR ($CDCl_3$, 600 MHz)

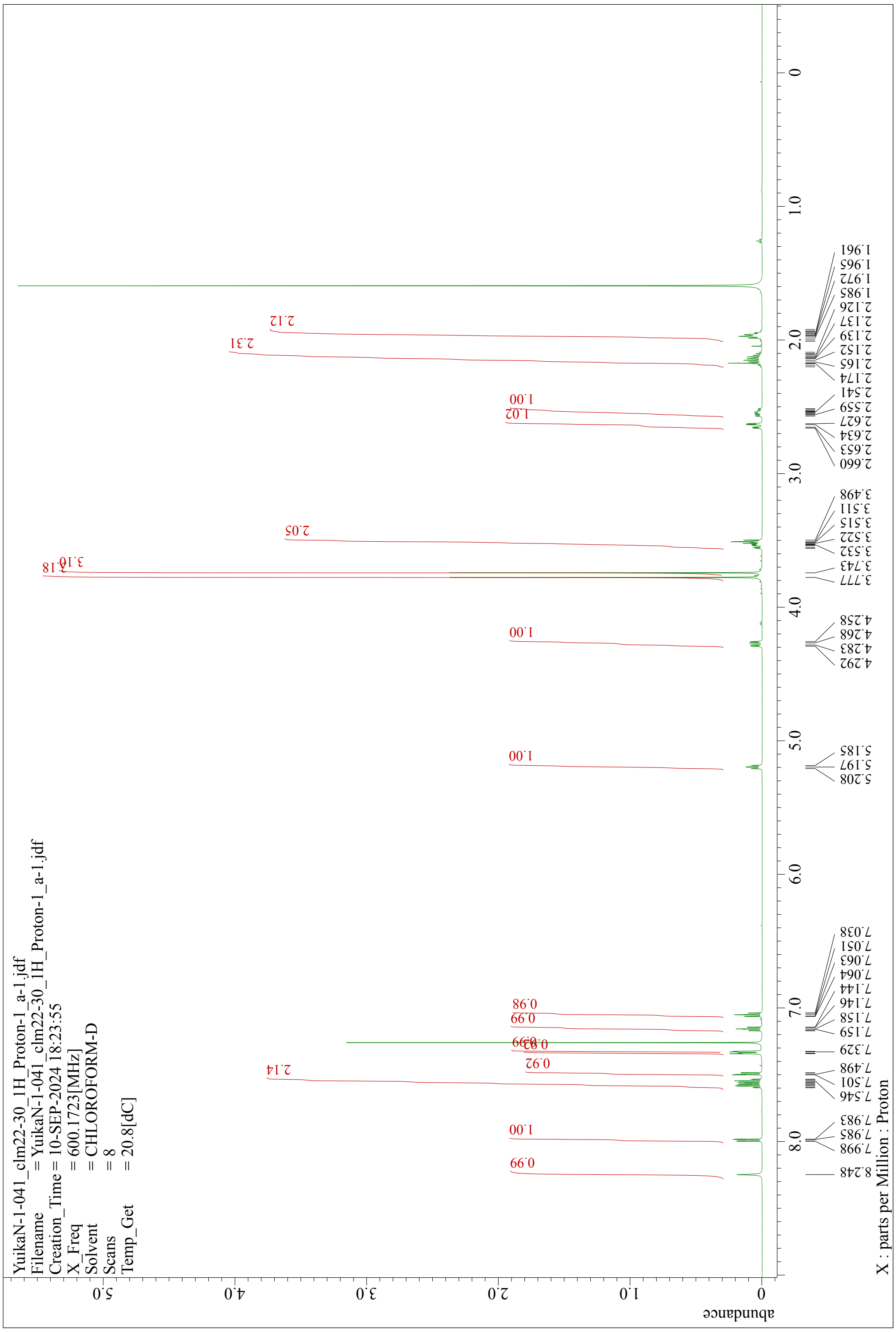

$^{13}C\{^1H\}$ NMR ($CDCl_3$, 150 MHz)

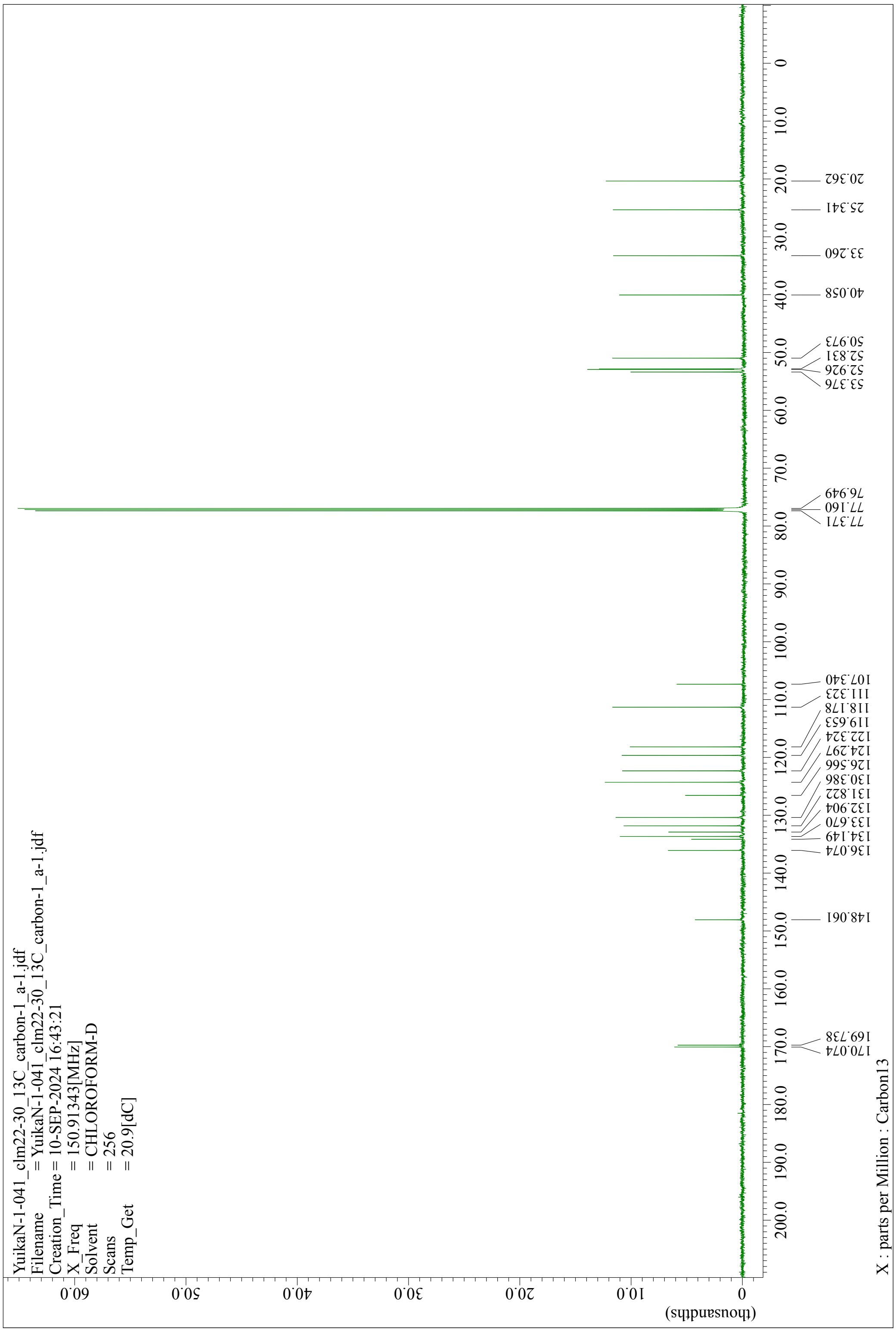

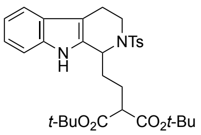

3e

^{1}H NMR ($CDCl_3$, 600 MHz)

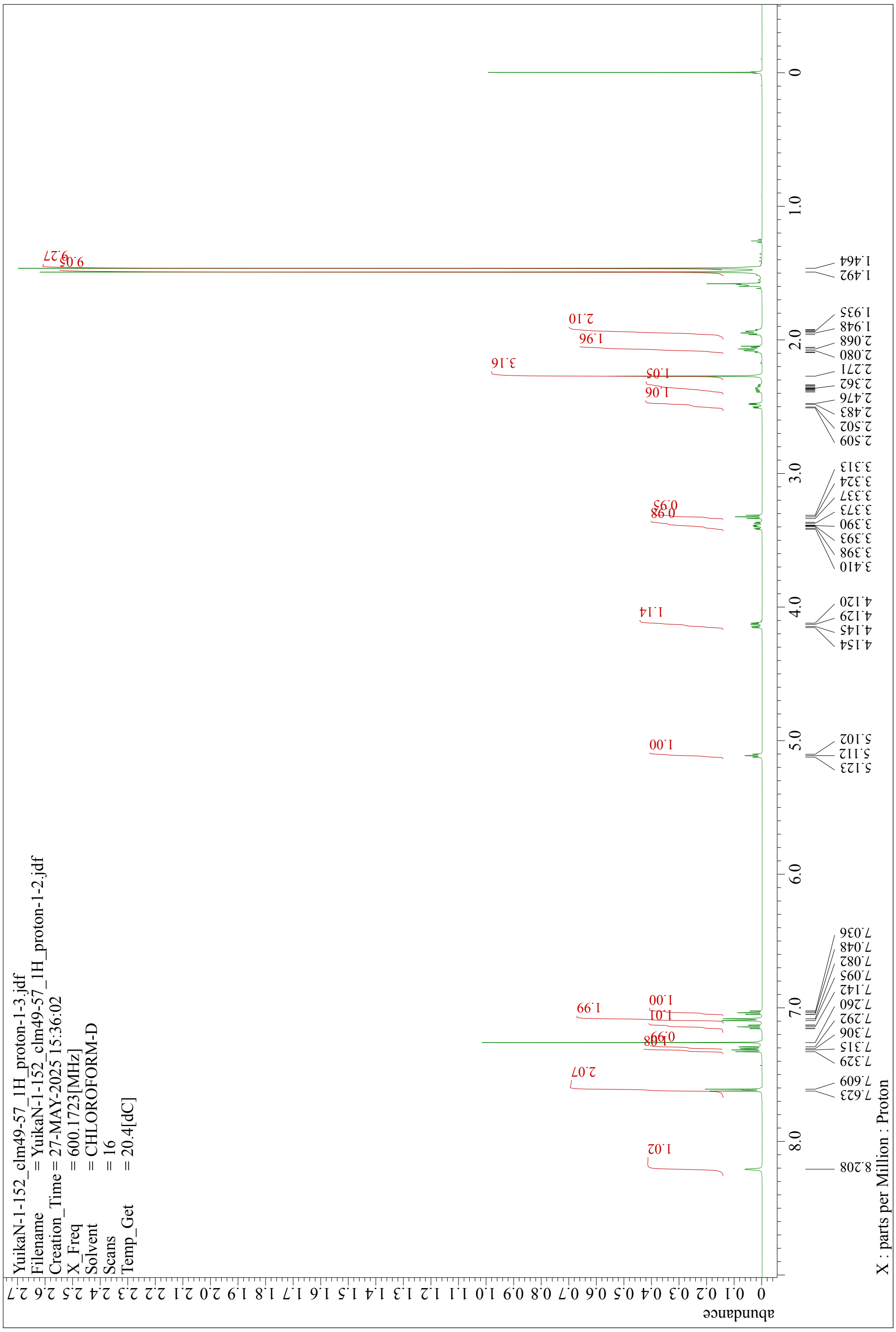

$^{13}C\{^1H\}$ NMR ($CDCl_3$, 150 MHz)

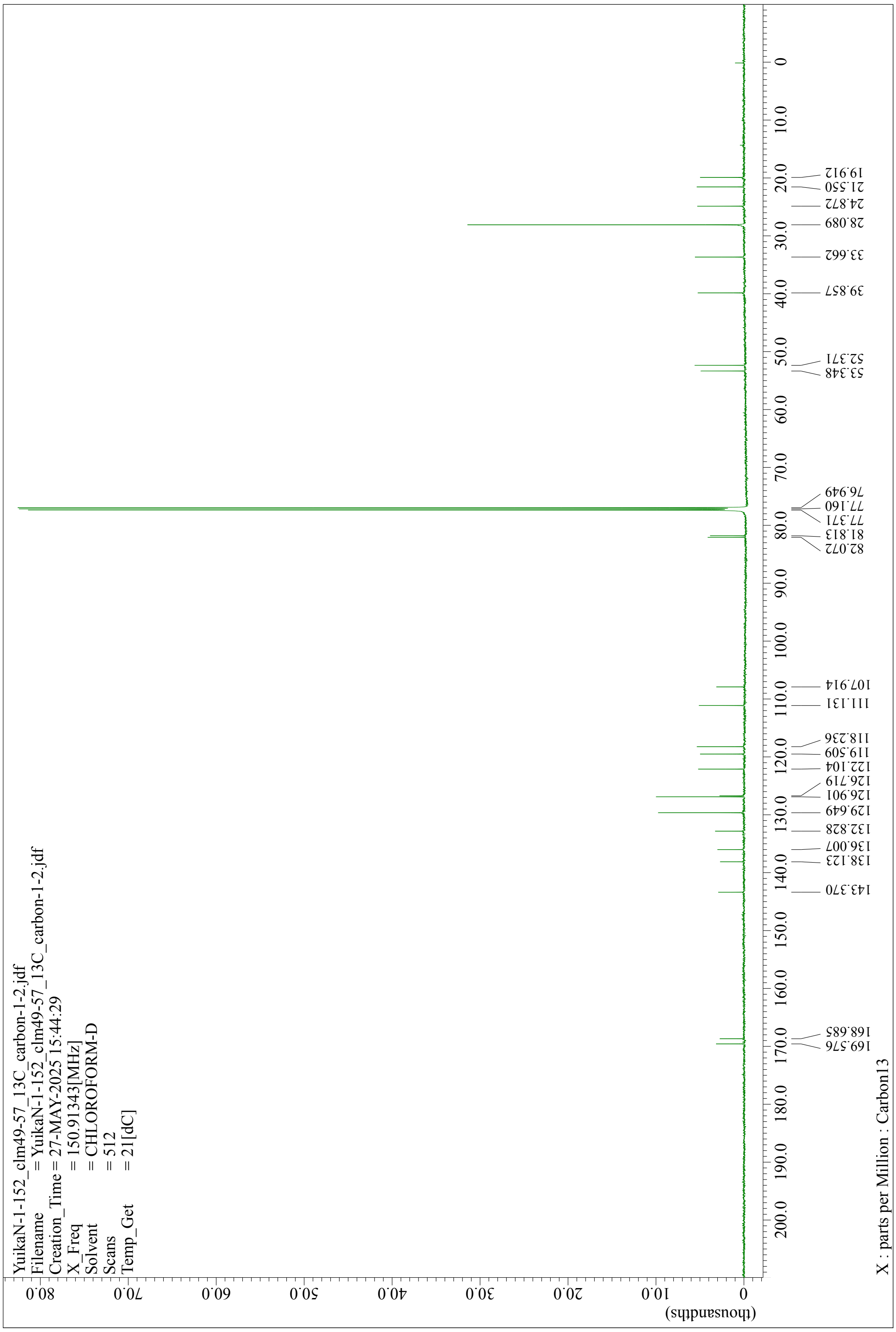

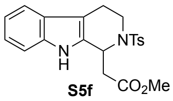

S5f

^{1}H NMR ($CDCl_3$, 600 MHz)

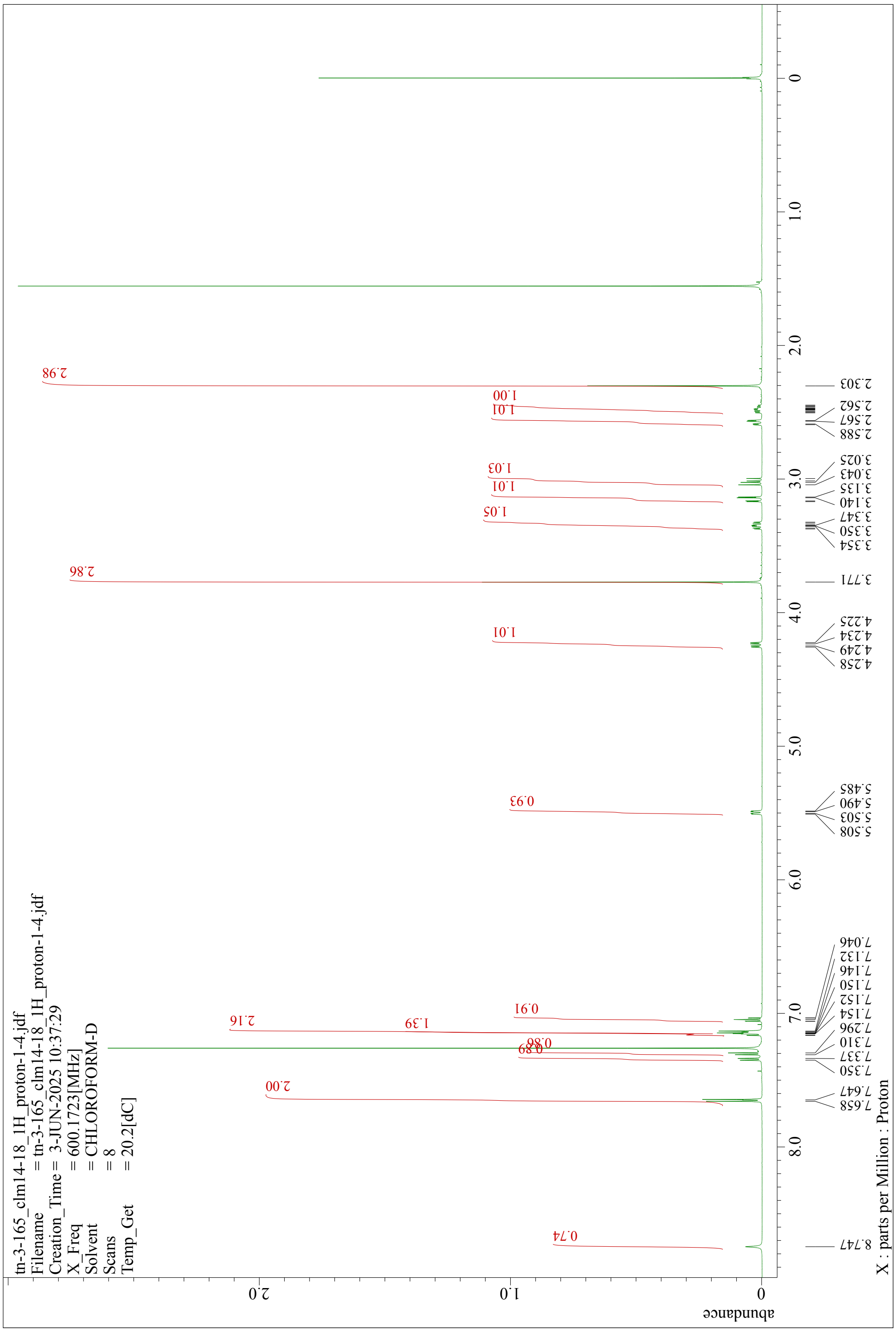

$^{13}C\{^1H\}$ NMR ($CDCl_3$, 150 MHz)

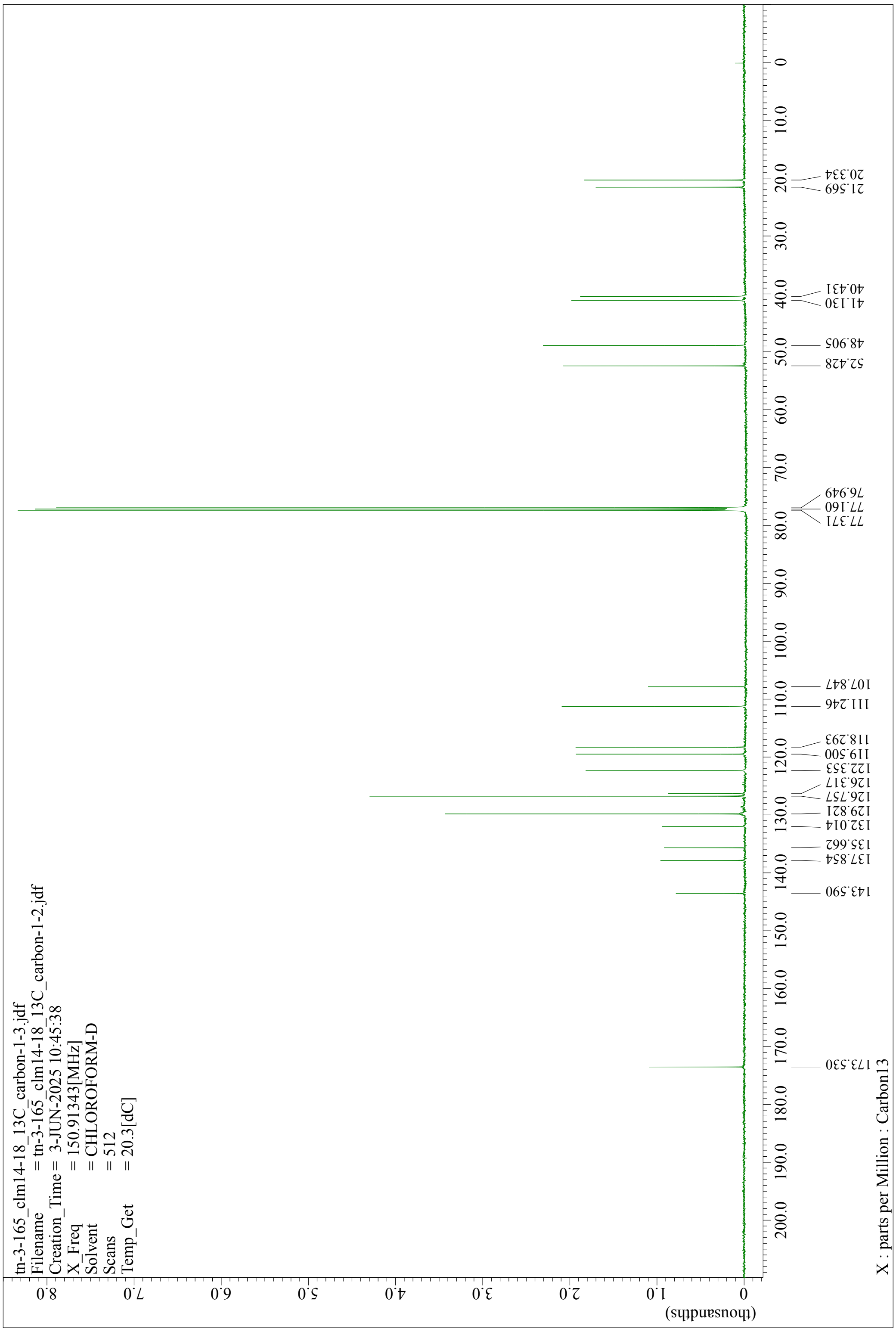

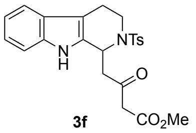

3f

^{1}H NMR ($CDCl_3$, 600 MHz)

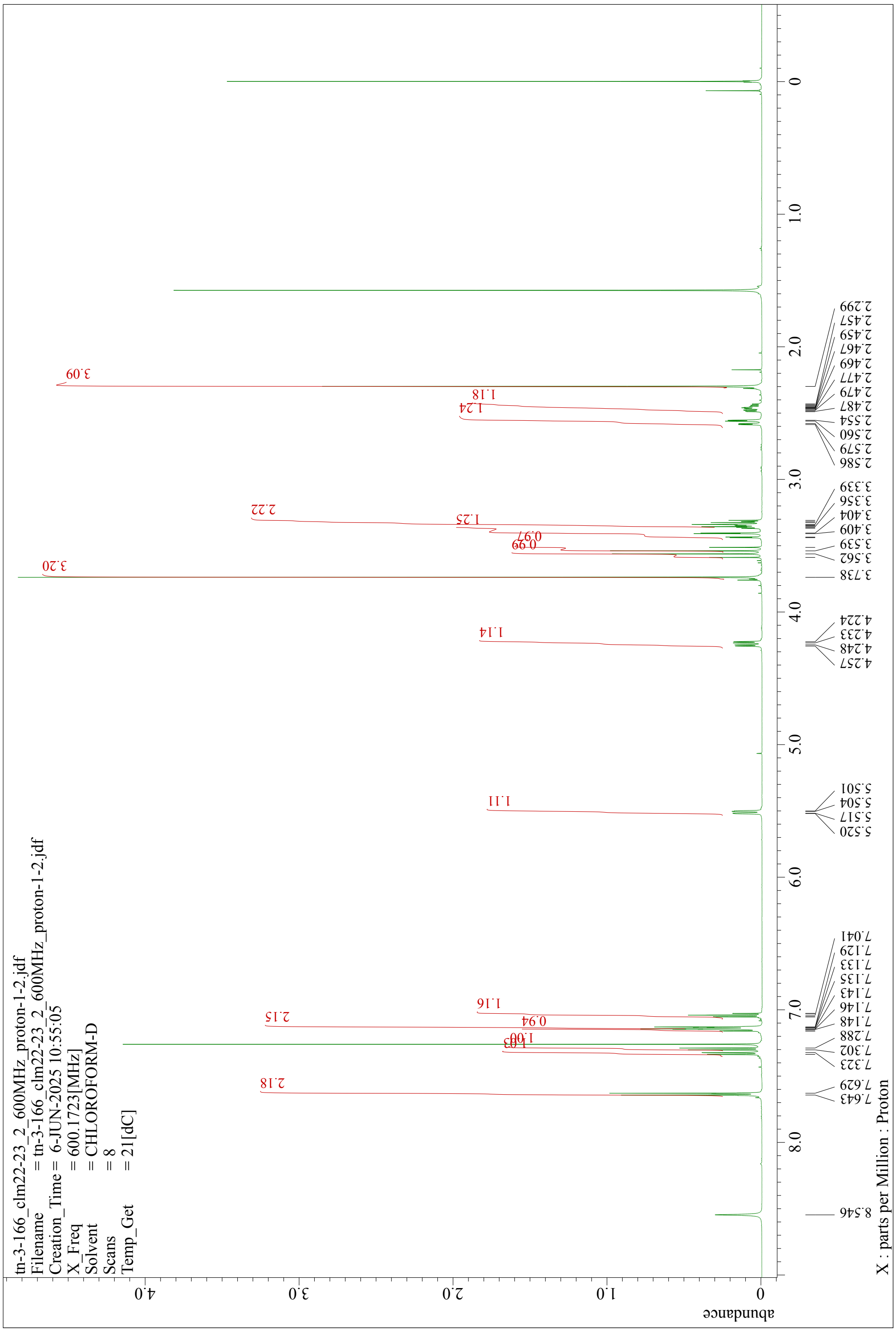

$^{13}C\{^1H\}$ NMR ($CDCl_3$, 150 MHz)

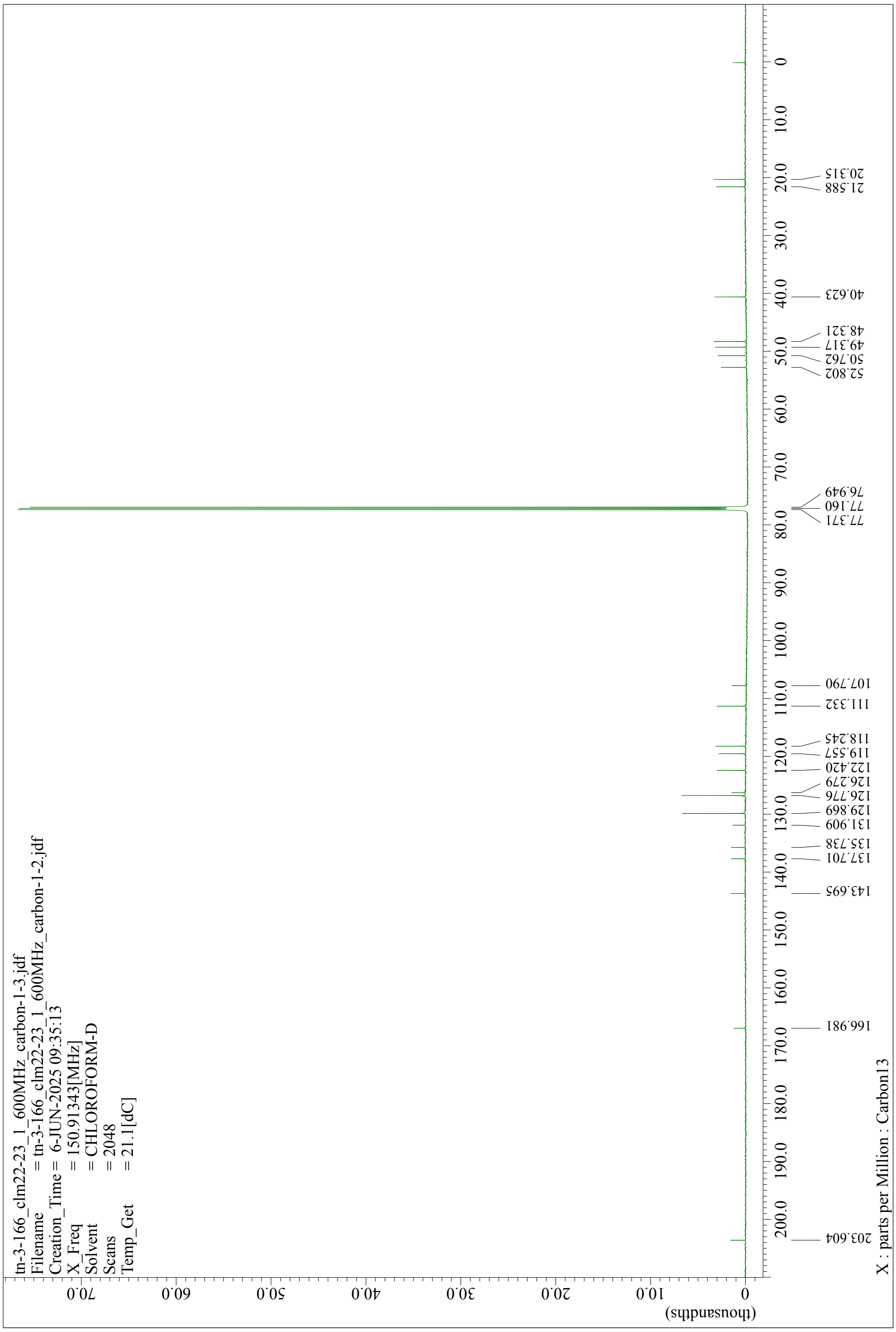

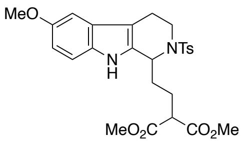

3g

^{1}H NMR ($CDCl_3$, 600 MHz)

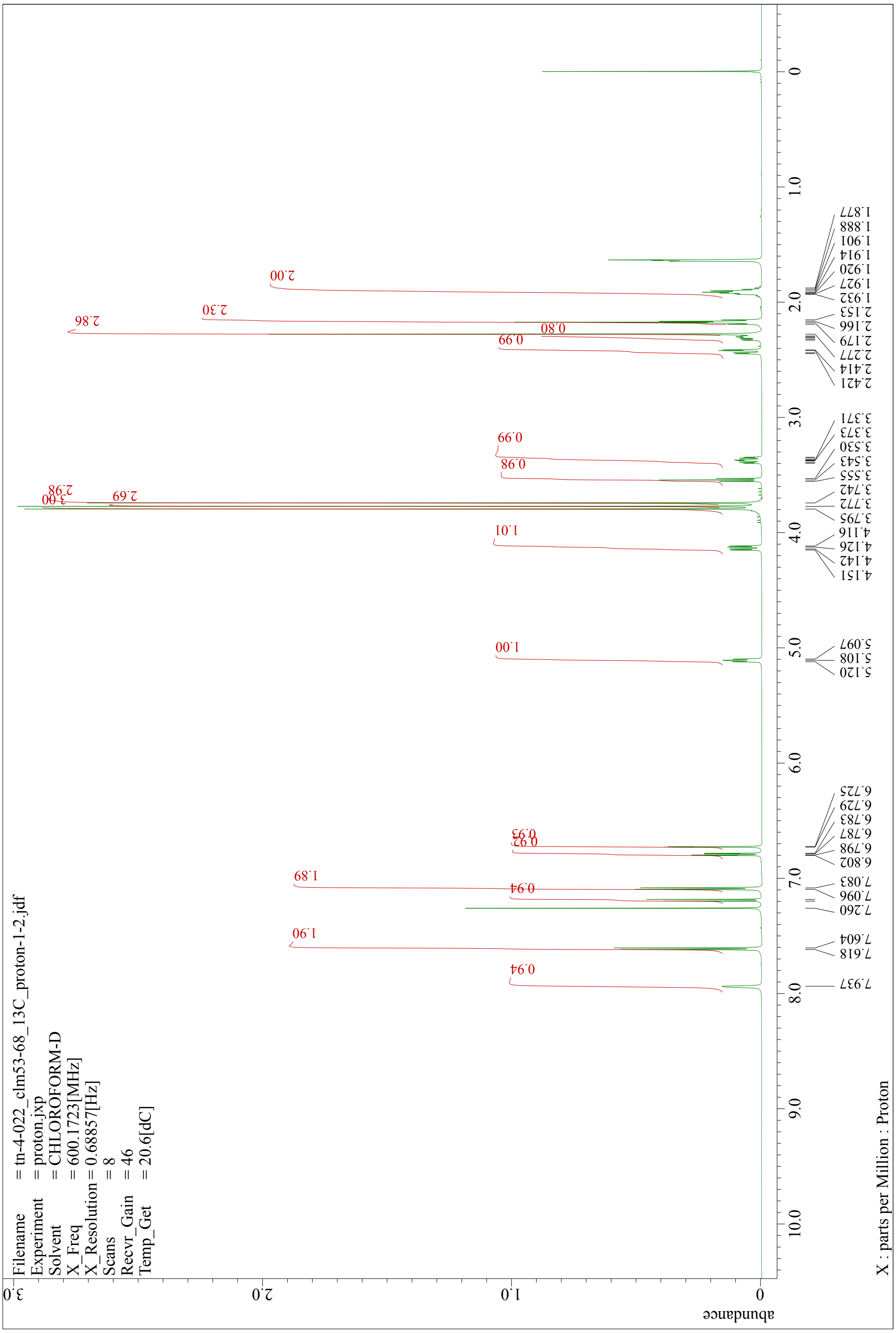

$^{13}C\{^{1}H\}$ NMR ($CDCl_3$, 150 MHz)

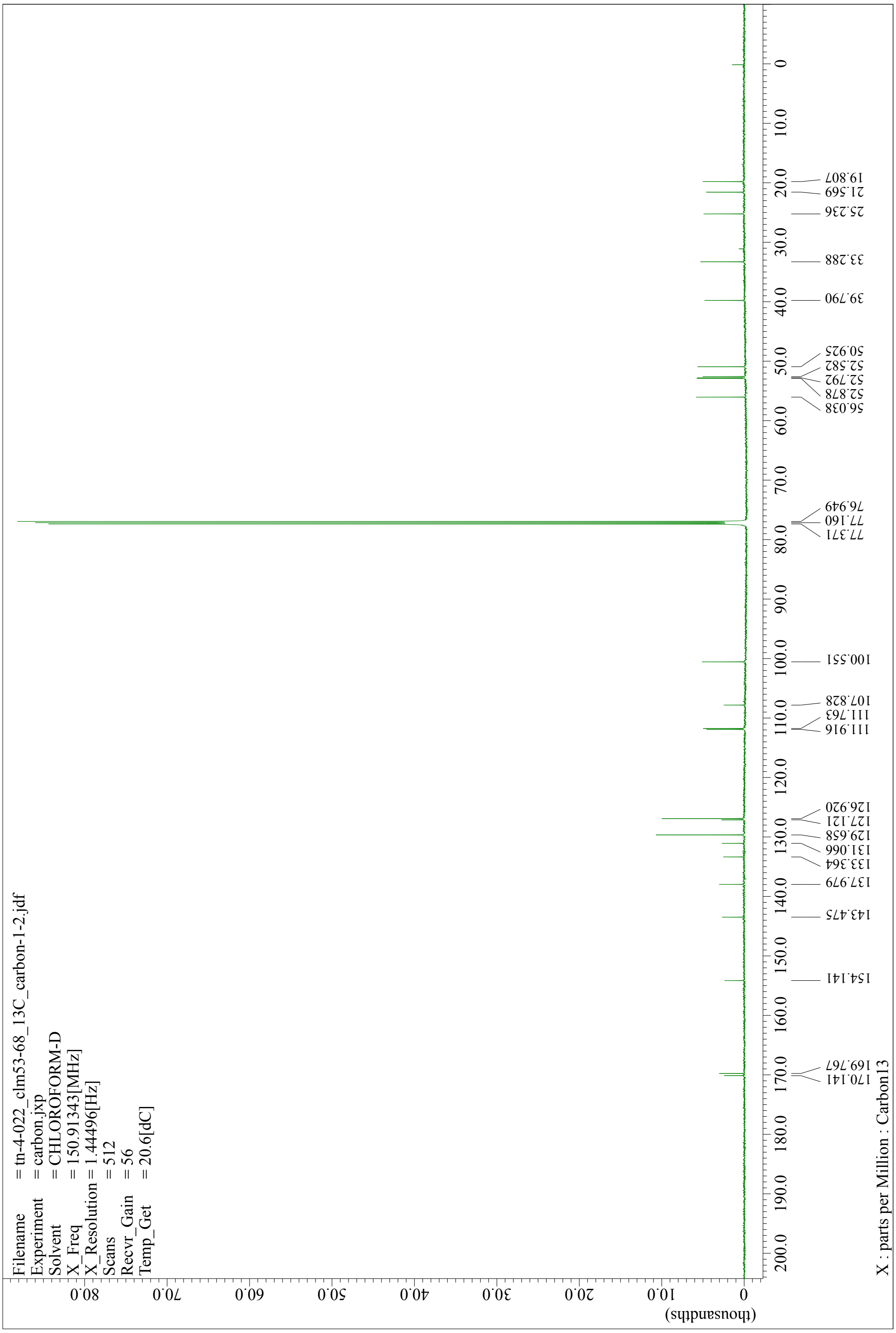

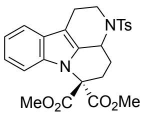

4a

^{1}H NMR ($CDCl_3$, 600 MHz)

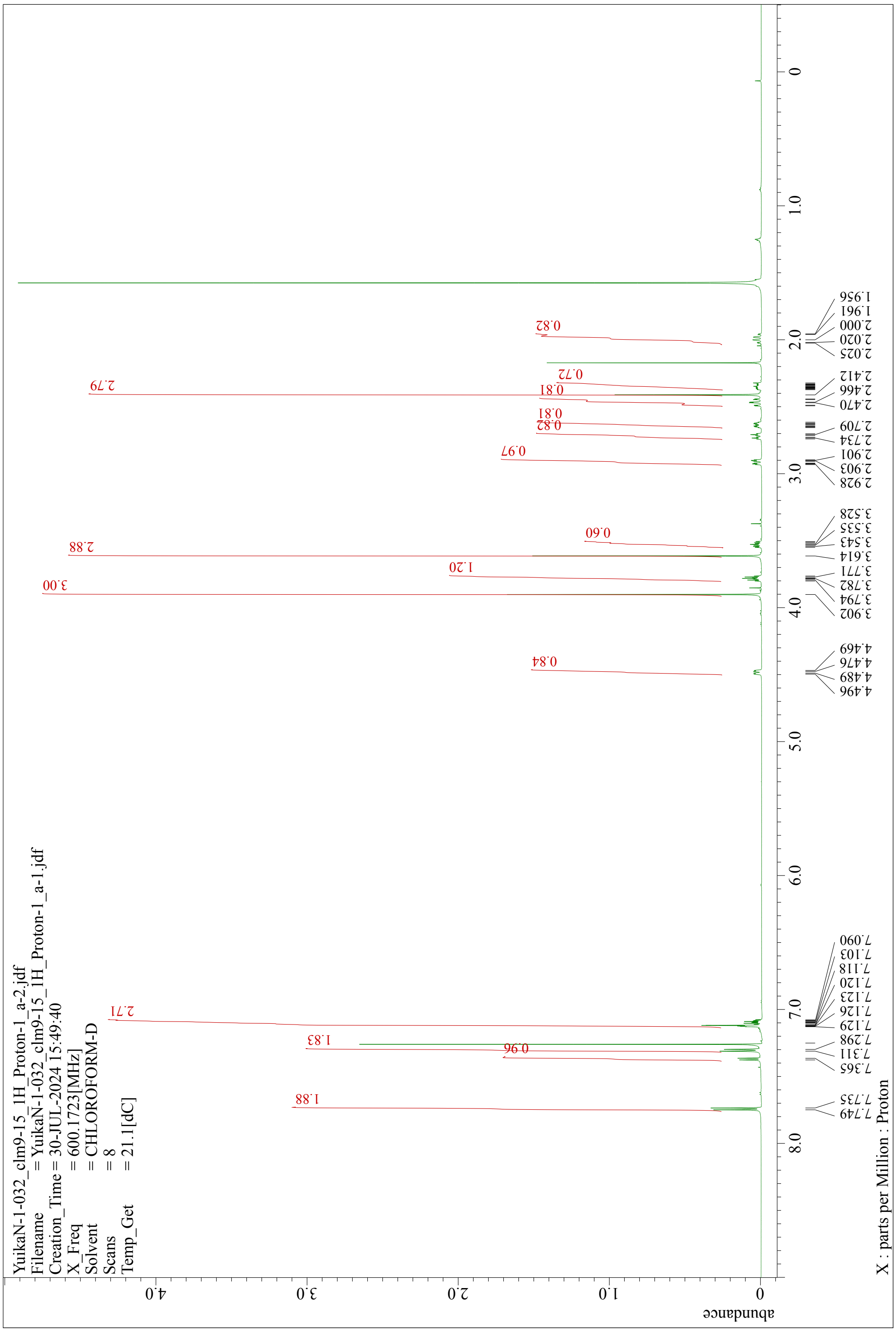

$^{13}C\{^{1}H\}$ NMR ($CDCl_3$, 150 MHz)

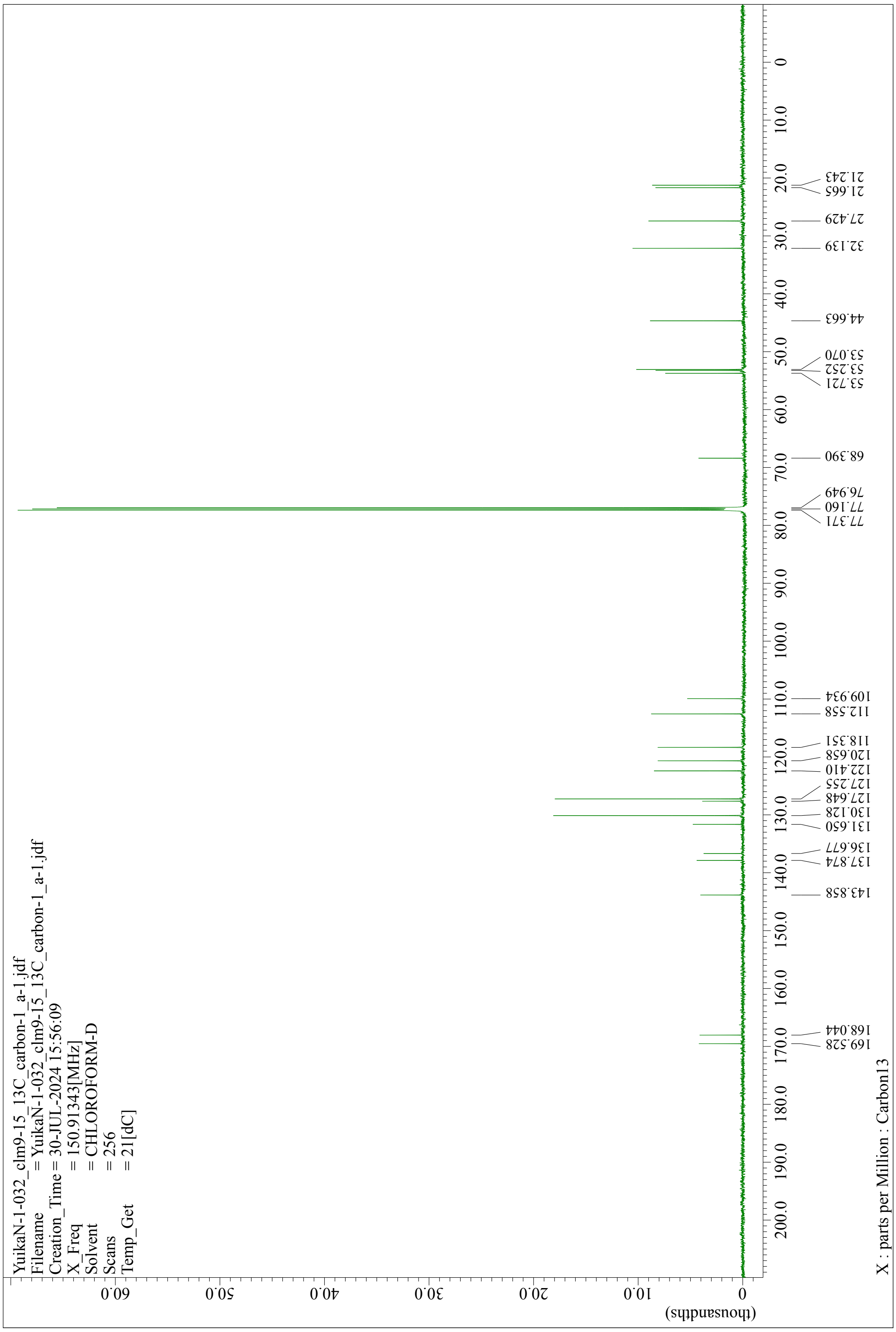

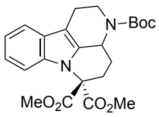

4b

^{1}H NMR ($CDCl_3$, 600 MHz)

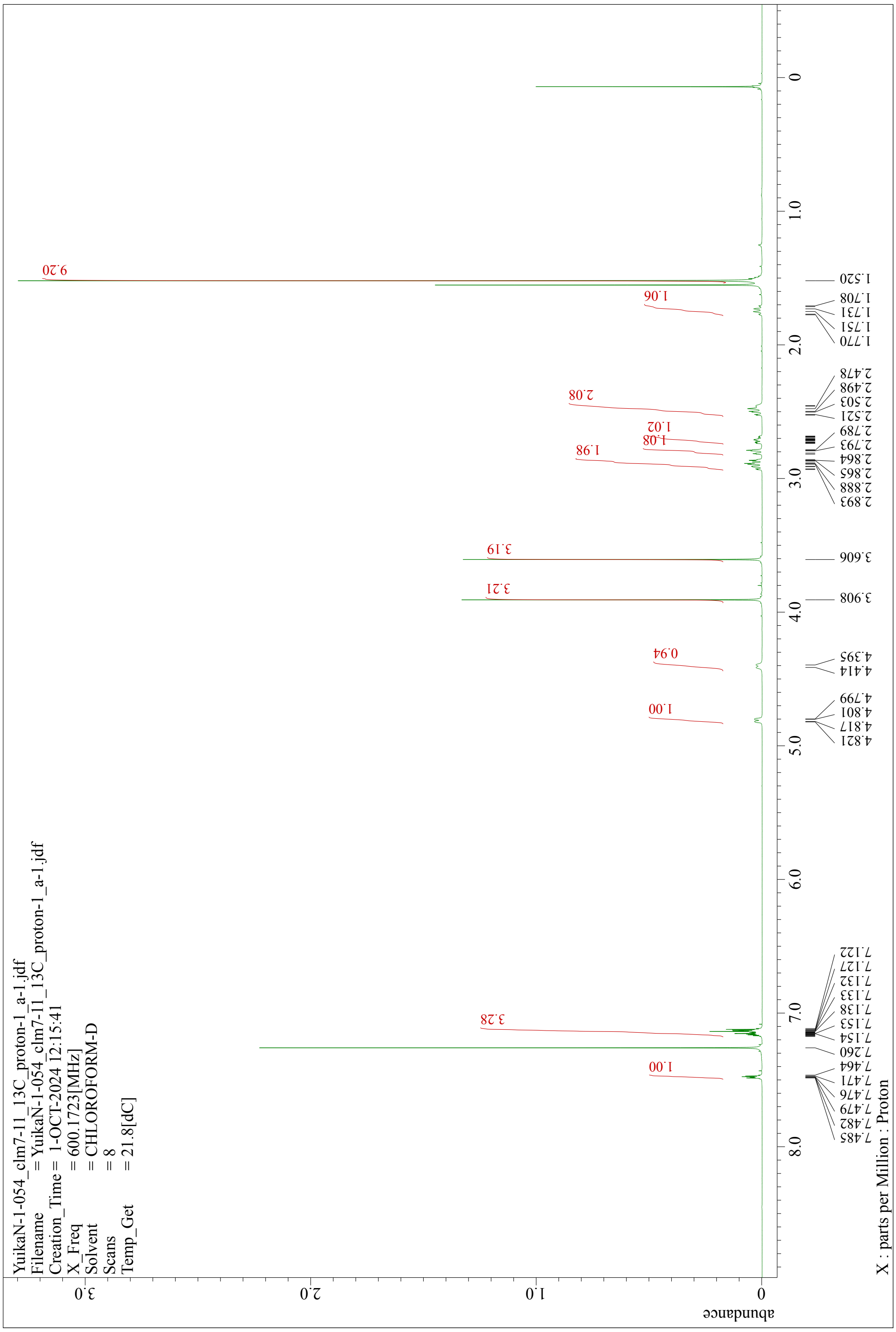

$^{13}C\{^1H\}$ NMR ($CDCl_3$, 150 MHz)

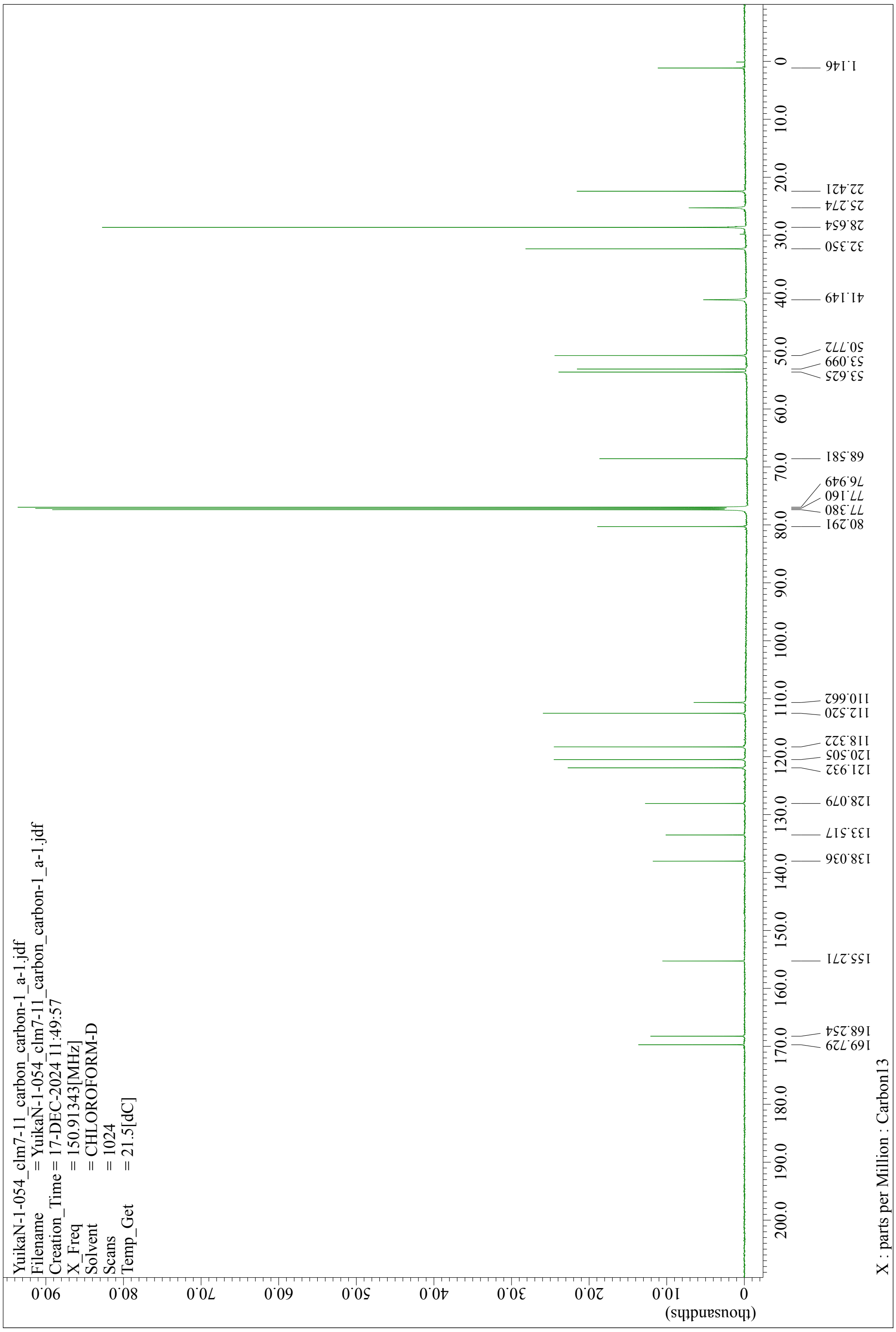

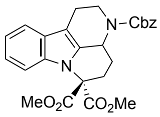

4c

^{1}H NMR ($CDCl_3$, 600 MHz)

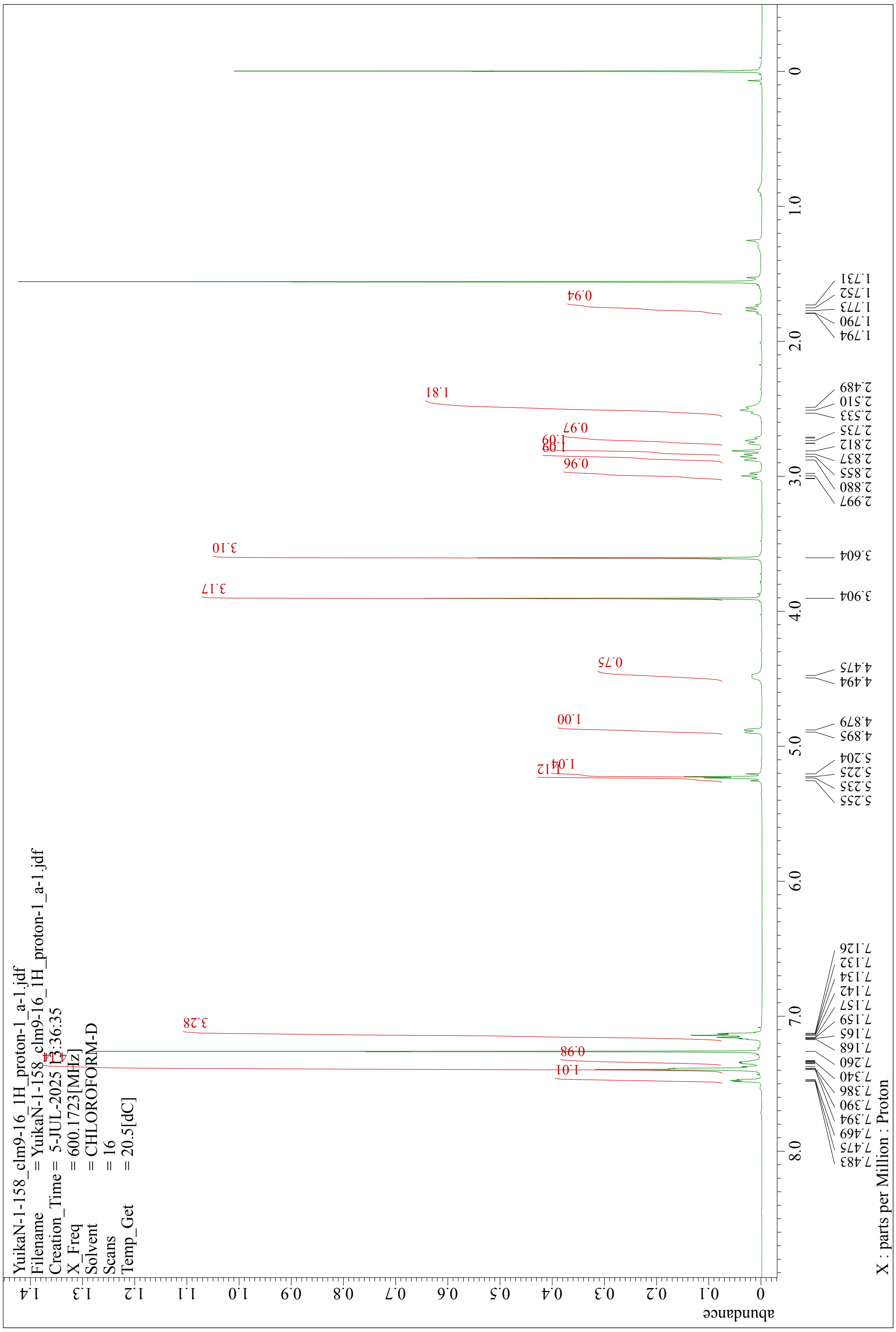

$^{13}C\{^1H\}$ NMR ($CDCl_3$, 150 MHz)

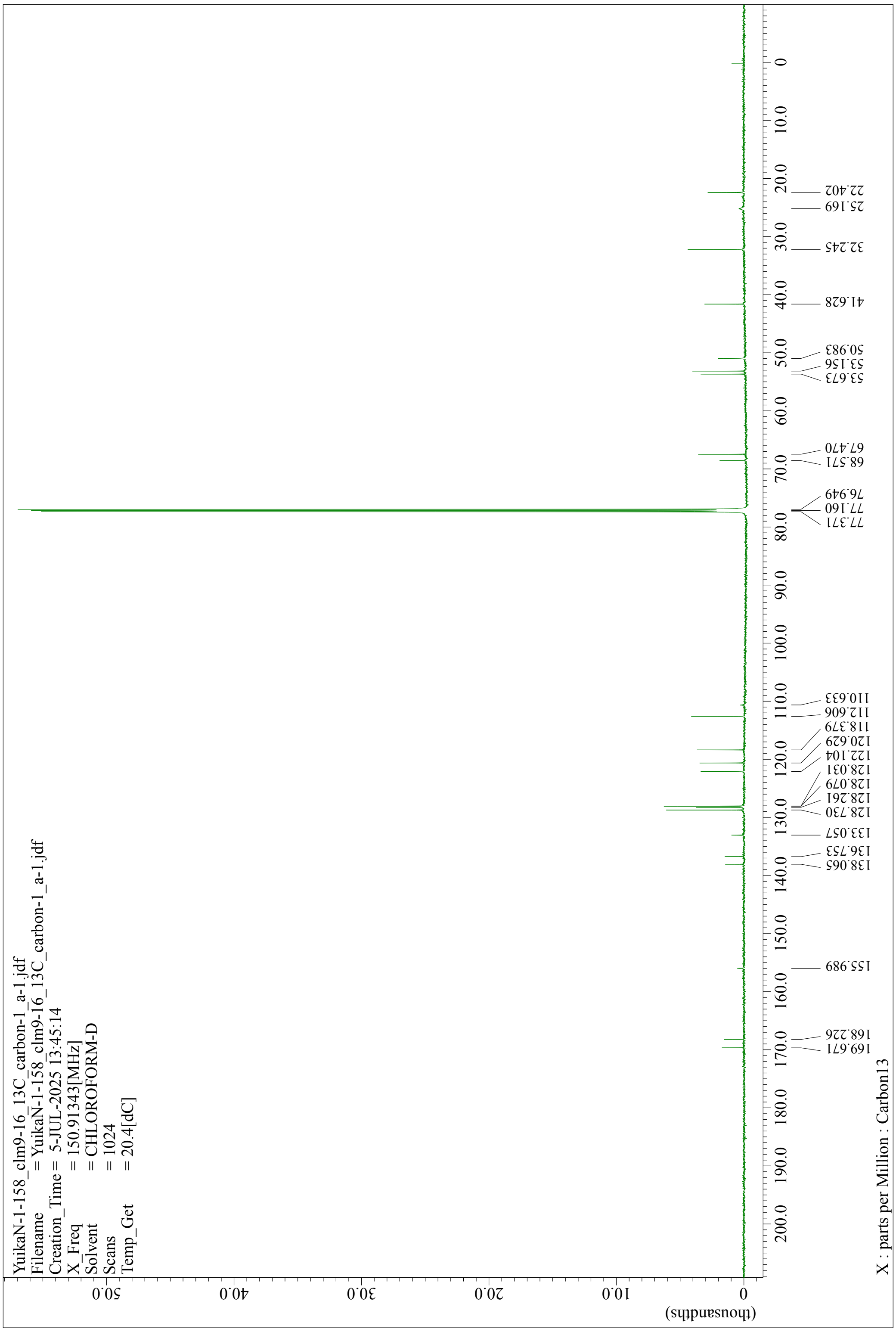

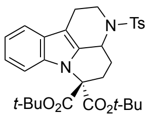

4e

^{1}H NMR ($CDCl_3$, 600 MHz)

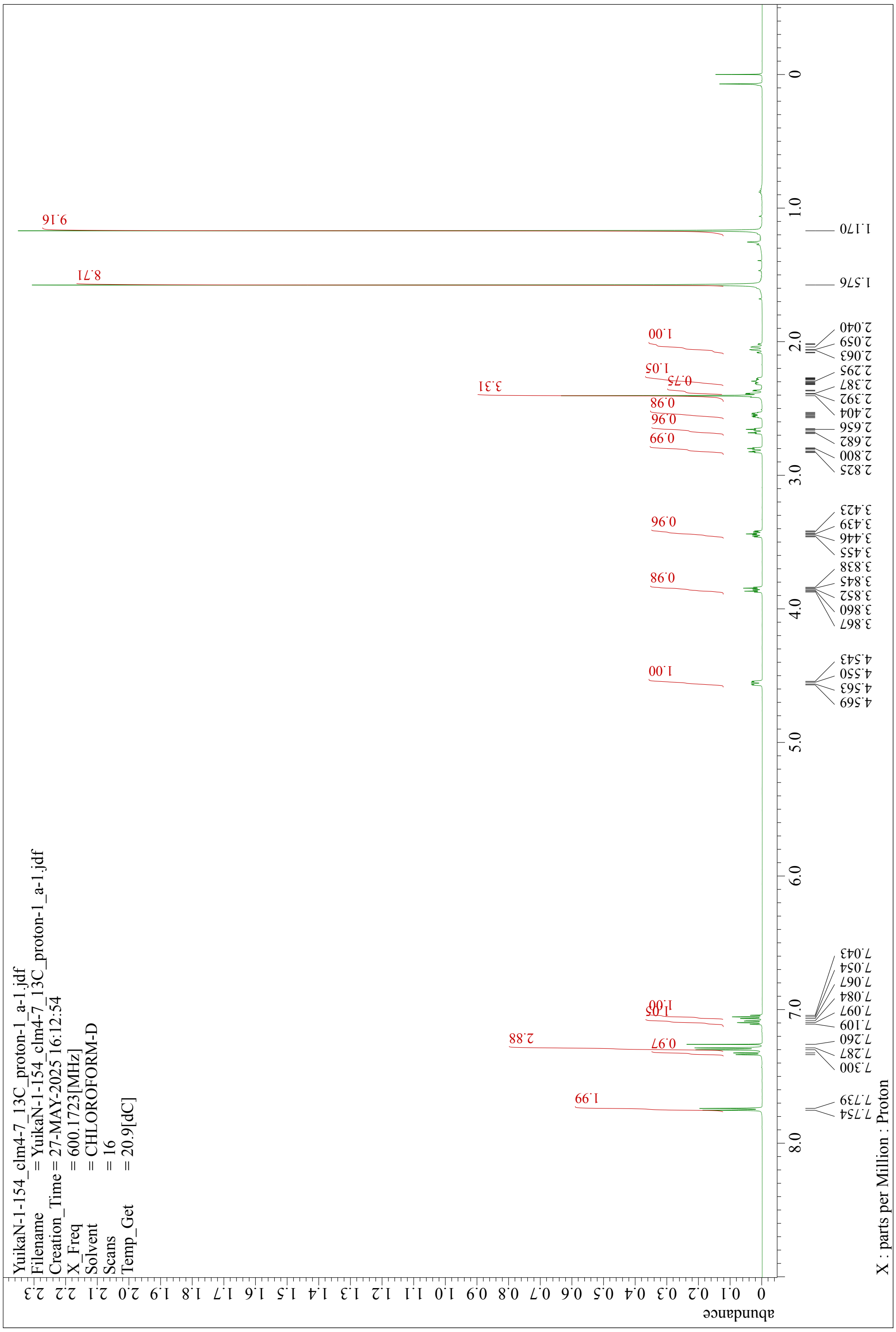

$^{13}C\{^{1}H\}$ NMR ($CDCl_3$, 150 MHz)

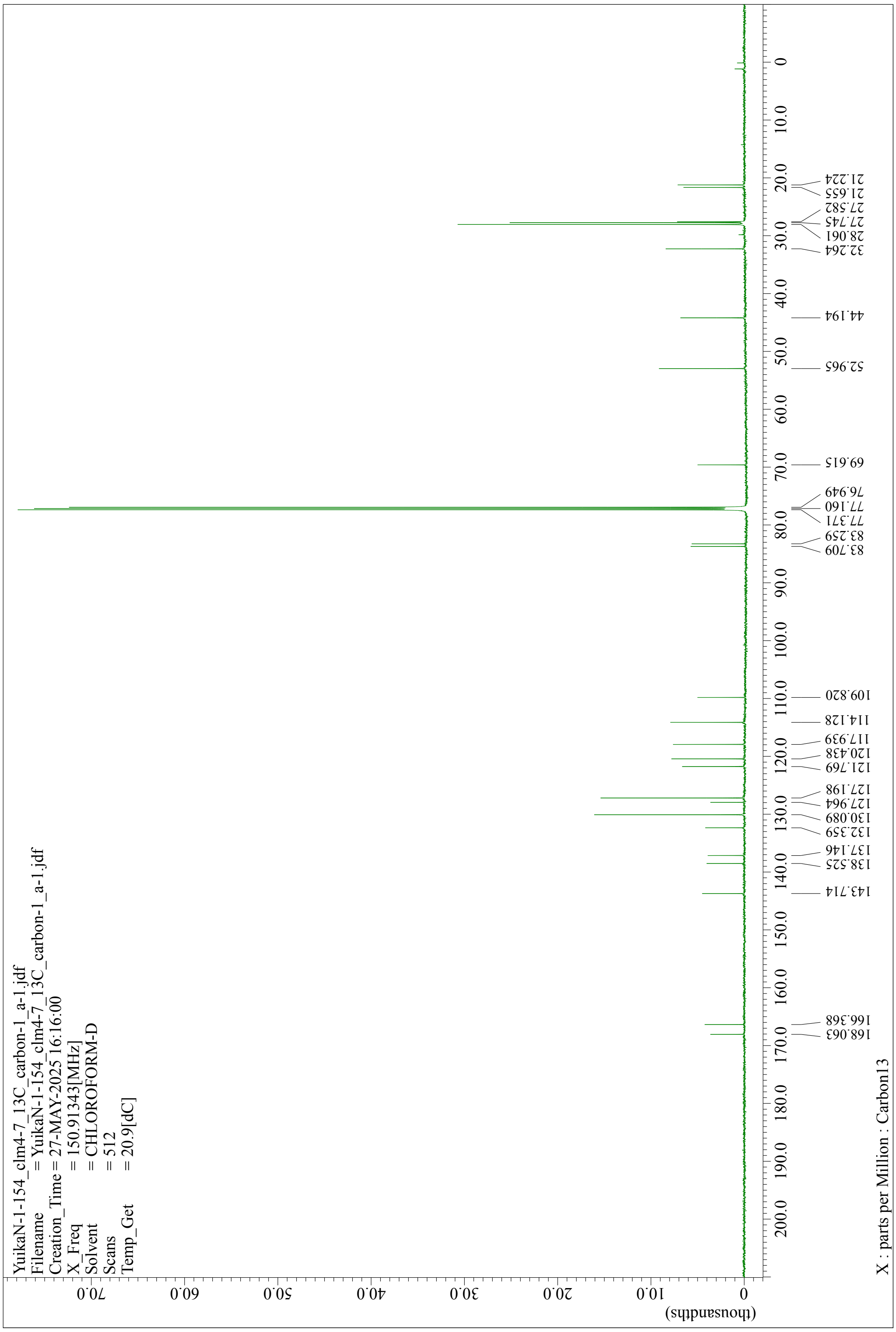

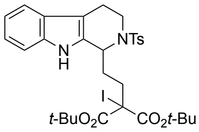

9e

^{1}H NMR ($CDCl_3$, 600 MHz)

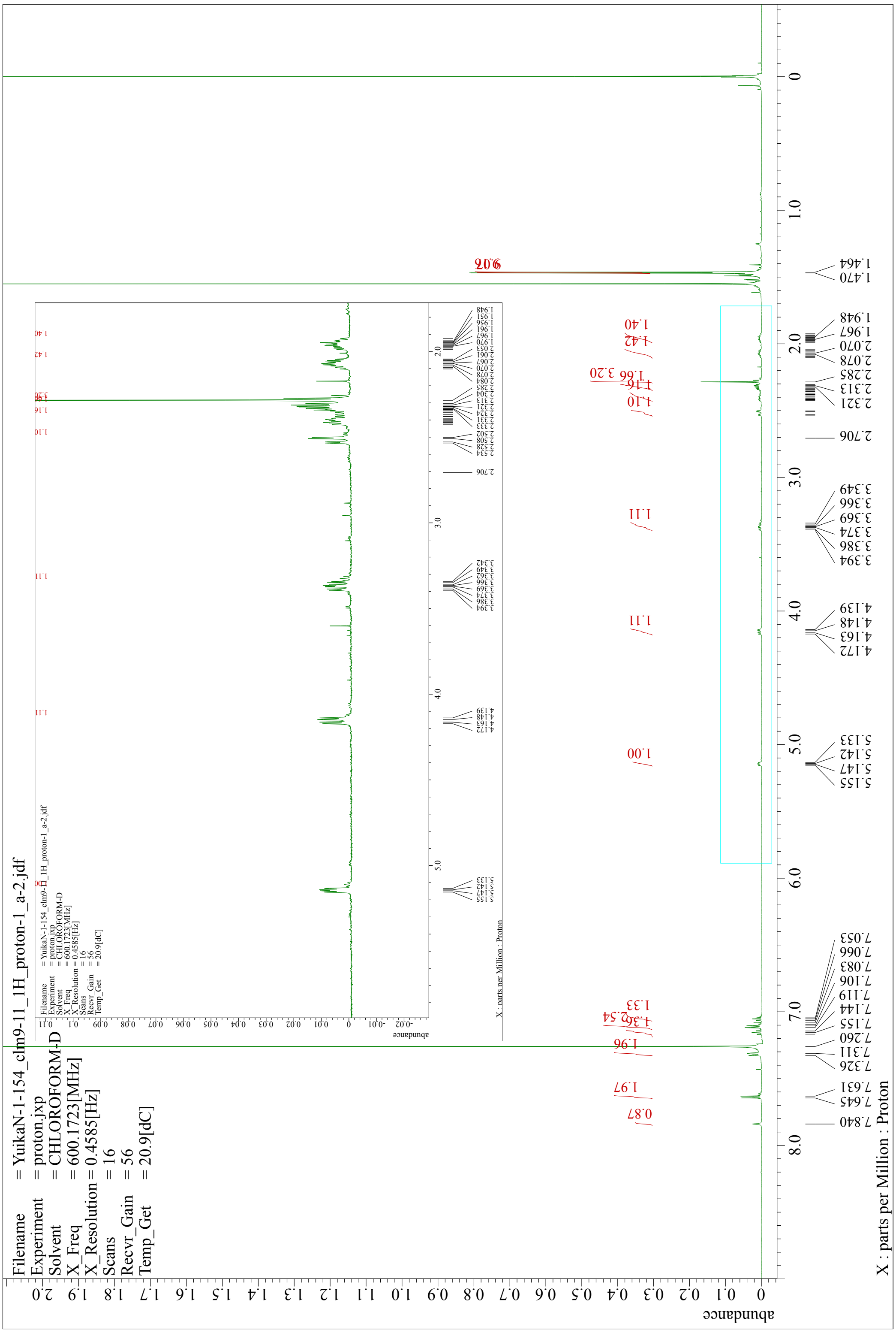

$^{13}C\{^{1}H\}$ NMR ($CDCl_3$, 150 MHz)

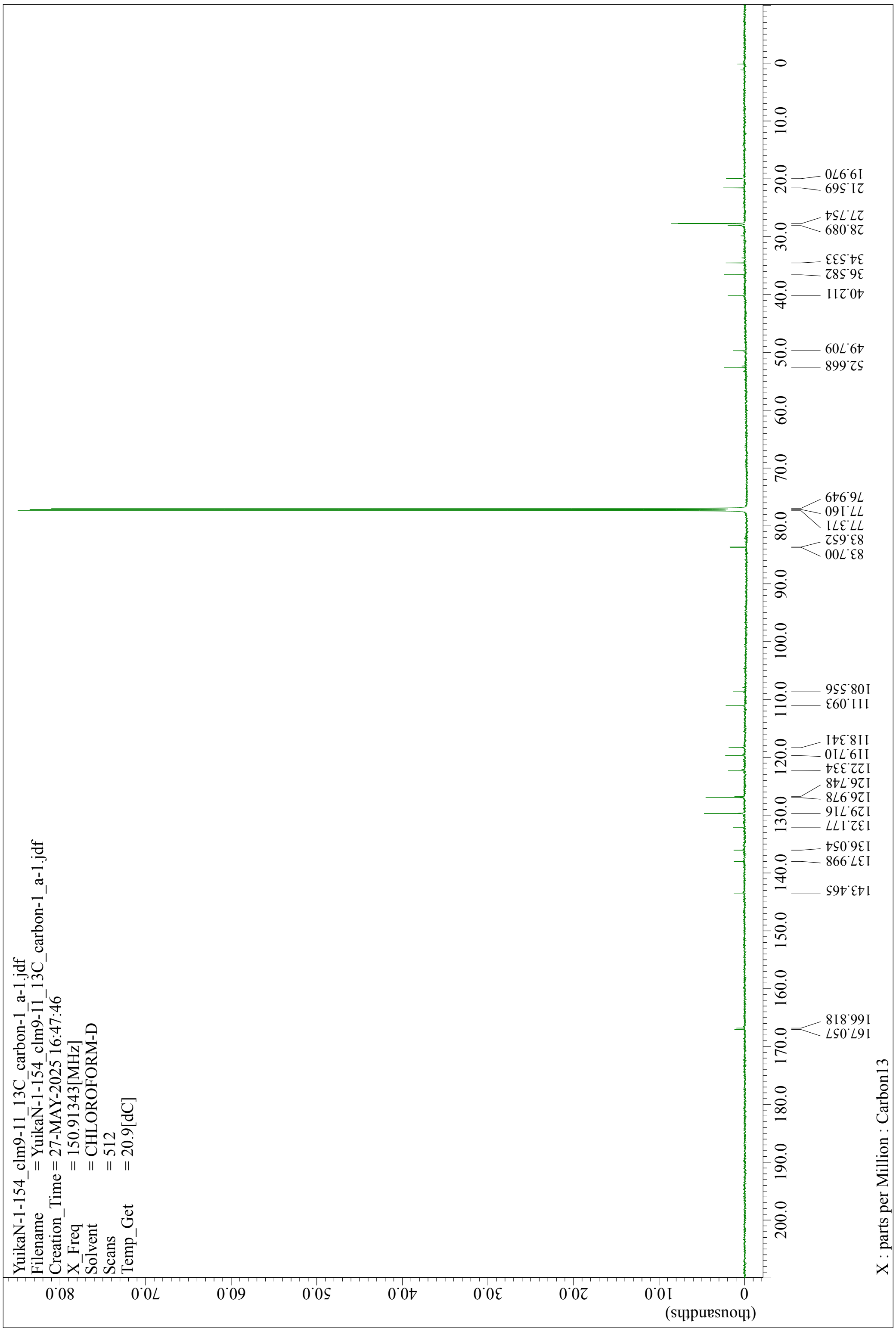

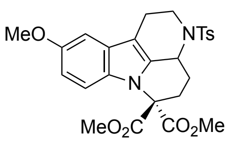

4g

^{1}H NMR ($CDCl_3$, 600 MHz)

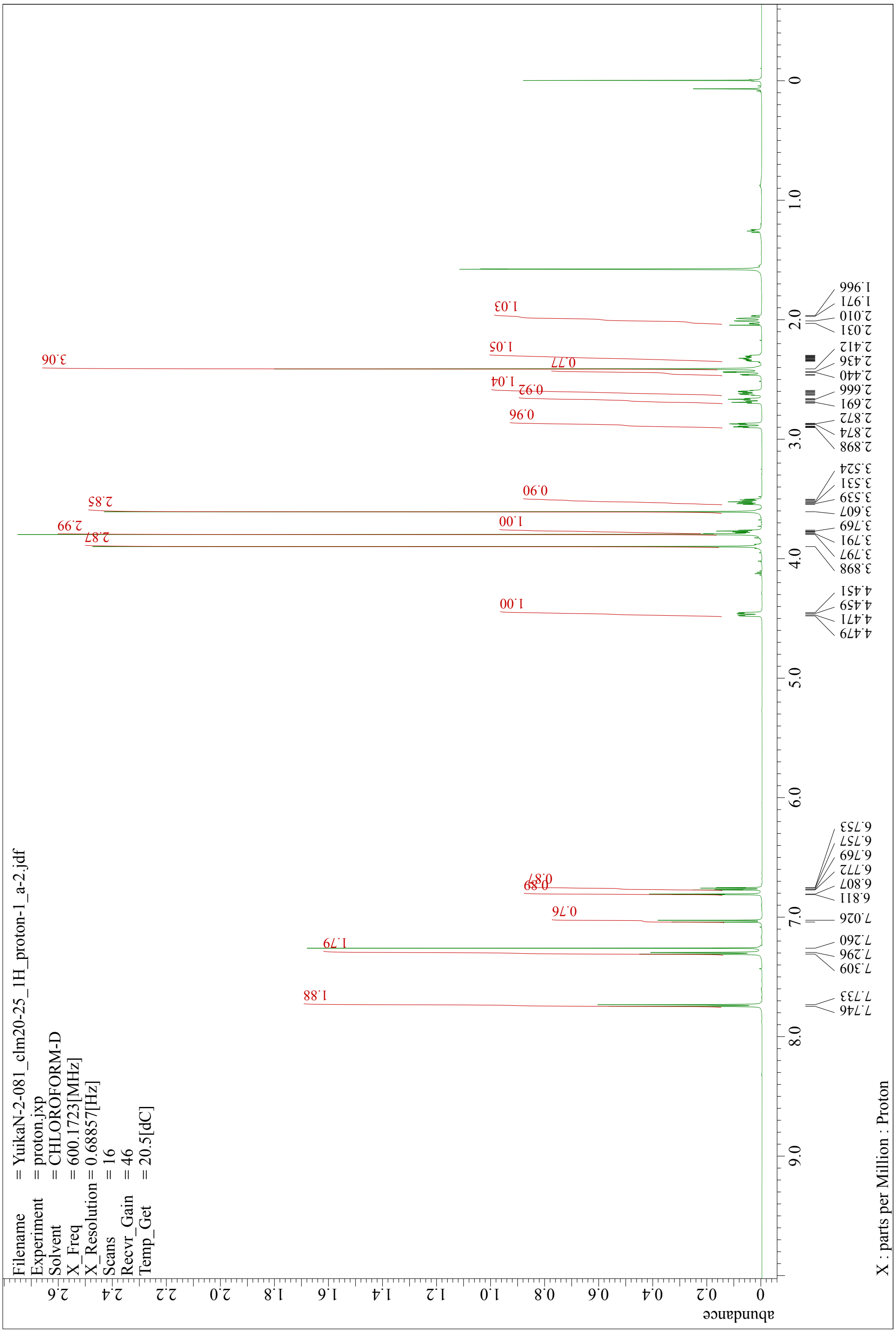

$^{13}C\{^{1}H\}$ NMR ($CDCl_3$, 150 MHz)

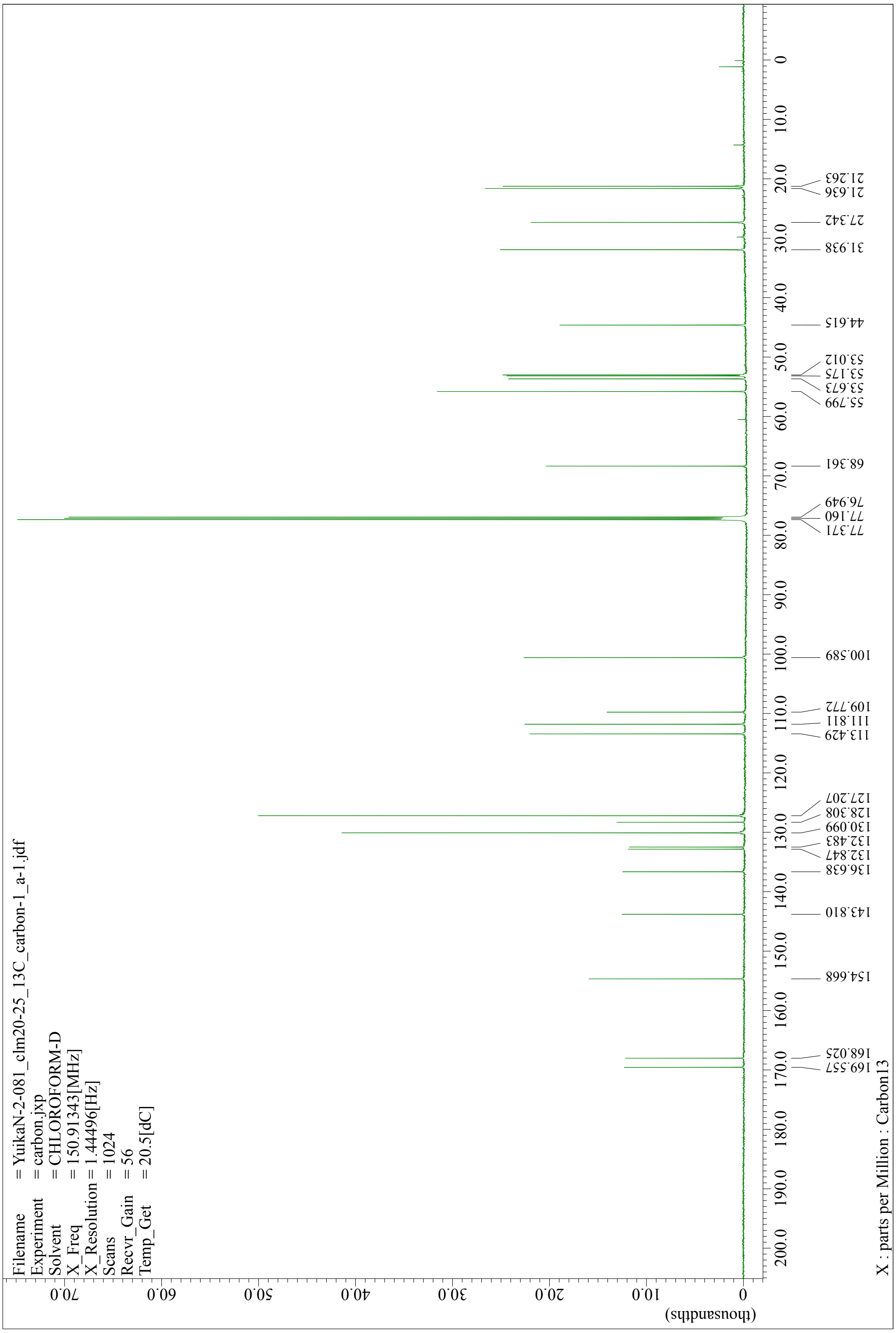

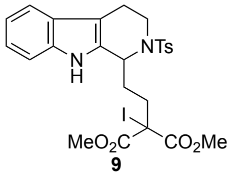

9

^{1}H NMR ($CDCl_3$, 600 MHz)

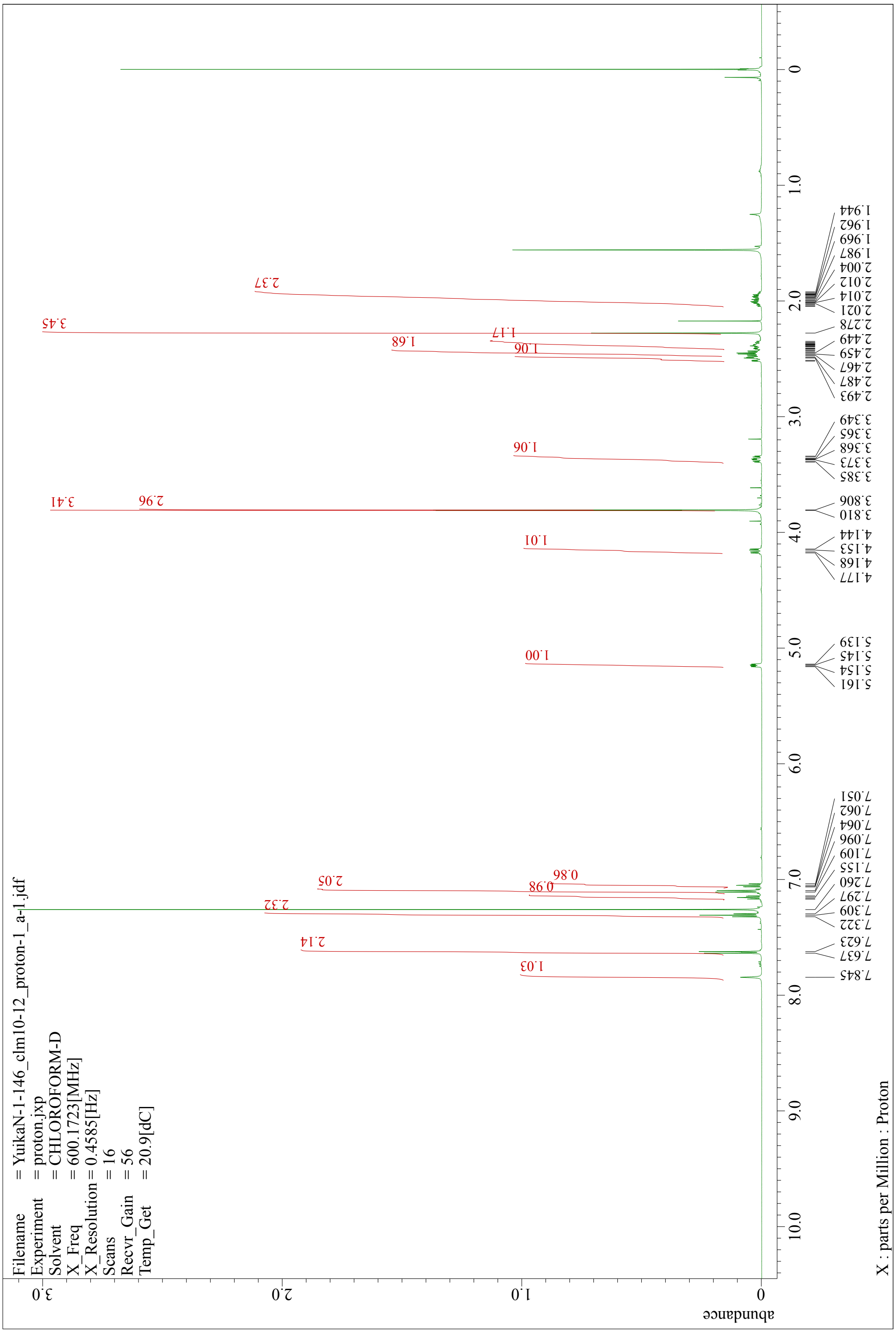

$^{13}C\{^{1}H\}$ NMR ($CDCl_3$, 150 MHz)

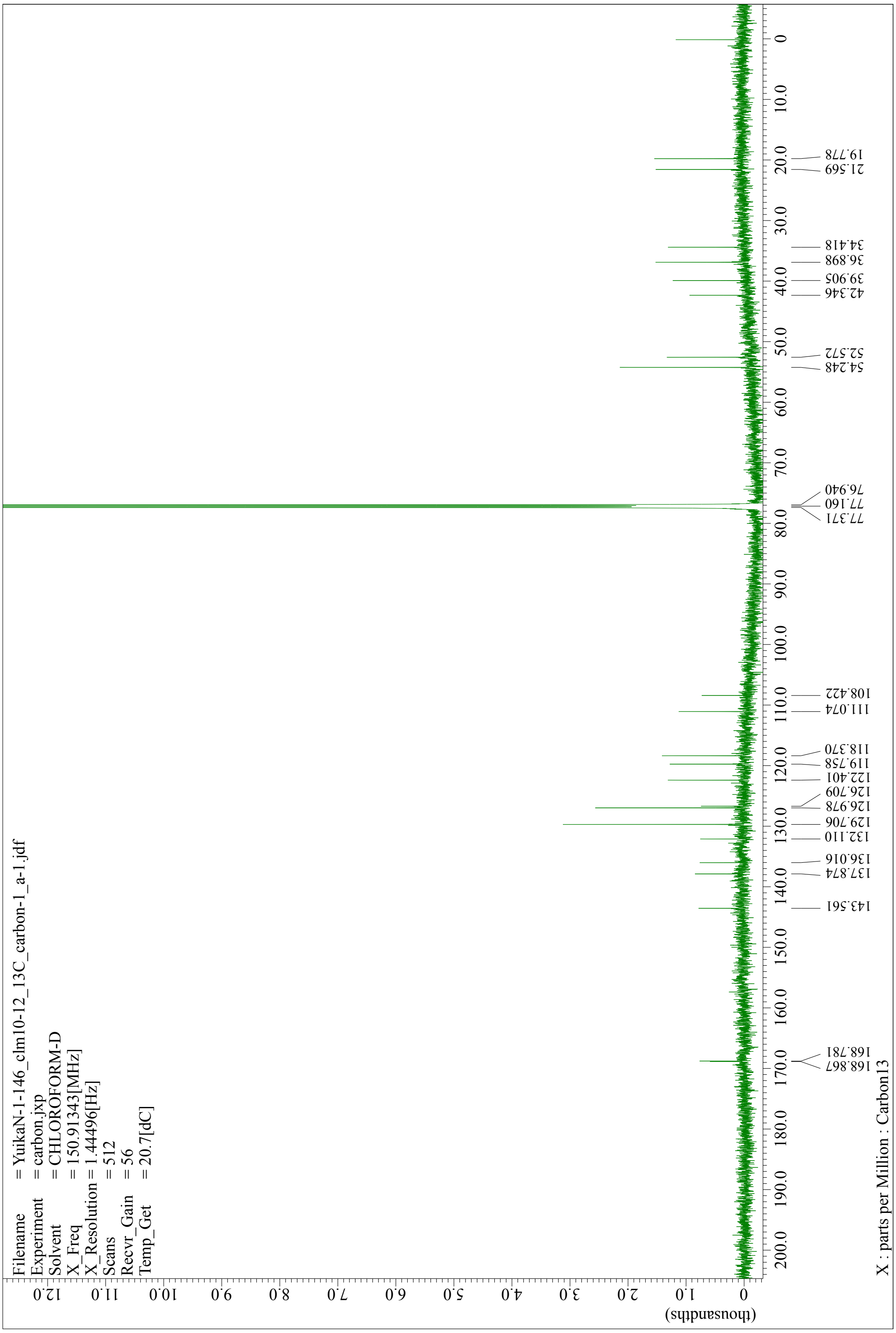

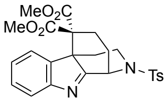

8a

^{1}H NMR ($CDCl_3$, 600 MHz)

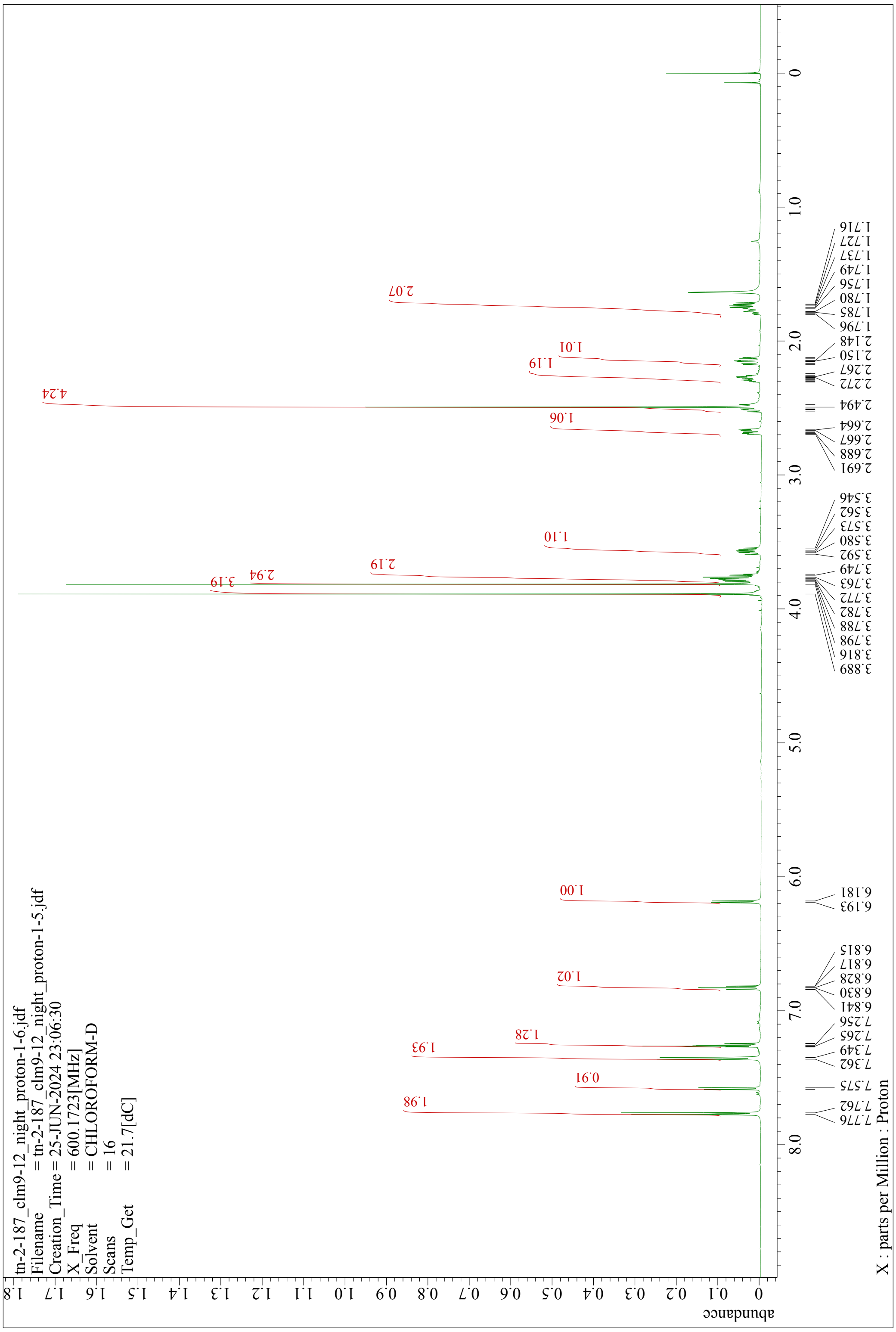

$^{13}C\{^{1}H\}$ NMR ($CDCl_3$, 150 MHz)

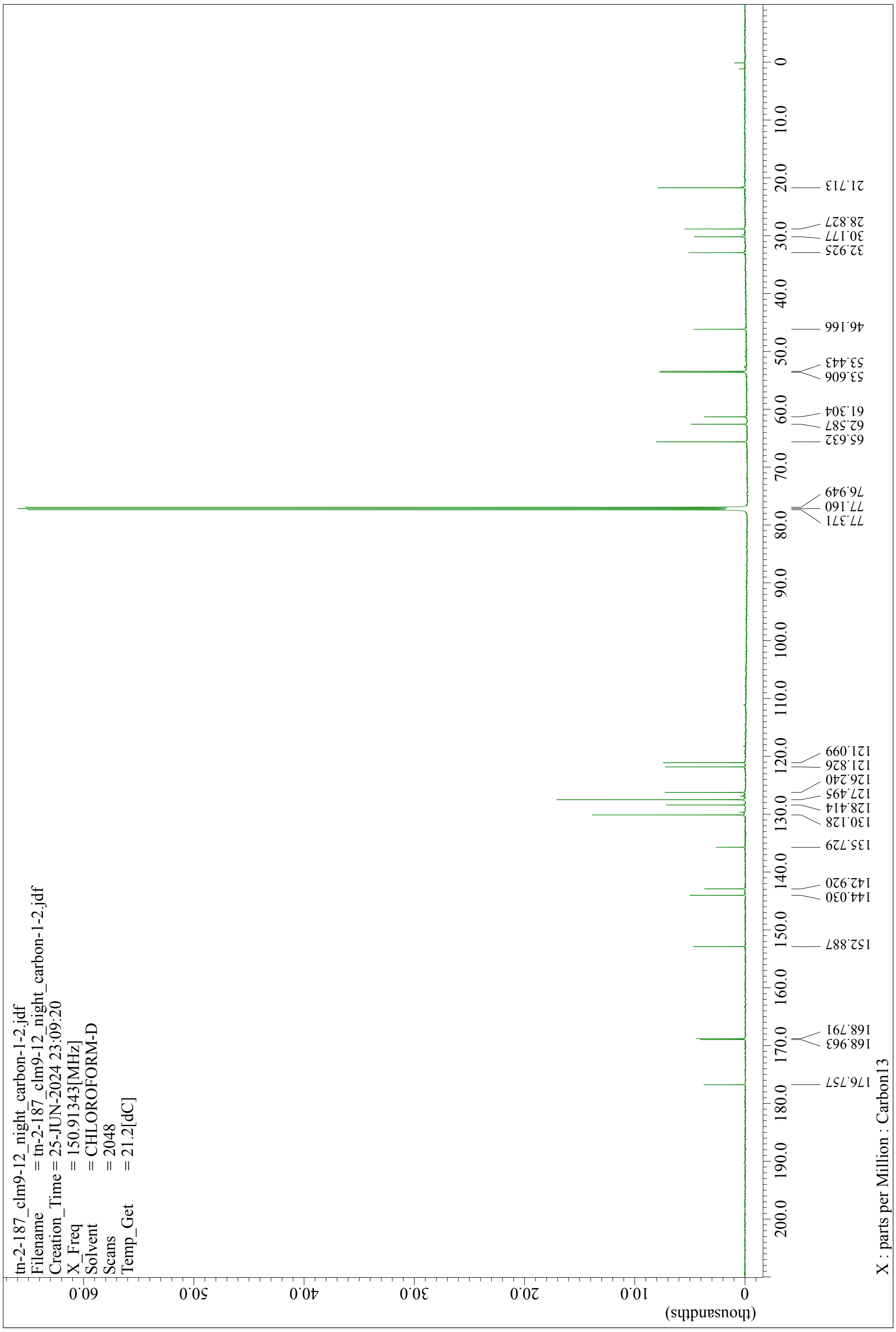

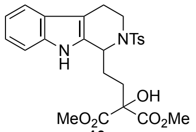

10

^{1}H NMR ($CDCl_3$, 600 MHz)

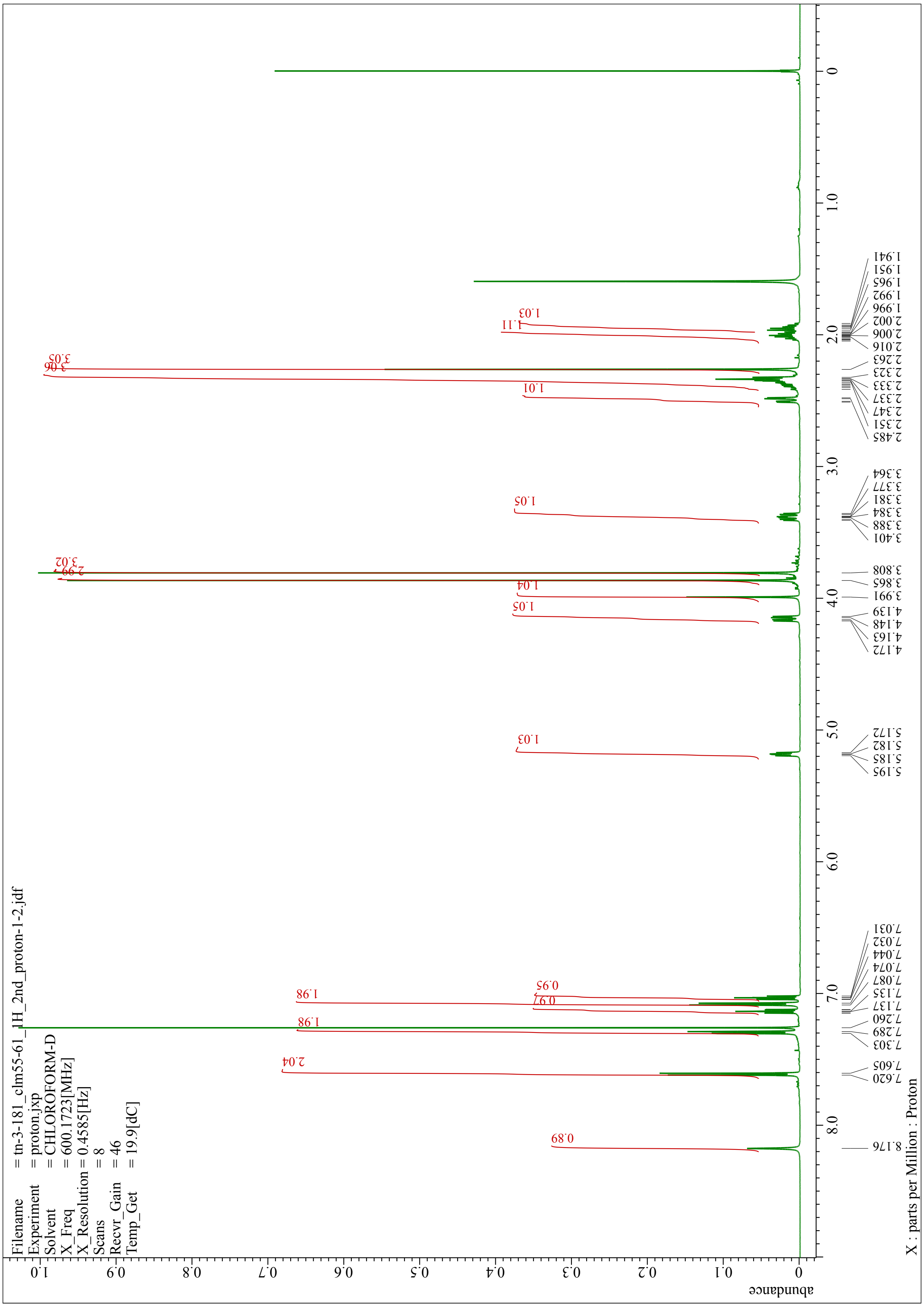

^{13}C NMR ($CDCl_3$, 150 MHz)

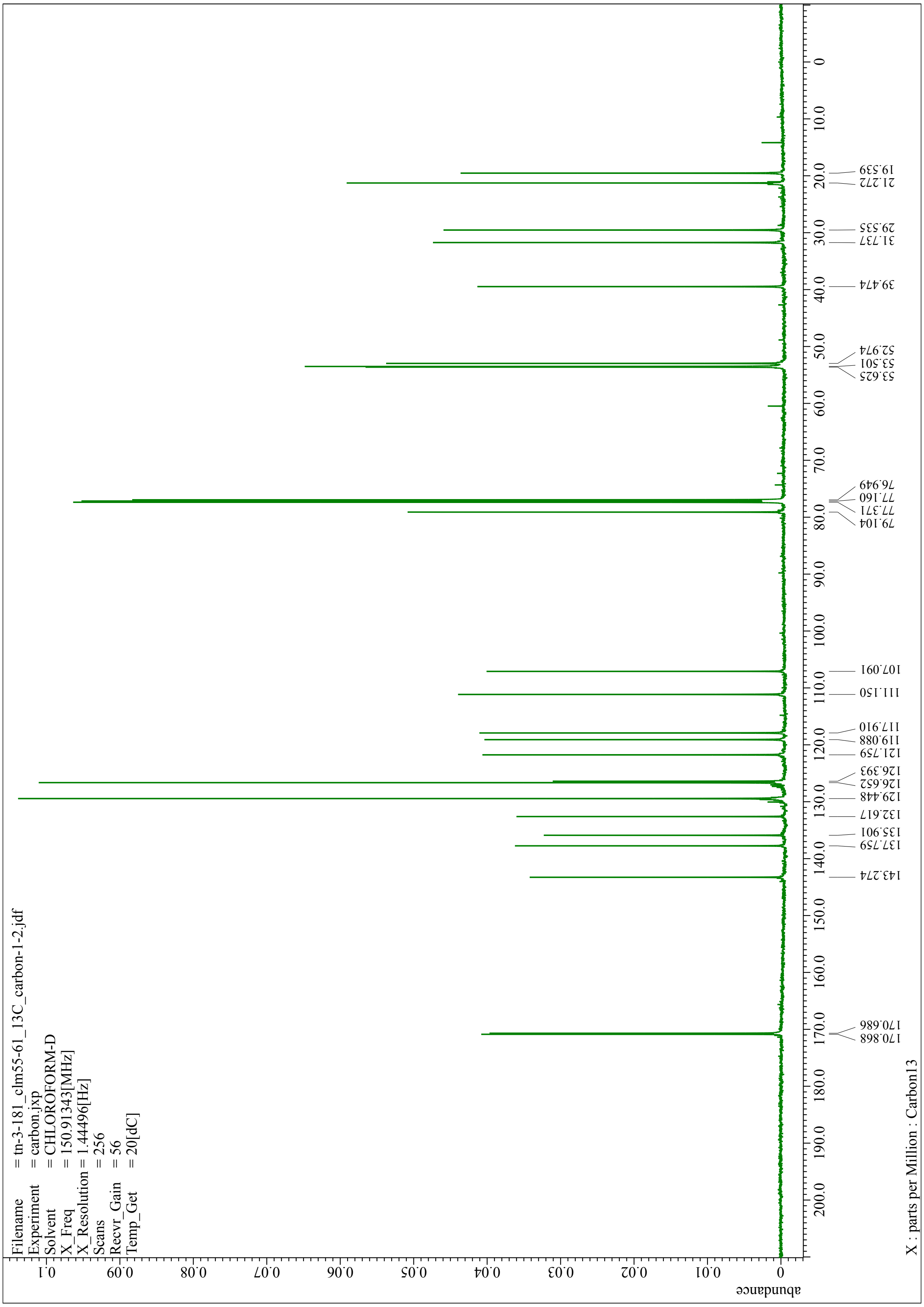

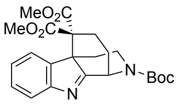

8b

^{1}H NMR (CDCl$_3$, 600 MHz)

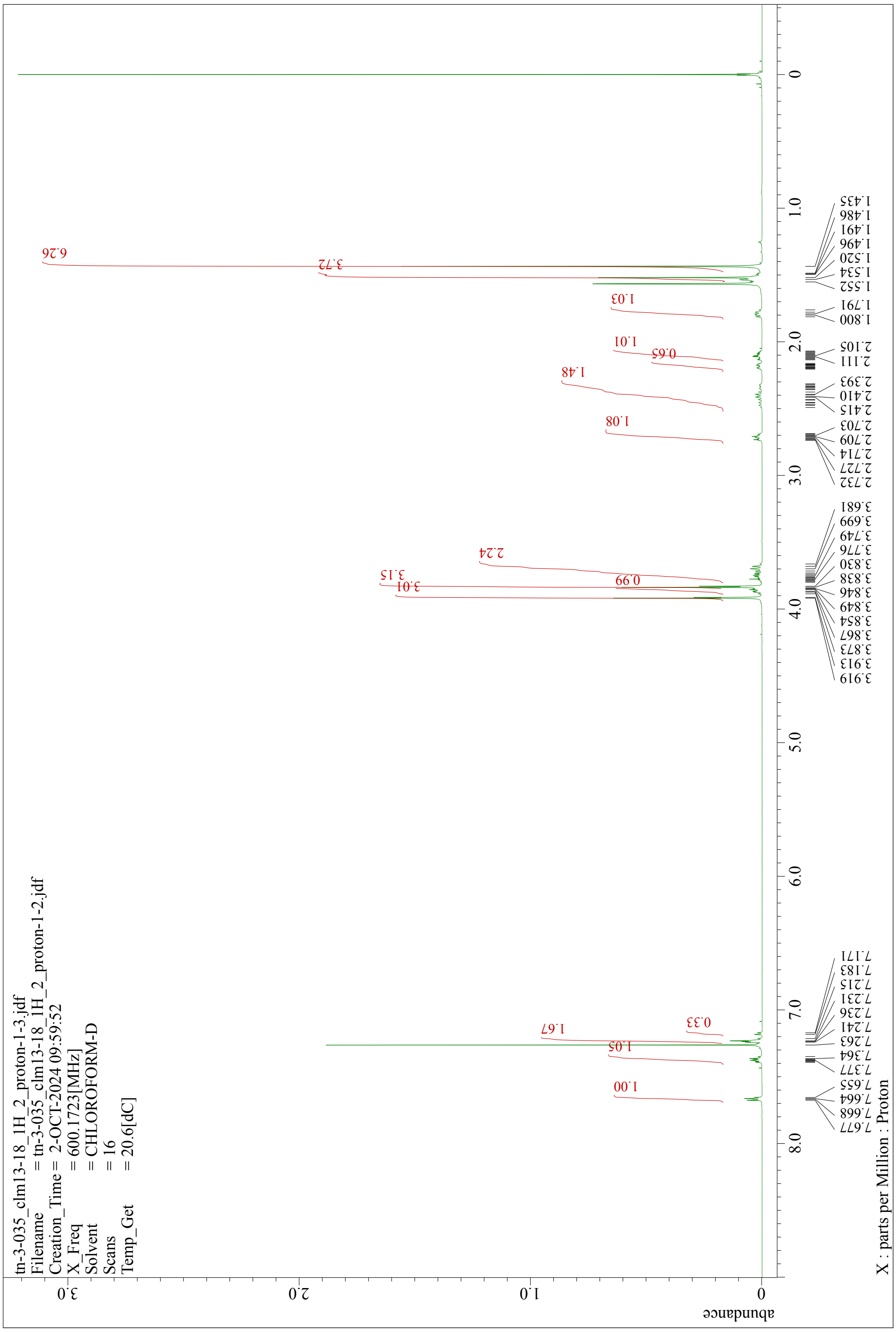

$^{13}C\{^{1}H\}$ NMR ($CDCl_3$, 150 MHz)

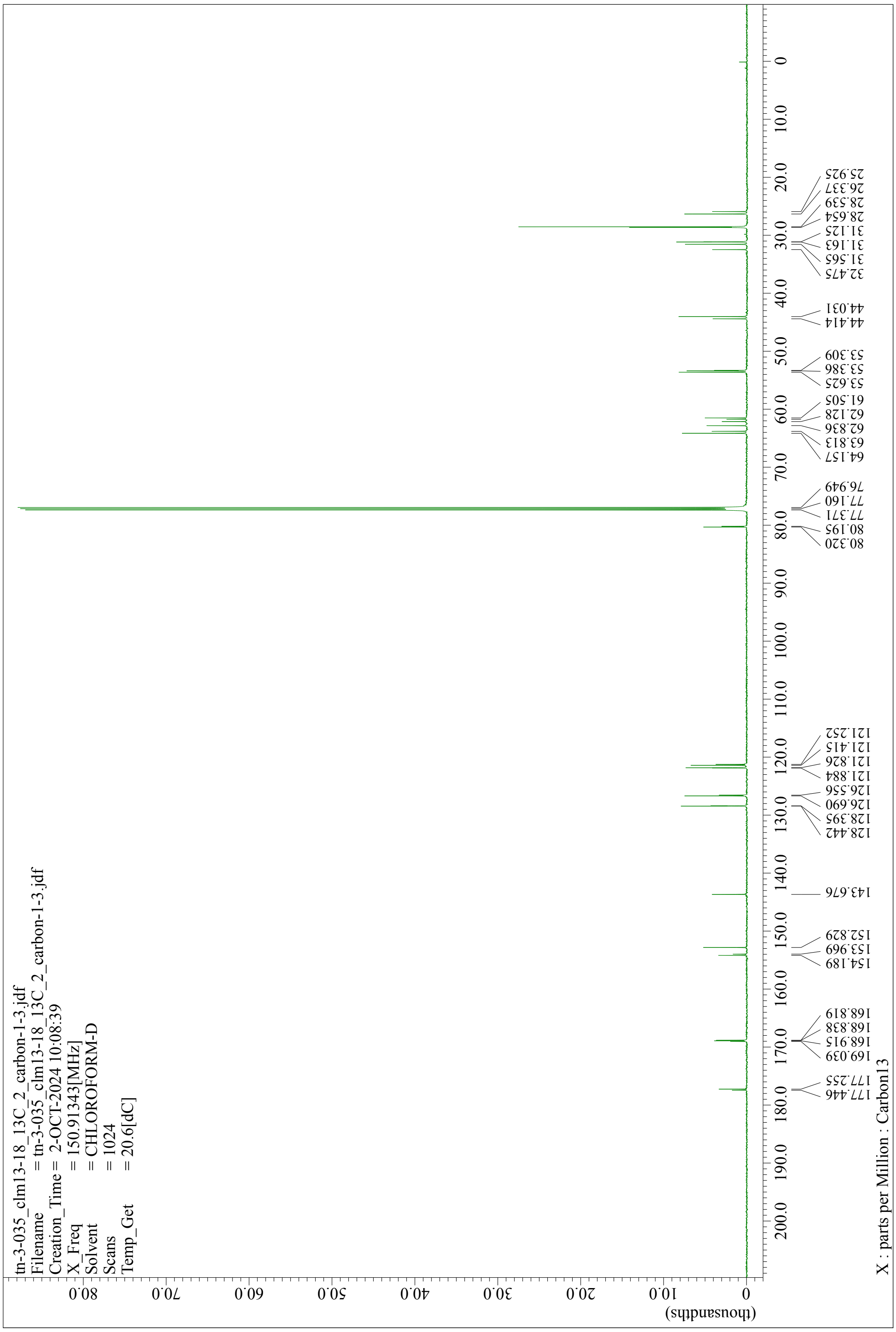

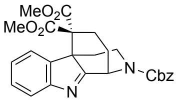

8c

^{1}H NMR ($CDCl_3$, 600 MHz)

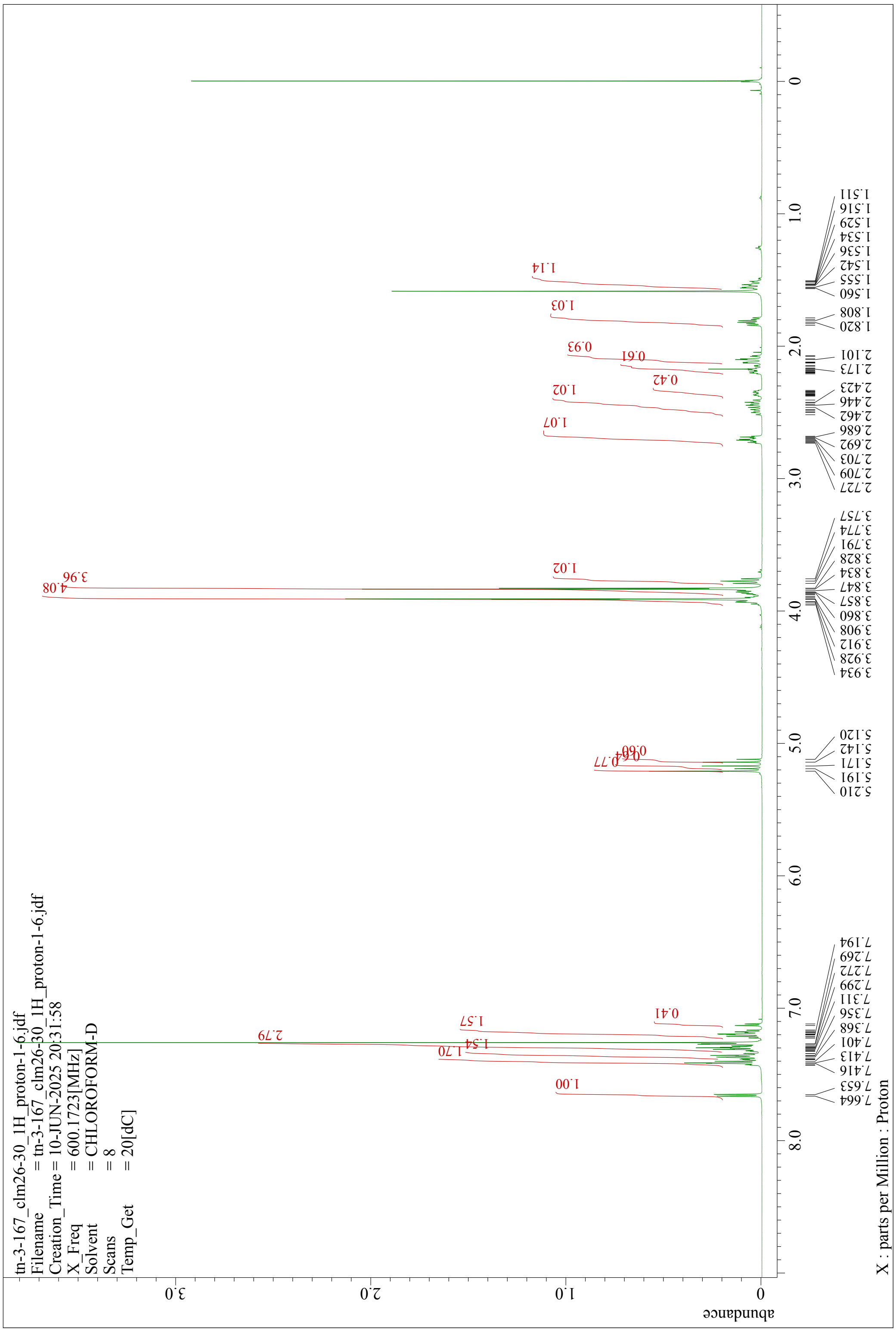

$^{13}C\{^{1}H\}$ NMR ($CDCl_3$, 150 MHz)

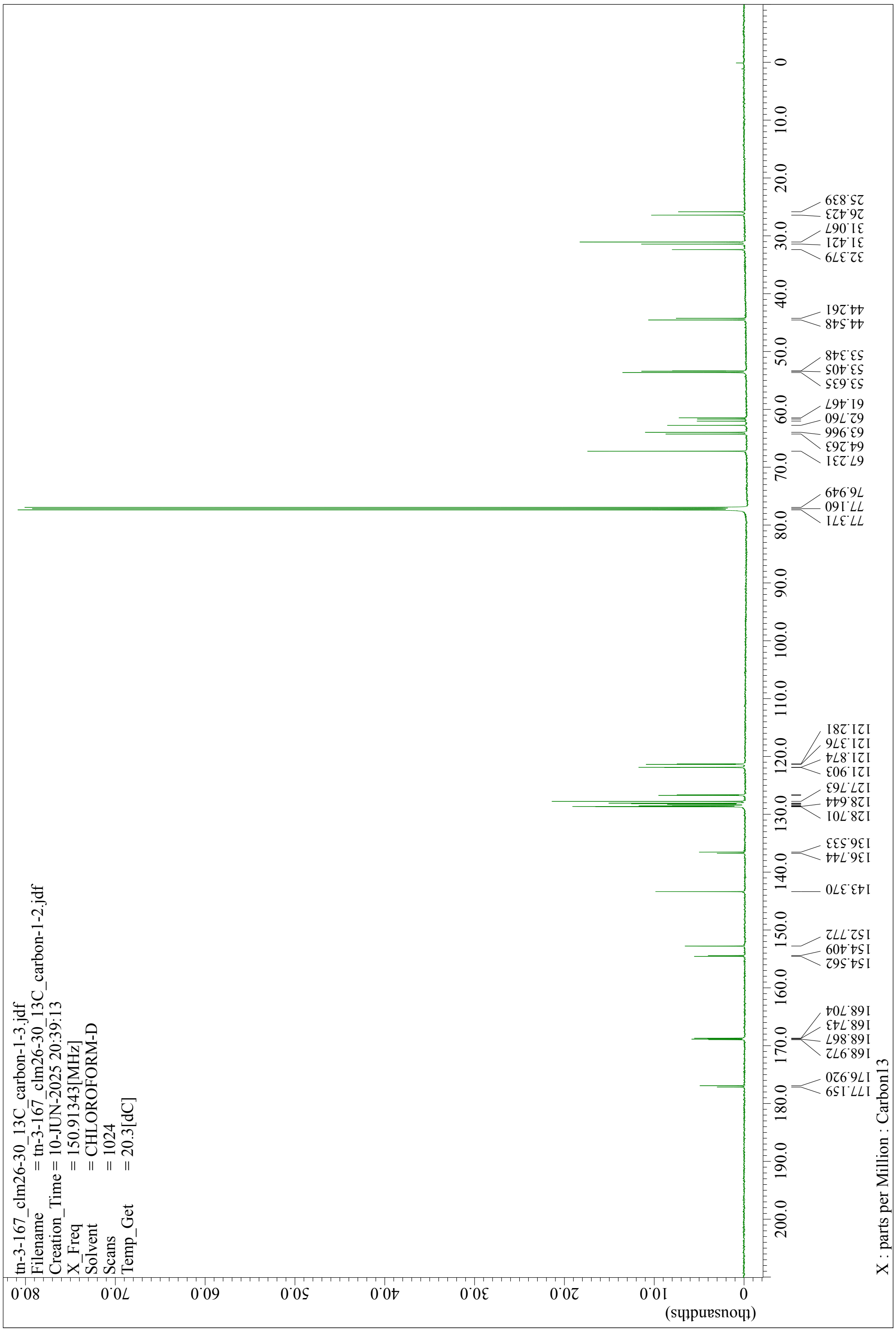

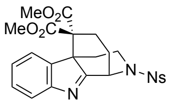

8d

^{1}H NMR ($CDCl_3$, 600 MHz)

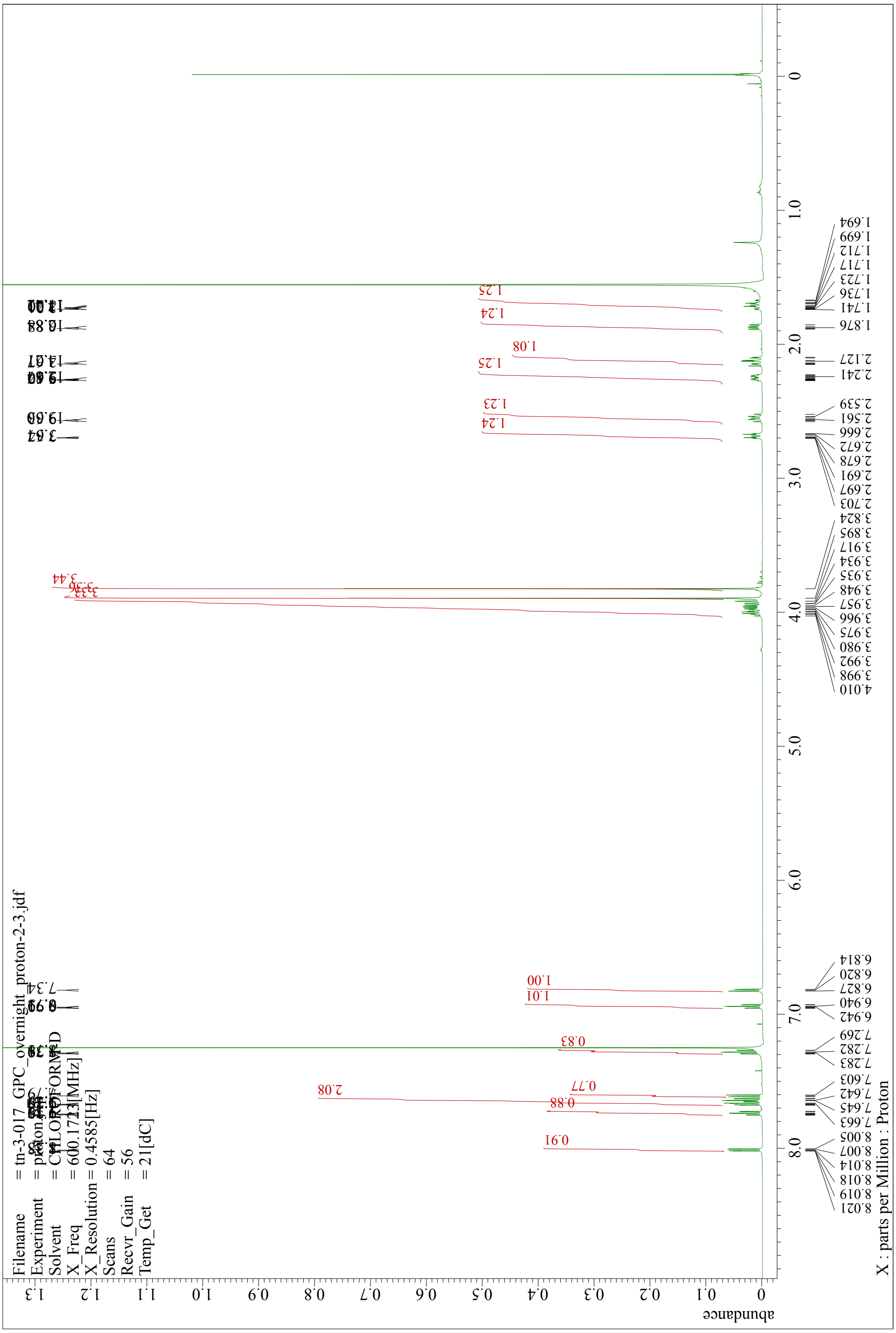

$^{13}C\{^{1}H\}$ NMR ($CDCl_3$, 150 MHz)

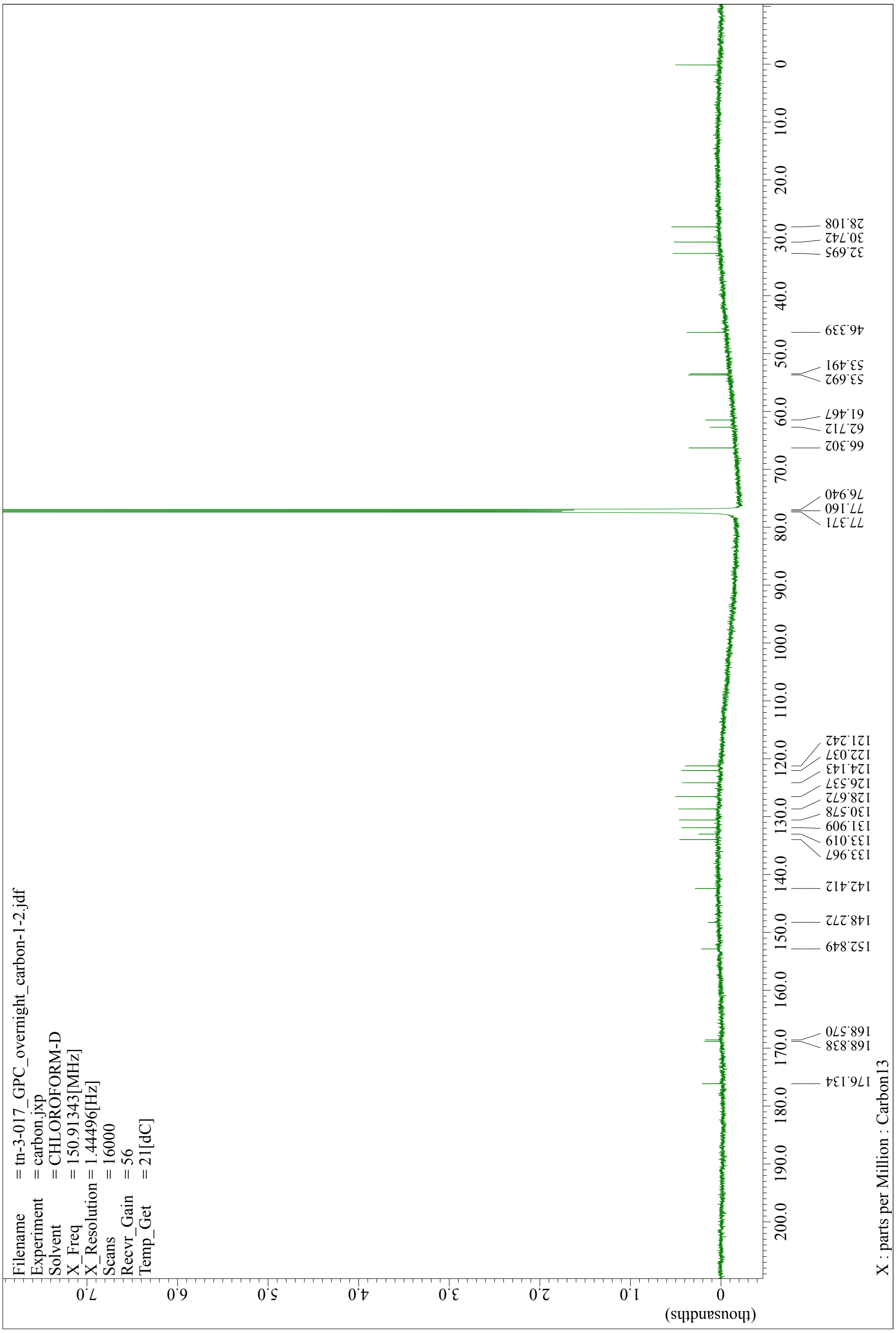

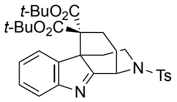

8e

^{1}H NMR ($CDCl_3$, 600 MHz)

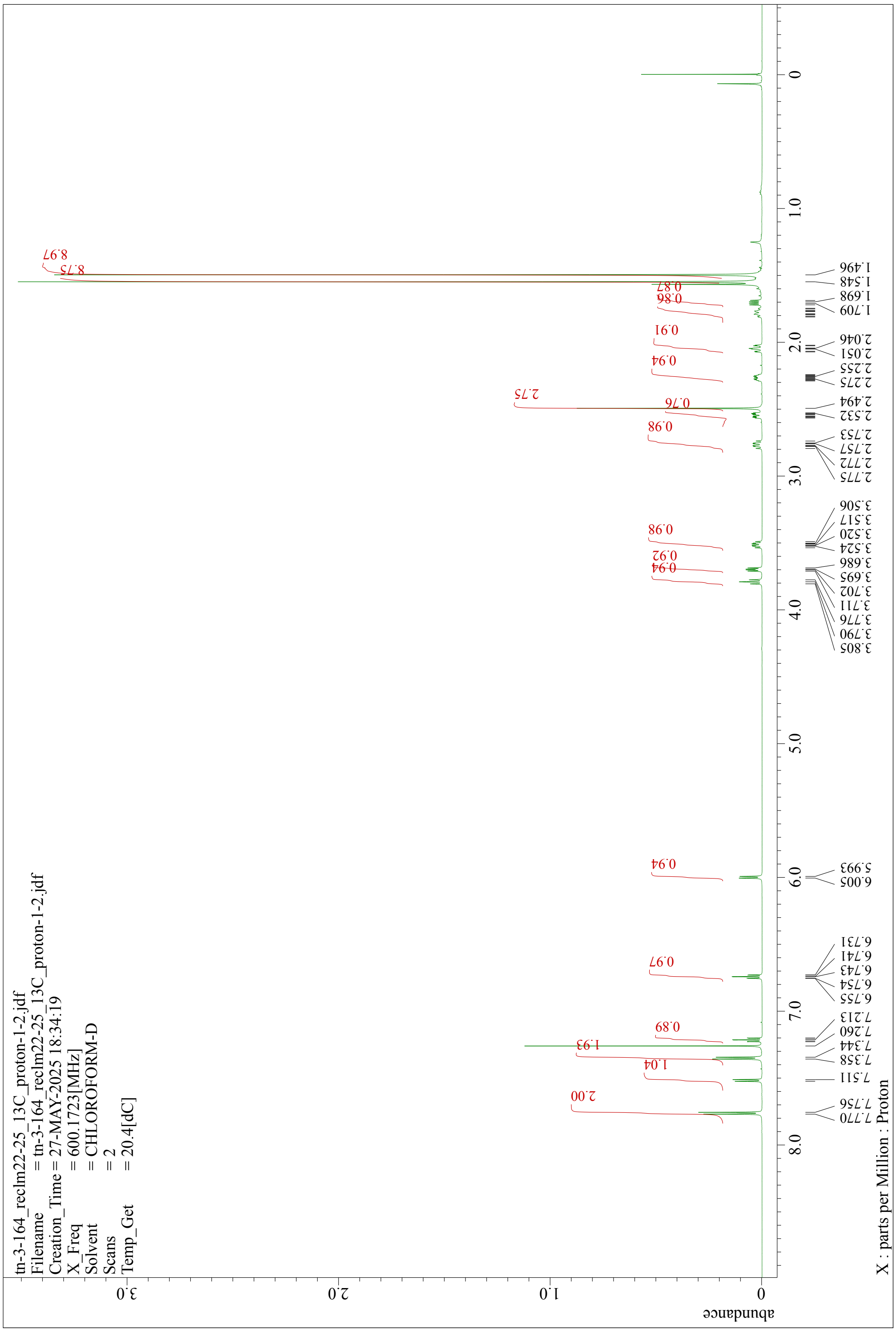

$^{13}C\{^1H\}$ NMR ($CDCl_3$, 150 MHz)

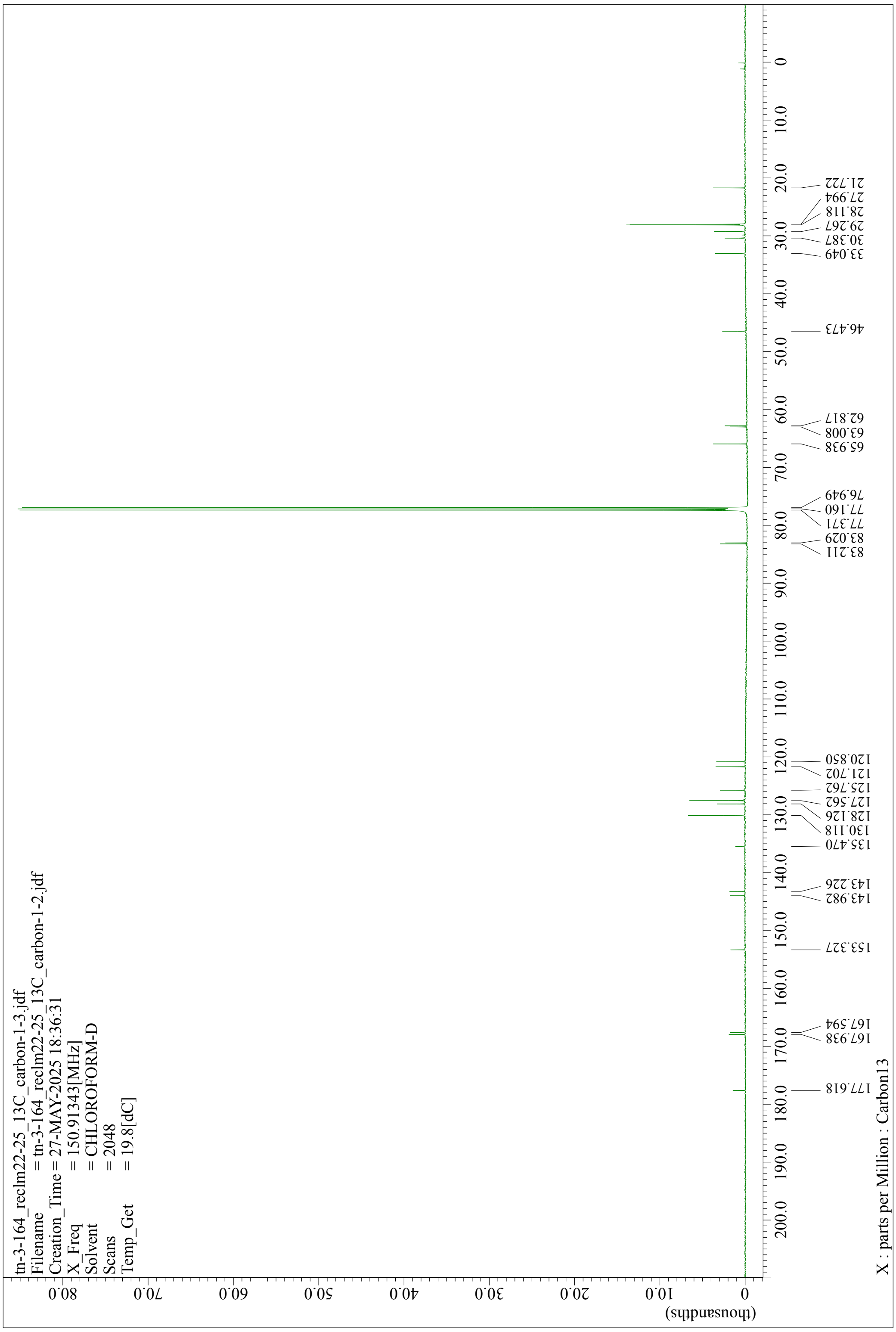

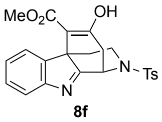

8f

^{1}H NMR ($CDCl_3$, 600 MHz)

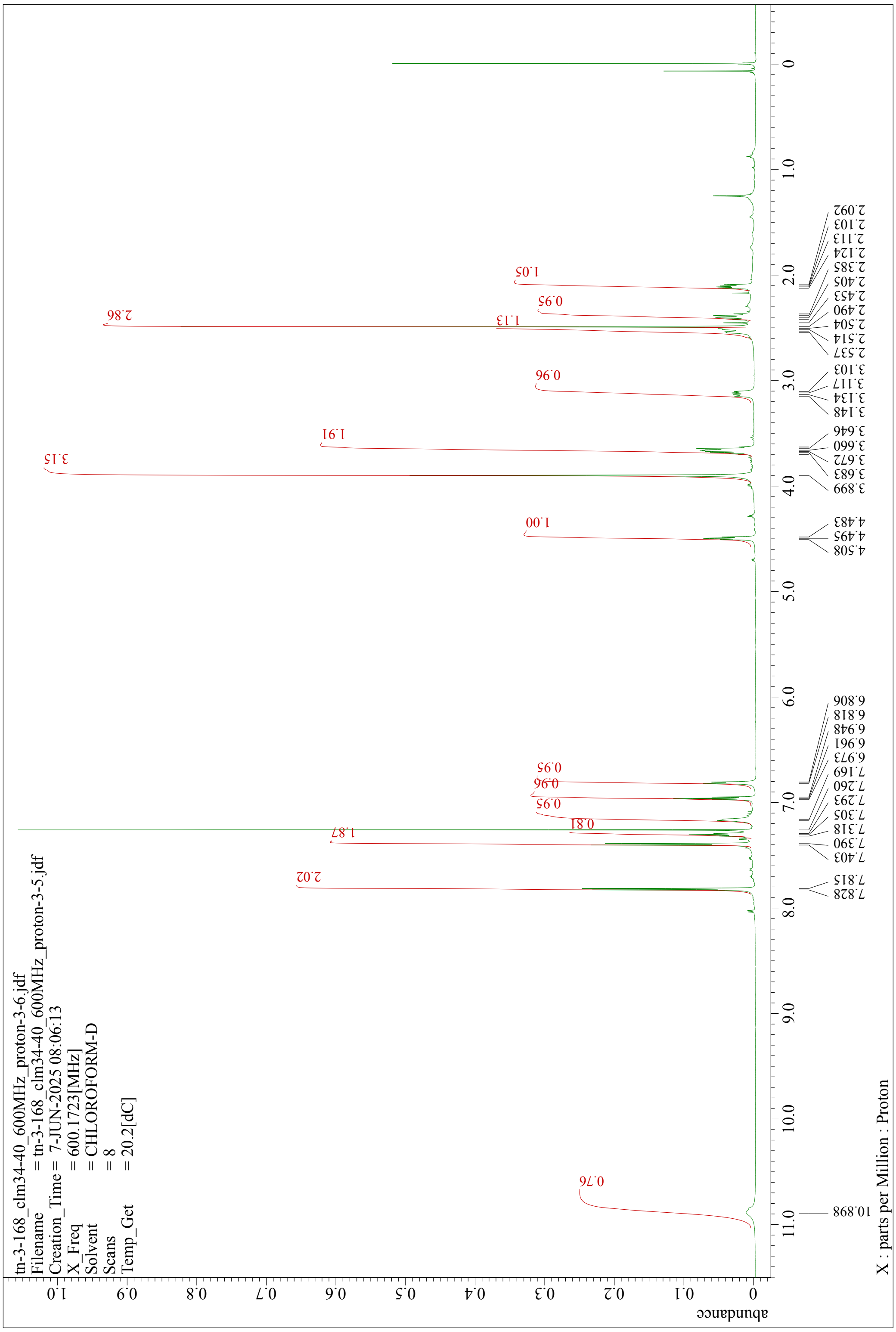

$^{13}C\{^{1}H\}$ NMR ($CDCl_3$, 150 MHz)

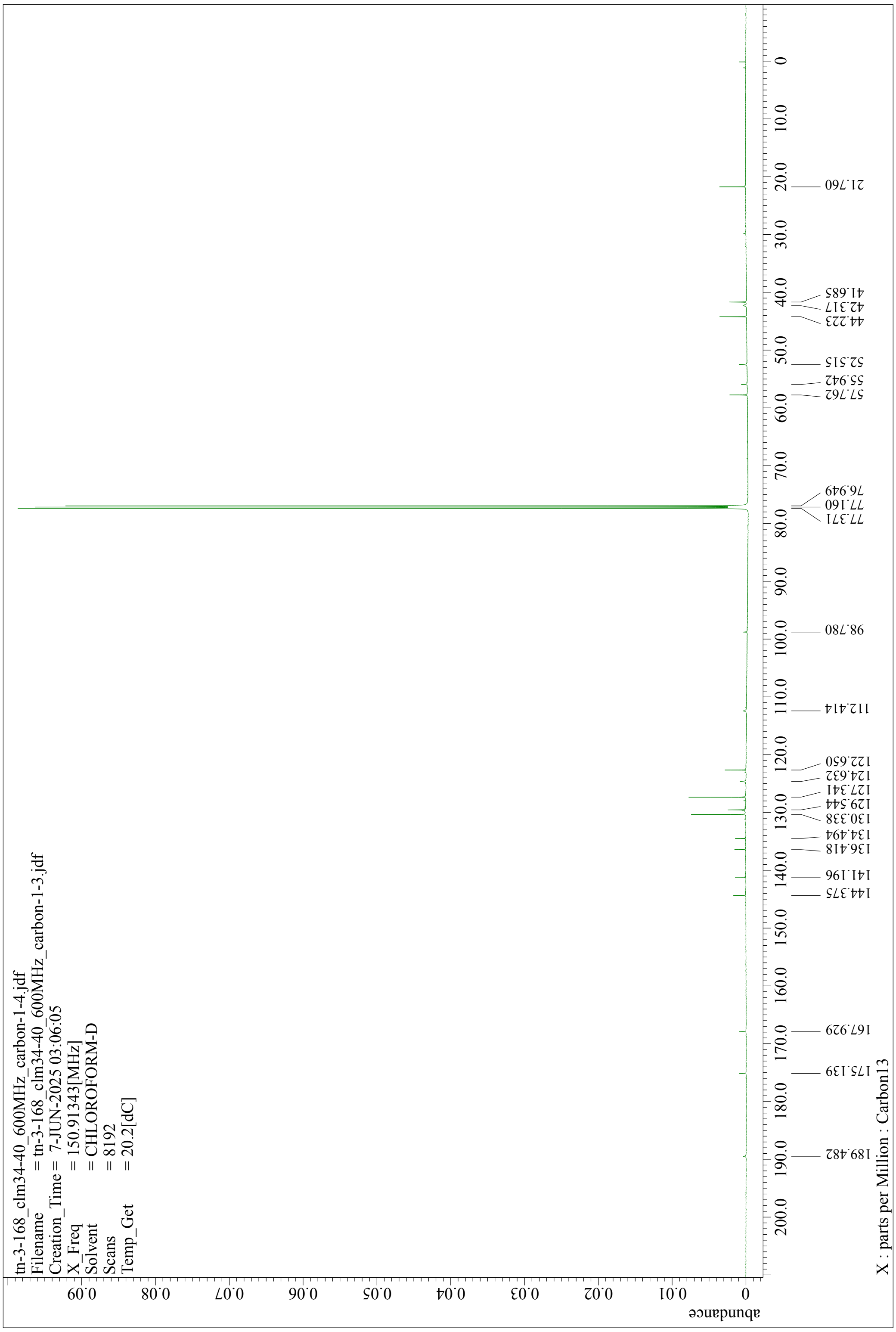

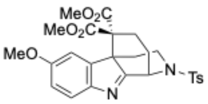

8g

^{1}H NMR ($CDCl_3$, 600 MHz)

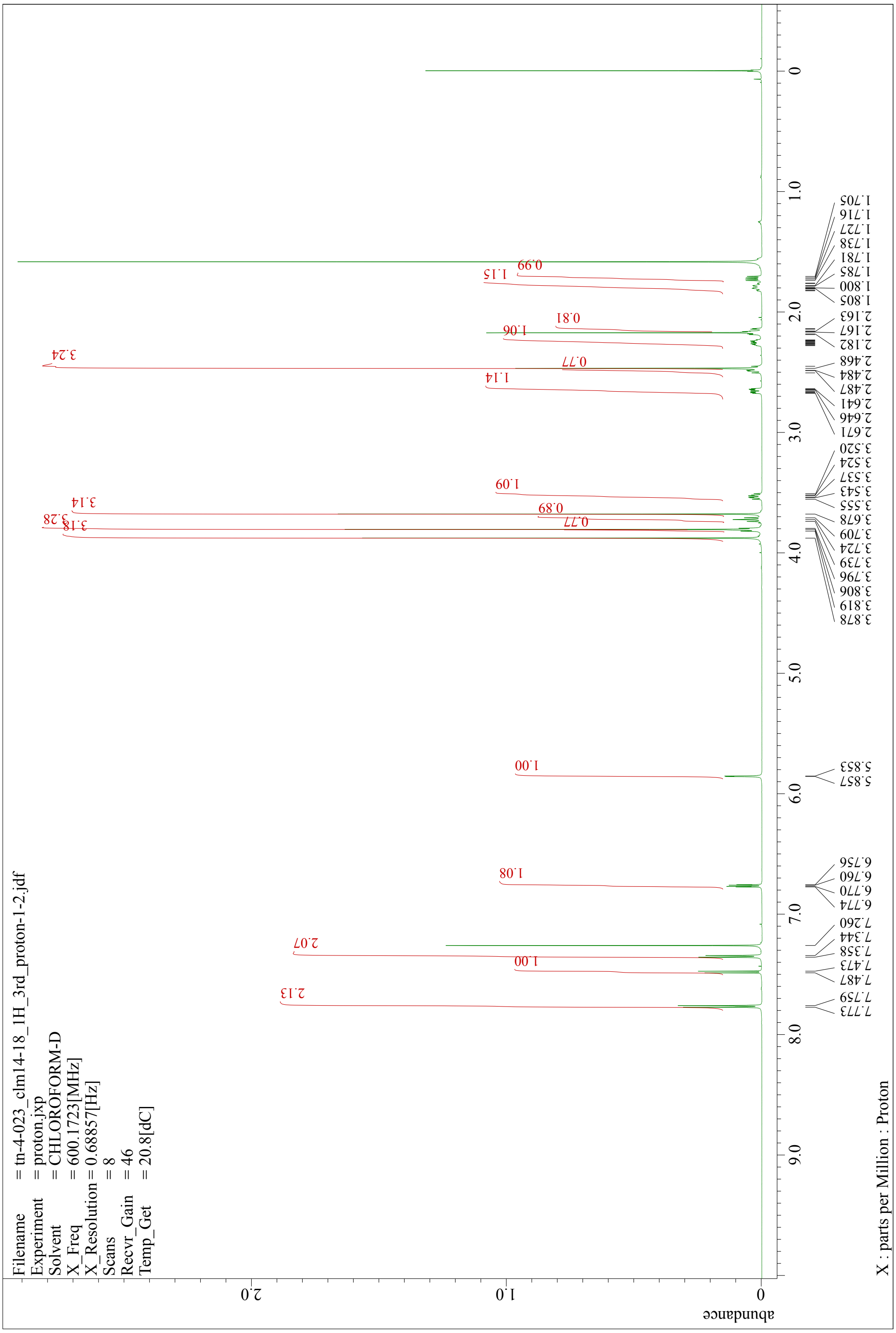

$^{13}C\{^{1}H\}$ NMR ($CDCl_3$, 150 MHz)

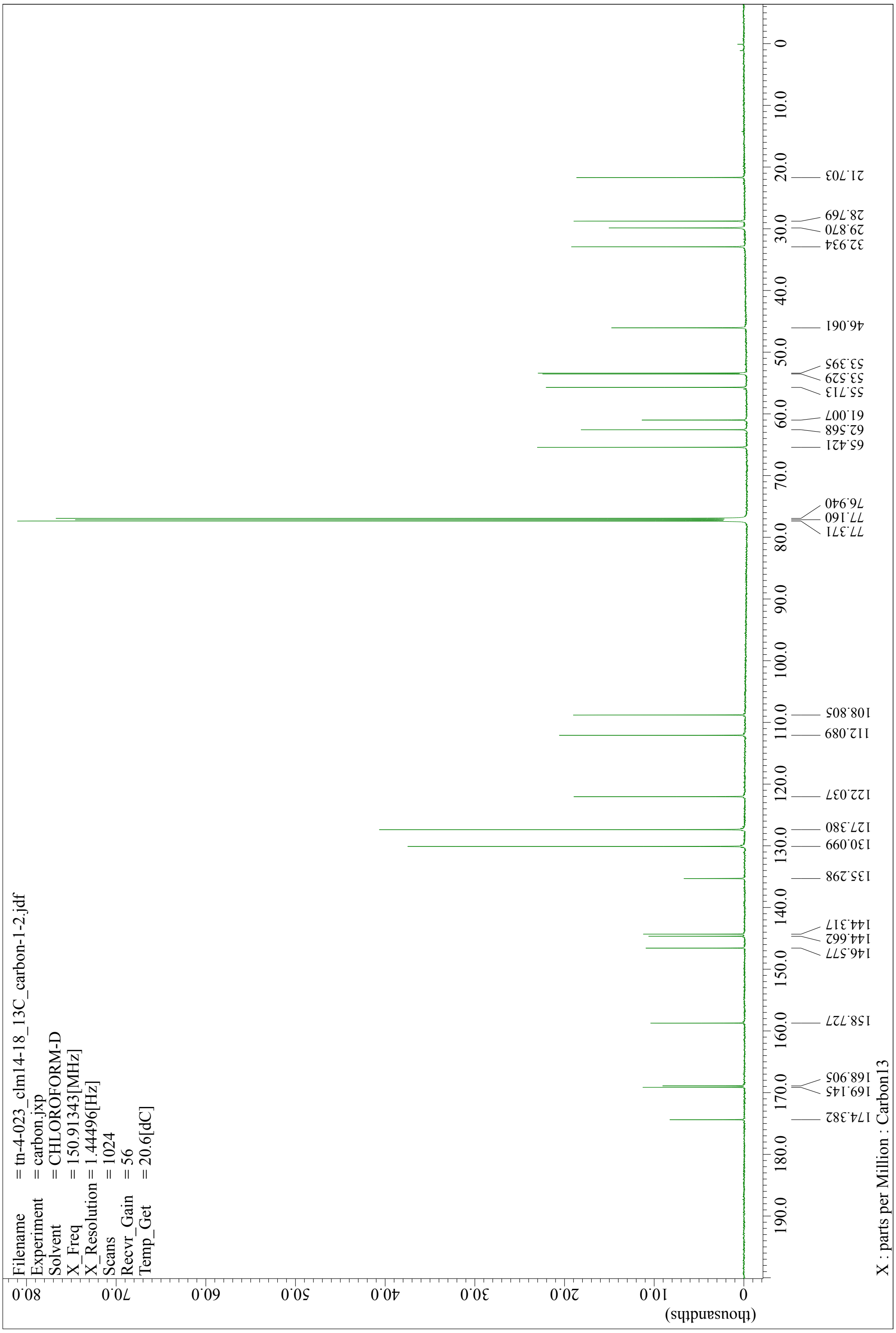

Supplement: Supplementary file 1 [file ol5c03645_si_001.pdf]
